# Pressure support and positive end-expiratory pressure versus T-piece during spontaneous breathing trial in difficult weaning from mechanical ventilation: study protocol for the SBT-ICU study

**DOI:** 10.1186/s13063-022-06896-4

**Published:** 2022-12-12

**Authors:** Mehdi Mezidi, Hodane Yonis, Louis Chauvelot, William Danjou, François Dhelft, Alwin Bazzani, Mehdi Girard, Laurent Bitker, Jean-Christophe Richard

**Affiliations:** 1 Medical Intensive Care Unit, Hospices Civils de Lyon, Croix-Rousse Hospital, Lyon, France; 2 Université de Lyon, Université Lyon 1, Lyon, France; 3 CREATIS INSERM U1294 CNRS UMR 5220, Villeurbanne, France

**Keywords:** Critical care, Spontaneous breathing trial, T-piece, Positive end-expiratory pressure, Difficult weaning

## Abstract

**Background:**

Spontaneous breathing trials are performed in critically ill intubated patients in order to assess readiness to be weaned from mechanical ventilation. In patients with difficult weaning (i.e. not extubated after their first SBT), performing SBT using pressure support with or without positive end-expiratory pressure or using T-piece is debated. As ventilatory support during SBT is greater on pressure support than on T-piece and as positive end-expiratory pressure can prevent weaning-induced pulmonary oedema, we hypothesized that their combination and large use of post-extubation non-invasive ventilation may shorten the time until successful extubation as compared with T-piece, without increasing the rate of reintubation.

**Methods:**

SBT-ICU is a monocentric prospective open labelled, randomized controlled superiority trial comparing two mechanical ventilation weaning strategies; i.e. daily spontaneous breathing trials using pressure support with positive end-expiratory pressure or T-piece. The primary outcome will be time until successful extubation (defined by as extubation, without reintubation or death within the seven following days).

**Discussion:**

This paper describes the protocol of the SBT-ICU trial. Enrolment of patients in the study is ongoing.

**Trial registration:**

ClinicalTrials.gov NCT03861117. Registered on March 1, 2019, before the beginning of inclusion.

**Supplementary Information:**

The online version contains supplementary material available at 10.1186/s13063-022-06896-4.

## Administrative information

Note: the numbers in curly brackets in this protocol refer to SPIRIT checklist item numbers. The order of the items has been modified to group similar items (see http://www.equator-network.org/reporting-guidelines/spirit-2013-statement-defining-standard-protocol-items-for-clinical-trials/).

**Table:** 

Title {1}	Pressure Support and Positive End-Expiratory Pressure versus T-piece during Spontaneous Breathing Trial in difficult weaning from mechanical ventilation. Study protocol for the SBT-ICU study
Trial registration {2a and 2b}.	Registered on ClinicalTrials.gov under identifier NCT03861117
Protocol version {3}	Version n°5, September 17, 2021
Funding {4}	Hospices Civils de Lyon
Author details {5a})	Medical Intensive Care, Hôpital de la Croix-Rousse, Hospices civils de Lyon
Name and contact information for the trial sponsor {5b}	Alexandre Pachot. Direction de la Recherche Clinique. Hospices Civils de Lyon. Lyon, FRANCE
Role of sponsor {5c}	The study funder had no part in study design; collection, management, analysis and interpretation of data; writing of the report; and in the decision to submit the report for publication. The study funder had no ultimate authority over any of these activities.

## Introduction

### Background and rationale {6a}

Invasive mechanical ventilation is applied in approximately 40% of critical care patients [1]. In order to wean the patient from the ventilator, the ability of the patient to breathe without mechanical support is assessed through spontaneous breathing trials (SBT).

SBT aims to reproduce a work of breathing close to the one the patient will have after extubation. Two main types of SBT are used commonly for critically ill patients: SBT with pressure support (SBT-PS) and SBT with a T-piece (SBT-TP). SBT-PS consists to lower the amount of pressure support (<10 cmH_2_O) with or without the use of positive end-expiratory pressure (PEEP). On the other hand, SBT-TP is done by disconnecting the patient from the ventilator and connecting the endotracheal tube to a T-piece to administer oxygen (if needed) while providing no ventilatory support. The type of SBT used was highly heterogeneous in a large international observational study [1]. However, it was recently recommended that the initial SBT should be conducted with pressure support rather than T-piece for hospitalized patients ventilated more than 24 h, although with a moderate quality of evidence [2]. Indeed, SBT-PS might be associated with a higher rate of success as compared to SBT-TP, according to a meta-analysis [3]. This may be explained by a lower work of breathing with SBT-PS than SBT-TP favouring earlier extubation [4].

The best SBT modality is still debated in difficult-to-wean patients, i.e. patients not extubated after their first SBT [1]. Recent data suggests that SBT-PS without PEEP could hasten successful extubation in patients at high risk of extubation failure [5]. Since weaning-induced pulmonary oedema (WIPO) is a frequent cause of SBT failure [6] and may be alleviated through the use of PEEP, a higher proportion of patients might succeed the SBT by combining PEEP and pressure support, but might be at higher risk of extubation failure, justifying an increased use of prophylactic post-extubation non-invasive ventilation (NIV).

Hence, we hypothesized that the combination of PEEP and pressure support during SBT and protocolized use of post-extubation NIV may shorten the time until successful extubation as compared with T-piece and post-extubation NIV performed as recommended [2]. We hypothesized that failure of a SBT-TP after a successful SBT-PS test would better identify patients at high risk of extubation failure and should trigger the use of prophylactic post-extubation NIV, to avoid reintubation.

### Objectives {7}

Main objective is to evaluate whether an assisted weaning strategy based on SBT-PS with PEEP followed by an SBT-TP to identify patients at high risk of extubation failure and to trigger the use of prophylactic post-extubation NIV (assisted weaning strategy, intervention group) leads to a *shorter time to successful extubation* in difficult-to-wean patients (i.e. patients failing their first SBT-TP) as compared to a weaning strategy based solely on SBT-TP (unassisted weaning strategy, control group) and prophylactic post-extubation NIV based on international guidelines [2].

#### Secondary objectives:

1. To evaluate whether if an assisted weaning strategy leads to a *higher rate of first successful extubation* in difficult-to-wean patients as compared to an unassisted weaning strategy.
2. To evaluate whether if an assisted weaning strategy leads to a *shorter duration of invasive mechanical ventilation* in difficult-to-wean patients as compared to an unassisted weaning strategy.
3. To evaluate whether if an assisted weaning strategy leads to a *shorter duration* of *mechanical ventilation* (i.e. invasive and non-invasive) in difficult-to-wean patients as compared to an unassisted weaning strategy.
4. To evaluate whether if an assisted weaning strategy leads to *more ventilator-free days (VFD) at day 28 and at day 90*, respectively, in difficult-to-wean patients as compared to an unassisted weaning strategy.
5. To evaluate whether if an assisted weaning strategy leads to a *shorter intensive care unit stay* in difficult-to-wean patients as compared to an unassisted weaning strategy.
6. To evaluate whether if an assisted weaning strategy leads to a *shorter hospital stay* in difficult-to-wean patients as compared to an unassisted weaning strategy.
7. To evaluate whether if an assisted weaning strategy leads to a *lower mortality in intensive care unit (ICU)*, *mortality at day 28 and mortality at day 90*, respectively, in difficult-to-wean patients as compared to an unassisted weaning strategy.
8. To evaluate whether if an assisted weaning strategy leads to a *higher reintubation rate* in difficult-to-wean patients as compared to an unassisted weaning strategy.

### Trial design {8}

The study is a monocentric prospective open labelled, randomized controlled superiority trial, with two parallel groups and balanced randomization with a 1:1 ratio.

#### Methods: participants, interventions and outcomes

The second version of this protocol (Additional file 1) was published on February 7th, 2019, before the inclusion of the 1st patient in the study. The current version is the fifth version of the protocol, which was accepted by the research ethics committee on September 17th, 2021 (Additional file 2). Modifications between version 2 and 5 are summarized in Table 1. The WHO Trial Registration Data Set is provided in Additional file 3.

**Table 1:** Summary of protocol versions

Version	Date	Comment
1	January 11th, 2019	Initial version submitted to research ethical committee
2	February 7th, 2019	Modifications following research ethical committee comments regarding SAE definition.
3	June 25th, 2019	Addition of the possibility to include patients unable to write due to physical disability
4	July 19th, 2019	Modifications following research ethical committee comments (refusal of next of kin consent by phone)
5	September 17th, 2021	Extension of recruitment period Sponsor contact information update Update on archive duration, according to French law

### Study setting {9}

The study is conducted in one ICU located in a French academic hospital.

### Eligibility criteria {10}

Inclusion criteria:

- Patient aged 18 years or older
- Intubated and ventilated in the intensive care unit for more than 24 h
- Patient ready for weaning evaluation (Table 2)
- Failure of a first SBT-TP (Table 3)

**Table 2:** Patient weaning readiness criteria

Hemodynamic criteria	Norepinephrine < 1mg/h Dobutamine ≤ 5μg/kg/min
Respiratory criteria	FiO_2_ ≤ 50% SpO_2_ ≥ 88% PEEP ≤ 5 cmH_2_O Respiratory rate ≤ 35/min
Neurological criterion	Obedience to simple command

All criteria must be fulfilled to assess weaning readiness status

*FiO*_*2*_ inspired oxygen fraction, *PEEP* positive end-expiratory pressure, *SpO*_*2*_ peripheral oxygen saturation

**Table 3:** Spontaneous breathing trial (SBT) failure criteria

**•** Variation of 20% of heart rate from pre-SBT value **•** Variation of 20% of systolic arterial pressure from pre-SBT value **•** Respiratory rate >35/min **•** SpO_2_<88% **•** Sweating, agitation, conscience alteration **•** Signs of respiratory distress: increased accessory muscle activity, facial signs of distress, dyspnea **•** Arterial pH<7.35 and PaCO_2_>45 mmHg	

*PaCO*_*2*_ Partial pressure of carbon dioxide in arterial blood, *SpO*_*2*_ Peripheral oxygen saturation

Exclusion criteria:

- Chronic neuromuscular disease
- Guillain-Barré Syndrome
- Central nervous system disease with consciousness disorder (i.e. inability to obey a simple command)
- Tracheostomy
- Chronic disease with life expectancy less than 1 year
- Pregnancy, breastfeeding
- Withholding life support regarding a reintubation
- Prisoner or patient interned in a psychiatric hospital
- Under legal protective measures
- Language barrier
- Lack of social security
- Lack of the patient’s consent (or of the next of kin where appropriate)
- Patient under an exclusion period after enrollment in another research study

### Who will take informed consent? {26a}

Before inclusion in the trial, written inform consent from the patient will be sought by investigators.

If the patient is unable to write due to physical disability, a witness independent from the investigators will attest that the patient has received a correct information and has consented to be included in the study (since protocol version 4).

If the patient is unable to give informed consent, written consent of patient’s legal representative will be sought by investigators. In that case, patient written inform consent will be sought as soon as its medical condition allows this procedure.

### Additional consent provisions for collection and use of participant data and biological specimens {26b}

Potential future studies intending unplanned use of the data generated in this trial will require an additional consent of included patients. Unplanned use of biological specimens will not be performed.

### Interventions

#### *Explanation for the choice of comparators* {6b}

The control group will perform SBT-TP to evaluate their capacity to be weaned. This modality of weaning is still commonly used in clinical practice, more commonly than SBT-PS [1], and the superiority of any of these techniques is controversial in difficult-to-wean patients.

#### *Intervention description* {11a} *(Fig.**1**)*

The interventions will be started within the 6 h following inclusion.

**Fig. 1:**
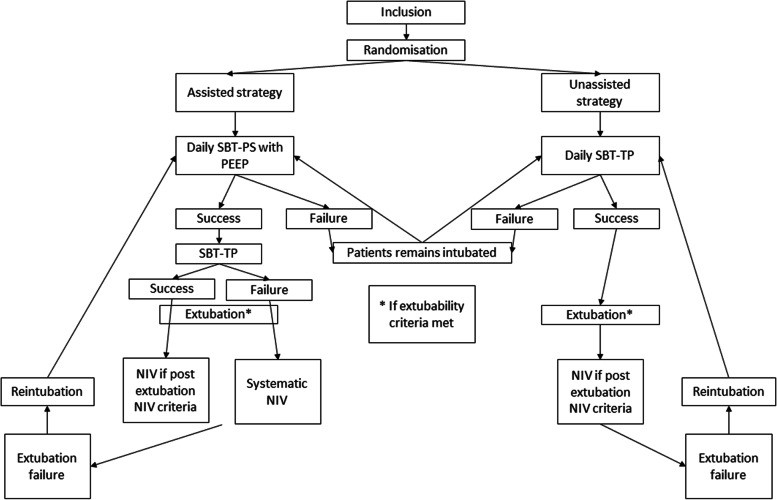
Description of interventions according to allocated group. SBT-PS with PEEP: spontaneous breathing trial with pressure support and positive end-expiratory pressure. SBT-TP with PEEP: spontaneous breathing trial with T-piece. NIV: non-invasive ventilation. Post-extubation NIV criteria were: age>65 years, chronic heart or respiratory failure, PaCO_2_ >45 mmHg during SBT, chronic obstructive pulmonary disease (COPD)

##### Intervention group (assisted weaning strategy)

While intubated and being able to perform a SBT (Table 2) [2, 7, 8], patients will perform a daily SBT with pressure support 7 cmH_2_O and PEEP 5 cmH_2_O during 30 min to assess readiness to be weaned from mechanical ventilation. This level of pressure support was chosen to standardize the intervention in the range recommended by international guidelines [2]. Inspired oxygen fraction (FiO_2_) is set between 21 and 50% in order to target a peripheral oxygen saturation (SpO_2_) between 94 and 98% (or between 88 and 92% for chronic obstructive pulmonary disease (COPD)/chronic respiratory failure (CRF) patients). SBT success is defined by the ability to perform the SBT-PS during 30 min without failure criteria (Table 3). In case of SBT failure, patient is switch back to previous respiratory parameters. In case of SBT success, an additional SBT-TP will be performed after 30 min of rest, for a maximal duration of 30 min, to further assess the risk of respiratory failure at atmospheric pressure. The modalities of the SBT-TP are the same as the control group (see below). If the patient meets extubability criteria on SBT-PS (see below), it will be extubated 2 to 3 h after resuming ventilation and will receive post-extubation NIV unless contraindicated [9] if SBT-TP was a failure or if meeting at least one of the following post-extubation prophylactic NIV criteria [2]: age > 65 years, chronic heart failure (CHF), CRF, carbon dioxide partial pressure in arterial blood (PaCO_2_) > 45 mmHg during SBT, or COPD.

##### Control group (unassisted weaning strategy)

While intubated and being able to perform a SBT (Table 2), patients will perform daily a 30-min SBT by disconnecting the patient from the ventilator and using a T-piece to administer 0 to 10 L/min oxygen targeting a peripheral oxygen saturation (SpO_2_) between 94 and 98% (or between 88 and 92% for COPD/CRF patients). SBT success is defined by the ability to perform the SBT-TP during 30 min without failure criteria (Table 3). In case of SBT failure, patient is switch back to previous respiratory parameters. If the patient succeeds the SBT-TP and meets extubability criteria, it will be extubated 2 to 3 h after resuming ventilation and will receive post-extubation NIV if meeting post-extubation NIV criteria (cf. supra) [2] unless contraindicated [9].

For both groups, extubability criteria will be the following (Additional file 4): cough strength (Semi-quantitative Cough Strength Score (SCSS) 3, 4 or 5 [10]), abundance of respiratory secretions score ≤2 [11], no planned surgery in the next 24 h and absence of suspicion for post-extubation laryngeal oedema. If the patient presents at least 3 extubability criteria and succeeds the SBT, the patient will be extubated.

If indicated, post-extubation prophylactic NIV will be performed per sessions of at least 1 to 2 h every 3 h, with a minimum of 8 h per day. If well tolerated during sleep, NIV may be performed during the whole night. Post-extubation NIV will be applied for at least 24 h, and possibly pursued per decision of the attending physician.

Post-extubation manual or instrumental respiratory physiotherapy will be provided in both groups with modalities decided by the clinician and respiratory physiotherapist and recorded in the case report forms. Post-extubation high-flow nasal cannula oxygen will be discouraged. Post-extubation rescue NIV will be allowed only if WIPO or hypercapnic failure for COPD/CRF patients is suspected.

Reintubation criteria will be the following: [1] respiratory failure defined by occurrence of at least one of the following criteria: respiratory rate>40/min, signs of respiratory distress, copious respiratory secretions, pH<7.35 with PaCO_2_>45 mmHg, SpO_2_<90% or oxygen partial pressure in arterial blood (PaO_2_) <60 mmHg under inspired oxygen fraction (FiO_2_) ≥ 60% or O_2_ 10 L/min [2]; coma (defined as coma Glasgow scale < 8) [3]; hemodynamic failure defined as de novo norepinephrine or epinephrine dose >1 mg/h or new onset of lactic acidosis.

In case of self-extubation, patients will be managed similarly to patients with scheduled extubation.

The use of tracheostomy for respiratory weaning is not encouraged in this study. If the clinician in charge decides to tracheostomize a patient, weaning management according to study allocation will cease but the subject will be analysed according to its study group. Cessation of invasive mechanical ventilation in this specific population will be defined as cessation of ventilatory support on the tracheostomy tube for at least 7 days.

### Criteria for discontinuing or modifying allocated interventions {11b}

Allocated interventions are discontinued if:

- The patient is extubated and without reintubation within the seven following days
- A withdrawal of life support or withholding of life support (no reintubation decision) is decided by the clinician
- The patient is discharged of ICU
- A severe adverse event related to the intervention occurs
- 90 days after the inclusion of the trial
- The patient is transferred to another ICU
- The patient or its surrogate withdraws consent
- The patient is tracheostomized

### Strategies to improve adherence to interventions {11c}

Adherence to study interventions will be checked daily by the investigators during staff with the clinicians. A document summarizing the study protocol (Additional file 5) will be provided to the investigators.

### Concomitant relevant care permitted or prohibited during the trial {11d}

During the trial participation, the patient cannot be included in a study related to weaning from mechanical ventilation. Tracheostomy and post-extubation high-flow nasal oxygen are not encouraged in the study but not strictly prohibited.

### Provisions for post-trial care {30}

None

### Outcomes {12}

#### Primary outcome

The primary outcome will be time between study inclusion and successful extubation. Successful extubation will be defined by the absence of reintubation or death within the 7 days following extubation.

#### Secondary outcomes

1. Rate of first successful extubation after *inclusion* defined as the ratio of the number of patients successfully extubated after their first extubation over the total number of patients per group.
2. Invasive mechanical ventilation duration (expressed in hours): total cumulative time spent on invasive mechanical ventilation since inclusion per group. Each time period spent on invasive mechanical ventilation from inclusion until successful extubation are summed up. Patients not experiencing successful extubation will be censored at day 90 or time of death (if earlier than day 90).
3. Mechanical (invasive and non-invasive) ventilation duration (expressed in hours): total cumulative time spent on invasive and non-invasive mechanical ventilation since inclusion per group.

- If the patient successfully is extubated with post-extubation NIV: date/time of NIV cessation minus date/time of inclusion (or date/time of ICU discharge if NIV not ceased at ICU discharge).
- If the patient is successfully extubated without post-extubation NIV: date/time of successful extubation minus date/time of inclusion.
- Patients not experiencing successful extubation will be censored at day 90 or time of death (if earlier than day 90).

(4) VFD at day-28 and day-90. VFD will be computed as follows from the day of inclusion:
  VFD = 28−*x* for VFD at day 28 or VFD = 90−*x* for VFD at day 90 if the patient is successfully weaned from invasive mechanical ventilation, with *x* being the number of days from *inclusion* to last successful extubation. Successful weaning from mechanical ventilation will be defined as extubation without reintubation within at least 7 days (or weaning from mechanical ventilation for at least 7 days for patients with tracheostomy).
  VFD = 0 if the patient dies between inclusion and day 28 for VFD at day 28, or if the patient dies between inclusion and day 90 for VFD at day 90.
  VFD = 0 if the patient is mechanically ventilated for more than 28 days after inclusion for VFD at day 28, or more than 90 days after *inclusion* for VFD at day 90.
(5) ICU stay defined as ICU length of stay in days between *inclusion* and ICU discharge.
(6) Hospital stay defined as hospital length of stay in days between *inclusion* and hospital discharge (to home or rehabilitation facility).
(7) ICU mortality, day 28 mortality and day 90 mortality defined as the ratio of the number of death over each period over the total number of patients per group.
(8) Reintubation rate defined as the number of patients with any reintubation divided by the number of patients per group.

Minor modifications occurred to secondary outcomes 1, 5, 6 and 8, after beginning of the trial, in order to improve reproducibility with comparable studies. For secondary outcomes 1 and 8, the denominator was changed from total number of patients extubated to total number of patients per group. For outcomes 5 and 6, length of stay was computed starting from study inclusion instead of ICU admission.

### Participant timeline {13}

Participant timeline is summarized in Table 4.

**Table 4:** Participant timeline

	Inclusion (day 1)	Day 2 to day 90	ICU discharge / death	Day 28	Day 90
Demographic data	X				
Patient weaning readiness	X	X^a^			
SBT result	X	X^a^			
Arterial blood gas	X	X^a^			
*If SBT success*					
Cough score	X	X^a^			
Abundancy of respiratory secretions	X	X^a^			
Extubability criteria	X	X^a^			
*If extubation*					
Extubation date and time	X^b^	X^b^			
Extubation type (protocolized/self-extubation)	X^b^	X^b^			
Post-extubation NIV characteristics	X^b^	X^b^			
Respiratory physiotherapy characteristics	X^c^	X^c^			
High-flow nasal oxygen use	X	X^c^			
Invasive mechanical ventilation status	X	X^c^			
*If reintubation*					
Reintubation criteria	X	X			
*If tracheostomy performed*					
Characteristics of tracheostomy			X		
*Follow-up data*					
Date/hour of cessation of invasive ventilation and NIV			X		
End of study					
Respiratory status			X	X	X
Mortality			X	X	X

^a^As long as the patient is intubated or tracheostomized

^b^On the day of extubation and the day after

^c^During the 7 days following extubation

*NIV* Non-invasive ventilation, *SBT* Spontaneous breathing trial

### Sample size {14}

In a pilot observational study on 88 patients performed in our ICU (unpublished data), median time between first SBT and extubation was 22 h with a strategy using SBT-PS and PEEP. We hypothesized that a between-arm difference of at least 24 h would be clinically relevant to prefer a SBT strategy over one another. With an alpha risk of 5%, a power of 80% and a hazard ratio of 2 (based on the ratio of median duration until successful extubation), a total of 66 successful extubations would be needed. Accounting for the expected mortality and weaning failure rates (evaluated at 30%), we plan to include 94 patients (47 per group).

### Recruitment {15}

In order to achieve recruitment, all the clinicians of the ICU received a detailed information about the study. All intubated patients will be screened daily to evaluate study eligibility.

## Assignment of interventions: allocation

### Sequence generation {16a}

Allocation sequence will be computer-generated with stratification into 3 strata:

- Patients with CHF (defined by an left ventricular ejection fraction < 45%)
- Patient with suspected or proven COPD
- Other patients

COPD is suspected in patients older than 40 years, with clinical features (dyspnea, chronic cough, respiratory secretions) and/or COPD risk factor (tobacco use, personal or professional exposure to fumes). COPD is proven by forced expiratory volume in 1 s (FEV_1_)/forced vital capacity (FVC) ratio postbronchodilatators less than 70%.

In case of concomitant CHF and COPD, patient will be stratified in COPD strata.

Randomization will be performed in each stratum, using random blocks of size 4, 6 and 8.

### Concealment mechanism {16b}

Allocation sequence will be concealed in a sealed opaque envelope with sequential number in each stratum. Envelope number was recorded in the case report form.

### Implementation {16c}

Clinicians and investigators are enrolling patients and assigning patients to intervention according to the allocation defined by the randomization.

## Assignment of interventions: blinding

### Who will be blinded {17a}

Due to the type of intervention, investigators and patients cannot be blinded from group allocation.

Data analysts will be blinded from group allocation, although this may be deducted from the additional SBT-TP performed in the interventional group and post-extubation NIV criteria variables in the dataset.

### Procedure for unblinding if needed {17b}

Due to the open design of the study, there is no unblinding procedure for care providers. After recording of the main outcome criterion of the last included patient into the CRF, a quality control will be performed on the database with blinding of study arm. Statistical analyses will begin after database lock.

## Data collection and management

### Plans for assessment and collection of outcomes {18a}

Assessment and collection of outcomes will be done by the clinicians in charge of the patients. Case report forms are provided in Additional files 6 and 7. Case report forms are then entered in database with range checks. In case of obviously wrong data, investigators can check the electronical medical file of the patient. Subjects will be assessed daily while hospitalized in the intensive care unit (ICU). Day 28 et day 90 assessment will be performed by investigators or delegated team members using electronic medical records, and phone call to the patient’s general practitioner and to any MD involved in patient care after ICU discharge if required.

### Plans to promote participant retention and complete follow-up {18b}

Missing values for primary and secondary outcomes assessed during ICU stay are not expected, since patients will remain hospitalized. Missing values for survival and respiratory status at day 28 or day 90 could occur for patients surviving at hospital discharge and the subsequent procedure will be applied to minimize the number of patients with incomplete follow-up. Upon enrolment, patient and their next of kin contact information will be stored in the digital health record at each study site. Patients will be contacted by phone call at day 28 and day 90 to assess vital and respiratory status. In case contact with the patient is lost, study technicians will contact the patient’s next of kin or the patient’s general practitioner in order to re-establish contact or assess vital and respiratory status.

### Data management {19}

Paper case report forms will be entered in excel sheets and stored in a secured location. To ensure correct text entry, range checks were implemented for all the variables.

### Confidentiality {27}

According to French law, case report forms are anonymized with the use of patient’s initials and month/year of birth instead of complete date of birth. A correspondence table is kept by the investigator in a separate location.

### Plans for collection, laboratory evaluation, and storage of biological specimens for genetic or molecular analysis in this trial/future use {33}

It is not planned to keep any biological specimen in this study.

## Statistical methods

### Statistical methods for primary and secondary outcomes {20a}

The statistical analysis plan [12] describing precisely the statistical methods is provided as Additional file 8. All the analyses will be carried out using R for Windows [13]. The comparisons will be considered statistically significant for a bilateral *p*-value < 0.05.

Intention-to-treat population will be all subjects who were randomized except patients secondary excluded from the study due to:

-Transfer to another ICU not participating to the study

-Consent withdrawal

-Lack of inclusion criteria or presence of exclusion criteria

All the analyses (including primary efficacy analysis) will be performed on this population.

## Descriptive analysis

Patients’ characteristics will be described in the two groups to verify allocation efficacy. Quantitative variables will be described using the following statistics: number of missing data, mean, standard deviation, quartiles, minimum and maximum values, number and rate of missing variables, and compared between groups with the Mann-Whitney test. Qualitative variables will be reported as absolute and relative frequencies, number and rate of missing variables and compared between groups with the chi-square test or the Fisher exact test. Univariable absolute difference will be reported for each variable as difference [95% confidence interval]. The Hodges-Lehmann method will be used to compute unbiased median differences for outcomes and their CI95%. For qualitative variables, 95% confidence interval will be computed through bootstrapping.

## Analysis of the primary outcome

The main analysis will be carried out by intention to treat, i.e. all the patients included in the study will be analysed in their initial randomization group regardless of whether the allocated ventilation strategy was effectively applied or not.

The primary outcome (time to successful extubation) will be analysed with a Cox model according to a cause-specific analysis [14, 15], using randomization strata as covariates, to estimate hazard ratio and CI95%. In case the proportional hazard assumption will not be fulfilled, an additional chronological covariate will be used in the Cox model or a log-rank test will be performed where appropriate.

To account for the competing risk of death, an additional sensitivity analysis will be performed on the main outcome. Cumulative incidence functions will be computed and analysed with the Fine and Grey competing risk regression model, using successful extubation as event and death as a competitive risk.

## Analysis of the secondary outcomes

The analysis of the secondary objectives will also be carried out by intention to treat. Qualitative secondary outcomes such as mortality or reintubation rate will be described in each group by the event proportion and compared using the chi-square test or the exact test of Fisher. Quantitative secondary outcomes such as the VFD will be described in each group by the mean and the standard deviation, median and quartiles and the minimum and maximum values. They will be compared between the two groups using the *t* test of Student or the test of Mann and Whitney. Univariable absolute difference will be reported for each variable as difference [95% confidence interval]. The Hodges-Lehmann method will be used to compute unbiased median differences for outcomes and their CI95%. For qualitative variables, 95% confidence interval will be computed through bootstrapping.

### Interim analyses {21b}

No interim analysis was planned owing to the small size of the study.

### Methods for additional analyses (e.g. subgroup analyses) {20b}

A subgroup exploratory analysis will be performed on the primary judgment criterion on the following subgroups:

- Randomization strata
- COVID status (COVID positive vs COVID negative)
- Bicarbonates on the day of extubation below vs. greater or equal to its median value
- PaCO_2_ at the end of SBT ≥ 45 mmHg vs < 45 mmHg, on the day of extubation
- Time between intubation and inclusion below vs. greater or equal to its median value
- Cough score below vs greater or equal to its median value

The study is not powered for subgroup analysis which should be considered exploratory.

### Methods in analysis to handle protocol non-adherence and any statistical methods to handle missing data {20c}

All analyses are done in intention to treat. Missing data per variable will be reported. No imputation is planned.

### Plans to give access to the full protocol, participant-level data and statistical code {31c}

Full protocol and anonymous participant-level data will be disclosed on reasonable request.

## Oversight and monitoring

### Composition of the coordinating centre and trial steering committee {5d}

No coordinating centre or steering committee are planned for this study, as the study is monocentric.

### Composition of the data monitoring committee, its role and reporting structure {21a}

No data monitoring committee is planned for this study, owing to its small size, and since procedures in both study arms are standard ICU procedures and not expected to be harmful.

### Adverse event reporting and harms {22}

Serious adverse event (SAE) information will be collected for the duration of the participant’s involvement in the trial. SAEs will be managed according to the best current standard of care and reported to the sponsor according to good clinical practices. All SAEs will be reported to the sponsor within one business day, in a structured narrative explaining the events that occurred. An internal safety monitor will adjudicate all SAEs for report completeness, seriousness of event and relationship to study interventions. After receiving an unexpected SAE report, the sponsor will notify the French regulatory agencies and the research ethics committee (CPP Ile de France VI). SAE will be reported in trial publication.

### Frequency and plans for auditing trial conduct {23}

One audit of trial conduct will be performed by the study sponsor (which is also the funder of the study).

### Plans for communicating important protocol amendments to relevant parties (e.g. trial participants, ethical committees) {25}

Important protocol amendments will be submitted for authorization to the ethical committee.

### Dissemination plans {31a}

Trial results will be published in a peer-reviewed medical journal.

Patients are offered to receive a summary of trial results.

## Discussion

Due to COVID-19 outbreak, inclusions were upheld from March to July 2020, following sponsor and health authority instructions.

### Trial status

This is the fifth version of the protocol, written on September 7, 2021. First patient was included on May 10, 2019. We expect to complete the recruitment by the end of 2022.

## Supplementary Information

**Additional file 1.**

**Additional file 2.**

**Additional file 3.**

**Additional file 4.**

**Additional file 5.**

**Additional file 6.**

**Additional file 7.**

**Additional file 8.**

**Additional file 9.**

**Additional file 10.**

**Additional file 11.**

**Additional file 12.**

**Additional file 13.**

**Additional file 14.**

**Additional file 15.**

**Additional file 16.**

## Data Availability

Final trial dataset will be available for the investigators, without restrictions.

## Abbreviations

CHF: Chronic heart failure
COPD: Chronic obstructive pulmonary disease
CRF: Chronic respiratory failure
FEV_1_: Forced expiratory volume in one second
FiO_2_: Inspired oxygen fraction
FVC: Forced vital capacity
ICU: Intensive care unit
NIV: Non-invasive ventilation
PaCO_2_: Carbon dioxide partial pressure in arterial blood
PaO_2_: Oxygen partial pressure in arterial blood
PEEP: Positive end-expiratory pressure
SAE: Serious adverse events
SBT: Spontaneous breathing trial
SBT-PS: Spontaneous breathing trial with pressure support
SBT-TP: Spontaneous breathing trial with T-piece
SCSS: Semi-quantitative cough strength score
SpO_2_: Peripheral oxygen saturation
VFD: Ventilator-free days
WIPO: Weaning-induced pulmonary oedema
