# Supplementary material for: Pressure support and positive end-expiratory pressure versus T-piece during spontaneous breathing trial in difficult weaning from mechanical ventilation: study protocol for the SBT-ICU study
Source: Trials. 2022 Dec 12;23:993. doi: 10.1186/s13063-022-06896-4 (PMC9742015; doi:10.1186/s13063-022-06896-4)
Supplement: Supplementary file 1 — Additional file 1. [file 13063_2022_6896_MOESM1_ESM.pdf]

Impact de la combinaison de l'aide inspiratoire et de la pression expiratoire positive pendant l'épreuve de sevrage respiratoire en comparaison de la pièce en T sur le délai jusqu'à l'extubation avec succès

SBT-ICU

Protocole impliquant la personne humaine du 2° hors produit de santé

Version 2 du 07/02/2019

Promoteur :

Hospices Civils de Lyon
BP 2251
3 quai des Célestins,
69229 LYON cedex 02

Investigateur principal :

Dr Mehdi MEZIDI
Réanimation Médicale
Hôpital de la Croix-Rousse — GHN
103 Grande Rue de la Croix-Rousse 69317 Lyon CEDEX 04
Tél : 04.26.10.92.75
Email : mehdi.mezidi@chu-lyon.fr

Code promoteur : 69HCL18_0982
N°ID-RDB : 2019-A00106-51
Numéro d'enregistrement clinicaltrials.gov : ***NCTXXXXX***
Avis favorable du CPP Ile de France VI le : 20/02/2019

RÉSUMÉ

**TITRE**	***Impact de la combinaison de l'aide inspiratoire et de la pression expiratoire positive pendant l'épreuve de sevrage respiratoire en comparaison de la pièce en T sur le délai jusqu'à l'extubation avec succès***<br>*SBT-ICU*
**PROMOTEUR**	Hospices Civils de Lyon<br>BP 2251<br>3 quai des Célestins,<br>69229 LYON cedex 02
**INVESTIGATEUR PRINCIPAL**	Dr Mehdi MEZIDI<br>Réanimation Médicale<br>Hôpital de la Croix-Rousse — GHN<br>103 Grande Rue de la Croix-Rousse 69317 Lyon CEDEX 04<br>Tél : 04.26.10.92.75<br>Email : mehdi.mezidi@chu-lyon.fr
**VERSION DU PROTOCOLE**	2 du 07/02/2019
**JUSTIFICATION/CONTEXTE**	Le recours à la ventilation mécanique invasive (via une sonde d'intubation reliée à un ventilateur) est extrêmement fréquent (plus de 40 %) en réanimation (1). Afin de pouvoir séparer définitivement le patient du respirateur, les cliniciens cherchent à déterminer si l'état du patient lui permet de respirer spontanément et sans support, via des tests de ventilation spontanée (TVS).<br><br>Le TVS a pour but d'essayer de reproduire des conditions physiologiques de travail respiratoire proches de celles que le patient aura après extubation. Il existe deux principaux types de TVS : avec Aide Inspiratoire (TVS-AI) ou avec une pièce en T (sans Aide Inspiratoire) (TVS-TT). En pratique, lors du TVS-AI, le patient est maintenu connecté au ventilateur. L'AI est abaissée à un faible niveau. Le TVS-TT consiste à déconnecter le patient du ventilateur et connecter la sonde d'intubation à une pièce en T, n'apportant aucun support respiratoire.<br>Le type de TVS réalisé dans un service de réanimation varie selon les équipes (TVS-TT 42 %, TVS-AI 40 %, autre 18 %(1)). Il a été récemment recommandé (2) de préférer le TVS-AI au TVS-TT, mais avec un niveau de preuve modéré.<br><br>Le TVS-AI pourrait être associé à un taux de succès de l'épreuve et un succès d'extubation discrètement plus élevé que le TVS-TT sur la base de méta-analyses d'études de qualité médiocre(3), mais il n'a pas été démontré de différence sur la mortalité à court terme.<br>L'utilisation d'une pression expiratoire positive (PEP) associée à l'AI pourrait augmenter significativement le taux de succès de l'épreuve notamment en cas de cardiopathie(4), du fait de l'impact bénéfique de la PEP sur un potentiel œdème aigu pulmonaire de sevrage.<br>La ventilation non invasive (VNI) consiste à apporter un support respiratoire via un masque (naso-buccal, le plus souvent). Elle peut être mise en place dès l'extubation du patient. Cette stratégie est recommandée (2,5) chez les patients à haut-risque d'échec d'extubation (patients hypercapniques lors du TVS, avec bronchopneumopathie chronique obstructive [BPCO] ou insuffisance cardiaque chronique, patients âgés de plus de 65 ans(6)).

	TVS-AI et TVS-TT sont souvent opposés. Nous faisons l'hypothèse que ces deux tests sont complémentaires et apportent des informations différentes. Le succès de TVS-TT permet d'envisager une extubation « simple » avec un faible risque de ré-intubation. Cependant, un nombre plus important de patients échouera ce test qui est plus difficile. À l'inverse, le TVS-AI est un test plus « facile » pour les patients, permettant d'envisager une extubation précoce chez plus de patients, parfois au prix d'un support par VNI.<br>Dès lors, il semble pertinent de réaliser séquentiellement un TVS-AI pour décider de la sevrabilité du patient puis, en cas de succès du TVS-AI, un TVS-TT pour détecter les patients à haut-risque de ré-intubation, pouvant alors bénéficier de VNI en post-extubation.<br>Une stratégie « assistée » de sevrage comprenant :<br>- La détermination de la sevrabilité par le TVS-AI<br>- L'utilisation d'une PEP pour prévenir l'OAP de sevrage<br>- Le dépistage des patients à haut risque de ré-intubation via le recours à un TVS-TT ou la présence des critères habituels<br>semble pouvoir raccourcir le délai jusqu'à l'extubation avec succès et diminuer le taux d'échec d'extubation par rapport à une stratégie « non assistée » comprenant :<br>- Détermination de la sevrabilité par le TVS-TT<br>- L'absence de PEP<br>- L'utilisation de la VNI seulement selon les critères habituels.
**Objectifs**	• Objectif principal : Déterminer si une stratégie d'extubation basée sur le TVS-AI, un niveau de PEP modéré et un dépistage des patients à haut-risque de ré-intubation par le TVS-TT permet de raccourcir le délai jusqu'à extubation avec succès chez des patients ayant échoué le premier TVS-TT en comparaison à une stratégie de sevrage basée sur le TVS-TT.<br><br>• Objectifs secondaires :<br>1. Déterminer si une stratégie d'extubation basée sur le TVS-AI, un niveau de PEP modéré et un dépistage des patients à haut-risque de ré-intubation par le TVS-TT permet **d'augmenter le taux de première extubation avec succès** chez des patients ayant échoué le premier TVS-TT en comparaison à une stratégie de sevrage basée sur le TVS-TT.<br>2. Déterminer si une stratégie d'extubation basée sur le TVS-AI, un niveau de PEP modéré et un dépistage des patients à haut-risque de ré-intubation par le TVS-TT permet de raccourcir ***la durée de ventilation mécanique invasive*** chez des patients ayant échoué le premier TVS-TT en comparaison à une stratégie de sevrage basée sur le TVS-TT.<br>3. Déterminer si une stratégie d'extubation basée sur le TVS-AI, un niveau de PEP modéré et un dépistage des patients à haut-risque de ré-intubation par le TVS-TT permet de raccourcir ***la durée totale de ventilation mécanique (invasive et non invasive)*** chez des patients ayant échoué le premier TVS-TT en comparaison à une stratégie de sevrage basée sur le TVS-TT.<br>*4.* Déterminer si une stratégie d'extubation basée sur le TVS-AI, un niveau de PEP modéré et un dépistage des patients à haut-risque par le TVS-TT permet d'augmenter ***le nombre de jours vivant sans ventilation mécanique invasive à J28 et J90*** chez des patients ayant échoué le premier TVS-TT en comparaison à une stratégie de sevrage basée sur le TVS-TT.

	5. Déterminer si une stratégie d'extubation basée sur le TVS-AI, un niveau de PEP modéré et un dépistage des patients à haut-risque de ré-intubation par le TVS-TT permet de raccourcir ***la durée de séjour en réanimation*** chez des patients ayant échoué le premier TVS-TT en comparaison à une stratégie de sevrage basée sur le TVS-TT.<br>6. Déterminer si une stratégie d'extubation basée sur le TVS-AI, un niveau de PEP modéré et un dépistage des patients à haut-risque de ré-intubation par le TVS-TT permet de raccourcir ***la durée d'hospitalisation*** chez des patients ayant échoué le premier TVS-TT en comparaison à une stratégie de sevrage basée sur le TVS-TT.<br>7. Déterminer si une stratégie d'extubation basée sur le TVS-AI, un niveau de PEP modéré et un dépistage des patients à haut-risque de ré-intubation par le TVS-TT permet **de réduire la mortalité à J28, J90** et à la sortie de réanimation chez des patients ayant échoué le premier TVS-TT en comparaison à une stratégie de sevrage basée sur le TVS-TT.<br>8. Déterminer si une stratégie d'extubation basée sur le TVS-AI, un niveau de PEP modéré et un dépistage des patients à haut-risque de ré-intubation par le TVS-TT est associé à un **taux de ré-intubation** similaire à une stratégie de sevrage basée sur le TVS-TT.
**METHODOLOGIE/SCHEMA DE LA RECHERCHE**	Essai contrôlé randomisé ouvert prospectif interventionnel monocentrique de supériorité comparant deux stratégies incluant des tests de ventilation spontanée utilisés dans le cadre du soin courant.<br>Recherche interventionnelle ne comportant que des risques et contraintes minimes du 2° selon l'article L1121-1 du Code de la Santé Publique.
**CRITERES DE JUGEMENT**	• Critère principal : Durée (en heures) entre l'inclusion dans l'étude et l'extubation avec succès (définie par l'absence de décès/ré-intubation OU la sortie vivant de réanimation, dans les 7 j suivants l'extubation), quel que soit le recours à la VNI. En cas d'intubation pour une intervention avec extubation dans les 24 heures suivant la fin du geste, l'échec n'est pas retenu. Chez les patients ne présentant pas les critères d'extubation avec succès, les données seront censurées à J90 ou à la date de décès si elle est plus précoce.<br><br>• Critères secondaires :<br>1. Taux de première extubation avec succès soit : nombre de patients extubés avec succès lors de la première extubation/nombre total de patients extubés<br>2. Durée de ventilation mécanique invasive (heures) : durée totale cumulée de ventilation mécanique invasive depuis l'inclusion<br>3. Durée de ventilation mécanique (heures) : durée totale cumulée de ventilation mécanique (invasive ou non invasive) depuis l'inclusion<br>4. Nombre de jours vivant sans ventilation mécanique invasive à J28 et J90 (jours) (VFD28 et VFD90 respectivement) : soit 28 (ou 90) moins le nombre de jours où le patient est ventilé mécaniquement (le VFD28 et le VFD90 valent zéro quand le patient est décédé dans les 28 ou 90 jours, respectivement)(12). Les jours sont comptés depuis l'inclusion.<br>5. Durée de séjour en réanimation (jours), de l'admission jusqu'à la sortie de réanimation (ou jusqu'à la date du décès si le patient décède en réanimation)<br>6. Durée d'hospitalisation (jours), de l'admission jusqu'à la sortie de l'hôpital vers le domicile ou un service de SSR (ou jusqu'à la date du décès si le patient décède à l'hôpital)

	7. Mortalité à la sortie de réanimation, J28 et J90. Vérification par contact téléphonique.<br>8. Taux de ré-intubation : soit = nombre total de ré-intubations/nombre de total d'extubation (un patient peut être réintubé plusieurs fois), durant le séjour en réanimation
**POPULATION CIBLE**	Cette étude portera sur des patients adultes hospitalisés en réanimation et ventilés mécaniquement depuis plus de 24 h, qui ont échoué leur premier TVS réalisé par une pièce en T.
**CRITERES D'INCLUSION**	1) Sujet majeur de 18 ans ou plus<br>2) Intubé et ventilé mécaniquement en réanimation depuis plus de 24 h<br>3) Présence des critères préalables de sevrabilité (cf. infra)<br>4) Échec du premier TVS-TT
**CRITERES DE NON-INCLUSION**	1) Patient atteint d'une pathologie neuro-musculaire chronique (sclérose latérale amyotrophique, myopathie, myasthénie…)<br>2) Patient ventilé pour syndrome de Guillain-Barré<br>3) Pathologie du système nerveux central (accident vasculaire cérébral récent, arrêt cardiaque avec pronostic neurologique défavorable, encéphalopathie…) responsable de troubles de conscience (définis par une absence de réponse aux ordres simples)<br>4) Patient porteur d'une-trachéotomie<br>5) Maladie chronique sous-jacente fatale en moins d'un an<br>6) Femme enceinte ou allaitante<br>7) Limitation de soins sur la ré-intubation<br>8) Personnes privées de liberté par décision judiciaire ou administrative<br>9) Personnes majeures protégées<br>10) Incompréhension linguistique de la personne qui sera chargée de recevoir l'information<br>11) Absence de couverture sociale<br>12) Absence de consentement du patient s'il est en état de le donner ou de ses proches le cas échéant<br>13) Participation à d'autres études ayant trait au sevrage respiratoire
**CRITERES DE SORTIE D'ETUDE**	1) Transfert dans une autre réanimation ne participant pas à l'étude<br>2) Retrait du consentement<br>3) En cas d'événement indésirable grave sur décision du clinicien
**PROCEDURES**	Les patients susceptibles de participer à l'étude seront identifiés au sein des services de réanimation dans le cadre de leur suivi médical régulier.<br>Les patients sont screenés tous les jours pour la présence des critères préalables de sevrabilité.<br>Un TVS-TT est réalisé pendant 30 minutes dès la présence de tous les critères de sevrabilité. En cas d'échec de ce premier TVS-TT, le patient sera informé par un médecin investigateur de l'existence du protocole (dans le cas où le patient est dans l'incapacité de recevoir cette information, celle-ci sera produite à son/ses proche(s)).<br>Après vérification des critères d'éligibilité et consentement, le patient est inclus dans l'étude (J1). Les patients sont randomisés entre deux stratégies : assistée ou non assistée.<br><br>Dans les 6 heures suivant l'inclusion, et au minimum 30 minutes après le premier TVS-TT, le protocole d'étude est débuté.<br>À J1, puis quotidiennement sont réalisés (si le patient présente toujours les critères préalables de sevrabilité), un TVS-AI (pour le groupe assisté) ou un

	TVS-TT (pour le groupe non assisté) pour une durée de 30 minutes. Quel que soit le résultat du TVS, les réglages antérieurs sont repris à la fin du TVS.<br>Pour le groupe assisté, le succès du TVS-AI suffit à prouver la sevrabilité du patient. Au moins 30 min après la reprise des réglages antérieurs, un TVS-TT d'une durée de 30 minutes est alors réalisé pour déterminer si le patient est à haut-risque de ré-intubation.<br>L'application de la VNI est systématique pour les patients ayant échoué le TVS-TT, alors que pour les patients ayant réussi les deux TVS, la VNI est appliquée selon les recommandations, sauf contre-indications (7).<br>En cas d'échec du TVS-AI, le patient reste intubé.<br>Pour le groupe non assisté, le patient doit réussir le TVS-TT pour prouver sa sevrabilité. Si les critères d'extubabilité sont présents, le patient est extubé au moins 2 h et au maximum 3 h après la fin du TVS. La VNI en post-extubation est appliquée selon les recommandations.<br><br>Une fois prise la décision d'extuber le patient, le patient est ventilé avec ses paramètres antérieurs. L'extubation a lieu au moins 2 h et au maximum 3 h après la fin du dernier TVS.<br>Le patient est extubé selon les procédures du service.<br>La VNI post-extubation est réalisée pour :<br>- Les patients du groupe assisté, qui ont échoué le TVS-TT<br>- Les patients des deux groupes, selon les recommandations (2,5) : âge>65 ans, insuffisance respiratoire ou cardiaque chronique, $PaCO_2$>45 mmHg en fin de TVS, bronchopneumopathie chronique obstructive
**RAPPORT BENEFICES/RISQUES**	*Bénéfices :*<br>Cette étude permettra d'améliorer les connaissances sur le sevrage respiratoire des patients de ventilation. Si l'étude est positive, la stratégie assistée permettra aux futurs patients de bénéficier d'une réduction du temps passé sous ventilation mécanique.<br>*Risques :*<br>Les patients reçoivent deux stratégies utilisées quotidiennement dans les services de réanimation. Il n'est pas attendu de risques supplémentaires liés à cette étude.<br>Au total, le rapport bénéfices/risques ne paraît pas défavorable.
**NOMBRE DE SUJETS**	47 patients analysables par groupe, soit 94 patients au total.<br>Au maximum, 104 patients seront inclus.
**DUREE DE L'ETUDE**	Durée de la période d'inclusion : 30 mois<br>Durée de la participation pour chaque sujet : 90 jours<br>Durée totale de l'étude : 30 mois + 90 jours soit 33 mois
**LIEU DE LA RECHERCHE**	Réanimation médicale, Hôpital de la Croix Rousse, Groupement Hospitalier Nord
**RETOMBEES ATTENDUES**	Cette étude, si elle est positive, confirmera qu'une stratégie « assistée » de sevrage respiratoire guidée par le TVS-AI permet d'extuber avec succès plus rapidement les patients. La ventilation mécanique invasive est associée au maintien en réanimation, à de l'inconfort et à de nombreux risques iatrogènes (auto-extubation, agression pulmonaire induite par la ventilation mécanique, obstruction de sonde…). La réduction de sa durée est donc primordiale pour les patients de réanimation.<br>Si l'étude est négative, cela signifiera qu'il est probable que les stratégies assistée et non assistée ont un impact équivalent sur le délai d'extubation avec succès.

**References**	1. Beduneau G, Pham T, Schortgen F, Piquilloud L, Zogheib E, Jonas M, et al. Epidemiology of Weaning Outcome according to a New Definition. The WIND Study. Am J Respir Crit Care Med. 3rd ed. 2017 Mar 15; 195 (6):772–83.<br><br>2. Schmidt GA, Girard TD, Kress JP, Morris PE, Ouellette DR, Alhazzani W, et al. Official Executive Summary of an American Thoracic Society/American College of Chest Physicians Clinical Practice Guideline: Liberation from Mechanical Ventilation in Critically Ill Adults. Am J Respir Crit Care Med. 2017 Jan; 195 (1):115–9.<br><br>3. Burns KEA, Soliman I, Adhikari NKJ, Zwein A, Wong JTY, Gomez-Builes C, et al. Trials directly comparing alternative spontaneous breathing trial techniques: a systematic review and meta-analysis. Crit Care. Critical Care; 2017 May 26; 21 (1):1–11.<br><br>4. Cabello B, Thille AW, Roche-Campo F, Brochard L, Gómez FJ, Mancebo J. Physiological comparison of three spontaneous breathing trials in difficult-to-wean patients. Intensive Care Med. 2010 Mar 30; 36 (7):1171–9.<br><br>5. SFAR, SRLF. Recommendations Formalisées d'Experts Intubation et extubation du patient de réanimation. 2016. Available from: https://www.srlf.org/wp-content/uploads/2016/09/20160927_RFE_Intubation-Extubation.pdf<br><br>6. Thille AW, Boissier F, Ben-Ghezala H, Razazi K, Mekontso-Dessap A, Brun-Buisson C, et al. Easily identified at-risk patients for extubation failure may benefit from noninvasive ventilation: a prospective before-after study. Crit Care. Critical Care; 2016 Feb 22; 20 (1):1–8.<br><br>7. SFAR, SPLF, SRLF. Conférence de Consensus commune : Ventilation non invasive au cours de l'insuffisance respiratoire aiguë (nouveau-né exclu). 2006 Nov ; : 1–8.

LISTE DES ABRÉVIATIONS

**AI**	Aide inspiratoire
**ANSM**	Agence Nationale de Sécurité des Médicaments et des produits de santé
**ARC**	Attaché de Recherche Clinique
**BPC**	Bonnes Pratiques Cliniques
**BPCO**	Broncho-pneumopathie chronique obstructive
**CNIL**	Commission Nationale Informatique et Liberté
**CPP**	Comité de Protection des Personnes
**CRF**	Case Report Form (cahier d'observation)
**EI**	Evènement Indésirable
**EIG**	Evènement Indésirable Grave
**GHN**	Groupement Hospitalier Nord
**HCL**	Hospices Civils de Lyon
**ICH**	International Conference on Harmonisation
**MR**	Méthodologie de Référence
**PEP**	Pression expiratoire positive
**TEC**	Technicien d'Étude Clinique
**TVS**	Test de ventilation spontanée
**VNI**	Ventilation non-invasive
**VRB**	Volontaires pour les Recherches Biomédicales

SOMMAIRE

1 INFORMATIONS GÉNÉRALES 11
1.1. Titre 11
1.2. Identifiants du projet et historique des mises à jour 11
1.3. Promoteur 11
1.4. Investigateurs 12
1.4.1. Investigateur principal 12
1.4.2. Investigateurs associés 12
1.5. Scientifique associé 13
2 JUSTIFICATION SCIENTIFIQUE 13
2.1 État actuel des connaissances — Rationnel 13
2.2 Hypothèse de la recherche 15
2.3 Justification des choix méthodologiques 15
2.4 Population cible 15
2.5 Rapport bénéfices/risques 15
2.6 Retombées attendues 15
3 OBJECTIFS DE LA RECHERCHE 16
3.1 Objectif principal 16
3.2 Objectifs secondaires 16
4 CONCEPTION DE LA RECHERCHE 17
4.1 Type d'étude 17
4.2 Méthode pour la randomisation 17
4.3 Critères de jugement 17
4.3.1 Critère de jugement principal 17
4.3.2 Critères de jugement secondaires 17
5 CRITÈRES D'ÉLIGIBILITÉ 18
5.1 Critères d'inclusion 18
5.2 Critères de non-inclusion 18
5.3 Critères d'exclusion secondaire 18
5.4 Modalités de recrutement et faisabilité 18
6 STRATÉGIES EXPÉRIMENTALES 19
6.1 Stratégie expérimentale : stratégie assistée de sevrage 19
6.2 Stratégie de comparaison : stratégie non assistée de sevrage 19
6.3 Insu 19
6.4 Traitements associés autorisés et interdits 19
7 ORGANISATION GÉNÉRALE 20
7.1 Calendrier de l'étude 20
7.2 Schéma général et tableau récapitulatif 20
7.3 Déroulement de l'étude 22
7.3.1 Screening 22
7.3.2 Visite d'inclusion/Randomisation 23
7.3.3 Visites de suivi 23
7.3.4 Visite de fin de la recherche 27
7.4 Règles d'arrêt temporaire ou définitif 27
8 ÉVALUATION DE LA SÉCURITÉ 29
9 ASPECTS STATISTIQUES 29
9.1 Nombre de sujets nécessaires 29
9.2 Description des méthodes statistiques 30
9.3 Méthode de prise en compte des données manquantes 31
9.4 Gestion des modifications apportées au plan d'analyse 31
9.5 Responsable des analyses et logiciel utilisé 31
10 DROITS D'ACCÈS AUX DONNÉES ET DOCUMENTS SOURCES 31

10.1 Accès aux données 31
10.2 Documents sources..31
10.3 Confidentialité des données 31
11 CONTRÔLE ET ASSURANCE DE LA QUALITÉ..32
12 CONSIDÉRATIONS ÉTHIQUES..32
12.1 Autorités compétentes..32
12.2 Modifications substantielles..32
12.3 Information du patient et formulaire de consentement écrit 33
12.4 Déclaration de conformité..33
12.5 Période d'exclusion..33
12.6 Indemnisation des sujets et inscription au fichier national des personnes se prêtant à une recherche biomédicale 33
13 GESTION ET CONSERVATION DES DONNÉES 34
13.1 Cahier d'observation 34
13.2 Gestion des données..34
13.3 CNIL..34
13.4 Archivage 34
14 FINANCEMENT ET ASSURANCE 35
14.1 Budget de l'étude 35
14.2 Assurance 35
15 RÈGLES RELATIVES À LA PUBLICATION 35
16 RÉFÉRENCES BIBLIOGRAPHIQUES 35
17 LISTE DES ANNEXES..37

1 INFORMATIONS GÉNÉRALES

1.1. Titre

Impact de la combinaison de l'aide inspiratoire, de la pression expiratoire positive pendant l'épreuve de sevrage respiratoire en comparaison de la pièce en T sur le délai jusqu'à l'extubation avec succès
SBT-ICU

1.2. Identifiants du projet et historique des mises à jour

Code promoteur : 69HCL18_0982
Numéro d'enregistrement clinicaltrials.gov : *NCTXXXXX*
Avis favorable du CPP Ile de France VI le : 20/02/2019

Historique des versions		
**Version**	**Date**	**Motif de la mise à jour**
1	11/01/2019	Version soumise au CPP
2	07/02/2019	Réponses aux Remarques du CPP

1.3. Promoteur

- *Identité :*

Hospices Civils de Lyon
BP 2251
3 Quai des Célestins
69229 LYON Cedex 02

- *Signature du protocole au nom du Promoteur :*

Muriel MALBEZIN, Directeur de la Recherche Clinique et de l'Innovation
Hospices Civils de Lyon, Direction de la Recherche Clinique et de l'Innovation, Siège Administratif, BP 2251, 3 Quai des Célestins, 69229 LYON Cedex 02
Tél : 04 72 40 68 52, Fax : 04 72 40 68 69

- *Responsable de la recherche au niveau du Promoteur :*

Valérie PLATTNER, médecin référent
Hospices Civils de Lyon, Direction de la Recherche Clinique et de l'Innovation, Siège Administratif, BP 2251, 3 Quai des Célestins, 69229 LYON Cedex 02
Tél : 04 72 40 68 40, Fax : 04 72 11 51 90

- *Responsable de la vigilance des essais au niveau du Promoteur :*

Marina NGUON, pharmacien référent
Hospices Civils de Lyon, Direction de la Recherche Clinique et de l'Innovation, Siège Administratif, BP 2251, 3 Quai des Célestins, 69229 LYON Cedex 02
Tél : 04 72 40 68 26, Fax : 04 72 11 51 90

1.4. Investigateurs

1.4.1. Investigateur principal

Dr Mehdi MEZIDI
Réanimation Médicale
Hôpital de la Croix-Rousse — GHN
103 Grande Rue de la Croix-Rousse 69317 Lyon CEDEX 04
Tél : 04.26.10.92.75
Email : mehdi.mezidi@chu-lyon.fr

1.4.2. Investigateurs associés

- *Pr Jean-Christophe RICHARD*
  Réanimation Médicale
  Hôpital de la Croix-Rousse — GHN
  103 Grande Rue de la Croix-Rousse 69317 Lyon CEDEX 04
  Tél : 04.26.10.92.72
  Email : j-christophe.richard@chu-lyon.fr

- *Dr Laurent BITKER*
  Réanimation Médicale
  Hôpital de la Croix-Rousse — GHN
  103 Grande Rue de la Croix-Rousse 69317 Lyon CEDEX 04
  Tél : 04.26.10.94.33
  Email : laurent.bitker@chu-lyon.fr

- *Dr Hodane YONIS*
  Réanimation Médicale
  Hôpital de la Croix-Rousse — GHN
  103 Grande Rue de la Croix-Rousse 69317 Lyon CEDEX 04
  Tél : 04.26.10.92.71
  Email : hodane.yonis@chu-lyon.fr

- *Dr Laure FOLLIET*
  Réanimation Médicale
  Hôpital de la Croix-Rousse — GHN
  103 Grande Rue de la Croix-Rousse 69317 Lyon CEDEX 04
  Tél : 04.26.10.92.90
  Email : laure.folliet@chu-lyon.fr

- *Dr Paul CHABERT*
  Réanimation Médicale
  Hôpital de la Croix-Rousse — GHN
  103 Grande Rue de la Croix-Rousse 69317 Lyon CEDEX 04
  Tél : 04.26.10.92.90
  Email : paul.chabert@chu-lyon.fr

- *Dr Louis CHAUVELOT*
  Réanimation Médicale
  Hôpital de la Croix-Rousse — GHN
  103 Grande Rue de la Croix-Rousse 69317 Lyon CEDEX 04
  Tél : 04.26.10.92.90
  Email : louis.chauvelot@chu-lyon.fr

1.5. Scientifique associé

Loredana BABOI
Réanimation Médicale
Hôpital de la Croix-Rousse — GHN
103 Grande Rue de la Croix-Rousse 69317 Lyon CEDEX 04
Tél : 04.26.10.92.90
Email : loredana.baboi@chu-lyon.fr

2 JUSTIFICATION SCIENTIFIQUE

2.1 État actuel des connaissances — Rationnel

a) Généralités

Le recours à la ventilation mécanique invasive (via une sonde d'intubation reliée à un ventilateur) est extrêmement fréquent (plus de 40 %) en réanimation (1). Les patients requièrent une ventilation mécanique du fait d'une pathologie respiratoire grave, d'une altération profonde de l'état de conscience, dans les suites d'une intervention chirurgicale ou dans un contexte d'état de choc. Les patients sont le plus souvent sédatés à la phase initiale de la prise en charge, puis après traitement de la cause de la défaillance respiratoire, la sédation est arrêtée, afin d'envisager une extubation.

Afin de pouvoir séparer définitivement le patient du respirateur, les cliniciens cherchent à déterminer si l'état du patient lui permet de respirer spontanément et sans support, via des tests de ventilation spontanée (TVS). Ces TVS sont débutés dès que le patient est stabilisé notamment sur les plans cardiaque, respiratoire et neurologique. En pratique, 24 % (intervalle de confiance (IC) à 95 % : 23–26) des patients intubés en réanimation n'auront pas de TVS, car décédés ou auto-extubés (1). 57 % IC95% [55–59] des patients réaliseront un TVS et seront extubés après (sevrage simple), 10 % IC95% [9–11] seront extubés dans les sept jours qui suivent le premier TVS (sevrage difficile) et 9 % IC95% [8–10] ne seront toujours pas extubés dans les sept jours qui suivent le premier TVS (sevrage prolongé). La mortalité est croissante entre les groupes sevrage simple, difficile et prolongé.

b) TVS : rôle de l'aide inspiratoire (AI)

Le TVS a pour but d'essayer de reproduire des conditions physiologiques de travail respiratoire proches de celles que le patient aura après extubation. Il existe deux principaux types de TVS : avec Aide Inspiratoire (TVS-AI) ou avec une pièce en T (sans Aide Inspiratoire) (TVS-TT). En pratique, lors du TVS-AI, le patient est maintenu connecté au ventilateur. L'AI est abaissée à un faible niveau (entre 4 et 10 cmH2O selon les études, Fig. 1). Cette hétérogénéité des pratiques rend difficile leur comparaison dans les méta-analyses. Le TVS-TT consiste à déconnecter le patient du ventilateur et connecter la sonde d'intubation à une pièce en T, n'apportant aucune forme d'AI ou de PEP, mais permettant de délivrer de l'oxygène si besoin. Quel que soit le type de TVS, sa durée varie entre 30 minutes et plusieurs heures, selon les équipes et il est recherché activement pendant son déroulement des critères cliniques et paracliniques d'échec. Les échecs de TVS peuvent s'expliquer par la survenue d'un œdème aigu pulmonaire (OAP) de sevrage (jusqu'à 60 % des échecs (8)) ou l'incapacité des muscles respiratoires à assurer une ventilation suffisante. En cas de réussite du TVS, il est alors conclu que l'état du patient permet une extubation.

Le type de TVS réalisé dans un service de réanimation varie selon les équipes (TVS-TT 42 %, TVS-AI 40 %, autre 18 % (1)). Il a été récemment recommandé (2) de préférer le TVS-AI au TVS-TT, mais avec un niveau de preuve modéré.

Le TVS-AI pourrait être associé à un taux de succès de l'épreuve et un succès d'extubation discrètement plus élevé que le TVS-TT sur la base de méta-analyses d'études de qualité médiocre (3), mais il n'a pas été démontré de différence sur la mortalité à court-terme.

Par ailleurs, il n'existe pas d'essai ayant comparé l'impact sur la durée de ventilation mécanique du TVS-AI par rapport au TVS-TT.

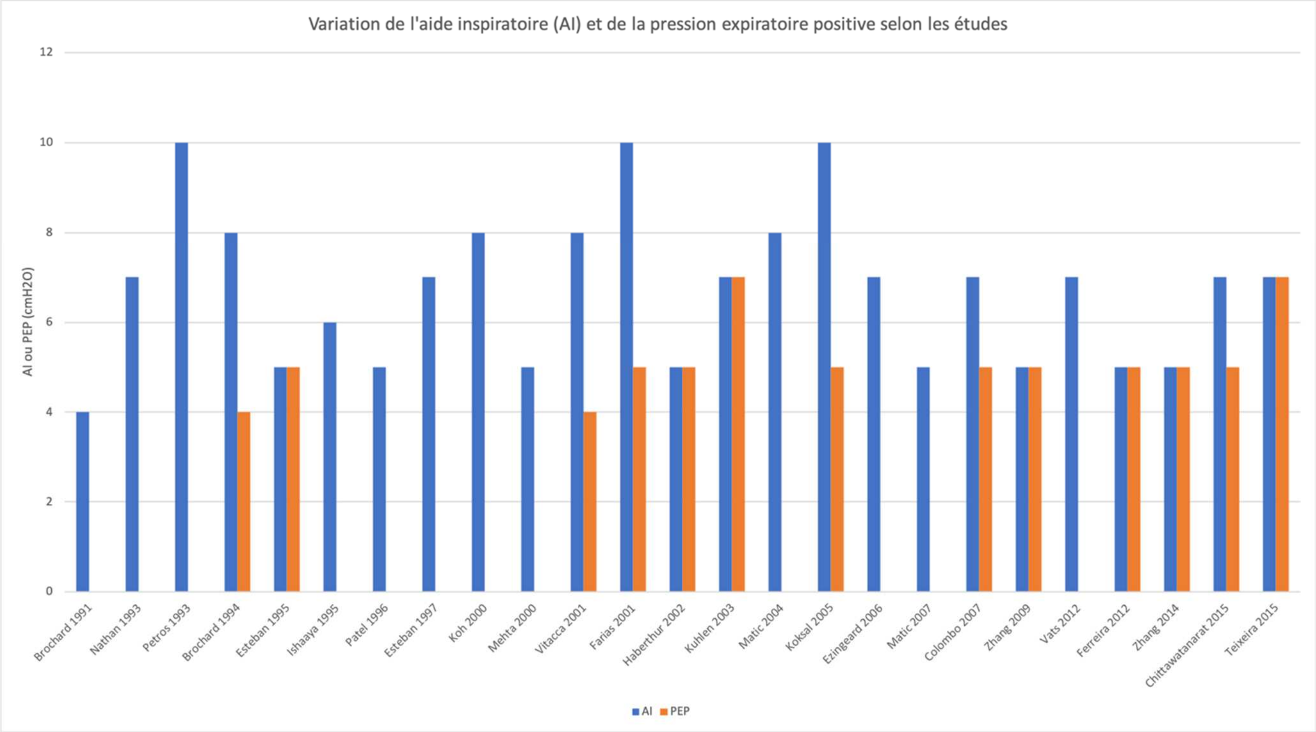

Fig. 1 Aide inspiratoire (AI) et pression expiratoire positive (PEP) utilisées dans les études

c) Rôle de la pression expiratoire positive (PEP)

Lors d'un TVS-AI, il est possible d'adjoindre une PEP en plus de l'AI. Le niveau de PEP est très variable selon les études (Fig. 1). L'utilisation d'une pression expiratoire positive (PEP) associée à l'AI pourrait augmenter significativement le taux de succès de l'épreuve notamment en cas d'hypervolémie ou de cardiopathie (4), du fait de l'effet protecteur de la PEP sur la survenue d'un potentiel OAP de sevrage.

d) Rôle de la ventilation non invasive (VNI) en post-extubation

La VNI consiste à apporter un support respiratoire via un masque. Elle peut être mise en place dès l'extubation du patient. Cette stratégie est recommandée (2,5) chez les patients à haut-risque d'échec d'extubation (patients hypercapniques lors du TVS, avec bronchopneumopathie chronique obstructive (BPCO) ou insuffisance cardiaque chronique, et patients âgés de plus de 65 ans (6)).

Le TVS-TT prédit le mieux le travail respiratoire en post-extubation (9), d'un point de vue physiologique. Cependant si une VNI est mise en place en post-extubation, le travail respiratoire est moindre. Dès lors, il ne semble pas nécessaire que le TVS reproduise exactement les conditions post-extubation sans aucun support respiratoire. Enfin, aucune étude ne s'est intéressée à l'impact d'une stratégie de sevrage sur la durée de ventilation mécanique. Certains auteurs ont proposé pour les patients difficiles à sevrer et qui échouent le TVS, d'extuber tout de même les patients avec un relais immédiat par VNI. Cette stratégie est associée à une amélioration du pronostic des patients BPCO (10), mais sans impact sur la durée de sevrage de toute ventilation dans une population générale de réanimation (11) et n'est pas recommandée.

e) Association du TVS-AI et du TVS-TT : dépistage des patients à haut risque d'échec

TVS-AI et TVS-TT sont souvent opposés. Nous faisons l'hypothèse que ces deux tests sont complémentaires et apportent des informations différentes. Le succès de TVS-TT permet d'envisager une extubation « simple » avec un faible risque de ré-intubation. Cependant, un nombre plus important de patients échouera ce test qui est plus difficile. À l'inverse, le TVS-AI est un test plus « facile » pour les patients, permettant d'envisager une extubation précoce chez plus de patients, assistée par un support par VNI chez les patients à haut risque d'échec.

Dès lors, il semble pertinent de réaliser séquentiellement un TVS-AI pour décider de la sevrabilité du patient puis, en cas de succès du TVS-AI, un TVS-TT pour détecter les patients à haut-risque de ré-intubation, pouvant alors bénéficier de VNI en post-extubation.

Une stratégie « assistée» de sevrage comprenant :
- La détermination de la sevrabilité par le TVS-AI
- L'utilisation d'une PEP pour prévenir l'OAP de sevrage
- Le dépistage des patients à haut risque de ré-intubation via le recours à un TVS-TT ou la présence des critères habituels

semble pouvoir raccourcir le délai jusqu'à l'extubation avec succès et diminuer le taux d'échec d'extubation par rapport à une stratégie « non assistée » comprenant :
- Détermination de la sevrabilité par le TVS-TT
- L'absence de PEP
- L'utilisation de la VNI seulement selon les critères habituels.

2.2 *Hypothèse de la recherche*

Une stratégie « assistée » de sevrage respiratoire fondée sur le TVS-AI, l'utilisation d'une PEP associée à un dépistage des patients à haut-risque par le TVS-TT permet une diminution du délai jusqu'à extubation avec succès, chez des patients ayant échoué leur premier TVS-TT.

2.3 *Justification des choix méthodologiques*

Le recours à un essai contrôlé randomisé permet de comparer au mieux les deux stratégies en s'affranchissant des biais associés aux études observationnelles.
Le critère de jugement principal est cliniquement pertinent :
- Il prend en compte les risques compétitifs présents (décès, ré-intubation, échec de sevrage)
- Une réduction de ce délai permet de réduire la durée d'intubation (associée à de l'inconfort, un risque d'infection nosocomiale, une augmentation de la durée de séjour en réanimation et des coûts associés…)

2.4 *Population cible*

Cette étude portera sur des patients adultes hospitalisés en réanimation et ventilés mécaniquement depuis plus de 24 h, qui ont échoué leur premier TVS réalisé par une pièce en T.

2.5 *Rapport bénéfices/risques*

Bénéfices :
Cette étude permettra d'améliorer les connaissances sur le sevrage respiratoire des patients de ventilation. Si l'étude est positive, la stratégie TVS-AI permettra aux futurs patients de bénéficier d'une réduction du temps passé sous ventilation mécanique invasive.
Risques :
Les patients reçoivent deux stratégies utilisées quotidiennement dans les services de réanimation. Il n'est pas attendu de risques supplémentaires liés à cette étude.

Au total, le rapport bénéfices/risques ne paraît pas défavorable.

2.6 *Retombées attendues*

Cette étude, si elle est positive, apportera les premières données suggérant qu'une stratégie « assistée » de sevrage respiratoire guidée par le TVS-AI permet d'extuber avec succès plus rapidement les patients. La

ventilation mécanique invasive est associée au maintien en réanimation, à de l'inconfort et à de nombreux risques iatrogènes (auto-extubation, agression pulmonaire induite par la ventilation mécanique, obstruction de sonde, pneumopathie nosocomiale…). La réduction de sa durée est donc primordiale pour les patients de réanimation.
Si l'étude est négative, elle permettra d'avoir une estimation de l'ampleur de l'effet bénéfique de la stratégie intervention.
Dans l'hypothèse où l'étude est positive ou si l'amplitude de l'effet en faveur de la stratégie intervention suggère un bénéfice pour le patient, cette étude servira d'étude pilote pour un essai randomisé de plus grande ampleur.

3 OBJECTIFS DE LA RECHERCHE

3.1 Objectif principal

Déterminer si une stratégie d'extubation basée sur le TVS-AI, un niveau de PEP modéré et un dépistage des patients à haut-risque par le TVS-TT permet de raccourcir ***le délai jusqu'à extubation avec succès*** chez des patients ayant échoué le premier TVS-TT en comparaison à une stratégie de sevrage basée sur le TVS-TT.

3.2 Objectifs secondaires

1. Déterminer si une stratégie d'extubation basée sur le TVS-AI, un niveau de PEP modéré et un dépistage des patients à haut-risque de ré-intubation par le TVS-TT permet **d'augmenter le taux de première extubation avec succès** chez des patients ayant échoué le premier TVS-TT en comparaison à une stratégie de sevrage basée sur le TVS-TT.
2. Déterminer si une stratégie d'extubation basée sur le TVS-AI, un niveau de PEP modéré et un dépistage des patients à haut-risque de ré-intubation par le TVS-TT permet de raccourcir ***la durée de ventilation mécanique invasive*** chez des patients ayant échoué le premier TVS-TT en comparaison à une stratégie de sevrage basée sur le TVS-TT.
3. Déterminer si une stratégie d'extubation basée sur le TVS-AI, un niveau de PEP modéré et un dépistage des patients à haut-risque de ré-intubation par le TVS-TT permet de raccourcir ***la durée ventilation mécanique (invasive et non invasive)*** chez des patients ayant échoué le premier TVS-TT en comparaison à une stratégie de sevrage basée sur le TVS-TT.
4. Déterminer si une stratégie d'extubation basée sur le TVS-AI, un niveau de PEP modéré et un dépistage des patients à haut-risque de ré-intubation par le TVS-TT permet d'augmenter ***le nombre de jours vivant sans ventilation mécanique invasive à J28 et J90*** chez des patients ayant échoué le premier TVS-TT en comparaison à une stratégie de sevrage basée sur le TVS-TT.
5. Déterminer si une stratégie d'extubation basée sur le TVS-AI, un niveau de PEP modéré et un dépistage des patients à haut-risque de ré-intubation par le TVS-TT permet de raccourcir ***la durée de séjour en réanimation*** chez des patients ayant échoué le premier TVS-TT en comparaison à une stratégie de sevrage basée sur le TVS-TT.
6. Déterminer si une stratégie d'extubation basée sur le TVS-AI, un niveau de PEP modéré et un dépistage des patients à haut-risque de ré-intubation par le TVS-TT permet de raccourcir ***la durée d'hospitalisation*** chez des patients ayant échoué le premier TVS-TT en comparaison à une stratégie de sevrage basée sur le TVS-TT.
7. Déterminer si une stratégie d'extubation basée sur le TVS-AI, un niveau de PEP modéré et un dépistage des patients à haut-risque de ré-intubation par le TVS-TT permet **de réduire la mortalité à J28, J90** et à la sortie de réanimation chez des patients ayant échoué le premier TVS-TT en comparaison à une stratégie de sevrage basée sur le TVS-TT.
8. Déterminer si une stratégie d'extubation basée sur le TVS-AI, un niveau de PEP modéré et un dépistage des patients à haut-risque de ré-intubation par le TVS-TT est associé à un **taux de ré-intubation** similaire à une stratégie de sevrage basée sur le TVS-TT.

4 CONCEPTION DE LA RECHERCHE

4.1 Type d'étude

Essai contrôlé randomisé ouvert prospectif interventionnel monocentrique de supériorité comparant deux stratégies incluant des tests de ventilation spontanée utilisés dans le cadre du soin courant.
Recherche interventionnelle ne comportant que des risques et contraintes minimes du 2° selon l'article L1121-1 du Code de la Santé Publique.

4.2 Méthode pour la randomisation

Liste de randomisation réalisée par ordinateur avec stratification sur l'existence d'une BPCO suspectée ou confirmée et l'existence d'une dysfonction ventriculaire gauche définie par une fraction d'éjection inférieure à 45 %. Les patients avec à la fois une BPCO prouvée ou suspectée et une dysfonction ventriculaire gauche seront classés dans le groupe BPCO (6).
La BPCO est suspectée (GOLD 2018) devant un tableau clinique de dyspnée, toux chronique, crachats et/ou une exposition au facteurs de risques de la maladie (tabagisme, exposition aux fumées/gaz/vapeurs de manière personnelle ou professionnelle) chez un patient de 40 ans ou plus. Elle est confirmée par les épreuves fonctionnelles respiratoires retrouvant un rapport de Tiffeneau post-bronchodilatateur<70%.

4.3 Critères de jugement

4.3.1 Critère de jugement principal

Durée (en heures) entre l'inclusion dans l'étude et l'extubation avec succès (définie par l'absence de décès/ré-intubation OU la sortie vivant de réanimation, dans les 7 j suivants l'extubation), quel que soit le recours à la VNI. En cas d'intubation pour une intervention avec extubation dans les 24 heures suivant la fin du geste, l'échec n'est pas retenu. Chez les patients ne présentant pas les critères d'extubation avec succès, les données seront censurées à J90 ou à la date de décès si elle est plus précoce. Ce critère de jugement sera analysé selon les méthodes de survie avec évaluation de l'impact de la stratégie via un modèle de Cox ajusté sur les critères de stratification (BPCO, insuffisance ventriculaire gauche).

4.3.2 Critères de jugement secondaires

1. Taux de première extubation avec succès soit : nombre de patients extubés avec succès lors de la première extubation/nombre total de patients extubés

2. Durée de ventilation mécanique invasive (heures) : durée totale cumulée de ventilation mécanique invasive depuis l'inclusion

3. Durée de ventilation mécanique (heures) : durée totale cumulée de ventilation mécanique (invasive ou non invasive) depuis l'inclusion

4. Nombre de jours vivant sans ventilation mécanique invasive à J28 et J90 (jours) (VFD28 et VFD90 respectivement) : soit 28 (ou 90) moins le nombre de jours où le patient est ventilé mécaniquement (le VFD28 et le VFD90 valent zéro quand le patient est décédé dans les 28 ou 90 jours, respectivement) (12). Les jours sont comptés à partir de l'inclusion

5. Durée de séjour en réanimation (jours), de l'admission jusqu'à la sortie de réanimation (ou jusqu'à la date du décès si le patient décède en réanimation)

6. Durée d'hospitalisation (jours), de l'admission jusqu'à la sortie de l'hôpital vers le domicile ou un service de SSR (ou jusqu'à la date du décès si le patient décède à l'hôpital).

7. Mortalité à la sortie de réanimation, J28 et J90. Vérification par contact téléphonique.

8. Taux de ré-intubation : soit = nombre total de ré-intubations/nombre total d'extubation (un patient peut être réintubé plusieurs fois) durant le séjour en réanimation

5 CRITÈRES D'ÉLIGIBILITÉ

5.1 Critères d'inclusion

1) Sujet majeur de 18 ans ou plus
2) Intubé et ventilé mécaniquement en réanimation depuis plus de 24 h
3) Présence des critères préalables de sevrabilité (cf. infra)
4) Échec du premier TVS-TT

5.2 Critères de non-inclusion

1) Patient atteint d'une pathologie neuro-musculaire chronique (sclérose latérale amyotrophique, myopathie, myasthénie…)
2) Patient ventilé pour syndrome de Guillain-Barré
3) Pathologie du système nerveux central (accident vasculaire cérébral récent, arrêt cardiaque avec pronostic neurologique défavorable, encéphalopathie…) responsable de troubles de conscience (définis par une absence de réponse aux ordres simples)
4) Patient porteur d'une-trachéotomie
5) Maladie chronique sous-jacente fatale en moins d'un an
6) Femme enceinte ou allaitante
7) Limitation de soins sur la ré-intubation
8) Personnes privées de liberté par décision judiciaire ou administrative
9) Personnes majeures protégées
10) Incompréhension linguistique de la personne qui sera chargée de recevoir l'information
11) Absence de couverture sociale
12) Absence de consentement du patient s'il est en état de le donner ou de ses proches le cas échéant
13) Participation à d'autres études ayant trait au sevrage respiratoire

5.3 Critères d'exclusion secondaire

1) Transfert dans une autre réanimation ne participant pas à l'étude
2) Retrait du consentement
3) En cas d'événement indésirable grave sur décision du clinicien

5.4 Modalités de recrutement et faisabilité

Dans une étude préliminaire rétrospective réalisée sur 88 patients consécutifs du service ventilés mécaniquement, 81 patients étaient ventilés plus de 24 h. 54 patients entraient dans un processus de sevrage respiratoire et 17 présentaient un sevrage respiratoire compliqué. Cette étude était réalisée sur une période de 110 jours, avec la stratégie assistée (bras intervention, utilisation du TVS-AI pour qualifier le sevrage comme compliqué). Il est attendu que le nombre de patients éligibles pour l'étude soit plus important puisque les critères de screening ont été identifiés avec le TVS-AI.
Ainsi, sur une année, il semble envisageable théoriquement d'inclure 56 patients par an à l'échelle d'un service. En comptant 15 % de patients non inclus pour refus de participation ou screening non réalisé, il semble réaliste d'inclure 48 patients par an. Le nombre de sujets nécessaire étant 94 analysables (cf. infra) et 104 à inclure au maximum pour tenir compte des patients à remplacer, l'étude est théoriquement réalisable en 23 mois à l'échelle d'un service des HCL.

À partir de ces données, il est prévu d'inclure l'ensemble des patients en 30 mois, au maximum.

6 STRATÉGIES EXPÉRIMENTALES

6.1 Stratégie expérimentale : stratégie assistée de sevrage

- **Définition et description de la stratégie :** Pour le groupe assisté, le succès du TVS-AI permet d'extuber le patient. Un faible niveau de PEP est appliqué lors du TVS-AI. L'application de la VNI est systématique pour les patients ayant échoué le TVS-TT, alors que pour les patients ayant réussi les deux TVS, la VNI est appliquée selon les recommandations (2,5).
- **Justification de la stratégie :** il est fait l'hypothèse que cette stratégie permet de réduire la durée jusqu'à extubation avec succès.
- **Description des contre-indications :** aucune

6.2 Stratégie de comparaison : stratégie non assistée de sevrage

- **Définition et description de la stratégie :** Pour le groupe non assisté, le patient doit réussir un TVS-TT pour être extubé. La VNI en post-extubation est appliquée selon les recommandations.
- **Justification de la stratégie :** cette stratégie est encore celle la plus couramment appliquée dans les réanimations (1)
- **Description des contre-indications :** aucune

6.3 Insu

- **Organisation**

L'insu n'est pas possible, car les patients seront extubés selon la stratégie dans laquelle ils ont été randomisés. Cependant, le critère de jugement principal n'est pas soumis à interprétation puisque l'extubation, l'assistance respiratoire post-extubation et la ré-intubation sont encadrées par des critères prédéterminés, contrôlant ainsi un potentiel biais d'évaluation.

6.4 Traitements associés autorisés et interdits

Aucun traitement n'est interdit durant l'étude.

7 ORGANISATION GÉNÉRALE

7.1 Calendrier de l'étude

Durée de la période d'inclusion : *30 mois*
Durée de la participation pour chaque patient : *90 jours*
Durée totale de l'étude : *= 30 mois + 90 jours de suivi soit 33 mois*
Début des inclusions : 1er – 2nd trimestre 2019

Dès la première inclusion, le promoteur doit informer sans délai le CPP de la date effective de démarrage de l'étude (date effective de démarrage = date de signature du consentement par la première personne qui se prête à la recherche).
La date de fin d'étude sera transmise par le promoteur au CPP dans un délai de 90 jours. La date de fin de la recherche correspond au terme de la participation de la dernière personne qui se prête à la recherche, ou le cas échéant, au terme défini dans le protocole.

7.2 Schéma général et tableau récapitulatif

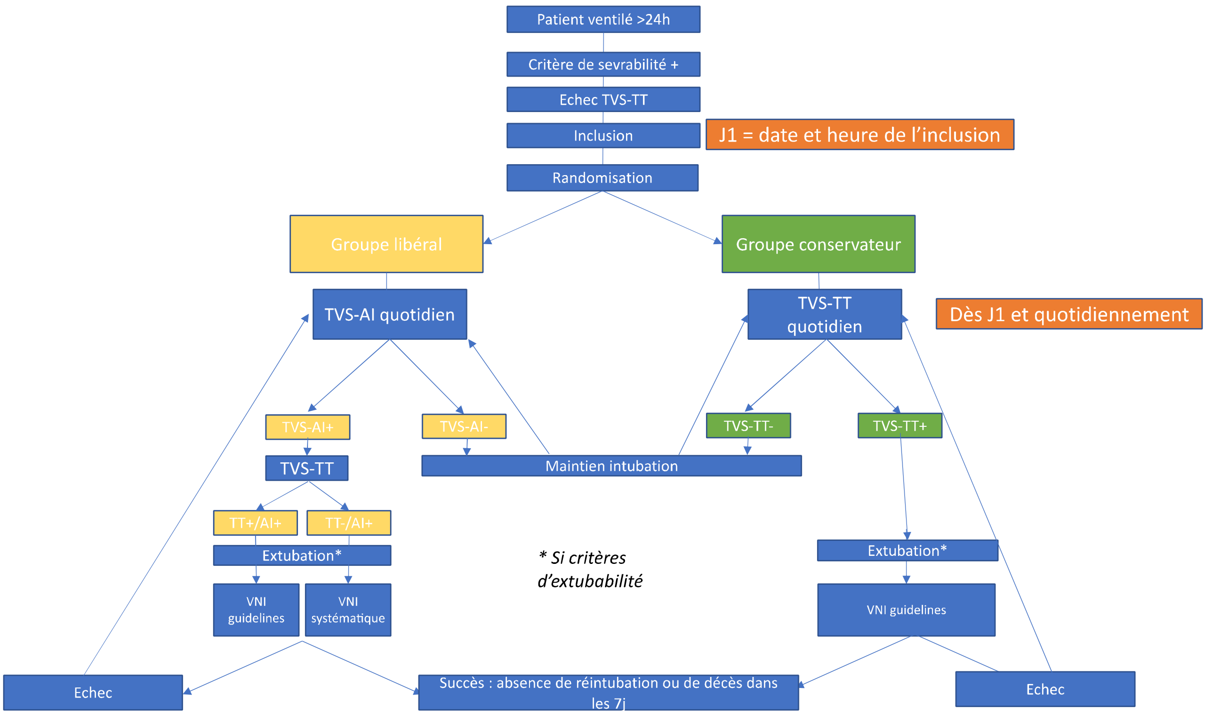

Fig. 2 Schéma général de l'étude

Les patients étant hospitalisés en permanence en réanimation, il n'est pas prévu de visite de suivi au-delà du séjour en réanimation. La mortalité sera évaluée à J28 et à J90 par contact téléphonique.

	Inclusion (J1)	J1-J90	Sortie/Décès	J28 +/- 7jours	J90 +/- 7 jours
**Données démographiques**	X				
Si patient intubé					
**Présence des critères de sevrabilité**		X*			
**Score de toux**		X*			
**Score d'encombrement respiratoire**		X*			
**Succès TVS**		X*			
**Gaz du sang**		X*			
**Critères d'extubabilité**		X*			
**Date et heure extubation**		X**			
**Type d'extubation (programmée/auto-extubation/selon protocole/violation protocole)**		X**			
**Caractéristiques de la VNI post-extubation**		X***			
**Caractéristiques du désencombrement respiratoire**		X***			
**Utilisation d'oxygénothérapie à haut débit**		X***			
**Devenir en fin de journée**		X***			
Si réintubation					
**Critères de réintubation**		X			
Si trachéotomie					
**Date/heure**			X		
**Méthode**			X		
**Indication**			X		
**Déventilation**			X		
**Fin d'étude**					
**Statut respiratoire**			X	X	X
**Mortalité**			X	X	X

* Tant que le patient est intubé ou trachéotomisé

** le jour de l'extubation et pendant 24 heures

*** dans les 7 jours suivant l'extubation

7.3 Déroulement de l'étude

7.3.1 Screening

Les patients susceptibles de participer à l'étude seront identifiés au sein du service de réanimation dans le cadre de leur suivi médical régulier.

Les patients sous ventilation invasive sont screenés tous les jours pour la présence des critères préalables de sevrabilité suivants (13-15) :

- Neurologiques : réponse aux ordres simples
- Respiratoires : $FiO_2 \leq 50$ %, $SpO_2 \geq 88$ %, PEP≤5 cmH_2O, fréquence respiratoire≤35/min
- Hémodynamiques : noradrénaline<1 mg/h, dobutamine≤5 µg/kg/min

Un TVS-TT est réalisé pendant 30 minutes dès la présence de tous les critères préalables de sevrabilité comme suit :

- Patient débranché du respirateur
- Sonde d'intubation raccordée à une pièce en T
- Administration possible d'O_2 pur à un débit permettant d'obtenir une SpO_2 entre 94 et 98 % (sauf chez les patients BPCO ou la cible de SpO_2 est abaissée entre 88 et 92 %). Le débit maximal d'oxygène autorisé est de 10L/min.

Les critères d'échecs seront recherchés pendant toute la durée du TVS-TT (Table 1). Le patient est rebranché au respirateur avec les réglages antérieurs dès l'apparition d'un critère d'échec. Les patients screenés non éligibles sont colligés pour la réalisation ultérieure d'un diagramme de flux.

- Variation > 20% de la fréquence cardiaque,
- Variation > 20% de la pression artérielle systolique,
- FR > 35/min,
- SpO_2 < 88%
- Sueurs, agitation,
- Troubles de conscience,
- Signes de détresse respiratoire autres: tirage sus-claviculaire, tirage inter-costal, contraction abdominale à l'expiration, balancement thoraco-abdominal.
- pH<7.35 et PCO_2>45mmHg

Table 1. Critères d'échec du test de ventilation spontanée (adapté de (16))

En cas d'échec de ce premier TVS-TT, le patient sera informé par un médecin investigateur de l'existence du protocole (dans le cas où le patient est dans l'incapacité de recevoir cette information, celle-ci sera produite à son/ses proche(s)). Toute explication nécessaire à la bonne compréhension de l'étude sera donnée au patient et/ou son proche, ainsi qu'une lettre d'information expliquant les objectifs et le déroulement du protocole. Le praticien remettra également un formulaire de consentement aux proches du malade en double exemplaire si le patient est dans l'incapacité de donner son consentement. Il sera laissé au patient ou à ses proches le délai de réflexion nécessaire pour décider de la participation de leur proche à l'étude.
Le formulaire de consentement doit être signé avant la réalisation de toute procédure liée à l'étude.
Si le patient donne son accord de participation (ou son [ses] proche(s) si le patient est dans l'incapacité de prendre une décision éclairée), le patient (ou son [ses] proche(s)) et l'investigateur datent et signent nominativement (nom et prénom en clair) deux exemplaires du formulaire de consentement. L'un est conservé par le proche du patient, l'autre est conservé dans le classeur investigateur de l'étude. *Dans le cas où le consentement est obtenu du proche du patient, les investigateurs chercheront auprès du patient, dès que son état le permet, son consentement à la poursuite de sa participation à l'étude.*

7.3.2 *Visite d'inclusion/Randomisation*

Après vérification des critères d'éligibilité et consentement, le patient est inclus dans l'étude. Les patients sont randomisés entre deux stratégies : assistée ou non assistée en utilisant une liste de randomisation réalisée par ordinateur avec stratification sur l'existence d'une BPCO et l'existence d'une dysfonction ventriculaire gauche (cf paragraphe 4.2). L'allocation est déterminée par l'ouverture d'une enveloppe opaque et scellée, numérotée.

Dans les 6h suivant l'inclusion, et au minimum 30 minutes après le premier TVS-TT, les patients débutent le protocole de sevrage et réalise un TVS.

7.3.3 *Visites de suivi*

1) TVS quotidiens

a. Groupe assisté

Dès J1 et quotidiennement, tant que le patient est intubé et présente les critères préalables de sevrabilité, un TVS-AI d'une durée de 30 minutes est réalisé.

Le TVS-AI est réalisé comme suit :

- Respirateur réglé en VSAI
- AI : 7 cmH2O
- PEP : 5 cmH2O
- Pente de pressurisation : 200 ms
- Trigger expiratoire libre (25 % recommandé)
- Trigger inspiratoire : 2 – 5 l/min
- FiO2 ≤ 50 %
- Suppression de la ventilation d'apnée

Les critères d'échec du TVS sont monitorés en continu pendant toute la durée du TVS par l'infirmier de réanimation (Table 1.)
Quel que soit le résultat du TVS, les réglages antérieurs sont repris à la fin du TVS.
Lors du TVS sont consignés dans le CRF : la FiO2 ou le débit d'O2, les critères d'échecs si présents, la gazométrie artérielle réalisée en fin d'épreuve, l'évaluation de la dyspnée par une échelle visuelle analogique (EVA) (17).

Les critères suivants d'extubabilité sont recherchés en cas de succès du TVS :

- Force de toux
- Score d'encombrement respiratoire
- Absence d'anesthésie générale prévue dans les 24 h
- Absence d'œdème laryngé (selon le clinicien)

La force de toux est évaluée par le score SCSS en cas de succès du TVS (18) :

- 0 (pas de toux)
- 1 (mouvement d'air audible dans la sonde d'intubation sans toux audible)
- 2 (toux audible très faible)
- 3 (toux clairement audible)
- 4 (toux forte)
- 5 (plusieurs toux fortes)

Ce score est évalué en demandant au patient de tousser le plus fort possible après déconnexion du respirateur de la sonde d'intubation.

L'encombrement respiratoire est évalué comme suit, en cas de succès du TVS (19) :

- 0 (absent)
- 1 (faible quantité)
- 2 (intermédiaire)
- 3 (abondant)
- 4 (très abondant)

Le succès du TVS-AI suffit à prouver la sevrabilité du patient. Au moins 30 min après la reprise des réglages antérieurs, un TVS-TT d'une durée de 30 minutes est alors réalisé pour déterminer si le patient est à haut-risque de ré-intubation. Si les critères d'extubabilité sont présents, le patient doit être extubé au moins 2h et au maximum dans les 3 heures suivant la fin du TVS-TT.
L'application de la VNI est systématique pour les patients ayant échoué le TVS-TT, alors que pour les patients ayant réussi les deux TVS, la VNI est appliquée selon les recommandations. La VNI est appliquée sauf contre-indications (Table 2) (7).
En cas d'échec du TVS-AI, le patient reste intubé.

- patient non coopérant, agité, opposant à la technique
- intubation imminente (sauf VNI en pré-oxygénation)
- coma (sauf coma hypercapnique de l'insuffisance respiratoire chronique [IRC])
- épuisement respiratoire
- état de choc, troubles du rythme ventriculaire graves
- sepsis sévère
- immédiatement après un arrêt cardio-respiratoire
- pneumothorax non drainé, plaie thoracique soufflante
- obstruction des voies aériennes supérieures (sauf apnées du sommeil, laryngo-trachéomalacie)
- vomissements incoercibles
- hémorragie digestive haute
- traumatisme crâniofacial grave
- tétraplégie traumatique aiguë à la phase initiale

Table 2 : contre-indications à la ventilation non invasive (d'après (7))

b. Groupe non-assisté

Dès J1 et quotidiennement, tant que le patient est intubé et présente les critères préalables de sevrabilité, un TVS-TT d'une durée de 30 minutes est réalisé.
Le TVS-TT est réalisé comme suit :
- Déconnexion de la sonde d'intubation du respirateur
- Branchement d'une pièce en T
- Administration d'oxygène humidifiée à bas débit avec dispositif Venturi

Les critères d'échec du TVS sont monitorés en continu pendant toute la durée du TVS par l'infirmier de réanimation (Table 1.)
A la fin du TVS, les paramètres respiratoires antérieurs sont repris.
Lors du TVS sont consignés dans le CRF : la FiO2 ou le débit d'O2, les critères d'échecs si présents, la gazométrie artérielle réalisée en fin d'épreuve, l'évaluation de la dyspnée par une échelle visuelle analogique (EVA) (17).

Les critères suivants d'extubabilité sont recherchés en cas de succès du TVS :
- Force de toux
- Score d'encombrement respiratoire
- Absence d'anesthésie générale prévue dans les 24 h
- Absence d'œdème laryngé (selon le clinicien)

La force de toux est évaluée par le score SCSS en cas de succès du TVS (18) :
- 0 (pas de toux)
- 1 (mouvement d'air audible dans la sonde d'intubation sans toux audible)
- 2 (toux audible très faible)
- 3 (toux clairement audible)
- 4 (toux forte)
- 5 (plusieurs toux fortes)

Ce score est évalué en demandant au patient de tousser le plus fort possible après déconnexion du respirateur de la sonde d'intubation.

L'encombrement respiratoire est évalué comme suit, en cas de succès du TVS (19) :

- 0 (absent)
- 1 (faible quantité)
- 2 (intermédiaire)
- 3 (abondant)
- 4 (très abondant)

Le patient doit réussir le TVS-TT pour prouver sa sevrabilité. Si les critères d'extubabilité sont présents, le patient est extubé au moins 2 h et au maximum dans les 3 heures suivant la fin du TVS. La VNI en post-extubation est appliquée selon les recommandations, en l'absence de contre-indications (Table 2.).
En cas d'échec du TVS-TT, le patient reste intubé.

2) Extubation

Le patient est extubé selon les procédures du service.
La date et l'heure de l'extubation sont notées dans le CRF.
S'il existe une indication de VNI, celle-ci est débutée immédiatement après l'extubation.
S'il n'y pas d'indication de VNI, un support par oxygène conventionnel (pas d'oxygène haut débit) peut être débuté si besoin, QSP SpO2 94-98 % (sauf BPCO : 88-92 %).

3) VNI post-extubation

a) Critères d'introduction

- Patients du groupe assisté, qui ont échoué le TVS-TT
- Patients des deux groupes, selon les recommandations (2,5) : âge>65 ans, insuffisance respiratoire ou cardiaque chronique, PaCO2>45 mmHg en fin de TVS (TVS-TT pour les deux groupes), bronchopneumopathie chronique obstructive
- Quel que soit le groupe, les contre-indications à la technique sont listées dans la Table 2.

b) Modalités et réglages

La ventilation non invasive est réalisée via un masque naso-buccal relié au respirateur de réanimation, en mode VSAI.

- AI : entre 5 et 15 cmH2O avec pour objectif un volume courant expiré de 6-8mL/kg de poids prédit par la taille (Homme : 50 + 0,9 × [Taille (cm) − 152,4] ; Femme : 45,5 + 0,9 × [Taille (cm) − 152,4])
- PEP : entre 5 et 10 cmH2O
- AI+PEP≤20 cmH2O, idéalement moins de 15 cmH2O
- Pente : 200 ms
- Trigger expiratoire libre (25 % par défaut)
- Trigger inspiratoire : 2 – 5 l/min à adapter secondairement en fonction des auto-déclenchements éventuels
- FiO2 QSP SpO2 94-98 % (sauf BPCO : 88-92 %)
- Temps inspiratoire maximal : 1.5 sec

La VNI est appliquée 1 h à 2 h/3 h, avec un minimum de 8 h par jour de durée cumulée. La nuit, si le patient s'endort avec la VNI, il est possible de la laisser en place en continu.
Entre les séances de VNI, si besoin, support par oxygène conventionnel (pas d'oxygène haut débit), QSP SpO2 94-98 % (sauf BPCO : 88-92 %).
La VNI est appliquée pendant une durée de 24 h au minimum, sa poursuite est laissée au choix du clinicien en charge du patient.
Dans le CRF est recueillie la durée quotidienne de VNI pendant les 7 jours qui suivent l'extubation.

4) Désencombrement bronchique

Le recours à une aide instrumentale ou non instrumentale à la toux et au désencombrement bronchique est laissé au libre choix du clinicien.
Les modalités et le nombre de séances sont enregistrés dans les 7 jours suivant l'extubation.

5) Ré-intubation

Le patient est réintubé s'il présente l'un des critères respiratoire, hémodynamique ou neurologique suivants :

a) Critères respiratoires

Il est fait le diagnostic de détresse respiratoire devant deux ou plus des éléments suivants (Table 3.) :

- Fréquence respiratoire > 40/min
- Signes de lutte
- Encombrement respiratoire
- pH<7,35
- SpO2<90 % ou PaO2<60 mmHg sous FiO2 60 % (en VNI) ou > 10 l O2 au masque haute concentration.

Table 3. Critères de détresse respiratoire aiguë

Si le patient n'est pas sous VNI à ce moment (et quel que soit son groupe et indication de VNI préalable), le recours à la VNI de sauvetage n'est autorisé que s'il existe une suspicion d'OAP post-extubation ou chez les patients BPCO/hypercapniques. Le recours à une diurétisation (OAP) et aérosols de bêtamimétiques (BPCO) sont encouragés ainsi que le recours à la morphine à visée anti-dyspnée.

Dans les autres cas, le recours à la VNI est déconseillé (2,5) sauf à visée de pré-oxygénation avant intubation.

En cas d'encombrement, il est possible de recourir à des méthodes instrumentales et/ou non instrumentales de désencombrement.

En cas de dyspnée laryngée, il est possible de recourir à des traitements par nébulisation ou parentéraux à visée anti-œdémateuse.

La persistance des critères de détresse respiratoire persistant au maximum après 1 h de prise en charge implique une ré-intubation en urgence. Le diagnostic étiologique de la détresse respiratoire est noté dans le CRF ainsi que la date et l'heure de la ré-intubation.

b) Critères hémodynamiques

Le recours à des doses majeures de noradrénaline (>1 mg/h), une hyperlactatémie doivent faire discuter par le clinicien le recours à l'intubation.

c) Critères neurologiques

Des troubles de conscience (définis par un score de Glasgow <8) non rapidement réversibles doivent faire envisager une intubation (hormis le cas d'une hypercapnie importante, pouvant justifier, le recours à une VNI).

La survenue de critères de ré-intubation est tracée dans le CRF. Les thérapeutiques entreprises sont notées ainsi que l'évolution (VNI, intubation, amélioration). Dans le cas d'une intubation sur critère neurologique, le clinicien en charge du patient évalue si la dégradation neurologique est secondaire à l'état respiratoire du patient.

Le critère de jugement principal est le délai (en heures) entre l'inclusion et l'extubation avec succès (définie par l'absence de décès/ré-intubation ou la sortie vivant de réanimation dans les sept jours suivants, quel que soit le recours à la VNI). En conséquence toute ré-intubation dans les sept premiers jours post-extubation est considérée comme un échec. Après amélioration clinique, le patient reprend les modalités de sevrage de son groupe de randomisation.

En cas de ré-intubation au-delà de 7 jours, la prise en charge du patient est laissée libre, le patient étant sorti du protocole de sevrage de l'étude (extubation avec succès).

En cas d'intubation pour une intervention avec extubation immédiate (dans les 24 h) (par exemple pour une intervention chirurgicale), l'échec n'est pas retenu.

6) Auto-extubation et extubation accidentelle

En cas d'extubation non programmée, le clinicien évalue la présence de signes de détresse respiratoire (Table 2.). Quel que soit le groupe de randomisation, il est conseillé le recours à une VNI selon les recommandations (2,5). Le support par oxygène est réalisé comme décrit précédemment. Les critères de ré-intubation sont ceux décrits précédemment. En cas de ré-intubation dans les 7 jours qui suivent l'auto-extubation, le patient poursuit l'étude avec les TVS quotidiens (comme les patients ayant un échec d'extubation). Il est sinon suivi comme les autres patients (sortie du protocole de sevrage de l'étude).

7) Sevrage prolongé et trachéotomie

Il n'est pas prévu dans le cadre du protocole le recours à une trachéotomie pour le sevrage respiratoire (20).
Si le clinicien en charge du patient décide de trachéotomiser le patient, la stratégie de sevrage respiratoire décrite plus haut n'est plus appliquée, car le sevrage respiratoire via une trachéotomie est spécifique.
Pour le groupe assisté, il est alors recommandé d'envisager la décanulation ou l'arrêt du support ventilatoire (oxygénothérapie seule) dès que le patient supporte une ventilation avec une AI≤5 cmH2O et une PEP ≤5 cm H2O.
Pour le groupe non assisté, il est alors recommandé d'envisager la décanulation ou l'arrêt du support ventilatoire si le patient supporte l'absence de ventilation.
Pour les deux groupes, l'arrêt de la ventilation invasive avec succès est considéré lorsque le patient est décanulé *ou* canulé, mais sans support respiratoire (i.e, nez artificiel, oxygénothérapie seule), sans nécessité de recanulation/intubation/reventilation dans les 7 jours qui suivent. Le calcul du critère de jugement principal est réalisé de cette façon pour ces patients.
En cas de sortie de réanimation, si le patient est ventilé via une canule de trachéotomie est considéré comme une absence de sevrage, et les durées de ventilation mécanique sont censurées à la sortie de réanimation, c'est-à-dire qu'elles ne seront plus recueillies au-delà de la date de sortie de réanimation correspondant à la date de censure.

8) Limitations de soins

Si devant l'évolution du patient, le clinicien en charge du patient décide d'un arrêt des thérapeutiques actives ou d'une extubation terminale, le patient sort du protocole de l'étude.

7.3.4 Visite de fin de la recherche

A la sortie de réanimation, J28 et à J90, évaluation du statut respiratoire (Non ventilé, Ventilation non invasive (sauf CPAP), Ventilation invasive (trachéotomie), Trachéotomie (sans ventilation), Décès, Perdu de vue).
De la même façon, la mortalité est renseignée à ces dates.
J28 et J90 sont comptés depuis l'admission.
Si à J28 et J90, le patient est sorti d'hospitalisation, la vérification de son statut vital sera réalisée par contact téléphonique.

7.4 Règles d'arrêt temporaire ou définitif

- *Arrêt de participation d'une personne à la recherche :*

Les sujets pourront retirer leur consentement et demander à sortir de l'étude à n'importe quel moment et quelle qu'en soit la raison. En cas de sortie prématurée, l'investigateur doit en documenter les raisons de façon aussi complète que possible.
L'investigateur pourra interrompre temporairement ou définitivement la participation d'un sujet à l'étude pour toute raison qui servirait au mieux les intérêts du sujet en particulier en cas d'évènements indésirables graves.
La mise en évidence d'un ou plusieurs critères de sortie d'étude tels que définis précédemment pourra également amener l'investigateur à arrêter la participation du sujet à la recherche.
En cas de sujet perdu de vue, l'investigateur mettra tout en œuvre pour reprendre contact avec la personne.
En cas de retrait de consentement, les données recueillies jusqu'à la date du retrait ne seront pas analysées
Les patients exclus secondairement de l'étude sont remplacés.

- *Arrêt d’une partie ou de la totalité de la recherche :*

L’étude peut être interrompue prématurément en cas de survenue d’évènements indésirables inattendus, graves nécessitant une revue du profil de la stratégie. De même, des évènements imprévus ou de nouvelles informations relatives à la méthode d’investigation, au vu desquels les objectifs de l’étude ne seront vraisemblablement pas atteints, peuvent amener le promoteur à interrompre prématurément l’étude.
Les Hospices Civils de Lyon se réservent le droit d’interrompre l’étude à tout moment, s’il s’avère que les objectifs d’inclusion ne sont pas atteints.
En cas d’arrêt prématuré de l’étude, l’information sera transmise par le promoteur dans un délai de 15 jours à l’ANSM et au CPP.

Si un patient est de nouveau intubé lors de son séjour en réanimation, la stratégie de sevrage n’est plus appliquée en cas de :

- absence de ré-intubation dans les 7 j jours qui suivent l’extubation programmée ou non
- arrêt de soin ou limitation de soins sur la ré-intubation
- sortie de réanimation
- survenue d’un EIG avec décision de retrait du protocole par l’investigateur
- 90 jours après l’inclusion

Le patient est exclu secondairement de l’étude en cas de

- transfert dans une autre réanimation ne participant pas à l’étude
- retrait du consentement
- survenue d’un EIG avec décision de retrait du protocole par l’investigateur

Dans ce cas, les données du patient ne sont pas analysées et un patient supplémentaire sera inclus pour conserver le nombre de sujets nécessaires calculé.

En cas de décès, le CRF est rempli jusqu’à la date du décès.

8 ÉVALUATION DE LA SÉCURITÉ

Un évènement indésirable est une réaction nocive et non voulue.

Un événement indésirable grave est tout événement:
- qui entraîne la **mort** ; ou
- qui **met en danger la vie de la personne** qui se prête à la recherche ; ou
- qui nécessite une **hospitalisation ou une prolongation d'hospitalisation** ; ou
- qui provoque une **incapacité ou un handicap importants ou durables** ; ou
- qui se traduit par une **anomalie ou une malformation congénitale** ; ou
- tout **autre événement médicalement important**

D'après l'article L1123-10, les dispositions relatives aux vigilances de chaque produit ou pratique faisant l'objet de la recherche sont applicables.

L'investigateur devra signaler tout évènement indésirable selon les procédures de gestion de la qualité et des soins de son établissement.

D'après l'article L1413-14, tout professionnel ou établissement de santé ayant constaté une infection nosocomiale ou tout autre évènement indésirable grave lié à des soins réalisés lors d'investigations, de traitements ou d'actions de prévention doit en faire la déclaration au directeur général de l'agence régionale de santé.

Les évènements indésirables sanitaires sont à signaler sur le portail de signalement des évènements indésirables sanitaires : **www.signalement-sante.gouv.fr**

L'investigateur notifie au promoteur via le cahier d'observation tous les événements indésirables et les résultats d'examens de biologie médicale anormaux définis dans le protocole comme déterminants pour l'évaluation de la sécurité des personnes.
Se référer aux critères de jugement.

9 ASPECTS STATISTIQUES

9.1 Nombre de sujets nécessaires

Dans cet essai, les patients sont randomisés en 2 groupes :
- groupe assisté : stratégie basée sur le TVS-AI, un faible niveau de PEP et un dépistage des patients à haut-risque de ré-intubation par la réalisation d'un TVS-TT
- groupe non assisté : stratégie basée sur le TVS-TT, groupe de référence

Le critère de jugement principal est le délai (en heures) entre l'inclusion et l'extubation avec succès (définie par l'absence de décès ou de ré-intubation ou la sortie vivant de réanimation dans les 7 j suivant l'extubation, quel que soit le recours à la VNI). Il s'agit d'un essai contrôlé randomisé ouvert prospectif interventionnel monocentrique de supériorité (test bilatéral) comparant deux tests de ventilation spontanée utilisés dans le cadre du soin courant.

Dans le cadre d'une étude préliminaire rétrospective réalisée sur 88 patients consécutifs du service de réanimation médicale de l'hôpital de la Croix-Rousse, ventilés mécaniquement, il a été retrouvé que la durée de ventilation mécanique invasive moyenne entre le 1er TVS et l'extubation était de 22h ± 26h. Les patients

étant pris en charge avec une stratégie combinant faible niveau de PEP et d'AI lors des TVS, soit la stratégie testée dans le bras intervention (assisté) de la présente étude.
On fait l'hypothèse que, dans le groupe assisté, la médiane de durée entre l'inclusion et l'extubation avec succès est de 24 h et on considère que cette stratégie serait cliniquement pertinente si la différence de délai entre l'inclusion et l'extubation avec succès était différente d'au moins 24 heures, soit une médiane de 48h pour le groupe non assisté.

Le critère de jugement principal est évalué par une analyse de survie. Du fait du risque compétitif (décès), deux méthodologies d'analyses sont possibles (21):

- Analyse du risque cause-spécifique
- Analyse du risque en sous-distribution

Il n'est pas attendu de l'intervention qu'elle modifie la survenue du risque compétitif. En conséquence, il est décidé de recourir à une analyse du risque cause-spécifique via un modèle de Cox incluant les variables de stratification (BPCO, insuffisance ventriculaire gauche) et en censurant le risque compétitif (décès).

En prenant un risque alpha de 5%, un risque béta de 20% et un hazard ratio de 2 (basé sur le rapport des médianes de durée jusqu'à l'extubation réussie), 66 évènements sont nécessaires. Afin de tenir compte des éventuels décès et des patients non sevrés (évalués à 30%), il est nécessaire d'inclure 94 patients (soit 47 par groupe) analysables. Les patients exclus secondairement de l'étude (transferés dans une autre réanimation ne participant pas à l'étude, ou retirant leur consentement, ou en cas de survenue d'un EIG avec décision de retrait du protocole par l'investigateur), sont évalués à 10% de la population et seront remplacés, ce qui nécessite d'inclure 104 patients au maximum.

9.2 Description des méthodes statistiques

Les variables quantitatives seront rapportées en médiane et espace interquartile et comparées avec le test non paramétrique de Mann-Whitney.

Les variables qualitatives seront rapportées en nombre (pourcentage) et comparées par un test de chi-2 ou de Fischer.

Le critère de jugement principal (délai entre l'inclusion et extubation avec succès) est soumis à un risque compétitif (décès du patient/échec de sevrage). L'analyse primaire de survie est toutefois réalisée selon une analyse cause-spécifique (modèle de Cox) (21,22), avec comme covariables les statuts BPCO et insuffisance cardiaque (soit les 2 strates de randomisation). Dans l'hypothèse d'une non proportionnalité des risques, la régression de Cox sera utilisée avec prédicteur chronologique si elle est possible ou un test du log rank sera réalisé le cas échéant.
Afin de prendre en compte des risques compétitifs, des analyses complémentaires seront réalisées :le critère de jugement principal sera décrit par des courbes de fonction d'incidence cumulée et analysée par un modèle de Fine et Gray (23), en prenant pour évènement l'extubation avec succès et pour risque compétitif (le décès). Le modèle prendra en compte les risques compétitifs et les strates de randomisation.

Les durées de ventilation seront analysées par un modèle de Fine et Gray, car ils permettent de tenir compte des durées de ventilation et du décès. Les nombres de jours vivant sans ventilation mécanique invasive à J28 et J90 seront comparés via un test de Wilcoxon.

Une analyse de sous-groupe selon la cause de l'intubation et les facteurs de risque d'extubation difficile (BPCO, insuffisance cardiaque…) sera réalisée.
Une valeur de $p < 0,05$ sera prise pour définir la significativité statistique. L'analyse est réalisée en intention de traiter.

9.3 *Méthode de prise en compte des données manquantes*

Les patients ayant des données manquantes ne seront pas remplacés. Les données manquantes seront prises en compte dans les analyses multivariées par la technique des imputations multiples par appariement sur la moyenne prévisionnelle.

9.4 *Gestion des modifications apportées au plan d'analyse*

Un plan d'analyse statistique détaillé sera rédigé avant le gel de la base des données. Il tiendra compte de toute modification du protocole ou de tout évènement inattendu survenu au cours de l'étude et ayant un impact sur les analyses présentées ci-dessus. Les analyses planifiées pourront être complétées en cohérence avec les objectifs de l'étude.
Toute modification apportée par la suite au plan d'analyse statistique devra être justifiée et donnera lieu à une nouvelle version du document. Ces déviations au plan d'analyse seront reportées dans le rapport final de l'étude. L'ensemble des documents sera conservé dans le dossier de l'étude.

9.5 *Responsable des analyses et logiciel utilisé*

Dr Mehdi Mezidi et Pr Jean-Christophe Richard du Service de réanimation médicale de l'Hôpital de la Croix Rousse. Logiciel R.

10 *DROITS D'ACCÈS AUX DONNÉES ET DOCUMENTS SOURCES*

10.1 *Accès aux données*

Conformément aux BPC :

- le promoteur est chargé d'obtenir l'accord de l'ensemble des parties impliquées dans la recherche afin de garantir l'accès direct à tous les lieux de déroulement de la recherche, aux données sources, aux documents sources et aux rapports dans un but de contrôle de qualité et d'audit par le promoteur ;

- les investigateurs mettront à disposition des personnes chargées du suivi, du contrôle de qualité ou de l'audit de la recherche biomédicale, les documents et données individuelles strictement nécessaires à ce contrôle, conformément aux dispositions législatives et réglementaires en vigueur (articles L.1121-3 et R.5121-13 du code de la santé publique).

10.2 *Documents sources*

Les documents sources sont définis comme tout document ou objet original permettant de prouver l'existence ou l'exactitude d'une donnée ou d'un fait enregistrés au cours de l'étude clinique. Ils seront conservés pendant 25 ans par l'investigateur ou par l'hôpital s'il s'agit d'un dossier médical hospitalier.

Le dossier source pour cette étude sera le dossier médical informatique.

10.3 *Confidentialité des données*

Conformément aux dispositions concernant la confidentialité des données auxquelles ont accès les personnes chargées du contrôle de qualité d'une recherche biomédicale (article L.1121-3 du code de la santé publique), conformément aux dispositions relatives à la confidentialité des informations concernant notamment les essais,

les personnes qui s'y prêtent et les résultats obtenus (article R. 5121-13 du code de la santé publique), les personnes ayant un accès direct aux données prendront toutes les précautions nécessaires en vue d'assurer la confidentialité des informations relatives aux essais, aux personnes qui s'y prêtent et notamment en ce qui concerne leur identité ainsi qu'aux résultats obtenus.

Ces personnes, au même titre que les investigateurs eux-mêmes, sont soumises au secret professionnel (selon les conditions définies par les articles 226-13 et 226-14 du code pénal).
Pendant la recherche ou à son issue, les données recueillies sur les personnes qui s'y prêtent et transmises au promoteur par les investigateurs (ou tous autres intervenants spécialisés) seront rendues pseudo-anonymes.

Elles ne doivent en aucun cas faire apparaître en clair les noms des personnes concernées ni leur adresse.

Seules la première lettre du nom du sujet et la première lettre de son prénom seront enregistrées, accompagnées d'un numéro codé propre à l'étude indiquant l'ordre d'inclusion des sujets.

Le promoteur s'assurera que chaque personne qui se prête à la recherche a donné son accord par écrit pour l'accès aux données individuelles la concernant et strictement nécessaire au contrôle de qualité de la recherche.

11 CONTRÔLE ET ASSURANCE DE LA QUALITÉ

Un Attaché de Recherche Clinique (ARC) mandaté par le promoteur s'assurera de la bonne réalisation de l'étude, du recueil des données générées par écrit, de leur documentation, enregistrement et rapport, en accord avec les Procédures Opératoires Standards mises en application au sein de la DRCI des Hospices Civils de Lyon et conformément aux Bonnes Pratiques Cliniques ainsi qu'aux dispositions législatives et réglementaires en vigueur.

L'investigateur et les membres de son équipe acceptent de se rendre disponibles lors des visites de Contrôle de Qualité effectuées à intervalles réguliers par l'Attaché de Recherche Clinique. Lors de ces visites, les éléments suivants pourront être revus en fonction du niveau de monitoring adapté à l'étude et déterminé conformément aux POS du Promoteur :
A minima :

- consentement éclairé

Toute visite fera l'objet d'un rapport de monitoring par compte-rendu écrit adressé à l'investigateur du centre visité et à la structure de coordination de la recherche.

D'autre part, les investigateurs s'engagent à accepter les audits d'assurance qualité effectués par des personnes mandatées par le promoteur ainsi que les inspections effectuées par les Autorités Compétentes. Toutes les données, tous les documents et rapports peuvent faire l'objet d'audits et d'inspections réglementaires sans que puisse être opposé le secret médical.

12 CONSIDÉRATIONS ÉTHIQUES

12.1 Autorités compétentes

Le protocole, la notice d'information et le formulaire de consentement de l'étude seront soumis pour avis au Comité de Protection des Personnes Ile de France VI (01 42 16 16 83).
La notification de l'avis favorable du CPP sera transmise au promoteur de l'étude. Le promoteur transmettra cet avis ainsi qu'un résumé du projet à l'ANSM.
Le promoteur s'engage à ce que le démarrage de l'étude ne se fasse qu'après obtention de l'avis favorable du CPP.

12.2 Modifications substantielles

En cas de modification substantielle apportée au protocole par l'investigateur, elle sera approuvée par le promoteur. Ce dernier devra obtenir préalablement à sa mise en œuvre un avis favorable du CPP. Un nouveau consentement des personnes participant à la recherche sera recueilli si nécessaire.

12.3 Information du patient et formulaire de consentement écrit

Les proches des patients seront informés de façon complète et loyale, en des termes compréhensibles, des objectifs et des contraintes de l'étude, des risques éventuels encourus, des mesures de surveillance et de sécurité nécessaires, de leurs droits de refuser de participer à l'étude ou de la possibilité de se rétracter à tout moment.

Toutes ces informations figurent sur un formulaire d'information et de consentement remis au proche du patient. Le consentement libre, éclairé et écrit du proche du patient sera recueilli par l'investigateur, ou un médecin qui le représente avant l'inclusion définitive dans l'étude. Une copie du formulaire d'information et de consentement signé par les deux parties sera remise au proche du patient, l'investigateur en conservera l'original.

Si le patient est compétent au moment de l'inclusion dans l'étude, le consentement sera recherché auprès de lui selon les modalités décrites précédemment. Au cas où le consentement ait été obtenu auprès d'un proche, la recherche du consentement du patient à la poursuite de l'étude sera effectuée dès que le patient sera compétent.

12.4 Déclaration de conformité

Le promoteur et l'investigateur s'engagent à ce que cette recherche soit conduite :
- conformément au protocole,
- conformément aux bonnes pratiques cliniques françaises et internationales actuellement en vigueur,
- conformément aux dispositions législatives et réglementaires actuellement en vigueur en France et au niveau international.

12.5 Période d'exclusion

Durant la durée de l'étude, le patient ne peut pas participer à d'autres études ayant trait au sevrage respiratoire.

12.6 Indemnisation des sujets et inscription au fichier national des personnes se prêtant à une recherche biomédicale

Il n'est pas prévu d'indemniser les volontaires qui accepteront de participer à l'étude. L'objet de la recherche étant en rapport avec l'état pathologique des sujets, l'inscription au fichier national des Volontaires se prêtant à une Recherche Biomédicale (VRB) n'est pas requise.

13 GESTION ET CONSERVATION DES DONNÉES

13.1 Cahier d'observation

Le cahier d'observation ne comportera que les données nécessaires à l'analyse en vue de publication. Les autres données relatives au patient et nécessaires à son suivi en dehors de l'étude, seront colligées dans son dossier médical.

Toutes les informations requises par le protocole doivent être consignées sur les cahiers d'observation et une explication doit être apportée pour chaque donnée manquante. Les données devront être recueillies au fur et à mesure qu'elles sont obtenues, et transcrites dans ces cahiers de façon nette et lisible.
Les données erronées relevées sur les cahiers d'observation seront clairement barrées et les nouvelles données seront copiées, à côté de l'information barrée, accompagnées des initiales, de la date et éventuellement d'une justification par l'investigateur ou la personne autorisée qui aura fait la correction.

13.2 Gestion des données

La simple saisie des données sera réalisée par un opérateur dans une base de données Excel, sécurisée par mot de passe complexe.
Les données sont validées par l'investigateur coordinateur.
La base de données est gelée après la saisie du dernier patient et après la vérification et correction éventuelle des données manquantes/aberrantes.
Après le gel de la base de données, une impression papier est réalisée et contresignée par les investigateurs.
L'ensemble des données est sauvegardé chaque soir, conservé pendant 4 semaines, puis archivé mensuellement sur disque dur.

13.3 CNIL

Cette étude entre dans le cadre de la « Méthodologie de Référence » (MR-001) en application des dispositions de l'article 54 alinéa 5 de la loi n° 78-17 du 6 janvier 1978 modifiée relative à l'informatique, aux fichiers et aux libertés. Ce changement a été homologué par décision du 5 janvier 2006 et modifiée le 21 juillet 2016. Les Hospices Civils de Lyon, promoteur de l'étude, ont signé un engagement de conformité à cette « Méthodologie de Référence ».

13.4 Archivage

Les documents suivants seront archivés par le nom de l'étude dans les locaux du service de réanimation médicale de l'Hôpital de la Croix Rousse des Hospices Civils de Lyon jusqu'à la fin de la période d'utilité pratique. Ces documents sont :

- Protocole et annexes, amendements éventuels,
- Formulaires d'information et consentements originaux signés
- Données individuelles (copies authentifiées de données brutes)
- Documents de suivi et courriers relatifs à la recherche
- Analyses statistiques
- Rapport final de l'étude

Le promoteur conservera l'ensemble des données de l'étude pendant 25 ans.

Aucun déplacement ou destruction ne pourra être effectué sans l'accord du promoteur. Au terme des 25 ans, le promoteur sera consulté pour destruction. Toutes les données, tous les documents et rapports pourront faire l'objet d'audit ou d'inspection.

14 FINANCEMENT ET ASSURANCE

14.1 Budget de l'étude

Les frais liés à cette recherche seront pris en charge sur des fonds propres des Hospices Civils de Lyon.
Aucun surcout hospitalier n'est engendré par la mise en œuvre de ce protocole car les stratégies sont déjà utilisées.

14.2 Assurance

Le promoteur a souscrit pour toute la durée de l'étude une assurance garantissant sa propre responsabilité civile ainsi que celle de tout médecin impliqué dans la réalisation de l'étude. Il assurera également l'indemnisation intégrale des conséquences dommageables à la recherche pour la personne qui s'y prête et ses ayants droit, sauf preuve à sa charge que le dommage n'est pas imputable à sa faute ou à celle de tout intervenant, sans que puisse être opposé le fait d'un tiers ou le retrait volontaire de la personne qui avait initialement consenti à se prêter à la recherche.
Le contrat d'assurance a été souscrit avant le démarrage de l'étude auprès de la Société Hospitalière d'Assurance Mutuelle, 18 rue Edouard Rochet, 69 008 Lyon, sous le numéro 159.077.

15 RÈGLES RELATIVES À LA PUBLICATION

Les communications et rapports scientifiques correspondant à cette étude seront réalisés sous la responsabilité de l'investigateur principal de l'étude avec l'accord des investigateurs associés. Les coauteurs du rapport et des publications seront les investigateurs et les cliniciens impliqués, ainsi que le biostatisticien et les chercheurs associés. Le Dr Mezidi sera premier auteur, le Pr Richard dernier auteur, et les autres coauteurs seront listés au prorata de leur contribution à l'étude.
Les règles de publications suivront les recommandations internationales (N Engl J Med, 1997 ; 336 : 309-315).
L'étude sera enregistrée sur un registre des essais cliniques en libre accès (clinicaltrials.gov) avant l'inclusion du 1er patient.

16 RÉFÉRENCES BIBLIOGRAPHIQUES

1. Beduneau G, Pham T, Schortgen F, Piquilloud L, Zogheib E, Jonas M, et al. Epidemiology of Weaning Outcome according to a New Definition. The WIND Study. Am J Respir Crit Care Med. 3rd ed. 2017 Mar 15;195(6):772–83.

2. Schmidt GA, Girard TD, Kress JP, Morris PE, Ouellette DR, Alhazzani W, et al. Official Executive Summary of an American Thoracic Society/American College of Chest Physicians Clinical Practice Guideline: Liberation from Mechanical Ventilation in Critically Ill Adults. Am J Respir Crit Care Med. 2017 Jan;195(1):115–9.

3. Burns KEA, Soliman I, Adhikari NKJ, Zwein A, Wong JTY, Gomez-Builes C, et al. Trials directly comparing alternative spontaneous breathing trial techniques: a systematic review and meta-analysis. Crit Care. Critical Care; 2017 May 26;21(1):1–11.

4. Cabello B, Thille AW, Roche-Campo F, Brochard L, Gómez FJ, Mancebo J. Physiological comparison of three spontaneous breathing trials in difficult-to-wean patients. Intensive Care Med. 2010 Mar 30;36(7):1171–9.

5. SFAR, SRLF. Recommendations Formalisées d'Experts Intubation et extubation du patient de réanimation. 2016. Available from: https://www.srlf.org/wp-content/uploads/2016/09/20160927_RFE_Intubation-Extubation.pdf

6. Thille AW, Boissier F, Ben-Ghezala H, Razazi K, Mekontso-Dessap A, Brun-Buisson C, et al. Easily identified at-risk patients for extubation failure may benefit from noninvasive ventilation: a prospective before-after study. Crit Care. Critical Care; 2016 Feb 22;20(1):1–8.

7. SFAR, SPLF, SRLF. Conférence de Consensus commune : Ventilation non invasive au cours de l'insuffisance respiratoire aiguë (nouveau-né exclu). 2006 Nov;:1–8.

8. Liu J, Shen F, Teboul J-L, Anguel N, Beurton A, Bezaz N, et al. Cardiac dysfunction induced by weaning from mechanical ventilation: incidence, risk factors, and effects of fluid removal. Crit Care. BioMed Central; 2016 Dec 1;20(1):369.

9. Sklar MC, Burns K, Rittayamai N, Lanys A, Rauseo M, Chen L, et al. Effort to Breathe with Various Spontaneous Breathing Trial Techniques. A Physiologic Meta-analysis. Am J Respir Crit Care Med. 2017 Jun;195(11):1477–85.

10. Burns KEA, Meade MO, Premji A, Adhikari NKJ. Noninvasive positive-pressure ventilation as a weaning strategy for intubated adults with respiratory failure. Cochrane Anaesthesia, Critical and Emergency Care Group, editor. Cochrane Database Syst Rev. 2013 Dec 9;24(12):CD004127.

11. Perkins GD, Mistry D, Gates S, Gao F, Snelson C, Hart N, et al. Effect of Protocolized Weaning With Early Extubation to Noninvasive Ventilation vs Invasive Weaning on Time to Liberation From Mechanical Ventilation Among Patients With Respiratory Failure. JAMA. 2018 Oct 22;:1–8.

12. Schoenfeld DA, Bernard GR, ARDS Network. Statistical evaluation of ventilator-free days as an efficacy measure in clinical trials of treatments for acute respiratory distress syndrome. Crit Care Med. 2002 Aug;30(8):1772–7.

13. Krishnan JA, Moore D, Robeson C, Rand CS, Fessler HE. A Prospective, Controlled Trial of a Protocol-based Strategy to Discontinue Mechanical Ventilation. Am J Respir Crit Care Med. 2004 Mar 15;169(6):673–8.

14. Navalesi P, Frigerio P, Moretti MP, Sommariva M, Vesconi S, Baiardi P, et al. Rate of reintubation in mechanically ventilated neurosurgical and neurologic patients: Evaluation of a systematic approach to weaning and extubation. Crit Care Med. 2008 Nov;36(11):2986–92.

15. Girard TD, Alhazzani W, Kress JP, Ouellette DR, Schmidt GA, Truwit JD, et al. An Official American Thoracic Society/American College of Chest Physicians Clinical Practice Guideline: Liberation from Mechanical Ventilation in Critically Ill Adults. Rehabilitation Protocols, Ventilator Liberation Protocols, and Cuff Leak Tests. Am J Respir Crit Care Med. 2017 Jan;195(1):120–33.

16. Boles J-M, Bion J, Connors A, Herridge M, Marsh B, Melot C, et al. Weaning from mechanical ventilation. European Respiratory Journal. 2007 May 1;29(5):1033–56.

17. Bausewein C, Farquhar M, Booth S, Gysels M, Higginson IJ. Measurement of breathlessness in advanced disease: A systematic review. Respiratory Medicine. 2007 Mar;101(3):399–410.

18. Khamiees M, Raju P, DeGirolamo A, Amoateng-Adjepong Y, Manthous CA. Predictors of Extubation Outcome in Patients Who Have Successfully Completed a Spontaneous Breathing Trial. Chest. 2001 Oct;120(4):1262–70.

19. Thille AW, Boissier F, Ben-Ghezala H, Razazi K, Mekontso-Dessap A, Brun-Buisson C. Risk Factors for and Prediction by Caregivers of Extubation Failure in ICU Patients. Crit Care Med. 2015 Mar;43(3):613–20.

20. Young D, Harrison DA, Cuthbertson BH, Rowan K, TracMan Collaborators. Effect of early vs late tracheostomy placement on survival in patients receiving mechanical ventilation: the TracMan randomized trial. JAMA. 2013 May 22;309(20):2121–9.

21. Tai B-C, Wee J, Machin D. Analysis and design of randomised clinical trials involving competing risks endpoints. Trials. BioMed Central Ltd; 2011 May 19;12(1):127.

22. Resche-Rigon M, Azoulay E, Chevret S. Evaluating mortality in intensive care units: contribution of competing risks analyses. Crit Care. 2006 Feb;10(1):R5.

23. Fine JP, Gray RJ. A Proportional Hazards Model for the Subdistribution of a Competing Risk. Journal of the American Statistical Association. 1999 Jun;94(446):496–509.

17 LISTE DES ANNEXES

Annexe 1 - Notice d'information et formulaire de consentement patient
Annexe 2 - Notice d'information et formulaire de consentement proche
Annexe 3- Notice d'information et formulaire de consentement patient – poursuite d'étude
