# Supplementary material for: Pressure support and positive end-expiratory pressure versus T-piece during spontaneous breathing trial in difficult weaning from mechanical ventilation: study protocol for the SBT-ICU study
Source: Trials. 2022 Dec 12;23:993. doi: 10.1186/s13063-022-06896-4 (PMC9742015; doi:10.1186/s13063-022-06896-4)
Supplement: Supplementary file 3 — Additional file 3. [file 13063_2022_6896_MOESM3_ESM.docx]

| 1. **Primary Registry and Trial Identifying Number** | ClinicalTrials.gov:  NCT03861117 |
| --- | --- |
| 1. **Date of Registration in Primary Registry** | March 1, 2019. |
| 1. **Secondary Identifying Numbers** |  |
| - - Identifiers assigned by the sponsor | Hospices Civils de Lyon: 69HCL18_0982 |
| - - Identifiers issued by ethics committees | IDRCB : 2019-A00106-51 |
| 1. **Source(s) of Monetary or Material Support** Major source(s) of monetary or material support for the trial (e.g. funding agency, foundation, company, institution). | Hospices Civils de Lyon |
| 1. **Primary Sponsor** | Hospices Civils de Lyon |
| 1. **Secondary Sponsor(s)** | None |
| 1. **Contact for Public Queries** | Alexandre PACHOT  E-mail: [alexandre.pachot@chu-lyon.fr](mailto:alexandre.pachot@chu-lyon.fr)  Tel.: 04 72 40 68 40  Address : Direction de la Recherche Clinique – Hospices Civils de Lyon. 3 quai des Célestins 69229 Lyon cedex 02 , FRANCE |
| 1. **Contact for Scientific Queries** | Principal Investigator  Dr Mehdi MEZIDI  E-mail: [mehdi.mezidi@chu-lyon.fr](mailto:mehdi.mezidi@chu-lyon.fr)  Tel.: 04 72 07 17 62  Address : Service de Médecine Intensive Réanimation.103 grande rue de la Croix-Rousse. 69004 Lyon, France  Scientific contact :  Pr Jean-Christophe RICHARD  E-mail: [j-christophe.richard@chu-lyon.fr](mailto:j-christophe.richard@chu-lyon.fr)  Tel.: 04 72 07 17 62  Address : Service de Médecine Intensive Réanimation.103 grande rue de la Croix-Rousse. 69004 Lyon, FRANCE |
| 1. **Public Title** | Pressure Support and Positive End-Expiratory Pressure versus T-piece during Spontaneous Breathing Trial in difficult weaning from mechanical ventilation |
| 1. **Scientific Title** | Pressure Support and Positive End-Expiratory Pressure versus T-piece during Spontaneous Breathing Trial in difficult weaning from mechanical ventilation (SBT-ICU) |
| 1. **Countries of Recruitment** | France |
| 1. **Health Condition(s) or Problem(s) Studied** | Critically ill patients with difficult weaning from mechanical ventilation |
| 1. **Intervention(s)** | Control group : unassisted weaning strategy based solely on spontaneous breathing trial with T-Piece  Intervention group : assisted weaning strategy based on spontaneous breathing trial with pressure support and positive end-expiratory pressure and spontaneous breathing trial with T-Piece to identify patients at high risk of extubation failure and trigger the use of prophylactic post-extubation non-invasive ventilation |
| 1. **Key Inclusion and Exclusion Criteria** Inclusion and exclusion criteria for participant selection, including age and sex. Other selection criteria may relate to clinical diagnosis and co-morbid conditions; exclusion criteria are often used to ensure patient safety.  If the study is conducted in healthy human volunteers not belonging to the target population (e.g. a preliminary safety study), enter "healthy human volunteer". | Inclusion criteria:   - Patient aged 18 years or older - Intubated and ventilated in the intensive care unit for more than 24 hours - Patient ready for weaning evaluation - Failure of a first SBT-TP   Exclusion criteria:   - Chronic neuromuscular disease - Guillain-Barré Syndrome - Central nervous system disease with consciousness disorder (i.e inability to obey a simple command) - Tracheostomy - Chronic disease with life expectancy less than 1 year - Pregnancy, breast feeding - Withholding life support regarding a reintubation - Prisoner or patient interned in a psychiatric hospital - Guardianship - Language barrier - Lack of medical insurance - Lack of the patient's consent (or of the next of kin where appropriate) - Patient under an exclusion period after enrollment in another research study |
| 1. **Study Type** Study type consists of: | Interventional monocentric prospective open labeled, randomized controlled superiority trial, with two parallel groups and balanced randomization using a randomization list stratified into 3 strata (according to patients prior diseases), with a 1:1 ratio, using random blocks of size 4, 6 and 8. |
| 1. **Date of First Enrollment** | 10/05/2019 |
| 1. **Sample Size** |  |
| - - Number of participants that the trial plans to enroll in total. | 94 |
| - - Number of participants that the trial has enrolled. | 87 |
| 1. **Recruitment Status** Recruitment status of this trial: | Still recruiting on July 11^th^, 2022 |
| 1. **Primary Outcome(s)** |  |
| - - The name of the outcome | Time to successful extubation |
| - - The metric or method of measurement used (be as specific as possible) | Duration (in hours) between study inclusion and successful extubation (defined by the absence of reintubation or death within the 7 days following extubation). Reintubation lasting less than 24h related to unplanned surgical procedure will not be considered as extubation failure. Patients extubated but exiting ICU within the 7 days following extubation will be considered as successful extubation. In patients not meeting extubation success criteria, data will be censored at day-90 or date of death whichever comes first |
| - - The timepoint(s) of primary interest | Day 90 or earlier if successful extubation or death |
| 1. **Key Secondary Outcomes** Secondary outcomes are outcomes which are of secondary interest or that are measured at timepoints of secondary interest. A secondary outcome may involve the same event, variable, or experience as the primary outcome, but measured at timepoints other than those of primary interest.  As for primary outcomes, for each secondary outcome provide: |  |
| - - The name of the outcome   - The metric or method of measurement used   - The timepoint(s) of interest | First successful extubation  Percentage of included patients  Day 90 or earlier if successful extubation or death |
| - - The name of the outcome   - The metric or method of measurement used   - The timepoint(s) of interest | Invasive mechanical ventilation duration  Hours  Day 90 or earlier if successful extubation or death |
| - - The name of the outcome   - The metric or method of measurement used   - The timepoint(s) of interest | Mechanical (invasive and non-invasive) ventilation duration  Hours  Day 90 or earlier if successful extubation or death |
| - - The name of the outcome   - The metric or method of measurement used   - The timepoint(s) of interest | Time from inclusion to successful extubation  Days  Mechanical ventilation |
| - - The name of the outcome   - The metric or method of measurement used   - The timepoint(s) of interest | Ventilator-free days at Day-28 and Day-90  Days  Day-28 and Day-90, respectively |
| - - The name of the outcome   - The metric or method of measurement used   - The timepoint(s) of interest | ICU length of stay between inclusion and ICU discharge  Days  ICU discharge |
| - - The name of the outcome   - The metric or method of measurement used   - The timepoint(s) of interest | Hospital length of stay between inclusion and hospital discharge  Days  Hospital discharge |
| - - The name of the outcome   - The metric or method of measurement used   - The timepoint(s) of interest | ICU, Day-28, Day-90 mortality  Percentage of included patients  ICU discharge, Day-28, Day-90, respectively |
| - - The name of the outcome   - The metric or method of measurement used   - The timepoint(s) of interest | Reintubation rate  Percentage of included patients  Day 90 or earlier if successful extubation or death |
| 1. **Ethics Review**: |  |
| - - Status | Approved |
| - - Date of approval | February 20th, 2019 |
| - - Name and contact details of Ethics committee(s) | Groupe Hospitalier Pitié-Salpêtrière - 4 bâtiment de la Force - 47, boulevard de l’Hôpital - 75013 PARIS France |
| 1. **Completion date** | Ongoing trial |
| 1. **Summary Results** It consists of: |  |
| - - Date of posting of results summaries | Planned on fourth quarter of 2022 |
| - - Date of the first journal publication of results | Planned on fourth quarter of 2022 |
| - - URL hyperlink(s) related to results and publications | Planned on fourth quarter of 2022 |
| - - Baseline Characteristics: Data collected at the beginning of a clinical study for all participants and for each arm or comparison group. These data include demographics, such as age and sex, and study-specific measures. | Will be included in journal publication of the study results |
| - - Participant flow: Information to document the progress and numbers of research participants through each stage of a study in a flow diagram or tabular format. | Will be included in journal publication of the study results |
| - - Adverse events: An unfavorable change in the health of a participant, including abnormal laboratory findings, and all serious adverse events and deaths that happen during a clinical study or within a certain time period after the study has ended. This change may or may not be caused by the intervention being studied. | Will be included in journal publication of the study results |
| - - Outcome measures: A table of data for each primary and secondary outcome measure and their respective measurement of precision (eg a 95% confidence interval) by arm (that is, initial assignment of participants to arms or groups) or comparison group (that is, analysis groups), including the result(s) of scientifically appropriate statistical analyses that were performed on the outcome measure data, if any. | Will be included in journal publication of the study results |
| - - URL link to protocol file(s) with version and date | Is included in the current publication |
| - - Brief summary | Will be included in journal publication of the study results |
| 1. **IPD sharing statement** Statement regarding the intended sharing of deidentified individual clinical trial participant-level data (IPD). Should indicate whether or not IPD will be shared, what IPD will be shared, when, by what mechanism, with whom and for what types of analyses. It consists of: |  |
| - - Plan to share IPD (Yes, No) | IPD of the whole dataset will be shared on reasonable request. |
| - - Plan description | Plan description will be shared on reasonable request. |
