# Supplementary material for: Pressure support and positive end-expiratory pressure versus T-piece during spontaneous breathing trial in difficult weaning from mechanical ventilation: study protocol for the SBT-ICU study
Source: Trials. 2022 Dec 12;23:993. doi: 10.1186/s13063-022-06896-4 (PMC9742015; doi:10.1186/s13063-022-06896-4)
Supplement: Supplementary file 4 — Additional file 4. [file 13063_2022_6896_MOESM4_ESM.docx]

| Cough strength (ref.) | Abundancy or respiratory secretions (ref) |
| --- | --- |
| 0: no cough on command  1: audible movement of air through the endotracheal tube but no audible cough  2: weakly (barely) audible cough  3: clearly audible cough  4: stronger cough  5: multiple sequential strong coughs | 0 (absent)  1 (low quantity)  2 (intermediate)  3 (abundant)  4 (very abundant) |

Additional table: extubability criteria
