# Supplementary material for: Pressure support and positive end-expiratory pressure versus T-piece during spontaneous breathing trial in difficult weaning from mechanical ventilation: study protocol for the SBT-ICU study
Source: Trials. 2022 Dec 12;23:993. doi: 10.1186/s13063-022-06896-4 (PMC9742015; doi:10.1186/s13063-022-06896-4)
Supplement: Supplementary file 5 — Additional file 5. [file 13063_2022_6896_MOESM5_ESM.pdf]

CRITERES DE REINTUBATION

CRITERES RESPIRATOIRES

Détresse respiratoire devant deux ou plus des éléments suivants :

- Fréquence respiratoire > 40/min
- Signes de lutte
- Encombrement respiratoire
- pH<7,35
- SpO2<90 % ou PaO2<60 mmHg sous FiO2 60 % (en VNI) ou 10 l O2 au masque haute concentration.

VNI de sauvetage seulement si suspicion d'OAP post-extubation ou chez les patients BPCO/hypercapniques. Diurétisation (OAP) et aérosols de béta-mimétiques (BPCO) sont encouragés ainsi que le recours à la morphine à visée anti-dyspnée.

Autres cas : VNI déconseillé sauf à visée de pré-oxygénation avant intubation.

En cas d'encombrement : méthodes invasives et non invasives de désencombrement.

En cas de dyspnée laryngée, traitements par nébulisation ou parentéraux à visée anti-œdémateuse.

Détresse respiratoire persistant au maximum après 1 h de PEC implique une réintubation en urgence.

Le diagnostic étiologique de la détresse respiratoire est noté dans le CRF ainsi que la date et l'heure de la réintubation.

CRITERES HEMODYNAMIQUES

Le recours à des doses majeures de noradrénaline (>1 µg/kg/min), une lactacidémie non contrôlée doivent faire discuter par le clinicien le recours à l'intubation.

CRITERES NEUROLOGIQUES

Des troubles de conscience (définis par un score de Glasgow <8) non rapidement réversibles doivent faire envisager une intubation (hormis le cas d'une hypercapnie importante, pouvant justifier, le recours à une VNI).

EXTUBATION NON PROGRAMMEE

Gérer comme une extubation programmée vis-à-vis de l'indication de VNI et de réintubation

CALENDRIER DE L'ETUDE

	Inclusion (J1)	J1-J90	Sortie/Décès	J28 +/- 7jours	J90 +/- 7 jours
Données démographiques	X				
Si patient intubé					
Présence des critères de sevrabilité		X*			
Score de toux		X*			
Score d'encombrement respiratoire		X*			
Succès TVS		X*			
Gaz du sang		X*			
Critères d'extubabilité		X*			
Date et heure extubation		X**			
Type d'extubation (programmée/auto-extubation/selon protocole/violation protocole)		X**			
Caractéristiques de la VNI post-extubation		X***			
Caractéristiques du désencombrement respiratoire		X***			
Utilisation d'oxygénothérapie à haut débit		X***			
Devenir en fin de journée		X***			
Si réintubation					
Critères de réintubation		X			
Si trachéotomie					
Date/heure			X		
Méthode			X		
Indication			X		
Déventilation			X		
Fin d'étude					
Statut respiratoire			X	X	X
Mortalité			X	X	X

CONTACTS

Investigateur principal : Dr Mehdi Mezidi
Téléphone : 04 26 10 92 64
Email : mehdi.mezidi@chu-lyon.fr
Attachée de recherche clinique : Loredana BABOI
Tel : 04 26 10 92 65
Email : loredana.baboi@chu-lyon.fr

SBT-ICU

Impact de la combinaison de l'aide inspiratoire et de la pression expiratoire positive pendant l'épreuve de sevrage respiratoire en comparaison de la pièce en T sur le délai jusqu'à l'extubation avec succès

CRITERES D'INCLUSION

1) Sujet majeur de 18 ans ou plus
2) Intubé et ventilé mécaniquement en réanimation depuis plus de 24 h
3) Présence des critères préalables de sevrabilité (cf.)
4) Échec pièce en T (→ faite à <u>tous</u> les patients du service)

CRITERES D'EXCLUSION

1) Patient atteint d'une pathologie neuro-musculaire chronique (sclérose latérale amyotrophique, myopathie, myasthénie…)
2) Patient ventilé pour syndrome de Guillain-Barré
3) Pathologie du système nerveux central (accident vasculaire cérébral récent, arrêt cardiaque avec pronostic neurologique défavorable, encéphalopathie…) responsable de troubles de conscience (définis par une absence de réponse aux ordres simples)
4) Patient porteur d'une-trachéotomie
5) Maladie chronique sous-jacente fatale en moins d'un an
6) Femme enceinte ou allaitante
7) Limitation de soins sur la ré-intubation
8) Personnes privées de liberté par décision judiciaire ou administrative ; Personnes majeures protégées
9) Incompréhension linguistique de la personne qui sera chargée de recevoir l'information
10) Absence de couverture sociale
11) Absence de consentement du patient s'il est en état de le donner ou de ses proches le cas échéant
12) Participation à d'autres études ayant trait au sevrage respiratoire

INCLUSION-RANDOMISATION

- Recueil du consentement du patient ou du proche
- Remplir CRF papier
- Déterminer le sous-groupe du patient :
  - Patients ayant une BPCO (+/- ICC (FE<45%)) = sous-groupe BPCO
  - Patients ayant une ICC sans BPCO = sous-groupe ICC
  - Patients n'ayant ni BPCO ni ICC = sous-groupe Autre
- Ouvrir l'enveloppe de randomisation selon le sous-groupe

A J1 DANS LES 6H SUIVANT L'INCLUSION PUIS QUOTIDIENNEMENT

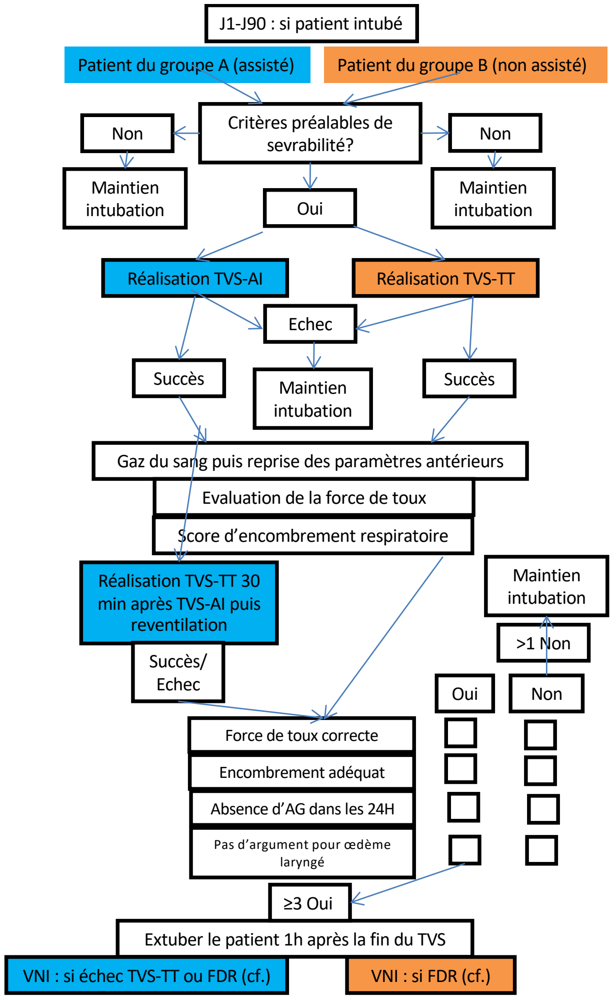

DEFINITIONS DE L'ETUDE

CRITERES PREALABLES DE SEVRABILITE

- Neurologiques : réponse aux ordres simples
- Respiratoires : $FiO_2 \leq 50$ %, $SpO_2 \geq 88$ %, PEP≤5 cmH2O, fréquence respiratoire≤35/min
- Hémodynamiques : noradrénaline<1 mg/h, dobutamine≤5 µg/kg/min

CRITERES D'ECHEC DES TVS

- Variation >20% de la FC
- Variation >20% de la PAS
- FR>35/min
- SpO2<88%
- Sueurs, agitation
- Troubles de conscience
- Signes autres de DRA : tirage sus-claviculaire ou inter-costal, contraction abdominale à l'expiration, balancement thoraco-abdominal
- pH<7.35 et pCO2>45mmHg

EVALUATION DE LA TOUX (→APRES SUCCES DU TVS)

0. pas de toux
1. mvt d'air audible dans la sonde d'IOT sans toux audible
2. toux audible très faible
3. toux clairement audible
4. toux forte
5. plusieurs toux fortes

Score réalisé après déconnexion du patient du respirateur en lui demandant de tousser le plus fort possible.

EVALUATION DE L'ENCOMBREMENT BRONCHIQUE

0. Absent
1. Faible quantité
2. Intermédiaire
3. Abondant
4. Très abondant

METHODES DE L'ETUDE

TVS-AI

Respirateur réglé en VSAI
AI : 7 cmH2O PEP : 5 cmH2O
Pente de pressurisation : 200 ms
Trigger expiratoire libre (25 % recommandé)
Trigger inspiratoire : 2 – 5 l/min
FiO2 ≤ 50 %
Suppression de la ventilation d'apnée
Durée 30 minutes

TVS-TT

Patient débranché du respirateur
Sonde d'intubation raccordée à une pièce en T
Administration possible d'O2 pur à un débit permettant d'obtenir une SpO2 [94–98]% (sauf BPCO, SpO2 [88–92]%).
Débit maximal d'oxygène 10L/min.
Durée 30 minutes

INDICATIONS DE VNI PROPHYLACTIQUE POST-EXTUBATION

Groupe A (assisté) :

- Si TVS-TT négatif
- Si âge>65 ans, insuffisance respiratoire ou cardiaque chronique, PaCO2>45 mmHg en fin de TVS-TT, BPCO

Groupe B (non assisté)

- Si âge>65 ans, insuffisance respiratoire ou cardiaque chronique, PaCO2>45 mmHg en fin de TVS-TT, BPCO

MODALITES DE LA VNI POST-EXTUBATION

Masque naso-buccal relié au respirateur de réanimation, en mode VSAI.
AI : 5-15 cmH2O, objectif VTe de 6-8mL/kg PPT
PEP : 5-10 cmH2O
AI+PEP≤20 cmH2O, idéalement moins de 15 cmH2O
Pente : 200 ms
Trigger expiratoire libre (25 % par défaut)
Trigger inspiratoire : 2 – 5 l/min à adapter secondairement en fonction des auto-déclenchements éventuels
FiO2 QSP SpO2 94-98 % (sauf BPCO : 88-92 %)
Temps inspiratoire maximal 1.5 sec
Séances 1 h à 2 h/3 h, avec un minimum de 8 h/j de durée cumulée. La nuit, si le patient s'endort avec la VNI, il est possible de la laisser en place en continu.
La VNI est appliquée pendant une durée de 24 h au minimum, sa poursuite est laissée au choix du clinicien en charge du patient.

OXYGENOTHERAPIE POST-EXTUBATION

Support par oxygène conventionnel (pas d'oxygène haut débit), QSP SpO2 94-98 % (sauf BPCO : 88-92 %)
