# Supplementary material for: Pressure support and positive end-expiratory pressure versus T-piece during spontaneous breathing trial in difficult weaning from mechanical ventilation: study protocol for the SBT-ICU study
Source: Trials. 2022 Dec 12;23:993. doi: 10.1186/s13063-022-06896-4 (PMC9742015; doi:10.1186/s13063-022-06896-4)
Supplement: Supplementary file 6 — Additional file 6. [file 13063_2022_6896_MOESM6_ESM.pdf]

IMPACT DE LA COMBINAISON DE L'AIDE INSPIRATOIRE ET DE LA PRESSION EXPIRATOIRE POSITIVE PENDANT L'EPREUVE DE SEVRAGE RESPIRATOIRE EN COMPARAISON DE LA PIECE EN T SUR LE DELAI JUSQU'A L'EXTUBATION AVEC SUCCES

SBT-ICU

CAHIER D'OBSERVATION

INITIALES DU PATIENT (*): |__|__|__|

(*) 1ère lettre PRENOM -Tiret -1ère lettre NOM
Exemple : Jean Dupont : J-D
Jean-Paul Dupont : JPD

N° PATIENT |__|__|__|

RANDOMISATION :

Bras A (stratégie assistée) ☐

Bras B (stratégie non assistée) ☐

STRATIFICATION

BPCO (+/- insuffisance cardiaque chronique) ☐

Insuffisance cardiaque chronique sans BPCO ☐

Pas de BPCO et pas d'insuffisance cardiaque chronique ☐

Investigateur Coordonnateur :
Dr Mehdi Mezidi
Service de réanimation médicale
Hôpital de la Croix-Rousse
103 Grande rue de la Croix Rousse
69004 LYON
Tél : 04 26 10 92 75 ; Fax : 04 72 07 17 74
Email : mehdi.mezidi@chu-lyon.fr

Promoteur de l'étude : Hospices Civils de Lyon
Direction de la Recherche Clinique et de l'Innovation
3, quai des Célestins
BP 2251
69229 Lyon Cedex 02
www.chu-lyon.fr

ADRESSES UTILES

Investigateur principal :
Dr Mehdi Mezidi
Service de réanimation médicale
Hôpital de la Croix-Rousse
103 Grande rue de la Croix Rousse
69004 LYON
Tél : 04 26 10 92 75 ; Fax : 04 72 07 17 74
Email : mehdi.mezidi@chu-lyon.fr

Modalités de remplissage des cahiers d'observation

Pour des raisons techniques, veuillez écrire de façon lisible en utilisant un stylo bille noire.

1. Pour les dates, toujours suivre la séquence : j j m m a a a a
2. Pour des valeurs numériques, inscrire un chiffre par case et cadrer à droite : 0 4 0
3. Cocher les cases avec une croix : ☒
4. Si une donnée a été inscrite par erreur, veuillez barrer la mauvaise réponse d'un seul trait de manière à la laisser lisible, inscrire la réponse correcte en marge, parapher et dater la correction :

   ~~0 2~~ 1 3 23/07/2004

5. Utiliser le système horaire de 24 heures : 1 4 H 2 5
6. Pour les valeurs définitivement manquantes, écrire ND (pour No Data) dans les cases N D
7. Monogramme = première lettre du prénom + première lettre du nom séparées par un tiret J - B

   ou pour les prénoms composés : premières lettres des prénoms (sans tiret) première lettre du nom. J P B

DEROULEMENT DE L'ETUDE

Déroulement

1. Explication de la recherche au patient/à son proche avec présentation de la notice d'information si non fait précédemment
2. Recueil du consentement du patient (ou de son proche). Noter le recueil du consentement du proche et/ou du patient à la participation du patient à l'étude dans son dossier médical (Easily Recherche)
3. Vérifier les critères d'inclusion et de non inclusion
4. Compléter la liste d'identification (dans le classeur investigateur)
5. Compléter le tableau des inclusions (dans le classeur investigateur) et le faxer au secteur promotion (04.72.11.51.90) de la Direction de la Recherche Clinique et de l'Innovation des HCL
6. Remplir les pages correspondant à la visite
7. Compléter le bordereau de fin d'étude
8. Remplir la page de signature et la signer
9. Compléter le tableau de recueil des évènements indésirables si applicable

VISITE D'INCLUSION

Date de la visite : |__|__| |__|__| |__|__|__|__| (jj/mm/aaaa)

Date du consentement : |__|__| |__|__| |__|__|__|__| (jj/mm/aaaa)

ELIGIBILITE

Critères d'inclusion			
N°		OUI	NON
01	Sujet majeur de 18 ans ou plus	☐	☐
02	Intubé et ventilé mécaniquement en réanimation depuis plus de 24 h	☐	☐
03	Présence des critères préalables de sevrabilité :<br>- Neurologiques : réponse aux ordres simples<br>- Respiratoires : $FiO_2 \leq 50$ %, $SpO_2 \geq 88$ %, PEP≤5 cmH_2O, fréquence respiratoire≤35/min<br>- Hémodynamiques : noradrénaline<1 mg/h, dobutamine≤5 µg/kg/min	☐	☐
04	Échec du premier test de ventilation spontanée avec tube en T (TVS-TT)	☐	☐

SI L'UNE DES REPONSES EST NON, LE PATIENT NE PEUT PAS ETRE INCLUS DANS L'ETUDE

Critères de non inclusion			
N°		OUI	NON
01	Patient atteint d'une pathologie neuro-musculaire chronique (sclérose latérale amyotrophique, myopathie, myasthénie…)	☐	☐
02	Patient ventilé pour syndrome de Guillain-Barré	☐	☐
03	Pathologie du système nerveux central (accident vasculaire cérébral récent, arrêt cardiaque avec pronostic neurologique défavorable, encéphalopathie…) responsable de troubles de conscience (définis par une absence de réponse aux ordres simples)	☐	☐
04	Patient porteur d'une trachéotomie	☐	☐
05	Maladie chronique sous-jacente fatale en moins d'un an	☐	☐
06	Femme enceinte ou allaitante	☐	☐
07	Limitation de soins sur la réintubation	☐	☐
08	Personnes privées de liberté par décision judiciaire ou administrative	☐	☐
09	Personnes majeures protégées		
10	Incompréhension linguistique de la personne qui sera chargée de recevoir l'information	☐	☐
11	Absence de couverture sociale	☐	☐
12	Absence de consentement du patient s'il est en état de le donner ou de ses proches le cas échéant	☐	☐
13	Participation à d'autres études ayant trait au sevrage respiratoire		

SI L'UNE DES REPONSES EST OUI, LE PATIENT NE PEUT PAS ETRE INCLUS DANS L'ETUDE

A l'issue de la visite d'inclusion, le patient est-il incluable dans l'essai ? Oui ☐ Non ☐

Si **NON**, remplir directement la page sortie d'étude et informer le promoteur.

RANDOMISATION

Bras A (stratégie assistée) ☐

Bras B (stratégie non assistée) ☐

STRATIFICATION

BPCO (+/- insuffisance cardiaque chronique) ☐
Insuffisance cardiaque chronique sans BPCO ☐
Pas de BPCO et pas d'insuffisance cardiaque chronique ☐

N° enveloppe : ……………………………….

➔ Cocher les cases correspondantes sur la couverture du CRF

Etude SBT-ICU
Impact of the combination of pressure support and positive end-expiratory pressure during spontaneous breathing trial on the time to successful extubation

FAX D'INCLUSION

LE JOUR DE LA VISITE D'INCLUSION,

COMPLETER CE DOCUMENT ET LE FAXER A :

☞ Au promoteur :DRCI des HCL   04.72.11.51.90

DATE DE LA VISITE D'INCLUSION : /___/___/-/___/___/-/___/___/___/___/

INITIALES : /___/___/___/
1ère lettre du prénom - 1ère lettre du nom

NUMERO DU PATIENT : /___/___/___/

SEXE : ☐ **F** ☐ **M**

AGE / _ _ _ /

Validation des critères d'inclusion et non inclusion :
☐ **OUI** ☐ **NON**

Bras de randomisation :
☐ **Bras A (stratégie assistée)** ☐ **Bras B (stratégie non-assistée)**

Nom de l'investigateur : Signature
Numéro de fax :
Date :

DONNEES A L'INCLUSION

HEURE D'INCLUSION+++++ : /___/___/H/___/___/

Initiales patient: |__|__|__|
(1ère lettre du prénom + première lettre du nom séparées par un tiret ou pour les prénoms composés : 1ère lettres des prénoms (sans tiret) première lettre du nom)

Date de naissance : |__|__| / |__|__|__|__| (mm/aaaa)

Sexe : F ☐ M ☐

Taille : |__|__|__| cm Poids : |__|__|__| kg

Date entrée à l'hôpital (jj/mm/aaaa)	|__|__| |__|__| |__|__|__|__|
Date entrée en réanimation (jj/mm/aaaa)	|__|__| |__|__| |__|__|__|__|
Date intubation (jj/mm/aaaa)	|__|__| |__|__| |__|__|__|__|
Heure intubation (hh:mm)	|__|__| : |__|__|
Cause(s) d'intubation (plusieurs réponses possibles)	☐ Respiratoire (SDRA, décompensation respiratoire…)<br>☐ Hémodynamique (état de choc…)<br>☐ Neurologique (coma…)<br>☐ Post-opératoire lourd<br>☐ Autre(en clair) : …………………………
Contexte d'admission	☐ médical ☐ chirurgical réglé<br>☐ chirurgical urgent ☐ traumatologique
Trachéotomie	☐ oui ☐non
Score IGS 2 dans les premières 24h de l'*admission en réanimation*	|__|__|__|
Score SOFA à l'*inclusion (données du jour calendaire)*	• Rapport PaO2/FiO2<br>☐≥400 ☐<400 ☐<300 ☐<200 ☐100<br>• Bilirubine (µmol/L)<br>☐<20 ☐≥20 ☐≥33 ☐≥102 ☐≥204<br>• Glasgow coma scale<br>☐15 ☐13-14 ☐10-12 ☐6-9 ☐<6<br>• Hémodynamique<br>☐ PAM ≥70 ☐PAM<70 ☐ Dobutamine<br>☐NAD≤0,1µg/kg/min ☐NAD>0,1µg/kg/min<br>• Créatininémie (µmol/L)<br>☐<110 et diurèse ≥ 500 ml<br>☐≥110 et diurèse ≥ 500 ml<br>☐≥171 et diurèse ≥ 500 ml<br>☐≥300 ou diurèse<500mL/j)<br>☐>442 ou diurèse<200mL/j)<br>• Plaquettes (G/L)<br>☐≥150 ☐<150 ☐<100 ☐<50 ☐<20

Antécédents	
BPCO	☐Non ☐Suspectée ☐Prouvée (EFR)<br>VEMS : ☐ inconnu ☐≥80%<br>☐50-80% ☐30-50%<br>☐<30% (ou <50% et PaO2 en air <50mmHg)
Insuffisance respiratoire chronique	☐Non ☐Oui<br>Si OUI : ☐Obstructive ☐Restrictive ☐Mixte
Oxygénothérapie ou VNI au long cours	☐Non ☐OLD☐ VNI☐
Insuffisance cardiaque chronique (FEVG<45%)	☐Non ☐Oui
Score de comorbidités de Charlson (cf. annexe)	|__|__|__|
Paramètres ventilatoires	
Mode ventilatoire à l'inclusion	☐VAC ☐VSAI ☐Autre
Ventilation minute expirée (L)	|__|__|__|,|__|
Fréquence respiratoire (/min)	|__|__|__|
PEP (cmH2O)	|__|__|
FiO2 (%)	|__|__|__|
Gazométrie artérielle avant le TVS-TT	pH |__|,|__|__| PaCO2 |__|__|__|(☐mmHg ☐kPa)<br>PaO2 |__|__|__|(☐mmHg ☐kPa)<br>HCO3 |__|__|,|__|(mmol/L)<br>Excès de base |__|__|__|,|__|(mmol/L)<br>Lactate |__|__|,|__|(mmol/L)

J1 (=inclusion) = |__|__|/|__|__/|__|__| de 0h à 23h59

- Critères préalables de sevrabilité : Présents (-> Faire TVS) ☐ Absents☐
- TVS : Succès ☐ Echec ☐ (maintien intubation)
- Si succès :
  - Gaz du sang : pH |__|,|__|__|. pCO2 |__|__|__|mmHg pO2 |__|__|__|mmHg FiO2|__|__|__|%
  - Score de toux : 0 ☐ 1 ☐ 2 ☐ 3 ☐ 4 ☐ 5 ☐
  - Score d'encombrement : 0 ☐ 1 ☐ 2 ☐ 3 ☐ 4 ☐
  - Patients du groupe A (assisté) : TVS-TT Succès ☐ Echec ☐ (-> VNI prophylactique)
- Evaluer extubabilité si succès du TVS :
  - Force de toux acceptable (≥3) : Oui ☐ Non ☐
  - Encombrement acceptable (≤2) : Oui ☐ Non ☐
  - Absence d'AG dans les 24h qui suivent : Oui ☐ Non ☐
  - Pas d'argument pour une obstruction laryngée : Oui ☐ Non ☐

➔ Si ≥3 critères « oui » : extubation proposée :

➔ Heure d'extubation (hh :mm): |__|__|:|__|__|

Et raison de la non-extubation le cas échéant : ______________________________

Patient extubé AUJOURD'HUI : OUI ☐ NON ☐.

- Extubation : Programmée selon protocole☐ Programmée avec violation de protocole☐ auto-extubation☐
- VNI prophylactique post-extubation OUI ☐ NON ☐
  - *Si VNI prophylactique*
  - selon protocole ☐ (TVS-TT (-)☐ âge>65ans☐ BPCO☐ Insuffisance cardiaque chronique ☐ Insuffisance respiratoire chronique☐, PaCO2>45 en fin de TVS-TT☐) violation protocole ☐
  - Durée cumulée sur les 24h : |__|__|h
  - *Si absence de VNI prophylactique*
  - selon protocole ☐ violation protocole ☐ contre-indication ☐
- VNI curative post-extubation NON ☐ selon protocole ☐ violation protocole ☐
- Optiflow post-extubation (non recommandé) OUI ☐ NON ☐
- Désencombrement bronchique : NON ☐ Toux manuellement assistée ☐ Toux assistée instrumentale ☐

Réintubation ce jour : OUI☐ (Heure : |__|__|:|__|__|) NON ☐
(critère(s) Neurologique☐ Hémodynamique ☐ autre ☐
Respiratoire ☐ (épuisement ☐ Encombrement ☐ Pneumopathie☐ OAP☐ Dyspnée laryngée☐))
Réintubation pour chirurgie avec intubation <24h OUI☐ NON ☐

A la fin de J1 le patient est :
Intubé ☐ Extubé ☐
Poursuite du protocole de sevrage ☐
Sortie du protocole de sevrage ☐ (remplir la partie « sortie d'essai »)
Poids du jour : |__|__|__|kg

J2 = |__|__|/|__|__/|__|__| de 0h à 23h59

Remplir si patient intubé le matin de J2

- Critères préalables de sevrabilité : Présents (-> Faire TVS) ☐ Absents☐
- TVS : Succès ☐ Echec ☐ (maintien intubation)
- Si succès :
  - Gaz du sang : pH |__|,|__|__|. pCO2 |__|__|__|mmHg pO2 |__|__|__|mmHg FiO2|__|__|__|%
  - Score de toux : 0 ☐ 1 ☐ 2 ☐ 3 ☐ 4 ☐ 5 ☐
  - Score d'encombrement : 0 ☐ 1 ☐ 2 ☐ 3 ☐ 4 ☐
  - Patients du groupe A (assisté) : TVS-TT Succès ☐ Echec ☐ (-> VNI prophylactique)
- Evaluer extubabilité si succès du TVS :
  - Force de toux acceptable (≥3) : Oui ☐ Non ☐
  - Encombrement acceptable (≤2) : Oui ☐ Non ☐
  - Absence d'AG dans les 24h qui suivent : Oui ☐ Non ☐
  - Pas d'argument pour une obstruction laryngée : Oui ☐ Non ☐

➔ Si ≥3 critères « oui » : extubation proposée :

➔ Heure d'extubation (hh :mm): |__|__|:|__|__|

Et raison de la non-extubation le cas échéant : ____________________________

Remplir si le patient n'est plus intubé à un moment de J2 (même quelques minutes ou depuis plusieurs jours)

Patient extubé AUJOURD'HUI OUI ☐ NON ☐

Sinon Date de la dernière extubation |__|__|/|__|__|/|__|__| Heure |__|__|:|__|__|

- Extubation (si réalisée AUJOURD'HUI)
  - programmée☐ (selon protocole☐ violation protocole☐)
  - auto-extubation ☐
- VNI prophylactique post-extubation OUI ☐ NON ☐
  - *Si VNI prophylactique*
  - selon protocole ☐ (TVS-TT (-)☐ âge>65ans☐ BPCO☐ Insuffisance cardiaque chronique ☐ Insuffisance respiratoire chronique☐, PaCO2>45 en fin de TVS-TT☐) violation protocole ☐
  - Durée cumulée sur les 24h : |__|__|h
  - *Si absence de VNI prophylactique*
  - selon protocole ☐ violation protocole ☐ contre-indication ☐
- VNI curative post-extubation NON ☐ selon protocole ☐ violation protocole ☐
- Optiflow post-extubation (non recommandé) OUI ☐ NON ☐
- Désencombrement bronchique : NON ☐ Toux manuellement assistée ☐ Toux assistée instrumentale ☐

Réintubation ce jour : OUI ☐ NON ☐ : si OUI Heure : |__|__|:|__|__|

(critère(s) Neurologique☐ Hémodynamique ☐ autre ☐

Respiratoire ☐ (épuisement ☐ Encombrement ☐ Pneumopathie☐ OAP☐ Dyspnée laryngée☐))

Réintubation pour chirurgie avec intubation <24h OUI☐ NON ☐

A la fin de J2 le patient est :

Intubé ☐ Extubé ☐

Poursuite du protocole de sevrage ☐

Sortie du protocole de sevrage ☐ (remplir la partie « sortie d'essai »)

Poids du jour : |__|__|__|kg

J3 = |__|__|/|__|__/|__|__| de 0h à 23h59

Remplir si patient intubé le matin de J3

- Critères préalables de sevrabilité : Présents (-> Faire TVS) ☐ Absents☐
- TVS : Succès ☐ Echec ☐ (maintien intubation)
- Si succès :
  - Gaz du sang : pH |__|,|__|__|. pCO2 |__|__|__|mmHg pO2 |__|__|__|mmHg FiO2|__|__|__|%
  - Score de toux : 0 ☐ 1 ☐ 2 ☐ 3 ☐ 4 ☐ 5 ☐
  - Score d'encombrement : 0 ☐ 1 ☐ 2 ☐ 3 ☐ 4 ☐
  - Patients du groupe A (assisté) : TVS-TT Succès ☐ Echec ☐ (-> VNI prophylactique)
- Evaluer extubabilité si succès du TVS :
  - Force de toux acceptable (≥3) : Oui ☐ Non ☐
  - Encombrement acceptable (≤2) : Oui ☐ Non ☐
  - Absence d'AG dans les 24h qui suivent : Oui ☐ Non ☐
  - Pas d'argument pour une obstruction laryngée : Oui ☐ Non ☐

➔ Si ≥3 critères « oui » : extubation proposée :

➔ Heure d'extubation (hh :mm): |__|__|:|__|__|

Et raison de la non-extubation le cas échéant : ______________________________

Remplir si le patient n'est plus intubé à un moment de J3 (même quelques minutes ou depuis plusieurs jours)

Patient extubé AUJOURD'HUI OUI ☐ NON ☐

Sinon Date de la dernière extubation |__|__|/|__|__|/|__|__| Heure |__|__|:|__|__|

- Extubation (si réalisée AUJOURD'HUI)
  - programmée☐ (selon protocole☐ violation protocole☐)
  - auto-extubation ☐
- VNI prophylactique post-extubation OUI ☐ NON ☐
  - *Si VNI prophylactique*
  - selon protocole ☐ (TVS-TT (-)☐ âge>65ans☐ BPCO☐ Insuffisance cardiaque chronique ☐ Insuffisance respiratoire chronique☐, PaCO2>45 en fin de TVS-TT☐) violation protocole ☐
  - Durée cumulée sur les 24h : |__|__|h
  - *Si absence de VNI prophylactique*
  - selon protocole ☐ violation protocole ☐ contre-indication ☐
- VNI curative post-extubation NON ☐ selon protocole ☐ violation protocole ☐
- Optiflow post-extubation (non recommandé) OUI ☐ NON ☐
- Désencombrement bronchique : NON ☐ Toux manuellement assistée ☐
  Toux assistée instrumentale ☐

Réintubation ce jour : OUI ☐ NON ☐ : si OUI Heure : |__|__|:|__|__|
(critère(s) Neurologique☐ Hémodynamique ☐ autre ☐
Respiratoire ☐ (épuisement ☐ Encombrement ☐ Pneumopathie☐ OAP☐ Dyspnée laryngée☐))
Réintubation pour chirurgie avec intubation <24h OUI☐ NON ☐

A la fin de J3 le patient est :
Intubé ☐ Extubé ☐
Poursuite du protocole de sevrage ☐
Sortie du protocole de sevrage ☐ (remplir la partie « sortie d'essai »)
Poids du jour : |__|__|__|kg

J4 = |__|__|/|__|__/|__|__| de 0h à 23h59

Remplir si patient intubé le matin de J4

- Critères préalables de sevrabilité : Présents (-> Faire TVS) ☐ Absents ☐
- TVS : Succès ☐ Echec ☐ (maintien intubation)
- Si succès :
  - Gaz du sang : pH |__|,|__|__|. pCO2 |__|__|__|mmHg pO2 |__|__|__|mmHg FiO2|__|__|__|%
  - Score de toux : 0 ☐ 1 ☐ 2 ☐ 3 ☐ 4 ☐ 5 ☐
  - Score d'encombrement : 0 ☐ 1 ☐ 2 ☐ 3 ☐ 4 ☐
  - Patients du groupe A (assisté) : TVS-TT Succès ☐ Echec ☐ (-> VNI prophylactique)
- Evaluer extubabilité si succès du TVS :
  - Force de toux acceptable (≥3) : Oui ☐ Non ☐
  - Encombrement acceptable (≤2) : Oui ☐ Non ☐
  - Absence d'AG dans les 24h qui suivent : Oui ☐ Non ☐
  - Pas d'argument pour une obstruction laryngée : Oui ☐ Non ☐

➔ Si ≥3 critères « oui » : extubation proposée :

➔ Heure d'extubation (hh :mm): |__|__|:|__|__|

Et raison de la non-extubation le cas échéant : ____________________________

Remplir si le patient n'est plus intubé à un moment de J4 (même quelques minutes ou depuis plusieurs jours)

Patient extubé AUJOURD'HUI OUI ☐ NON ☐

Sinon Date de la dernière extubation |__|__|/|__|__|/|__|__| Heure |__|__|:|__|__|

- Extubation (si réalisée AUJOURD'HUI)
  - programmée☐ (selon protocole☐ violation protocole☐)
  - auto-extubation ☐
- VNI prophylactique post-extubation OUI ☐ NON ☐
  - *Si VNI prophylactique*
  - selon protocole ☐ (TVS-TT (-)☐ âge>65ans☐ BPCO☐ Insuffisance cardiaque chronique ☐ Insuffisance respiratoire chronique☐, PaCO2>45 en fin de TVS-TT☐) violation protocole ☐
  - Durée cumulée sur les 24h : |__|__|h
  - *Si absence de VNI prophylactique*
  - selon protocole ☐ violation protocole ☐ contre-indication ☐
- VNI curative post-extubation NON ☐ selon protocole ☐ violation protocole ☐
- Optiflow post-extubation (non recommandé) OUI ☐ NON ☐
- Désencombrement bronchique : NON ☐ Toux manuellement assistée ☐ Toux assistée instrumentale ☐

Réintubation ce jour : OUI ☐ NON ☐ : si OUI Heure : |__|__|:|__|__|

(critère(s) Neurologique☐ Hémodynamique ☐ autre ☐

Respiratoire ☐ (épuisement ☐ Encombrement ☐ Pneumopathie☐ OAP☐ Dyspnée laryngée☐))

Réintubation pour chirurgie avec intubation <24h OUI☐ NON ☐

A la fin de J4 le patient est :

Intubé ☐ Extubé ☐

Poursuite du protocole de sevrage ☐

Sortie du protocole de sevrage ☐ (remplir la partie « sortie d'essai »)

Poids du jour : |__|__|__|kg

J5 = |__|__|/|__|__/|__|__| de 0h à 23h59

Remplir si patient intubé le matin de J5

- Critères préalables de sevrabilité : Présents (-> Faire TVS) ☐ Absents ☐
- TVS : Succès ☐ Echec ☐ (maintien intubation)
- Si succès :
  - Gaz du sang : pH |__|,|__|__|. pCO2 |__|__|__| mmHg pO2 |__|__|__| mmHg FiO2 |__|__|__| %
  - Score de toux : 0 ☐ 1 ☐ 2 ☐ 3 ☐ 4 ☐ 5 ☐
  - Score d'encombrement : 0 ☐ 1 ☐ 2 ☐ 3 ☐ 4 ☐
  - Patients du groupe A (assisté) : TVS-TT Succès ☐ Echec ☐ (-> VNI prophylactique)
- Evaluer extubabilité si succès du TVS :
  - Force de toux acceptable (≥3) : Oui ☐ Non ☐
  - Encombrement acceptable (≤2) : Oui ☐ Non ☐
  - Absence d'AG dans les 24h qui suivent : Oui ☐ Non ☐
  - Pas d'argument pour une obstruction laryngée : Oui ☐ Non ☐

➔ Si ≥3 critères « oui » : extubation proposée :

➔ Heure d'extubation (hh :mm): |__|__|:|__|__|

Et raison de la non-extubation le cas échéant : ____________________________

Remplir si le patient n'est plus intubé à un moment de J5 (même quelques minutes ou depuis plusieurs jours)

Patient extubé AUJOURD'HUI OUI ☐ NON ☐

Sinon Date de la dernière extubation |__|__|/|__|__|/|__|__| Heure |__|__|:|__|__|

- Extubation (si réalisée AUJOURD'HUI)
  - programmée ☐ (selon protocole ☐ violation protocole ☐)
  - auto-extubation ☐
- VNI prophylactique post-extubation OUI ☐ NON ☐
  - *Si VNI prophylactique*
  - selon protocole ☐ (TVS-TT (-) ☐ âge>65ans ☐ BPCO ☐ Insuffisance cardiaque chronique ☐ Insuffisance respiratoire chronique ☐, PaCO2>45 en fin de TVS-TT ☐) violation protocole ☐
  - Durée cumulée sur les 24h : |__|__| h
  - *Si absence de VNI prophylactique*
  - selon protocole ☐ violation protocole ☐ contre-indication ☐
- VNI curative post-extubation NON ☐ selon protocole ☐ violation protocole ☐
- Optiflow post-extubation (non recommandé) OUI ☐ NON ☐
- Désencombrement bronchique : NON ☐ Toux manuellement assistée ☐
  Toux assistée instrumentale ☐

Réintubation ce jour : OUI ☐ NON ☐ : si OUI Heure : |__|__|:|__|__|

(critère(s) Neurologique ☐ Hémodynamique ☐ autre ☐

Respiratoire ☐ (épuisement ☐ Encombrement ☐ Pneumopathie ☐ OAP ☐ Dyspnée laryngée ☐))

Réintubation pour chirurgie avec intubation <24h OUI ☐ NON ☐

A la fin de J5 le patient est :

Intubé ☐ Extubé ☐

Poursuite du protocole de sevrage ☐

Sortie du protocole de sevrage ☐ (remplir la partie « sortie d'essai »)

Poids du jour : |__|__|__| kg

J6 = |__|__|/|__|__/|__|__| de 0h à 23h59

Remplir si patient intubé le matin de J6

- Critères préalables de sevrabilité : Présents (-> Faire TVS) ☐ Absents☐
- TVS : Succès ☐ Echec ☐ (maintien intubation)
- Si succès :
  - Gaz du sang : pH |__|,|__|__|. pCO2 |__|__|__|mmHg pO2 |__|__|__|mmHg FiO2|__|__|__|%
  - Score de toux : 0 ☐ 1 ☐ 2 ☐ 3 ☐ 4 ☐ 5 ☐
  - Score d'encombrement : 0 ☐ 1 ☐ 2 ☐ 3 ☐ 4 ☐
  - Patients du groupe A (assisté) : TVS-TT Succès ☐ Echec ☐ (-> VNI prophylactique)
- Evaluer extubabilité si succès du TVS :
  - Force de toux acceptable (≥3) : Oui ☐ Non ☐
  - Encombrement acceptable (≤2) : Oui ☐ Non ☐
  - Absence d'AG dans les 24h qui suivent : Oui ☐ Non ☐
  - Pas d'argument pour une obstruction laryngée : Oui ☐ Non ☐

➔ Si ≥3 critères « oui » : extubation proposée :

➔ Heure d'extubation (hh :mm): |__|__|:|__|__|

Et raison de la non-extubation le cas échéant : ____________________________

Remplir si le patient n'est plus intubé à un moment de J6 (même quelques minutes ou depuis plusieurs jours)

Patient extubé AUJOURD'HUI OUI ☐ NON ☐

Sinon Date de la dernière extubation |__|__|/|__|__|/|__|__| Heure |__|__|:|__|__|

- Extubation (si réalisée AUJOURD'HUI)
  - programmée☐ (selon protocole☐ violation protocole☐)
  - auto-extubation ☐
- VNI prophylactique post-extubation OUI ☐ NON ☐
  - *Si VNI prophylactique*
  - selon protocole ☐ (TVS-TT (-)☐ âge>65ans☐ BPCO☐ Insuffisance cardiaque chronique ☐ Insuffisance respiratoire chronique☐, PaCO2>45 en fin de TVS-TT☐) violation protocole ☐
  - Durée cumulée sur les 24h : |__|__|h
  - *Si absence de VNI prophylactique*
  - selon protocole ☐ violation protocole ☐ contre-indication ☐
- VNI curative post-extubation NON ☐ selon protocole ☐ violation protocole ☐
- Optiflow post-extubation (non recommandé) OUI ☐ NON ☐
- Désencombrement bronchique : NON ☐ Toux manuellement assistée ☐ Toux assistée instrumentale ☐

Réintubation ce jour : OUI ☐ NON ☐ : si OUI Heure : |__|__|:|__|__|

(critère(s) Neurologique☐ Hémodynamique ☐ autre ☐

Respiratoire ☐ (épuisement ☐ Encombrement ☐ Pneumopathie☐ OAP☐ Dyspnée laryngée☐))

Réintubation pour chirurgie avec intubation <24h OUI☐ NON ☐

A la fin de J6 le patient est :

Intubé ☐ Extubé ☐

Poursuite du protocole de sevrage ☐

Sortie du protocole de sevrage ☐ (remplir la partie « sortie d'essai »)

Poids du jour : |__|__|__|kg

J7 = |__|__|/|__|__/|__|__| de 0h à 23h59

Remplir si patient intubé le matin de J7

- Critères préalables de sevrabilité : Présents (-> Faire TVS) ☐ Absents ☐
- TVS : Succès ☐ Echec ☐ (maintien intubation)
- Si succès :
  - Gaz du sang : pH |__|,|__|__|. pCO2 |__|__|__|mmHg pO2 |__|__|__|mmHg FiO2|__|__|__|%
  - Score de toux : 0 ☐ 1 ☐ 2 ☐ 3 ☐ 4 ☐ 5 ☐
  - Score d'encombrement : 0 ☐ 1 ☐ 2 ☐ 3 ☐ 4 ☐
  - Patients du groupe A (assisté) : TVS-TT Succès ☐ Echec ☐ (-> VNI prophylactique)
- Evaluer extubabilité si succès du TVS :
  - Force de toux acceptable (≥3) : Oui ☐ Non ☐
  - Encombrement acceptable (≤2) : Oui ☐ Non ☐
  - Absence d'AG dans les 24h qui suivent : Oui ☐ Non ☐
  - Pas d'argument pour une obstruction laryngée : Oui ☐ Non ☐

➔ Si ≥3 critères « oui » : extubation proposée :

➔ Heure d'extubation (hh :mm): |__|__|:|__|__|

Et raison de la non-extubation le cas échéant : ____________________________

Remplir si le patient n'est plus intubé à un moment de J7 (même quelques minutes ou depuis plusieurs jours)

Patient extubé AUJOURD'HUI OUI ☐ NON ☐

Sinon Date de la dernière extubation |__|__|/|__|__|/|__|__| Heure |__|__|:|__|__|

- Extubation (si réalisée AUJOURD'HUI)
  - programmée ☐ (selon protocole ☐ violation protocole ☐)
  - auto-extubation ☐
- VNI prophylactique post-extubation OUI ☐ NON ☐
  - *Si VNI prophylactique*
  - selon protocole ☐ (TVS-TT (-) ☐ âge>65ans ☐ BPCO ☐ Insuffisance cardiaque chronique ☐ Insuffisance respiratoire chronique ☐, PaCO2>45 en fin de TVS-TT ☐) violation protocole ☐
  - Durée cumulée sur les 24h : |__|__|h
  - *Si absence de VNI prophylactique*
  - selon protocole ☐ violation protocole ☐ contre-indication ☐
- VNI curative post-extubation NON ☐ selon protocole ☐ violation protocole ☐
- Optiflow post-extubation (non recommandé) OUI ☐ NON ☐
- Désencombrement bronchique : NON ☐ Toux manuellement assistée ☐
  Toux assistée instrumentale ☐

Réintubation ce jour : OUI ☐ NON ☐ : si OUI Heure : |__|__|:|__|__|

(critère(s) Neurologique ☐ Hémodynamique ☐ autre ☐

Respiratoire ☐ (épuisement ☐ Encombrement ☐ Pneumopathie ☐ OAP ☐ Dyspnée laryngée ☐))

Réintubation pour chirurgie avec intubation <24h OUI ☐ NON ☐

A la fin de J7 le patient est :

Intubé ☐ Extubé ☐

Poursuite du protocole de sevrage ☐

Sortie du protocole de sevrage ☐ (remplir la partie « sortie d'essai »)

Poids du jour : |__|__|__|kg

J8 = |__|__|/|__|__/|__|__| de 0h à 23h59

Remplir si patient intubé le matin de J8

- Critères préalables de sevrabilité : Présents (-> Faire TVS) ☐ Absents☐
- TVS : Succès ☐ Echec ☐ (maintien intubation)
- Si succès :
  - Gaz du sang : pH |__|,|__|__|. pCO2 |__|__|__|mmHg pO2 |__|__|__|mmHg FiO2|__|__|__|%
  - Score de toux : 0 ☐ 1 ☐ 2 ☐ 3 ☐ 4 ☐ 5 ☐
  - Score d'encombrement : 0 ☐ 1 ☐ 2 ☐ 3 ☐ 4 ☐
  - Patients du groupe A (assisté) : TVS-TT Succès ☐ Echec ☐ (-> VNI prophylactique)
- Evaluer extubabilité si succès du TVS :
  - Force de toux acceptable (≥3) : Oui ☐ Non ☐
  - Encombrement acceptable (≤2) : Oui ☐ Non ☐
  - Absence d'AG dans les 24h qui suivent : Oui ☐ Non ☐
  - Pas d'argument pour une obstruction laryngée : Oui ☐ Non ☐

➔ Si ≥3 critères « oui » : extubation proposée :

➔ Heure d'extubation (hh :mm): |__|__|:|__|__|

Et raison de la non-extubation le cas échéant : ____________________________

Remplir si le patient n'est plus intubé à un moment de J8 (même quelques minutes ou depuis plusieurs jours)

Patient extubé AUJOURD'HUI OUI ☐ NON ☐

Sinon Date de la dernière extubation |__|__|/|__|__|/|__|__| Heure |__|__|:|__|__|

- Extubation (si réalisée AUJOURD'HUI)
  - programmée☐ (selon protocole☐ violation protocole☐)
  - auto-extubation ☐
- VNI prophylactique post-extubation OUI ☐ NON ☐
  - *Si VNI prophylactique*
  - selon protocole ☐ (TVS-TT (-)☐ âge>65ans☐ BPCO☐ Insuffisance cardiaque chronique ☐ Insuffisance respiratoire chronique☐, PaCO2>45 en fin de TVS-TT☐) violation protocole ☐
  - Durée cumulée sur les 24h : |__|__|h
  - *Si absence de VNI prophylactique*
  - selon protocole ☐ violation protocole ☐ contre-indication ☐
- VNI curative post-extubation NON ☐ selon protocole ☐ violation protocole ☐
- Optiflow post-extubation (non recommandé) OUI ☐ NON ☐
- Désencombrement bronchique : NON ☐ Toux manuellement assistée ☐ Toux assistée instrumentale ☐

Réintubation ce jour : OUI ☐ NON ☐ : si OUI Heure : |__|__|:|__|__|

(critère(s) Neurologique☐ Hémodynamique ☐ autre ☐

Respiratoire ☐ (épuisement ☐ Encombrement ☐ Pneumopathie☐ OAP☐ Dyspnée laryngée☐))

Réintubation pour chirurgie avec intubation <24h OUI☐ NON ☐

A la fin de J8 le patient est :

Intubé ☐ Extubé ☐

Poursuite du protocole de sevrage ☐

Sortie du protocole de sevrage ☐ (remplir la partie « sortie d'essai »)

Poids du jour : |__|__|__|kg

J9 = |__|__|/|__|__/|__|__| de 0h à 23h59

Remplir si patient intubé le matin de J9

- Critères préalables de sevrabilité : Présents (-> Faire TVS) ☐ Absents ☐
- TVS : Succès ☐ Echec ☐ (maintien intubation)
- Si succès :
  - Gaz du sang : pH |__|,|__|__|. pCO2 |__|__|__|mmHg pO2 |__|__|__|mmHg FiO2|__|__|__|%
  - Score de toux : 0 ☐ 1 ☐ 2 ☐ 3 ☐ 4 ☐ 5 ☐
  - Score d'encombrement : 0 ☐ 1 ☐ 2 ☐ 3 ☐ 4 ☐
  - Patients du groupe A (assisté) : TVS-TT Succès ☐ Echec ☐ (-> VNI prophylactique)
- Evaluer extubabilité si succès du TVS :
  - Force de toux acceptable (≥3) : Oui ☐ Non ☐
  - Encombrement acceptable (≤2) : Oui ☐ Non ☐
  - Absence d'AG dans les 24h qui suivent : Oui ☐ Non ☐
  - Pas d'argument pour une obstruction laryngée : Oui ☐ Non ☐

➔ Si ≥3 critères « oui » : extubation proposée :

➔ Heure d'extubation (hh :mm): |__|__|:|__|__|

Et raison de la non-extubation le cas échéant : ______________________________

Remplir si le patient n'est plus intubé à un moment de J9 (même quelques minutes ou depuis plusieurs jours)

Patient extubé AUJOURD'HUI OUI ☐ NON ☐

Sinon Date de la dernière extubation |__|__|/|__|__|/|__|__| Heure |__|__|:|__|__|

- Extubation (si réalisée AUJOURD'HUI)
  - programmée ☐ (selon protocole ☐ violation protocole ☐)
  - auto-extubation ☐
- VNI prophylactique post-extubation OUI ☐ NON ☐
    - *Si VNI prophylactique*
  - selon protocole ☐ (TVS-TT (-) ☐ âge>65ans ☐ BPCO ☐ Insuffisance cardiaque chronique ☐ Insuffisance respiratoire chronique ☐, PaCO2>45 en fin de TVS-TT ☐) violation protocole ☐
  - Durée cumulée sur les 24h : |__|__|h
    - *Si absence de VNI prophylactique*
  - selon protocole ☐ violation protocole ☐ contre-indication ☐
- VNI curative post-extubation NON ☐ selon protocole ☐ violation protocole ☐
- Optiflow post-extubation (non recommandé) OUI ☐ NON ☐
- Désencombrement bronchique : NON ☐ Toux manuellement assistée ☐ Toux assistée instrumentale ☐

Réintubation ce jour : OUI ☐ NON ☐ : si OUI Heure : |__|__|:|__|__|

(critère(s) Neurologique ☐ Hémodynamique ☐ autre ☐

Respiratoire ☐ (épuisement ☐ Encombrement ☐ Pneumopathie ☐ OAP ☐ Dyspnée laryngée ☐))

Réintubation pour chirurgie avec intubation <24h OUI ☐ NON ☐

A la fin de J9 le patient est :

Intubé ☐ Extubé ☐

Poursuite du protocole de sevrage ☐

Sortie du protocole de sevrage ☐ (remplir la partie « sortie d'essai »)

Poids du jour : |__|__|__| kg

J10 = |__|__|/|__|__/|__|__| de 0h à 23h59

Remplir si patient intubé le matin de J10

- Critères préalables de sevrabilité : Présents (-> Faire TVS) ☐ Absents ☐
- TVS : Succès ☐ Echec ☐ (maintien intubation)
- Si succès :
  - Gaz du sang : pH |__|,|__|__|. pCO2 |__|__|__|mmHg pO2 |__|__|__|mmHg FiO2|__|__|__|%
  - Score de toux : 0 ☐ 1 ☐ 2 ☐ 3 ☐ 4 ☐ 5 ☐
  - Score d'encombrement : 0 ☐ 1 ☐ 2 ☐ 3 ☐ 4 ☐
  - Patients du groupe A (assisté) : TVS-TT Succès ☐ Echec ☐ (-> VNI prophylactique)
- Evaluer extubabilité si succès du TVS :
  - Force de toux acceptable (≥3) : Oui ☐ Non ☐
  - Encombrement acceptable (≤2) : Oui ☐ Non ☐
  - Absence d'AG dans les 24h qui suivent : Oui ☐ Non ☐
  - Pas d'argument pour une obstruction laryngée : Oui ☐ Non ☐

➔ Si ≥3 critères « oui » : extubation proposée :

➔ Heure d'extubation (hh :mm): |__|__|:|__|__|

Et raison de la non-extubation le cas échéant : ______________________________

Remplir si le patient n'est plus intubé à un moment de J10 (même quelques minutes ou depuis plusieurs jours)

Patient extubé AUJOURD'HUI OUI ☐ NON ☐

Sinon Date de la dernière extubation |__|__|/|__|__|/|__|__| Heure |__|__|:|__|__|

- Extubation (si réalisée AUJOURD'HUI)
  - programmée ☐ (selon protocole ☐ violation protocole ☐)
  - auto-extubation ☐
- VNI prophylactique post-extubation OUI ☐ NON ☐
  - *Si VNI prophylactique*
  - selon protocole ☐ (TVS-TT (-) ☐ âge>65ans ☐ BPCO ☐ Insuffisance cardiaque chronique ☐ Insuffisance respiratoire chronique ☐, PaCO2>45 en fin de TVS-TT ☐) violation protocole ☐
  - Durée cumulée sur les 24h : |__|__|h
  - *Si absence de VNI prophylactique*
  - selon protocole ☐ violation protocole ☐ contre-indication ☐
- VNI curative post-extubation NON ☐ selon protocole ☐ violation protocole ☐
- Optiflow post-extubation (non recommandé) OUI ☐ NON ☐
- Désencombrement bronchique : NON ☐ Toux manuellement assistée ☐ Toux assistée instrumentale ☐

Réintubation ce jour : OUI ☐ NON ☐ : si OUI Heure : |__|__|:|__|__|
(critère(s) Neurologique ☐ Hémodynamique ☐ autre ☐
Respiratoire ☐ (épuisement ☐ Encombrement ☐ Pneumopathie ☐ OAP ☐ Dyspnée laryngée ☐))
Réintubation pour chirurgie avec intubation <24h OUI ☐ NON ☐

A la fin de J10 le patient est :
Intubé ☐ Extubé ☐
Poursuite du protocole de sevrage ☐
Sortie du protocole de sevrage ☐ (remplir la partie « sortie d'essai »)
Poids du jour : |__|__|__|kg

J11 = |__|__|/|__|__/|__|__| de 0h à 23h59

Remplir si patient intubé le matin de J11

- Critères préalables de sevrabilité : Présents (-> Faire TVS) ☐ Absents☐
- TVS : Succès ☐ Echec ☐ (maintien intubation)
- Si succès :
  - Gaz du sang : pH |__|,|__|__|. pCO2 |__|__|__|mmHg pO2 |__|__|__|mmHg FiO2|__|__|__|%
  - Score de toux : 0 ☐ 1 ☐ 2 ☐ 3 ☐ 4 ☐ 5 ☐
  - Score d'encombrement : 0 ☐ 1 ☐ 2 ☐ 3 ☐ 4 ☐
  - Patients du groupe A (assisté) : TVS-TT Succès ☐ Echec ☐ (-> VNI prophylactique)
- Evaluer extubabilité si succès du TVS :
  - Force de toux acceptable (≥3) : Oui ☐ Non ☐
  - Encombrement acceptable (≤2) : Oui ☐ Non ☐
  - Absence d'AG dans les 24h qui suivent : Oui ☐ Non ☐
  - Pas d'argument pour une obstruction laryngée : Oui ☐ Non ☐

➔ Si ≥3 critères « oui » : extubation proposée :

➔ Heure d'extubation (hh :mm): |__|__|:|__|__|

Et raison de la non-extubation le cas échéant : ____________________________

Remplir si le patient n'est plus intubé à un moment de J11 (même quelques minutes ou depuis plusieurs jours)

Patient extubé AUJOURD'HUI OUI ☐ NON ☐

Sinon Date de la dernière extubation |__|__|/|__|__|/|__|__| Heure |__|__|:|__|__|

- Extubation (si réalisée AUJOURD'HUI)
  - programmée☐ (selon protocole☐ violation protocole☐)
  - auto-extubation ☐
- VNI prophylactique post-extubation OUI ☐ NON ☐
  - *Si VNI prophylactique*
  - selon protocole ☐ (TVS-TT (-)☐ âge>65ans☐ BPCO☐ Insuffisance cardiaque chronique ☐ Insuffisance respiratoire chronique☐, PaCO2>45 en fin de TVS-TT☐) violation protocole ☐
  - Durée cumulée sur les 24h : |__|__|h
  - *Si absence de VNI prophylactique*
  - selon protocole ☐ violation protocole ☐ contre-indication ☐
- VNI curative post-extubation NON ☐ selon protocole ☐ violation protocole ☐
- Optiflow post-extubation (non recommandé) OUI ☐ NON ☐
- Désencombrement bronchique : NON ☐ Toux manuellement assistée ☐ Toux assistée instrumentale ☐

Réintubation ce jour : OUI ☐ NON ☐ : si OUI Heure : |__|__|:|__|__|

(critère(s) Neurologique☐ Hémodynamique ☐ autre ☐

Respiratoire ☐ (épuisement ☐ Encombrement ☐ Pneumopathie☐ OAP☐ Dyspnée laryngée☐))

Réintubation pour chirurgie avec intubation <24h OUI☐ NON ☐

A la fin de J11 le patient est :

Intubé ☐ Extubé ☐

Poursuite du protocole de sevrage ☐

Sortie du protocole de sevrage ☐ (remplir la partie « sortie d'essai »)

Poids du jour : |__|__|__|kg

J12 = |__|__|/|__|__/|__|__| de 0h à 23h59

Remplir si patient intubé le matin de J12

- Critères préalables de sevrabilité : Présents (-> Faire TVS) ☐ Absents☐
- TVS : Succès ☐ Echec ☐ (maintien intubation)
- Si succès :
  - Gaz du sang : pH |__|,|__|__|. pCO2 |__|__|__|mmHg pO2 |__|__|__|mmHg FiO2|__|__|__|%
  - Score de toux : 0 ☐ 1 ☐ 2 ☐ 3 ☐ 4 ☐ 5 ☐
  - Score d'encombrement : 0 ☐ 1 ☐ 2 ☐ 3 ☐ 4 ☐
  - Patients du groupe A (assisté) : TVS-TT Succès ☐ Echec ☐ (-> VNI prophylactique)
- Evaluer extubabilité si succès du TVS :
  - Force de toux acceptable (≥3) : Oui ☐ Non ☐
  - Encombrement acceptable (≤2) : Oui ☐ Non ☐
  - Absence d'AG dans les 24h qui suivent : Oui ☐ Non ☐
  - Pas d'argument pour une obstruction laryngée : Oui ☐ Non ☐

➔ Si ≥3 critères « oui » : extubation proposée :

➔ Heure d'extubation (hh :mm): |__|__|:|__|__|

Et raison de la non-extubation le cas échéant : ______________________________

Remplir si le patient n'est plus intubé à un moment de J12 (même quelques minutes ou depuis plusieurs jours)

Patient extubé AUJOURD'HUI OUI ☐ NON ☐

Sinon Date de la dernière extubation |__|__|/|__|__|/|__|__| Heure |__|__|:|__|__|

- Extubation (si réalisée AUJOURD'HUI)
  - programmée☐ (selon protocole☐ violation protocole☐)
  - auto-extubation ☐
- VNI prophylactique post-extubation OUI ☐ NON ☐
  - *Si VNI prophylactique*
  - selon protocole ☐ (TVS-TT (-)☐ âge>65ans☐ BPCO☐ Insuffisance cardiaque chronique ☐ Insuffisance respiratoire chronique☐, PaCO2>45 en fin de TVS-TT☐) violation protocole ☐
  - Durée cumulée sur les 24h : |__|__|h
  - *Si absence de VNI prophylactique*
  - selon protocole ☐ violation protocole ☐ contre-indication ☐
- VNI curative post-extubation NON ☐ selon protocole ☐ violation protocole ☐
- Optiflow post-extubation (non recommandé) OUI ☐ NON ☐
- Désencombrement bronchique : NON ☐ Toux manuellement assistée ☐
  Toux assistée instrumentale ☐

Réintubation ce jour : OUI ☐ NON ☐ : si OUI Heure : |__|__|:|__|__|

(critère(s) Neurologique☐ Hémodynamique ☐ autre ☐

Respiratoire ☐ (épuisement ☐ Encombrement ☐ Pneumopathie☐ OAP☐ Dyspnée laryngée☐))

Réintubation pour chirurgie avec intubation <24h OUI☐ NON ☐

A la fin de J12 le patient est :

Intubé ☐ Extubé ☐

Poursuite du protocole de sevrage ☐

Sortie du protocole de sevrage ☐ (remplir la partie « sortie d'essai »)

Poids du jour : |__|__|__|kg

DEMANDER CRF ADDITIONNEL !!!!!!!!!!!!!!!

J13 = |__|__|/|__|__/|__|__| de 0h à 23h59

<u>Remplir si patient intubé le matin de J13</u>

- Critères préalables de sevrabilité : Présents (-> Faire TVS) ☐ Absents☐
- TVS : Succès ☐ Echec ☐ (maintien intubation)
- Si succès :
  - Gaz du sang : pH |__|,|__|__|. pCO_2 |__|__|__|mmHg pO_2 |__|__|__|mmHg FiO_2|__|__|__|%
  - Score de toux : 0 ☐ 1 ☐ 2 ☐ 3 ☐ 4 ☐ 5 ☐
  - Score d'encombrement : 0 ☐ 1 ☐ 2 ☐ 3 ☐ 4 ☐
  - Patients du <u>groupe A</u> (assisté) : TVS-TT Succès ☐ Echec ☐ (-> VNI prophylactique)
- Evaluer extubabilité si succès du TVS :
  - Force de toux acceptable (≥3) : Oui ☐ Non ☐
  - Encombrement acceptable (≤2) : Oui ☐ Non ☐
  - Absence d'AG dans les 24h qui suivent : Oui ☐ Non ☐
  - Pas d'argument pour une obstruction laryngée : Oui ☐ Non ☐

➔ Si ≥3 critères « oui » : extubation proposée :

➔ Heure d'extubation (hh :mm): |__|__|:|__|__|

Et raison de la non-extubation le cas échéant : ______________________________

<u>Remplir si le patient n'est plus intubé à un moment de J13 (même quelques minutes ou depuis plusieurs jours)</u>

Patient extubé AUJOURD'HUI OUI ☐ NON ☐

Sinon Date de la dernière extubation |__|__|/|__|__|/|__|__| Heure |__|__|:|__|__|

- Extubation (<u>si réalisée AUJOURD'HUI</u>)
  - programmée☐ (selon protocole☐ violation protocole☐)
  - auto-extubation ☐
- VNI <u>prophylactique</u> post-extubation OUI ☐ NON ☐
  - ▪ *Si VNI prophylactique*
  - <u>selon protocole</u> ☐ (TVS-TT (-)☐ âge>65ans☐ BPCO☐ Insuffisance cardiaque chronique ☐ Insuffisance respiratoire chronique☐, $PaCO_2$>45 en fin de TVS-TT☐) <u>violation protocole</u> ☐
  - Durée cumulée sur les 24h : |__|__|h
  - ▪ *Si absence de VNI prophylactique*
  - selon protocole ☐ violation protocole ☐ contre-indication ☐
- VNI <u>curative</u> post-extubation NON ☐ selon protocole ☐ violation protocole ☐
- Optiflow post-extubation (non recommandé) OUI ☐ NON ☐
- Désencombrement bronchique : NON ☐ Toux manuellement assistée ☐ Toux assistée instrumentale ☐

Réintubation ce jour : OUI☐ NON ☐ : si OUI Heure : |__|__|:|__|__|
(critère(s) Neurologique☐ Hémodynamique ☐ autre ☐
Respiratoire ☐ (épuisement ☐ Encombrement ☐ Pneumopathie☐ OAP☐ Dyspnée laryngée☐))
Réintubation pour chirurgie avec intubation <24h OUI☐ NON ☐

A la fin de J13 le patient est :
Intubé ☐ Extubé ☐
Poursuite du protocole de sevrage ☐
Sortie du protocole de sevrage ☐ (remplir la partie « sortie d'essai »)
Poids du jour : |__|__|__|kg

<u>DEMANDER CRF ADDITIONNEL !!!!!!!!!!!!!!!</u>

J14 = |__|__|/|__|__/|__|__| de 0h à 23h59

Remplir si patient intubé le matin de J14

- Critères préalables de sevrabilité : Présents (-> Faire TVS) ☐ Absents☐
- TVS : Succès ☐ Echec ☐ (maintien intubation)
- Si succès :
  - Gaz du sang : pH |__|,|__|__|. pCO2 |__|__|__|mmHg pO2 |__|__|__|mmHg FiO2|__|__|__|%
  - Score de toux : 0 ☐ 1 ☐ 2 ☐ 3 ☐ 4 ☐ 5 ☐
  - Score d'encombrement : 0 ☐ 1 ☐ 2 ☐ 3 ☐ 4 ☐
  - Patients du groupe A (assisté) : TVS-TT Succès ☐ Echec ☐ (-> VNI prophylactique)
- Evaluer extubabilité si succès du TVS :
  - Force de toux acceptable (≥3) : Oui ☐ Non ☐
  - Encombrement acceptable (≤2) : Oui ☐ Non ☐
  - Absence d'AG dans les 24h qui suivent : Oui ☐ Non ☐
  - Pas d'argument pour une obstruction laryngée : Oui ☐ Non ☐

➔ Si ≥3 critères « oui » : extubation proposée :

➔ Heure d'extubation (hh :mm): |__|__|:|__|__|

Et raison de la non-extubation le cas échéant : ______________________________

Remplir si le patient n'est plus intubé à un moment de J14 (même quelques minutes ou depuis plusieurs jours)

Patient extubé AUJOURD'HUI OUI ☐ NON ☐

Sinon Date de la dernière extubation |__|__|/|__|__|/|__|__| Heure |__|__|:|__|__|

- Extubation (si réalisée AUJOURD'HUI)
  - programmée☐ (selon protocole☐ violation protocole☐)
  - auto-extubation ☐
- VNI prophylactique post-extubation OUI ☐ NON ☐
  - *Si VNI prophylactique*
  - selon protocole ☐ (TVS-TT (-)☐ âge>65ans☐ BPCO☐ Insuffisance cardiaque chronique ☐ Insuffisance respiratoire chronique☐, PaCO2>45 en fin de TVS-TT☐) violation protocole ☐
  - Durée cumulée sur les 24h : |__|__|h
  - *Si absence de VNI prophylactique*
  - selon protocole ☐ violation protocole ☐ contre-indication ☐
- VNI curative post-extubation NON ☐ selon protocole ☐ violation protocole ☐
- Optiflow post-extubation (non recommandé) OUI ☐ NON ☐
- Désencombrement bronchique : NON ☐ Toux manuellement assistée ☐ Toux assistée instrumentale ☐

Réintubation ce jour : OUI ☐ NON ☐ : si OUI Heure : |__|__|:|__|__|

(critère(s) Neurologique☐ Hémodynamique ☐ autre ☐

Respiratoire ☐ (épuisement ☐ Encombrement ☐ Pneumopathie☐ OAP☐ Dyspnée laryngée☐))

Réintubation pour chirurgie avec intubation <24h OUI☐ NON ☐

A la fin de J14 le patient est :

Intubé ☐ Extubé ☐

Poursuite du protocole de sevrage ☐

Sortie du protocole de sevrage ☐ (remplir la partie « sortie d'essai »)

Poids du jour : |__|__|__|kg

DEMANDER CRF ADDITIONNEL !!!!!!!!!!!!!!!!

J15 = |__|__|/|__|__/|__|__| de 0h à 23h59

Remplir si patient intubé le matin de J15

- Critères préalables de sevrabilité : Présents (-> Faire TVS) ☐ Absents ☐
- TVS : Succès ☐ Echec ☐ (maintien intubation)
- Si succès :
  - Gaz du sang : pH |__|,|__|__|. pCO2 |__|__|__|mmHg pO2 |__|__|__|mmHg FiO2|__|__|__|%
  - Score de toux : 0 ☐ 1 ☐ 2 ☐ 3 ☐ 4 ☐ 5 ☐
  - Score d'encombrement : 0 ☐ 1 ☐ 2 ☐ 3 ☐ 4 ☐
  - Patients du groupe A (assisté) : TVS-TT Succès ☐ Echec ☐ (-> VNI prophylactique)
- Evaluer extubabilité si succès du TVS :
  - Force de toux acceptable (≥3) : Oui ☐ Non ☐
  - Encombrement acceptable (≤2) : Oui ☐ Non ☐
  - Absence d'AG dans les 24h qui suivent : Oui ☐ Non ☐
  - Pas d'argument pour une obstruction laryngée : Oui ☐ Non ☐

➔ Si ≥3 critères « oui » : extubation proposée :

➔ Heure d'extubation (hh :mm): |__|__|:|__|__|

Et raison de la non-extubation le cas échéant : ______________________________

Remplir si le patient n'est plus intubé à un moment de J15 (même quelques minutes ou depuis plusieurs jours)

Patient extubé AUJOURD'HUI OUI ☐ NON ☐

Sinon Date de la dernière extubation |__|__|/|__|__|/|__|__| Heure |__|__|:|__|__|

- Extubation (si réalisée AUJOURD'HUI)
  - programmée ☐ (selon protocole ☐ violation protocole ☐)
  - auto-extubation ☐
- VNI prophylactique post-extubation OUI ☐ NON ☐
  - *Si VNI prophylactique*
  - selon protocole ☐ (TVS-TT (-) ☐ âge>65ans ☐ BPCO ☐ Insuffisance cardiaque chronique ☐ Insuffisance respiratoire chronique ☐, PaCO2>45 en fin de TVS-TT ☐) violation protocole ☐
  - Durée cumulée sur les 24h : |__|__|h
  - *Si absence de VNI prophylactique*
  - selon protocole ☐ violation protocole ☐ contre-indication ☐
- VNI curative post-extubation NON ☐ selon protocole ☐ violation protocole ☐
- Optiflow post-extubation (non recommandé) OUI ☐ NON ☐
- Désencombrement bronchique : NON ☐ Toux manuellement assistée ☐
  Toux assistée instrumentale ☐

Réintubation ce jour : OUI ☐ NON ☐ : si OUI Heure : |__|__|:|__|__|

(critère(s) Neurologique ☐ Hémodynamique ☐ autre ☐

Respiratoire ☐ (épuisement ☐ Encombrement ☐ Pneumopathie ☐ OAP ☐ Dyspnée laryngée ☐))

Réintubation pour chirurgie avec intubation <24h OUI ☐ NON ☐

A la fin de J15 le patient est :

Intubé ☐ Extubé ☐

Poursuite du protocole de sevrage ☐

Sortie du protocole de sevrage ☐ (remplir la partie « sortie d'essai »)

Poids du jour : |__|__|__|kg

CONTINUER SUR CRF ADDITIONNEL !!!!!!!!!!!!!!!

TRACHEOTOMIE

Patient trachéotomisé lors du séjour en réanimation : Oui ☐ Non ☐

Date de la trachéotomie |__|__|/|__|__|/|__|__|

Heure |__|__|:|__|__|

Méthode : Percutanée ☐ Chirurgicale ☐

Indication (s) :

- Sevrage respiratoire jugé difficile ☐
- Troubles de la déglutition/encombrement ☐
- Autre (détailler) ☐ ______________________________

Patient déventilé avec succès :

- Oui ☐ Date de la trachéotomie |__|__|/|__|__|/|__|__|

Heure |__|__|:|__|__|

- Non ☐

SORTIE D'ESSAI

Sortie d'essai : Prématurée ☐ Fin du protocole ☐

Date de sortie : |__|__| |__|__| |__|__|

Motif de sortie prématurée :

- Transfert dans une autre réanimation ne participant pas à l'étude .... ☐
- Évènement Indésirable Grave sur décision du clinicien .... ☐
- Retrait du consentement .... ☐
- Autres, détailler : .... ☐

Fin de la stratégie de sevrage :

- Absence de réintubation dans les sept jours suivant l'extubation .... ☐
- Arrêt des thérapeutiques actives ou limitation sur une ré-intubation ... ☐
- Sortie vivant de réanimation .... ☐
- Décès .... ☐
- Patient toujours intubé à J90.. ☐
- Autres, détailler : .... ☐

EVENEMENTS INDESIRABLES

Survenue d'un évènement indésirable grave

Non ☐ Oui ☐ → remplir dernière page du CRF

MORTALITE et STATUT RESPIRATOIRE

Statut à la sortie de réanimation :
Vivant ☐ Mort ☐ Perdu de vue ☐

Statut à J28 (comptabilisé depuis l'inclusion)
Vivant ☐ Mort ☐ Perdu de vue ☐

Statut à J90 (comptabilisé depuis l'inclusion)
Vivant ☐ Mort ☐ Perdu de vue ☐

Statut respiratoire à la sortie de réanimation :
Non ventilé ☐ Ventilation non invasive (sauf CPAP) ☐
Ventilation invasive (trachéotomie) ☐ Trachéotomie (sans ventilation) ☐
Si VNI réalisée en post-extubation puis arrêtée : date et heure de fin :
|__|__|/|__|__|/|__|__| |__|__| :|__|__|

Statut respiratoire à J28 (comptabilisé depuis l'inclusion)
Non ventilé ☐ Ventilation non invasive (sauf CPAP) ☐
Ventilation invasive (trachéotomie) ☐ Trachéotomie (sans ventilation) ☐
Perdu de vue ☐

Statut respiratoire à J90 (comptabilisé depuis l'inclusion)
Non ventilé ☐ Ventilation non invasive (sauf CPAP) ☐
Ventilation invasive (trachéotomie) ☐ Trachéotomie (sans ventilation) ☐
Perdu de vue ☐

Date de sortie de réanimation : |__|__|/|__|__|/|__|__|

Date de sortie de l'hôpital (domicile ou SSR ou EHPAD) : |__|__|/|__|__|/|__|__|

Extubation avec succès : Oui ☐ Décès ☐ Echec sevrage ☐

Rang extubation avec succès : |__|__|

Date/heure extubation avec succès : |__|__|/|__|__|/|__|__| |__|__| :|__|__|

Durée VM invasive : |__|__|__|__|h

Durée VM totale : |__|__|__|__|h

Nombre jours VM invasive à J28 (28 si décès) : |__|__|j

Nombre jours VM invasive à J90 (90 si décès) : |__|__|j

Nombre réintubation : |__|__|

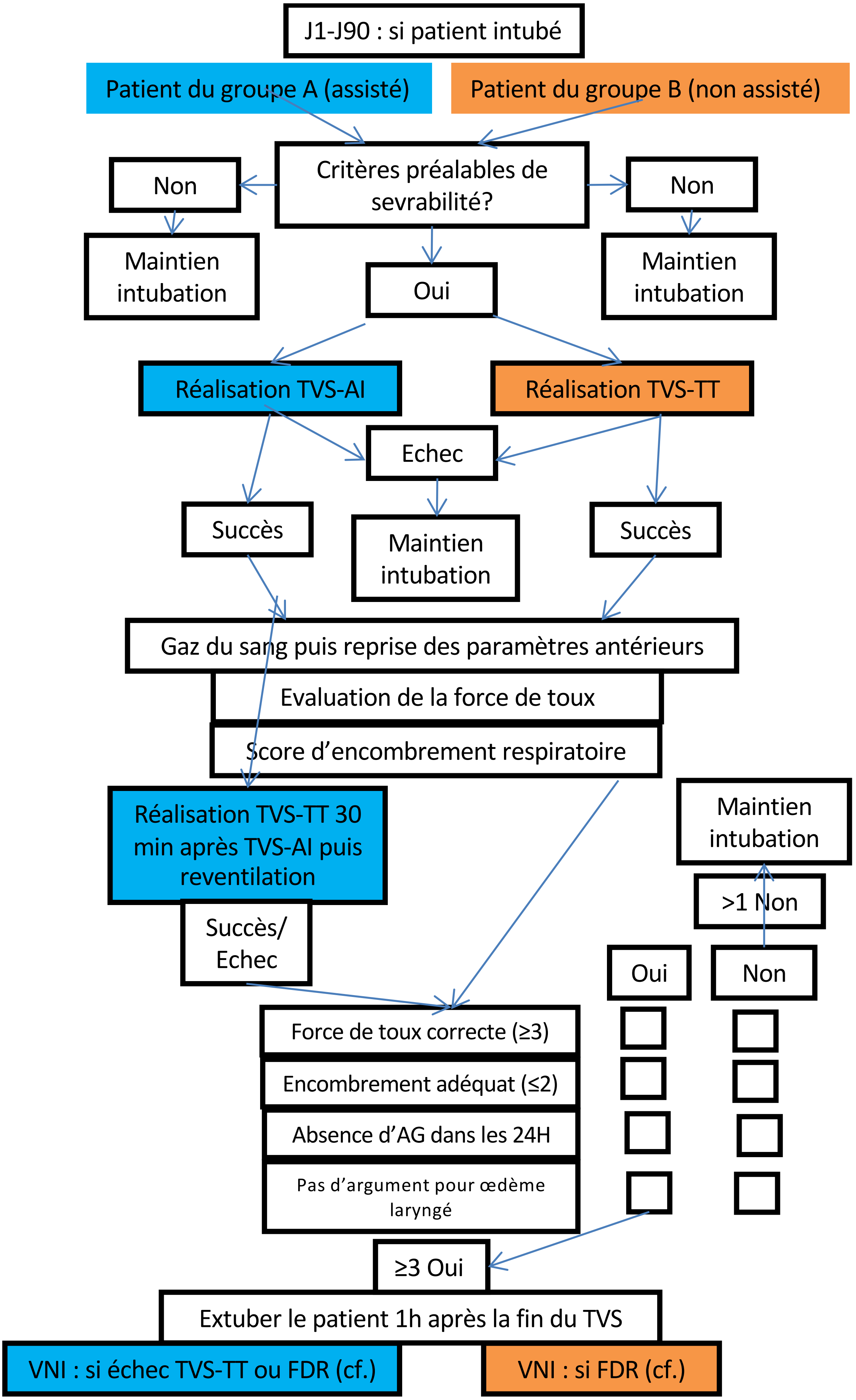
J1-J90 : si patient intubé
Patient du groupe A (assisté)
Patient du groupe B (non assisté)
Critères préalables de sevrabilité?
Non
Non
Maintien intubation
Maintien intubation
Oui
Réalisation TVS-AI
Réalisation TVS-TT
Echec
Succès
Maintien intubation
Succès
Gaz du sang puis reprise des paramètres antérieurs
Evaluation de la force de toux
Score d'encombrement respiratoire
Réalisation TVS-TT 30 min après TVS-AI puis reventilation
Maintien intubation
>1 Non
Succès/ Echec
Oui
Non
Force de toux correcte (≥3)
Encombrement adéquat (≤2)
Absence d'AG dans les 24H
Pas d'argument pour œdème laryngé
≥3 Oui
Extuber le patient 1h après la fin du TVS
VNI : si échec TVS-TT ou FDR (cf.)
VNI : si FDR (cf.)

CRITERES PREALABLES DE SEVRABILITE

- Neurologiques : réponse aux ordres simples
- Respiratoires : $FiO_2 \leq 50$ %, $SpO_2 \geq 88$ %, PEP≤5 cmH_2O, fréquence respiratoire≤35/min
- Hémodynamiques : noradrénaline<1 mg/h, dobutamine≤5 µg/kg/min

Critères et modalités de l'étude

Tests de ventilation spontanée (TVS)

TVS-AI

Respirateur réglé en VSAI
AI : 7 cmH_2O PEP : 5 cmH_2O
Pente de pressurisation : 200 ms
Trigger expiratoire libre (25 % recommandé)
Trigger inspiratoire : 2 – 5 l/min
$FiO_2 \leq 50$ %
Suppression de la ventilation d'apnée
Durée 30 minutes

TVS-TT

Patient débranché du respirateur
Sonde d'intubation raccordée à une pièce en T
Administration possible d'O_2 pur à un débit permettant d'obtenir une SpO_2 [94–98]% (sauf BPCO, SpO_2 [88–92]%).
Débit maximal d'oxygène 10L/min.
Durée 30 minutes

Critères d'échec des TVS

Variation > 20% de la fréquence cardiaque,
Variation > 20% de la pression artérielle systolique,
FR > 35/min,
SpO_2 < 88%
Sueurs, agitation,
Troubles de conscience,
Signes de détresse respiratoire autres: tirage sus-claviculaire, tirage inter-costal, contraction abdominale à l'expiration, balancement thoraco-abdominal.
pH<7.35 et PCO_2>45mmHg

Evaluation de la toux (après succès de TVS)

0 pas de toux
1 mouvement d'air audible dans la sonde d'intubation sans toux audible
2 toux audible très faible
3 toux clairement audible
4 toux forte
5 plusieurs toux fortes.
Ce score est évalué en demandant au patient de tousser le plus fort possible après déconnexion du respirateur de la sonde d'intubation.

Evaluation de l'encombrement bronchique

0 (absent)
1 (faible quantité)
2 (intermédiaire)
3 (abondant)
4 (très abondant)

Critères et modalités de l'étude

VNI post-extubation

Masque naso-buccal relié au respirateur de réanimation, en mode VSAI.
AI : entre 5 et 15 cmH2O avec pour objectif un volume courant expiré de 6-8mL/kg de poids prédit par la taille (Homme : 50 + 0,9 × [Taille (cm) − 152,4] ; Femme : 45,5 + 0,9 × [Taille (cm) − 152,4])
PEP : entre 5 et 10 cmH2O
AI+PEP≤20 cmH2O, idéalement moins de 15 cmH2O
Pente : 200 ms
Trigger expiratoire libre (25 % par défaut)
Trigger inspiratoire : 2 – 5 l/min à adapter secondairement en fonction des auto-déclenchements éventuels
FiO2 QSP SpO2 94-98 % (sauf BPCO : 88-92 %)
Temps inspiratoire maximal 1.5 sec
Séances 1 h à 2 h/3 h, avec un minimum de 8 h par jour de durée cumulée. La nuit, si le patient s'endort avec la VNI, il est possible de la laisser en place en continu.
Entre les séances de VNI, si besoin, support par oxygène conventionnel (pas d'oxygène haut débit), QSP SpO2 94-98 % (sauf BPCO : 88-92 %).La VNI est appliquée pendant une durée de 24 h au minimum, sa poursuite est laissée au choix du clinicien en charge du patient.

Critères de VNI post-extubation

Groupe A (assisté) :

- Si TVS-TT négatif
- Si âge>65 ans, insuffisance respiratoire ou cardiaque chronique, PaCO2>45 mmHg en fin de TVS-TT, BPCO

Groupe B (non assisté)

- Si âge>65 ans, insuffisance respiratoire ou cardiaque chronique, PaCO2>45 mmHg en fin de TVS-TT, BPCO

Oxygénothérapie post-extubation

Support par oxygène conventionnel (pas d'oxygène haut débit), QSP SpO2 94-98 % (sauf BPCO : 88-92 %).

Critères de réintubation

•Critère respiratoire
Il est fait le diagnostic de détresse respiratoire devant deux ou plus des éléments suivants :

- Fréquence respiratoire > 40/min
- Signes de lutte
- Encombrement respiratoire
- pH<7,35
- SpO2<90 % ou PaO2<60 mmHg sous FiO2 60 % (en VNI) ou 10 l O2 au masque haute concentration.

Si le patient n'est pas sous VNI à ce moment (et quel que soit son groupe et indication de VNI préalable), le recours à la VNI de sauvetage n'est autorisé que s'il existe une suspicion d'OAP post-extubation ou chez les patients BPCO/hypercapniques.
Le recours à une diurétisation (OAP) et aérosols de béta-mimétiques (BPCO) sont encouragés ainsi que le recours à la morphine à visée anti-dyspnée.
Dans les autres cas, le recours à la VNI est déconseillé (2,5) sauf à visée de pré-oxygénation avant intubation.
En cas d'encombrement, il est possible de recourir à des méthodes invasives et non invasives de désencombrement.
En cas de dyspnée laryngée, il est possible de recourir des traitements par nébulisation ou parentéraux à visée anti-œdémateuse.
La persistance des critères de détresse respiratoire persistant au maximum après 1 h de prise en charge implique une réintubation en urgence. Le diagnostic étiologique de la détresse respiratoire est noté dans le CRF ainsi que la date et l'heure de la réintubation.
•Critères hémodynamiques
Le recours à des doses majeures de noradrénaline (>1 µg/kg/min), une lactacidémie non contrôlée doivent faire discuter par le clinicien le recours à l'intubation.
•Critères neurologiques
Des troubles de conscience (définis par un score de Glasgow <8) non rapidement réversibles doivent faire envisager une intubation (hormis le cas d'une hypercapnie importante, pouvant justifier, le recours à une VNI).

Score de Charlson

1 point	50-60 ans	❑ OUI ❑ NON
	Infarctus myocardique	❑ OUI ❑ NON
	Insuffisance cardiaque	❑ OUI ❑ NON
	Insuffisance vasculaire périphérique	❑ OUI ❑ NON
	Maladie cérébrovasculaire	❑ OUI ❑ NON
	Démence	❑ OUI ❑ NON
	Maladie pulmonaire chronique	❑ OUI ❑ NON
	Maladie du tissu conjonctif	❑ OUI ❑ NON
	Maladie ulcéreuses	❑ OUI ❑ NON
	Hépatopathie	❑ OUI ❑ NON
	Diabète	❑ OUI ❑ NON
2 points	61-70 ans	❑ OUI ❑ NON
	Hémiplégie	❑ OUI ❑ NON
	Maladie rénale modérée à sévère	❑ OUI ❑ NON
	Diabète avec lésion organique	❑ OUI ❑ NON
	Tumeurs de toute origine	❑ OUI ❑ NON
3 points	71-80 ans	
	Hépatopathie modérée à sévère	❑ OUI ❑ NON
4 points	81-90 ans	
5 points	Plus de 90 ans	
6 points	Tumeur solide métastatique	❑ OUI ❑ NON
	SIDA	❑ OUI ❑ NON

SIGNATURE

Cette page doit être signée par l'investigateur principal du centre

Je soussigné, ____ (titre, prénom, nom)

Certifie que les données recueillies dans ce cahier d'observation sont exactes et que les corrections ont été faites conformément aux données du dossier médical.

Fait à
Le :

Signature

Initiales patient : |__|__|__| N° patient : |__|__|__| N° centre : |__|__|__|

1ère lettre du prénom - 1ère lettre du nom

**PATIENT**	INITIALES : |__|__|__| INCLUSION N°: ____________

**Evénement** (un seul événement par ligne)	**Date de début** (JJ/MM/AA)	**Date de fin** (JJ/MM/AA)	**Relié à l'étude**		**Intensité[a]**	**Evolution[b]**	**Mesures prises[c]**
			**Oui**	**Non***			
	|__|__| |__|__| |__|__|	|__|__| |__|__| |__|__|	☐	|__|	|__|	|__|	|__|
	|__|__| |__|__| |__|__|	|__|__| |__|__| |__|__|	☐	|__|	|__|	|__|	|__|
	|__|__| |__|__| |__|__|	|__|__| |__|__| |__|__|	☐	|__|	|__|	|__|	|__|
	|__|__| |__|__| |__|__|	|__|__| |__|__| |__|__|	☐	|__|	|__|	|__|	|__|
	|__|__| |__|__| |__|__|	|__|__| |__|__| |__|__|	☐	|__|	|__|	|__|	|__|
	|__|__| |__|__| |__|__|	|__|__| |__|__| |__|__|	☐	|__|	|__|	|__|	|__|
	|__|__| |__|__| |__|__|	|__|__| |__|__| |__|__|	☐	|__|	|__|	|__|	|__|
	|__|__| |__|__| |__|__|	|__|__| |__|__| |__|__|	☐	|__|	|__|	|__|	|__|
