# Supplementary material for: Pressure support and positive end-expiratory pressure versus T-piece during spontaneous breathing trial in difficult weaning from mechanical ventilation: study protocol for the SBT-ICU study
Source: Trials. 2022 Dec 12;23:993. doi: 10.1186/s13063-022-06896-4 (PMC9742015; doi:10.1186/s13063-022-06896-4)
Supplement: Supplementary file 7 — Additional file 7. [file 13063_2022_6896_MOESM7_ESM.pdf]

IMPACT DE LA COMBINAISON DE L'AIDE INSPIRATOIRE ET DE LA PRESSION EXPIRATOIRE POSITIVE PENDANT L'EPREUVE DE SEVRAGE RESPIRATOIRE EN COMPARAISON DE LA PIECE EN T SUR LE DELAI JUSQU'A L'EXTUBATION AVEC SUCCES

SBT-ICU

CAHIER D'OBSERVATION additionnel J16-J90

INITIALES DU PATIENT (*): |__|__|__|

(*) 1ère lettre PRENOM -Tiret -1ère lettre NOM
Exemple : Jean Dupont : J-D
Jean-Paul Dupont : JPD

N° PATIENT |__|__|__|
RANDOMISATION :
Bras A (stratégie assistée) ☐
Bras B (stratégie non assistée) ☐

STRATIFICATION
BPCO (+/- insuffisance cardiaque chronique) ☐
Insuffisance cardiaque chronique sans BPCO ☐
Pas de BPCO et pas d'insuffisance cardiaque chronique ☐

Investigateur Coordonnateur :
Dr Mehdi Mezidi
Service de réanimation médicale
Hôpital de la Croix-Rousse
103 Grande rue de la Croix Rousse
69004 LYON
Tél : 04 26 10 92 75 ; Fax : 04 72 07 17 74
Email : mehdi.mezidi@chu-lyon.fr

Promoteur de l'étude : Hospices Civils de Lyon
Direction de la Recherche Clinique et de l'Innovation
3, quai des Célestins
BP 2251
69229 Lyon Cedex 02
www.chu-lyon.fr

J16 = |__|__|/|__|__/|__|__| de 0h à 23h59

Remplir si patient intubé le matin de J16

- Critères préalables de sevrabilité : Présents (-> Faire TVS) ☐ Absents☐
- TVS : Succès ☐ Echec ☐ (maintien intubation)
- Si succès :
  - Gaz du sang : pH |__|,|__|__|. pCO2 |__|__|__|mmHg pO2 |__|__|__|mmHg FiO2|__|__|__|%
  - Score de toux : 0 ☐ 1 ☐ 2 ☐ 3 ☐ 4 ☐ 5 ☐
  - Score d'encombrement : 0 ☐ 1 ☐ 2 ☐ 3 ☐ 4 ☐
  - Patients du groupe A (assisté) : TVS-TT Succès ☐ Echec ☐ (-> VNI prophylactique)
- Evaluer extubabilité si succès du TVS :
  - Force de toux acceptable (≥3) : Oui ☐ Non ☐
  - Encombrement acceptable (≤2) : Oui ☐ Non ☐
  - Absence d'AG dans les 24h qui suivent : Oui ☐ Non ☐
  - Pas d'argument pour une obstruction laryngée : Oui ☐ Non ☐

➔ Si ≥3 critères « oui » : extubation proposée :

➔ Heure d'extubation (hh :mm): |__|__|:|__|__|

Et raison de la non-extubation le cas échéant : ____________________________

Remplir si le patient n'est plus intubé à un moment de J16 (même quelques minutes ou depuis plusieurs jours)

Patient extubé AUJOURD'HUI OUI ☐ NON ☐

Sinon Date de la dernière extubation |__|__|/|__|__|/|__|__| Heure |__|__|:|__|__|

- Extubation (si réalisée AUJOURD'HUI)
  - programmée☐ (selon protocole☐ violation protocole☐)
  - auto-extubation ☐
- VNI prophylactique post-extubation OUI ☐ NON ☐
  - *Si VNI prophylactique*
  - selon protocole ☐ (TVS-TT (-)☐ âge>65ans☐ BPCO☐ Insuffisance cardiaque chronique ☐ Insuffisance respiratoire chronique☐, PaCO2>45 en fin de TVS-TT☐) violation protocole ☐
  - Durée cumulée sur les 24h : |__|__|h
  - *Si absence de VNI prophylactique*
  - selon protocole ☐ violation protocole ☐ contre-indication ☐
- VNI curative post-extubation NON ☐ selon protocole ☐ violation protocole ☐
- Optiflow post-extubation (non recommandé) OUI ☐ NON ☐
- Désencombrement bronchique : NON ☐ Toux manuellement assistée ☐
  Toux assistée instrumentale ☐

Réintubation ce jour : OUI ☐ NON ☐ : si OUI Heure : |__|__|:|__|__|
(critère(s) Neurologique☐ Hémodynamique ☐ autre ☐
Respiratoire ☐ (épuisement ☐ Encombrement ☐ Pneumopathie☐ OAP☐ Dyspnée laryngée☐))
Réintubation pour chirurgie avec intubation <24h OUI☐ NON ☐

A la fin de J16 le patient est :
Intubé ☐ Extubé ☐
Poursuite du protocole de sevrage ☐
Sortie du protocole de sevrage ☐ (remplir la partie « sortie d'essai »)
Poids du jour : |__|__|__| kg

J17 = |__|__|/|__|__/|__|__| de 0h à 23h59

Remplir si patient intubé le matin de J17

- Critères préalables de sevrabilité : Présents (-> Faire TVS) ☐ Absents☐
- TVS : Succès ☐ Echec ☐ (maintien intubation)
- Si succès :
  - Gaz du sang : pH |__|,|__|__|. pCO2 |__|__|__|mmHg pO2 |__|__|__|mmHg FiO2|__|__|__|%
  - Score de toux : 0 ☐ 1 ☐ 2 ☐ 3 ☐ 4 ☐ 5 ☐
  - Score d'encombrement : 0 ☐ 1 ☐ 2 ☐ 3 ☐ 4 ☐
  - Patients du groupe A (assisté) : TVS-TT Succès ☐ Echec ☐ (-> VNI prophylactique)
- Evaluer extubabilité si succès du TVS :
  - Force de toux acceptable (≥3) : Oui ☐ Non ☐
  - Encombrement acceptable (≤2) : Oui ☐ Non ☐
  - Absence d'AG dans les 24h qui suivent : Oui ☐ Non ☐
  - Pas d'argument pour une obstruction laryngée : Oui ☐ Non ☐

➔ Si ≥3 critères « oui » : extubation proposée :

➔ Heure d'extubation (hh :mm): |__|__|:|__|__|

Et raison de la non-extubation le cas échéant : ____________________________

Remplir si le patient n'est plus intubé à un moment de J17 (même quelques minutes ou depuis plusieurs jours)

Patient extubé AUJOURD'HUI OUI ☐ NON ☐

Sinon Date de la dernière extubation |__|__|/|__|__|/|__|__| Heure |__|__|:|__|__|

- Extubation (si réalisée AUJOURD'HUI)
  - programmée☐ (selon protocole☐ violation protocole☐)
  - auto-extubation ☐
- VNI prophylactique post-extubation OUI ☐ NON ☐
  - *Si VNI prophylactique*
  - selon protocole ☐ (TVS-TT (-)☐ âge>65ans☐ BPCO☐ Insuffisance cardiaque chronique ☐ Insuffisance respiratoire chronique☐, PaCO2>45 en fin de TVS-TT☐) violation protocole ☐
  - Durée cumulée sur les 24h : |__|__|h
  - *Si absence de VNI prophylactique*
  - selon protocole ☐ violation protocole ☐ contre-indication ☐
- VNI curative post-extubation NON ☐ selon protocole ☐ violation protocole ☐
- Optiflow post-extubation (non recommandé) OUI ☐ NON ☐
- Désencombrement bronchique : NON ☐ Toux manuellement assistée ☐
  Toux assistée instrumentale ☐

Réintubation ce jour : OUI ☐ NON ☐ : si OUI Heure : |__|__|:|__|__|

(critère(s) Neurologique☐ Hémodynamique ☐ autre ☐

Respiratoire ☐ (épuisement ☐ Encombrement ☐ Pneumopathie☐ OAP☐ Dyspnée laryngée☐))

Réintubation pour chirurgie avec intubation <24h OUI☐ NON ☐

A la fin de J17 le patient est :

Intubé ☐ Extubé ☐

Poursuite du protocole de sevrage ☐

Sortie du protocole de sevrage ☐ (remplir la partie « sortie d'essai »)

Poids du jour : |__|__|__| kg

J18 = |__|__|/|__|__/|__|__| de 0h à 23h59

Remplir si patient intubé le matin de J18

- Critères préalables de sevrabilité : Présents (-> Faire TVS) ☐ Absents☐
- TVS : Succès ☐ Echec ☐ (maintien intubation)
- Si succès :
  - Gaz du sang : pH |__|,|__|__|. pCO2 |__|__|__|mmHg pO2 |__|__|__|mmHg FiO2|__|__|__|%
  - Score de toux : 0 ☐ 1 ☐ 2 ☐ 3 ☐ 4 ☐ 5 ☐
  - Score d'encombrement : 0 ☐ 1 ☐ 2 ☐ 3 ☐ 4 ☐
  - Patients du groupe A (assisté) : TVS-TT Succès ☐ Echec ☐ (-> VNI prophylactique)
- Evaluer extubabilité si succès du TVS :
  - Force de toux acceptable (≥3) : Oui ☐ Non ☐
  - Encombrement acceptable (≤2) : Oui ☐ Non ☐
  - Absence d'AG dans les 24h qui suivent : Oui ☐ Non ☐
  - Pas d'argument pour une obstruction laryngée : Oui ☐ Non ☐

➔ Si ≥3 critères « oui » : extubation proposée :

➔ Heure d'extubation (hh :mm): |__|__|:|__|__|

Et raison de la non-extubation le cas échéant : ____________________________

Remplir si le patient n'est plus intubé à un moment de J18 (même quelques minutes ou depuis plusieurs jours)

Patient extubé AUJOURD'HUI OUI ☐ NON ☐

Sinon Date de la dernière extubation |__|__|/|__|__|/|__|__| Heure |__|__|:|__|__|

- Extubation (si réalisée AUJOURD'HUI)
  - programmée☐ (selon protocole☐ violation protocole☐)
  - auto-extubation ☐
- VNI prophylactique post-extubation OUI ☐ NON ☐
  - *Si VNI prophylactique*
  - selon protocole ☐ (TVS-TT (-)☐ âge>65ans☐ BPCO☐ Insuffisance cardiaque chronique ☐ Insuffisance respiratoire chronique☐, PaCO2>45 en fin de TVS-TT☐) violation protocole ☐
  - Durée cumulée sur les 24h : |__|__|h
  - *Si absence de VNI prophylactique*
  - selon protocole ☐ violation protocole ☐ contre-indication ☐
- VNI curative post-extubation NON ☐ selon protocole ☐ violation protocole ☐
- Optiflow post-extubation (non recommandé) OUI ☐ NON ☐
- Désencombrement bronchique : NON ☐ Toux manuellement assistée ☐ Toux assistée instrumentale ☐

Réintubation ce jour : OUI ☐ NON ☐ : si OUI Heure : |__|__|:|__|__|
(critère(s) Neurologique☐ Hémodynamique ☐ autre ☐
Respiratoire ☐ (épuisement ☐ Encombrement ☐ Pneumopathie☐ OAP☐ Dyspnée laryngée☐))
Réintubation pour chirurgie avec intubation <24h OUI☐ NON ☐

A la fin de J18 le patient est :
Intubé ☐ Extubé ☐
Poursuite du protocole de sevrage ☐
Sortie du protocole de sevrage ☐ (remplir la partie « sortie d'essai »)
Poids du jour : |__|__|__| kg

J19 = |__|__|/|__|__/|__|__| de 0h à 23h59

Remplir si patient intubé le matin de J19

- Critères préalables de sevrabilité : Présents (-> Faire TVS) ☐ Absents☐
- TVS : Succès ☐ Echec ☐ (maintien intubation)
- Si succès :
  - Gaz du sang : pH |__|,|__|__|. pCO2 |__|__|__|mmHg pO2 |__|__|__|mmHg FiO2|__|__|__|%
  - Score de toux : 0 ☐ 1 ☐ 2 ☐ 3 ☐ 4 ☐ 5 ☐
  - Score d'encombrement : 0 ☐ 1 ☐ 2 ☐ 3 ☐ 4 ☐
  - Patients du groupe A (assisté) : TVS-TT Succès ☐ Echec ☐ (-> VNI prophylactique)
- Evaluer extubabilité si succès du TVS :
  - Force de toux acceptable (≥3) : Oui ☐ Non ☐
  - Encombrement acceptable (≤2) : Oui ☐ Non ☐
  - Absence d'AG dans les 24h qui suivent : Oui ☐ Non ☐
  - Pas d'argument pour une obstruction laryngée : Oui ☐ Non ☐

➔ Si ≥3 critères « oui » : extubation proposée :

➔ Heure d'extubation (hh :mm): |__|__|:|__|__|

Et raison de la non-extubation le cas échéant : ____________________________

Remplir si le patient n'est plus intubé à un moment de J19 (même quelques minutes ou depuis plusieurs jours)

Patient extubé AUJOURD'HUI OUI ☐ NON ☐

Sinon Date de la dernière extubation |__|__|/|__|__|/|__|__| Heure |__|__|:|__|__|

- Extubation (si réalisée AUJOURD'HUI)
  - programmée☐ (selon protocole☐ violation protocole☐)
  - auto-extubation ☐
- VNI prophylactique post-extubation OUI ☐ NON ☐
  - *Si VNI prophylactique*
  - selon protocole ☐ (TVS-TT (-)☐ âge>65ans☐ BPCO☐ Insuffisance cardiaque chronique ☐ Insuffisance respiratoire chronique☐, PaCO2>45 en fin de TVS-TT☐) violation protocole ☐
  - Durée cumulée sur les 24h : |__|__|h
  - *Si absence de VNI prophylactique*
  - selon protocole ☐ violation protocole ☐ contre-indication ☐
- VNI curative post-extubation NON ☐ selon protocole ☐ violation protocole ☐
- Optiflow post-extubation (non recommandé) OUI ☐ NON ☐
- Désencombrement bronchique : NON ☐ Toux manuellement assistée ☐ Toux assistée instrumentale ☐

Réintubation ce jour : OUI ☐ NON ☐ : si OUI Heure : |__|__|:|__|__|

(critère(s) Neurologique☐ Hémodynamique ☐ autre ☐

Respiratoire ☐ (épuisement ☐ Encombrement ☐ Pneumopathie☐ OAP☐ Dyspnée laryngée☐))

Réintubation pour chirurgie avec intubation <24h OUI☐ NON ☐

A la fin de J19 le patient est :

Intubé ☐ Extubé ☐

Poursuite du protocole de sevrage ☐

Sortie du protocole de sevrage ☐ (remplir la partie « sortie d'essai »)

Poids du jour : |__|__|__|kg

J20 = |__|__|/|__|__/|__|__| de 0h à 23h59

Remplir si patient intubé le matin de J20

- Critères préalables de sevrabilité : Présents (-> Faire TVS) ☐ Absents☐
- TVS : Succès ☐ Echec ☐ (maintien intubation)
- Si succès :
  - Gaz du sang : pH |__|,|__|__|. pCO2 |__|__|__|mmHg pO2 |__|__|__|mmHg FiO2|__|__|__|%
  - Score de toux : 0 ☐ 1 ☐ 2 ☐ 3 ☐ 4 ☐ 5 ☐
  - Score d'encombrement : 0 ☐ 1 ☐ 2 ☐ 3 ☐ 4 ☐
  - Patients du groupe A (assisté) : TVS-TT Succès ☐ Echec ☐ (-> VNI prophylactique)
- Evaluer extubabilité si succès du TVS :
  - Force de toux acceptable (≥3) : Oui ☐ Non ☐
  - Encombrement acceptable (≤2) : Oui ☐ Non ☐
  - Absence d'AG dans les 24h qui suivent : Oui ☐ Non ☐
  - Pas d'argument pour une obstruction laryngée : Oui ☐ Non ☐

➔ Si ≥3 critères « oui » : extubation proposée :

➔ Heure d'extubation (hh :mm): |__|__|:|__|__|

Et raison de la non-extubation le cas échéant : ____________________________

Remplir si le patient n'est plus intubé à un moment de J20 (même quelques minutes ou depuis plusieurs jours)

Patient extubé AUJOURD'HUI OUI ☐ NON ☐

Sinon Date de la dernière extubation |__|__|/|__|__|/|__|__| Heure |__|__|:|__|__|

- Extubation (si réalisée AUJOURD'HUI)
  - programmée☐ (selon protocole☐ violation protocole☐)
  - auto-extubation ☐
- VNI prophylactique post-extubation OUI ☐ NON ☐
  - *Si VNI prophylactique*
  - selon protocole ☐ (TVS-TT (-)☐ âge>65ans☐ BPCO☐ Insuffisance cardiaque chronique ☐ Insuffisance respiratoire chronique☐, PaCO2>45 en fin de TVS-TT☐) violation protocole ☐
  - Durée cumulée sur les 24h : |__|__|h
  - *Si absence de VNI prophylactique*
  - selon protocole ☐ violation protocole ☐ contre-indication ☐
- VNI curative post-extubation NON ☐ selon protocole ☐ violation protocole ☐
- Optiflow post-extubation (non recommandé) OUI ☐ NON ☐
- Désencombrement bronchique : NON ☐ Toux manuellement assistée ☐
  Toux assistée instrumentale ☐

Réintubation ce jour : OUI ☐ NON ☐ : si OUI Heure : |__|__|:|__|__|

(critère(s) Neurologique☐ Hémodynamique ☐ autre ☐

Respiratoire ☐ (épuisement ☐ Encombrement ☐ Pneumopathie☐ OAP☐ Dyspnée laryngée☐))

Réintubation pour chirurgie avec intubation <24h OUI☐ NON ☐

A la fin de J20 le patient est :

Intubé ☐ Extubé ☐

Poursuite du protocole de sevrage ☐

Sortie du protocole de sevrage ☐ (remplir la partie « sortie d'essai »)

Poids du jour : |__|__|__| kg

J21 = |__|__|/|__|__/|__|__| de 0h à 23h59

Remplir si patient intubé le matin de J21

- Critères préalables de sevrabilité : Présents (-> Faire TVS) ☐ Absents ☐
- TVS : Succès ☐ Echec ☐ (maintien intubation)
- Si succès :
  - Gaz du sang : pH |__|,|__|__|. pCO2 |__|__|__|mmHg pO2 |__|__|__|mmHg FiO2|__|__|__|%
  - Score de toux : 0 ☐ 1 ☐ 2 ☐ 3 ☐ 4 ☐ 5 ☐
  - Score d'encombrement : 0 ☐ 1 ☐ 2 ☐ 3 ☐ 4 ☐
  - Patients du groupe A (assisté) : TVS-TT Succès ☐ Echec ☐ (-> VNI prophylactique)
- Evaluer extubabilité si succès du TVS :
  - Force de toux acceptable (≥3) : Oui ☐ Non ☐
  - Encombrement acceptable (≤2) : Oui ☐ Non ☐
  - Absence d'AG dans les 24h qui suivent : Oui ☐ Non ☐
  - Pas d'argument pour une obstruction laryngée : Oui ☐ Non ☐

➔ Si ≥3 critères « oui » : extubation proposée :

➔ Heure d'extubation (hh :mm): |__|__|:|__|__|

Et raison de la non-extubation le cas échéant : ____________________________

Remplir si le patient n'est plus intubé à un moment de J21 (même quelques minutes ou depuis plusieurs jours)

Patient extubé AUJOURD'HUI OUI ☐ NON ☐

Sinon Date de la dernière extubation |__|__|/|__|__|/|__|__| Heure |__|__|:|__|__|

- Extubation (si réalisée AUJOURD'HUI)
  - programmée☐ (selon protocole☐ violation protocole☐)
  - auto-extubation ☐
- VNI prophylactique post-extubation OUI ☐ NON ☐
  - *Si VNI prophylactique*
  - selon protocole ☐ (TVS-TT (-)☐ âge>65ans☐ BPCO☐ Insuffisance cardiaque chronique ☐ Insuffisance respiratoire chronique☐, PaCO2>45 en fin de TVS-TT☐) violation protocole ☐
  - Durée cumulée sur les 24h : |__|__|h
  - *Si absence de VNI prophylactique*
  - selon protocole ☐ violation protocole ☐ contre-indication ☐
- VNI curative post-extubation NON ☐ selon protocole ☐ violation protocole ☐
- Optiflow post-extubation (non recommandé) OUI ☐ NON ☐
- Désencombrement bronchique : NON ☐ Toux manuellement assistée ☐
  Toux assistée instrumentale ☐

Réintubation ce jour : OUI ☐ NON ☐ : si OUI Heure : |__|__|:|__|__|
(critère(s) Neurologique☐ Hémodynamique ☐ autre ☐
Respiratoire ☐ (épuisement ☐ Encombrement ☐ Pneumopathie☐ OAP☐ Dyspnée laryngée☐))
Réintubation pour chirurgie avec intubation <24h OUI☐ NON ☐

A la fin de J21 le patient est :
Intubé ☐ Extubé ☐
Poursuite du protocole de sevrage ☐
Sortie du protocole de sevrage ☐ (remplir la partie « sortie d'essai »)
Poids du jour : |__|__|__| kg

J22 = |__|__|/|__|__/|__|__| de 0h à 23h59

Remplir si patient intubé le matin de J22

- Critères préalables de sevrabilité : Présents (-> Faire TVS) ☐ Absents ☐
- TVS : Succès ☐ Echec ☐ (maintien intubation)
- Si succès :
  - Gaz du sang : pH |__|,|__|__|. pCO2 |__|__|__| mmHg pO2 |__|__|__| mmHg FiO2 |__|__|__| %
  - Score de toux : 0 ☐ 1 ☐ 2 ☐ 3 ☐ 4 ☐ 5 ☐
  - Score d'encombrement : 0 ☐ 1 ☐ 2 ☐ 3 ☐ 4 ☐
  - Patients du groupe A (assisté) : TVS-TT Succès ☐ Echec ☐ (-> VNI prophylactique)
- Evaluer extubabilité si succès du TVS :
  - Force de toux acceptable (≥3) : Oui ☐ Non ☐
  - Encombrement acceptable (≤2) : Oui ☐ Non ☐
  - Absence d'AG dans les 24h qui suivent : Oui ☐ Non ☐
  - Pas d'argument pour une obstruction laryngée : Oui ☐ Non ☐

➔ Si ≥3 critères « oui » : extubation proposée :

➔ Heure d'extubation (hh :mm): |__|__|:|__|__|

Et raison de la non-extubation le cas échéant : ____________________________

Remplir si le patient n'est plus intubé à un moment de J22 (même quelques minutes ou depuis plusieurs jours)

Patient extubé AUJOURD'HUI OUI ☐ NON ☐

Sinon Date de la dernière extubation |__|__|/|__|__|/|__|__| Heure |__|__|:|__|__|

- Extubation (si réalisée AUJOURD'HUI)
  - programmée ☐ (selon protocole ☐ violation protocole ☐)
  - auto-extubation ☐
- VNI prophylactique post-extubation OUI ☐ NON ☐
  - *Si VNI prophylactique*
  - selon protocole ☐ (TVS-TT (-) ☐ âge>65ans ☐ BPCO ☐ Insuffisance cardiaque chronique ☐ Insuffisance respiratoire chronique ☐, PaCO2>45 en fin de TVS-TT ☐) violation protocole ☐
  - Durée cumulée sur les 24h : |__|__| h
  - *Si absence de VNI prophylactique*
  - selon protocole ☐ violation protocole ☐ contre-indication ☐
- VNI curative post-extubation NON ☐ selon protocole ☐ violation protocole ☐
- Optiflow post-extubation (non recommandé) OUI ☐ NON ☐
- Désencombrement bronchique : NON ☐ Toux manuellement assistée ☐
  Toux assistée instrumentale ☐

Réintubation ce jour : OUI ☐ NON ☐ : si OUI Heure : |__|__|:|__|__|

(critère(s) Neurologique ☐ Hémodynamique ☐ autre ☐

Respiratoire ☐ (épuisement ☐ Encombrement ☐ Pneumopathie ☐ OAP ☐ Dyspnée laryngée ☐))

Réintubation pour chirurgie avec intubation <24h OUI ☐ NON ☐

A la fin de J22 le patient est :

Intubé ☐ Extubé ☐

Poursuite du protocole de sevrage ☐

Sortie du protocole de sevrage ☐ (remplir la partie « sortie d'essai »)

Poids du jour : |__|__|__| kg

J23 = |__|__|/|__|__/|__|__| de 0h à 23h59

Remplir si patient intubé le matin de J23

- Critères préalables de sevrabilité : Présents (-> Faire TVS) ☐ Absents☐
- TVS : Succès ☐ Echec ☐ (maintien intubation)
- Si succès :
  - Gaz du sang : pH |__|,|__|__|. pCO2 |__|__|__|mmHg pO2 |__|__|__|mmHg FiO2|__|__|__|%
  - Score de toux : 0 ☐ 1 ☐ 2 ☐ 3 ☐ 4 ☐ 5 ☐
  - Score d'encombrement : 0 ☐ 1 ☐ 2 ☐ 3 ☐ 4 ☐
  - Patients du groupe A (assisté) : TVS-TT Succès ☐ Echec ☐ (-> VNI prophylactique)
- Evaluer extubabilité si succès du TVS :
  - Force de toux acceptable (≥3) : Oui ☐ Non ☐
  - Encombrement acceptable (≤2) : Oui ☐ Non ☐
  - Absence d'AG dans les 24h qui suivent : Oui ☐ Non ☐
  - Pas d'argument pour une obstruction laryngée : Oui ☐ Non ☐

➔ Si ≥3 critères « oui » : extubation proposée :

➔ Heure d'extubation (hh :mm): |__|__|:|__|__|

Et raison de la non-extubation le cas échéant : ______________________________

Remplir si le patient n'est plus intubé à un moment de J23 (même quelques minutes ou depuis plusieurs jours)

Patient extubé AUJOURD'HUI OUI ☐ NON ☐

Sinon Date de la dernière extubation |__|__|/|__|__|/|__|__| Heure |__|__|:|__|__|

- Extubation (si réalisée AUJOURD'HUI)
  - programmée☐ (selon protocole☐ violation protocole☐)
  - auto-extubation ☐
- VNI prophylactique post-extubation OUI ☐ NON ☐
  - *Si VNI prophylactique*
  - selon protocole ☐ (TVS-TT (-)☐ âge>65ans☐ BPCO☐ Insuffisance cardiaque chronique ☐ Insuffisance respiratoire chronique☐, PaCO2>45 en fin de TVS-TT☐) violation protocole ☐
  - Durée cumulée sur les 24h : |__|__|h
  - *Si absence de VNI prophylactique*
  - selon protocole ☐ violation protocole ☐ contre-indication ☐
- VNI curative post-extubation NON ☐ selon protocole ☐ violation protocole ☐
- Optiflow post-extubation (non recommandé) OUI ☐ NON ☐
- Désencombrement bronchique : NON ☐ Toux manuellement assistée ☐
  Toux assistée instrumentale ☐

Réintubation ce jour : OUI ☐ NON ☐ : si OUI Heure : |__|__|:|__|__|

(critère(s) Neurologique☐ Hémodynamique ☐ autre ☐

Respiratoire ☐ (épuisement ☐ Encombrement ☐ Pneumopathie☐ OAP☐ Dyspnée laryngée☐))

Réintubation pour chirurgie avec intubation <24h OUI☐ NON ☐

A la fin de J23 le patient est :

Intubé ☐ Extubé ☐

Poursuite du protocole de sevrage ☐

Sortie du protocole de sevrage ☐ (remplir la partie « sortie d'essai »)

Poids du jour : |__|__|__| kg

J24 = |__|__|/|__|__/|__|__| de 0h à 23h59

Remplir si patient intubé le matin de J24

- Critères préalables de sevrabilité : Présents (-> Faire TVS) ☐ Absents☐
- TVS : Succès ☐ Echec ☐ (maintien intubation)
- Si succès :
  - Gaz du sang : pH |__|,|__|__|. pCO2 |__|__|__|mmHg pO2 |__|__|__|mmHg FiO2|__|__|__|%
  - Score de toux : 0 ☐ 1 ☐ 2 ☐ 3 ☐ 4 ☐ 5 ☐
  - Score d'encombrement : 0 ☐ 1 ☐ 2 ☐ 3 ☐ 4 ☐
  - Patients du groupe A (assisté) : TVS-TT Succès ☐ Echec ☐ (-> VNI prophylactique)
- Evaluer extubabilité si succès du TVS :
  - Force de toux acceptable (≥3) : Oui ☐ Non ☐
  - Encombrement acceptable (≤2) : Oui ☐ Non ☐
  - Absence d'AG dans les 24h qui suivent : Oui ☐ Non ☐
  - Pas d'argument pour une obstruction laryngée : Oui ☐ Non ☐

➔ Si ≥3 critères « oui » : extubation proposée :

➔ Heure d'extubation (hh :mm): |__|__|:|__|__|

Et raison de la non-extubation le cas échéant : ____________________________

Remplir si le patient n'est plus intubé à un moment de J24 (même quelques minutes ou depuis plusieurs jours)

Patient extubé AUJOURD'HUI OUI ☐ NON ☐

Sinon Date de la dernière extubation |__|__|/|__|__|/|__|__| Heure |__|__|:|__|__|

- Extubation (si réalisée AUJOURD'HUI)
  - programmée☐ (selon protocole☐ violation protocole☐)
  - auto-extubation ☐
- VNI prophylactique post-extubation OUI ☐ NON ☐
  - *Si VNI prophylactique*
  - selon protocole ☐ (TVS-TT (-)☐ âge>65ans☐ BPCO☐ Insuffisance cardiaque chronique ☐ Insuffisance respiratoire chronique☐, PaCO2>45 en fin de TVS-TT☐) violation protocole ☐
  - Durée cumulée sur les 24h : |__|__|h
  - *Si absence de VNI prophylactique*
  - selon protocole ☐ violation protocole ☐ contre-indication ☐
- VNI curative post-extubation NON ☐ selon protocole ☐ violation protocole ☐
- Optiflow post-extubation (non recommandé) OUI ☐ NON ☐
- Désencombrement bronchique : NON ☐ Toux manuellement assistée ☐
  Toux assistée instrumentale ☐

Réintubation ce jour : OUI ☐ NON ☐ : si OUI Heure : |__|__|:|__|__|
(critère(s) Neurologique☐ Hémodynamique ☐ autre ☐
Respiratoire ☐ (épuisement ☐ Encombrement ☐ Pneumopathie☐ OAP☐ Dyspnée laryngée☐))
Réintubation pour chirurgie avec intubation <24h OUI☐ NON ☐

A la fin de J24 le patient est :
Intubé ☐ Extubé ☐
Poursuite du protocole de sevrage ☐
Sortie du protocole de sevrage ☐ (remplir la partie « sortie d'essai »)
Poids du jour : |__|__|__| kg

J25 = |__|__|/|__|__/|__|__| de 0h à 23h59

Remplir si patient intubé le matin de J25

- Critères préalables de sevrabilité : Présents (-> Faire TVS) ☐ Absents☐
- TVS : Succès ☐ Echec ☐ (maintien intubation)
- Si succès :
  - Gaz du sang : pH |__|,|__|__|. pCO2 |__|__|__|mmHg pO2 |__|__|__|mmHg FiO2|__|__|__|%
  - Score de toux : 0 ☐ 1 ☐ 2 ☐ 3 ☐ 4 ☐ 5 ☐
  - Score d'encombrement : 0 ☐ 1 ☐ 2 ☐ 3 ☐ 4 ☐
  - Patients du groupe A (assisté) : TVS-TT Succès ☐ Echec ☐ (-> VNI prophylactique)
- Evaluer extubabilité si succès du TVS :
  - Force de toux acceptable (≥3) : Oui ☐ Non ☐
  - Encombrement acceptable (≤2) : Oui ☐ Non ☐
  - Absence d'AG dans les 24h qui suivent : Oui ☐ Non ☐
  - Pas d'argument pour une obstruction laryngée : Oui ☐ Non ☐

➔ Si ≥3 critères « oui » : extubation proposée :

➔ Heure d'extubation (hh :mm): |__|__|:|__|__|

Et raison de la non-extubation le cas échéant : ____________________________

Remplir si le patient n'est plus intubé à un moment de J25 (même quelques minutes ou depuis plusieurs jours)

Patient extubé AUJOURD'HUI OUI ☐ NON ☐

Sinon Date de la dernière extubation |__|__|/|__|__|/|__|__| Heure |__|__|:|__|__|

- Extubation (si réalisée AUJOURD'HUI)
  - programmée☐ (selon protocole☐ violation protocole☐)
  - auto-extubation ☐
- VNI prophylactique post-extubation OUI ☐ NON ☐
  - *Si VNI prophylactique*
  - selon protocole ☐ (TVS-TT (-)☐ âge>65ans☐ BPCO☐ Insuffisance cardiaque chronique ☐ Insuffisance respiratoire chronique☐, PaCO2>45 en fin de TVS-TT☐) violation protocole ☐
  - Durée cumulée sur les 24h : |__|__|h
  - *Si absence de VNI prophylactique*
  - selon protocole ☐ violation protocole ☐ contre-indication ☐
- VNI curative post-extubation NON ☐ selon protocole ☐ violation protocole ☐
- Optiflow post-extubation (non recommandé) OUI ☐ NON ☐
- Désencombrement bronchique : NON ☐ Toux manuellement assistée ☐
  Toux assistée instrumentale ☐

Réintubation ce jour : OUI ☐ NON ☐ : si OUI Heure : |__|__|:|__|__|
(critère(s) Neurologique☐ Hémodynamique ☐ autre ☐
Respiratoire ☐ (épuisement ☐ Encombrement ☐ Pneumopathie☐ OAP☐ Dyspnée laryngée☐))
Réintubation pour chirurgie avec intubation <24h OUI☐ NON ☐

A la fin de J25 le patient est :
Intubé ☐ Extubé ☐
Poursuite du protocole de sevrage ☐
Sortie du protocole de sevrage ☐ (remplir la partie « sortie d'essai »)
Poids du jour : |__|__|__| kg

J26 = |__|__|/|__|__/|__|__| de 0h à 23h59

Remplir si patient intubé le matin de J26

- Critères préalables de sevrabilité : Présents (-> Faire TVS) ☐ Absents☐
- TVS : Succès ☐ Echec ☐ (maintien intubation)
- Si succès :
  - Gaz du sang : pH |__|,|__|__|. pCO2 |__|__|__|mmHg pO2 |__|__|__|mmHg FiO2|__|__|__|%
  - Score de toux : 0 ☐ 1 ☐ 2 ☐ 3 ☐ 4 ☐ 5 ☐
  - Score d'encombrement : 0 ☐ 1 ☐ 2 ☐ 3 ☐ 4 ☐
  - Patients du groupe A (assisté) : TVS-TT Succès ☐ Echec ☐ (-> VNI prophylactique)
- Evaluer extubabilité si succès du TVS :
  - Force de toux acceptable (≥3) : Oui ☐ Non ☐
  - Encombrement acceptable (≤2) : Oui ☐ Non ☐
  - Absence d'AG dans les 24h qui suivent : Oui ☐ Non ☐
  - Pas d'argument pour une obstruction laryngée : Oui ☐ Non ☐

➔ Si ≥3 critères « oui » : extubation proposée :

➔ Heure d'extubation (hh :mm): |__|__|:|__|__|

Et raison de la non-extubation le cas échéant : ______________________________

Remplir si le patient n'est plus intubé à un moment de J26 (même quelques minutes ou depuis plusieurs jours)

Patient extubé AUJOURD'HUI OUI ☐ NON ☐

Sinon Date de la dernière extubation |__|__|/|__|__|/|__|__| Heure |__|__|:|__|__|

- Extubation (si réalisée AUJOURD'HUI)
  - programmée☐ (selon protocole☐ violation protocole☐)
  - auto-extubation ☐
- VNI prophylactique post-extubation OUI ☐ NON ☐
  - *Si VNI prophylactique*
  - selon protocole ☐ (TVS-TT (-)☐ âge>65ans☐ BPCO☐ Insuffisance cardiaque chronique ☐ Insuffisance respiratoire chronique☐, PaCO2>45 en fin de TVS-TT☐) violation protocole ☐
  - Durée cumulée sur les 24h : |__|__|h
  - *Si absence de VNI prophylactique*
  - selon protocole ☐ violation protocole ☐ contre-indication ☐
- VNI curative post-extubation NON ☐ selon protocole ☐ violation protocole ☐
- Optiflow post-extubation (non recommandé) OUI ☐ NON ☐
- Désencombrement bronchique : NON ☐ Toux manuellement assistée ☐
  Toux assistée instrumentale ☐

Réintubation ce jour : OUI ☐ NON ☐ : si OUI Heure : |__|__|:|__|__|

(critère(s) Neurologique☐ Hémodynamique ☐ autre ☐

Respiratoire ☐ (épuisement ☐ Encombrement ☐ Pneumopathie☐ OAP☐ Dyspnée laryngée☐))

Réintubation pour chirurgie avec intubation <24h OUI☐ NON ☐

A la fin de J26 le patient est :

Intubé ☐ Extubé ☐

Poursuite du protocole de sevrage ☐

Sortie du protocole de sevrage ☐ (remplir la partie « sortie d'essai »)

Poids du jour : |__|__|__|kg

J27 = |__|__|/|__|__/|__|__| de 0h à 23h59

Remplir si patient intubé le matin de J27

- Critères préalables de sevrabilité : Présents (-> Faire TVS) ☐ Absents☐
- TVS : Succès ☐ Echec ☐ (maintien intubation)
- Si succès :
  - Gaz du sang : pH |__|,|__|__|. pCO2 |__|__|__|mmHg pO2 |__|__|__|mmHg FiO2|__|__|__|%
  - Score de toux : 0 ☐ 1 ☐ 2 ☐ 3 ☐ 4 ☐ 5 ☐
  - Score d'encombrement : 0 ☐ 1 ☐ 2 ☐ 3 ☐ 4 ☐
  - Patients du groupe A (assisté) : TVS-TT Succès ☐ Echec ☐ (-> VNI prophylactique)
- Evaluer extubabilité si succès du TVS :
  - Force de toux acceptable (≥3) : Oui ☐ Non ☐
  - Encombrement acceptable (≤2) : Oui ☐ Non ☐
  - Absence d'AG dans les 24h qui suivent : Oui ☐ Non ☐
  - Pas d'argument pour une obstruction laryngée : Oui ☐ Non ☐

➔ Si ≥3 critères « oui » : extubation proposée :

➔ Heure d'extubation (hh :mm): |__|__|:|__|__|

Et raison de la non-extubation le cas échéant : ____________________________

Remplir si le patient n'est plus intubé à un moment de J27 (même quelques minutes ou depuis plusieurs jours)

Patient extubé AUJOURD'HUI OUI ☐ NON ☐

Sinon Date de la dernière extubation |__|__|/|__|__|/|__|__| Heure |__|__|:|__|__|

- Extubation (si réalisée AUJOURD'HUI)
  - programmée☐ (selon protocole☐ violation protocole☐)
  - auto-extubation ☐
- VNI prophylactique post-extubation OUI ☐ NON ☐
  - *Si VNI prophylactique*
  - selon protocole ☐ (TVS-TT (-)☐ âge>65ans☐ BPCO☐ Insuffisance cardiaque chronique ☐ Insuffisance respiratoire chronique☐, PaCO2>45 en fin de TVS-TT☐) violation protocole ☐
  - Durée cumulée sur les 24h : |__|__|h
  - *Si absence de VNI prophylactique*
  - selon protocole ☐ violation protocole ☐ contre-indication ☐
- VNI curative post-extubation NON ☐ selon protocole ☐ violation protocole ☐
- Optiflow post-extubation (non recommandé) OUI ☐ NON ☐
- Désencombrement bronchique : NON ☐ Toux manuellement assistée ☐ Toux assistée instrumentale ☐

Réintubation ce jour : OUI ☐ NON ☐ : si OUI Heure : |__|__|:|__|__|
(critère(s) Neurologique☐ Hémodynamique ☐ autre ☐
Respiratoire ☐ (épuisement ☐ Encombrement ☐ Pneumopathie☐ OAP☐ Dyspnée laryngée☐))
Réintubation pour chirurgie avec intubation <24h OUI☐ NON ☐

A la fin de J27 le patient est :
Intubé ☐ Extubé ☐
Poursuite du protocole de sevrage ☐
Sortie du protocole de sevrage ☐ (remplir la partie « sortie d'essai »)
Poids du jour : |__|__|__| kg

J28 = |__|__|/|__|__/|__|__| de 0h à 23h59

Remplir si patient intubé le matin de J28

- Critères préalables de sevrabilité : Présents (-> Faire TVS) ☐ Absents ☐
- TVS : Succès ☐ Echec ☐ (maintien intubation)
- Si succès :
    - Gaz du sang : pH |__|,|__|__|. pCO2 |__|__|__|mmHg pO2 |__|__|__|mmHg FiO2|__|__|__|%
    - Score de toux : 0 ☐ 1 ☐ 2 ☐ 3 ☐ 4 ☐ 5 ☐
    - Score d'encombrement : 0 ☐ 1 ☐ 2 ☐ 3 ☐ 4 ☐
    - Patients du groupe A (assisté) : TVS-TT Succès ☐ Echec ☐ (-> VNI prophylactique)
- Evaluer extubabilité si succès du TVS :
    - Force de toux acceptable (≥3) : Oui ☐ Non ☐
    - Encombrement acceptable (≤2) : Oui ☐ Non ☐
    - Absence d'AG dans les 24h qui suivent : Oui ☐ Non ☐
    - Pas d'argument pour une obstruction laryngée : Oui ☐ Non ☐

➔ Si ≥3 critères « oui » : extubation proposée :

➔ Heure d'extubation (hh :mm): |__|__|:|__|__|

Et raison de la non-extubation le cas échéant : ____________________________

Remplir si le patient n'est plus intubé à un moment de J28 (même quelques minutes ou depuis plusieurs jours)

Patient extubé AUJOURD'HUI OUI ☐ NON ☐

Sinon Date de la dernière extubation |__|__|/|__|__|/|__|__| Heure |__|__|:|__|__|

- Extubation (si réalisée AUJOURD'HUI)
    - programmée☐ (selon protocole☐ violation protocole☐)
    - auto-extubation ☐
- VNI prophylactique post-extubation OUI ☐ NON ☐
    - *Si VNI prophylactique*
    - selon protocole ☐ (TVS-TT (-)☐ âge>65ans☐ BPCO☐ Insuffisance cardiaque chronique ☐ Insuffisance respiratoire chronique☐, PaCO2>45 en fin de TVS-TT☐) violation protocole ☐
    - Durée cumulée sur les 24h : |__|__|h
    - *Si absence de VNI prophylactique*
    - selon protocole ☐ violation protocole ☐ contre-indication ☐
- VNI curative post-extubation NON ☐ selon protocole ☐ violation protocole ☐
- Optiflow post-extubation (non recommandé) OUI ☐ NON ☐
- Désencombrement bronchique : NON ☐ Toux manuellement assistée ☐ Toux assistée instrumentale ☐

Réintubation ce jour : OUI ☐ NON ☐ : si OUI Heure : |__|__|:|__|__|
(critère(s) Neurologique☐ Hémodynamique ☐ autre ☐
Respiratoire ☐ (épuisement ☐ Encombrement ☐ Pneumopathie☐ OAP☐ Dyspnée laryngée☐))
Réintubation pour chirurgie avec intubation <24h OUI☐ NON ☐

A la fin de J28 le patient est :
Intubé ☐ Extubé ☐
Poursuite du protocole de sevrage ☐
Sortie du protocole de sevrage ☐ (remplir la partie « sortie d'essai »)
Poids du jour : |__|__|__| kg

J29 = |__|__|/|__|__/|__|__| de 0h à 23h59

Remplir si patient intubé le matin de J29

- Critères préalables de sevrabilité : Présents (-> Faire TVS) ☐ Absents☐
- TVS : Succès ☐ Echec ☐ (maintien intubation)
- Si succès :
  - Gaz du sang : pH |__|,|__|__|. pCO2 |__|__|__|mmHg pO2 |__|__|__|mmHg FiO2|__|__|__|%
  - Score de toux : 0 ☐ 1 ☐ 2 ☐ 3 ☐ 4 ☐ 5 ☐
  - Score d'encombrement : 0 ☐ 1 ☐ 2 ☐ 3 ☐ 4 ☐
  - Patients du groupe A (assisté) : TVS-TT Succès ☐ Echec ☐ (-> VNI prophylactique)
- Evaluer extubabilité si succès du TVS :
  - Force de toux acceptable (≥3) : Oui ☐ Non ☐
  - Encombrement acceptable (≤2) : Oui ☐ Non ☐
  - Absence d'AG dans les 24h qui suivent : Oui ☐ Non ☐
  - Pas d'argument pour une obstruction laryngée : Oui ☐ Non ☐

➔ Si ≥3 critères « oui » : extubation proposée :

➔ Heure d'extubation (hh :mm): |__|__|:|__|__|

Et raison de la non-extubation le cas échéant : ____________________________

Remplir si le patient n'est plus intubé à un moment de J29 (même quelques minutes ou depuis plusieurs jours)

Patient extubé AUJOURD'HUI OUI ☐ NON ☐

Sinon Date de la dernière extubation |__|__|/|__|__|/|__|__| Heure |__|__|:|__|__|

- Extubation (si réalisée AUJOURD'HUI)
  - programmée☐ (selon protocole☐ violation protocole☐)
  - auto-extubation ☐
- VNI prophylactique post-extubation OUI ☐ NON ☐
  - *Si VNI prophylactique*
  - selon protocole ☐ (TVS-TT (-)☐ âge>65ans☐ BPCO☐ Insuffisance cardiaque chronique ☐ Insuffisance respiratoire chronique☐, PaCO2>45 en fin de TVS-TT☐) violation protocole ☐
  - Durée cumulée sur les 24h : |__|__|h
  - *Si absence de VNI prophylactique*
  - selon protocole ☐ violation protocole ☐ contre-indication ☐
- VNI curative post-extubation NON ☐ selon protocole ☐ violation protocole ☐
- Optiflow post-extubation (non recommandé) OUI ☐ NON ☐
- Désencombrement bronchique : NON ☐ Toux manuellement assistée ☐
  Toux assistée instrumentale ☐

Réintubation ce jour : OUI ☐ NON ☐ : si OUI Heure : |__|__|:|__|__|

(critère(s) Neurologique☐ Hémodynamique ☐ autre ☐

Respiratoire ☐ (épuisement ☐ Encombrement ☐ Pneumopathie☐ OAP☐ Dyspnée laryngée☐))

Réintubation pour chirurgie avec intubation <24h OUI☐ NON ☐

A la fin de J29 le patient est :

Intubé ☐ Extubé ☐

Poursuite du protocole de sevrage ☐

Sortie du protocole de sevrage ☐ (remplir la partie « sortie d'essai »)

Poids du jour : |__|__|__| kg

J30 = |__|__|/|__|__/|__|__| de 0h à 23h59

Remplir si patient intubé le matin de J30

- Critères préalables de sevrabilité : Présents (-> Faire TVS) ☐ Absents☐
- TVS : Succès ☐ Echec ☐ (maintien intubation)
- Si succès :
  - Gaz du sang : pH |__|,|__|__|. pCO2 |__|__|__|mmHg pO2 |__|__|__|mmHg FiO2|__|__|__|%
  - Score de toux : 0 ☐ 1 ☐ 2 ☐ 3 ☐ 4 ☐ 5 ☐
  - Score d'encombrement : 0 ☐ 1 ☐ 2 ☐ 3 ☐ 4 ☐
  - Patients du groupe A (assisté) : TVS-TT Succès ☐ Echec ☐ (-> VNI prophylactique)
- Evaluer extubabilité si succès du TVS :
  - Force de toux acceptable (≥3) : Oui ☐ Non ☐
  - Encombrement acceptable (≤2) : Oui ☐ Non ☐
  - Absence d'AG dans les 24h qui suivent : Oui ☐ Non ☐
  - Pas d'argument pour une obstruction laryngée : Oui ☐ Non ☐

➔ Si ≥3 critères « oui » : extubation proposée :

➔ Heure d'extubation (hh :mm): |__|__|:|__|__|

Et raison de la non-extubation le cas échéant : ____________________________

Remplir si le patient n'est plus intubé à un moment de J30 (même quelques minutes ou depuis plusieurs jours)

Patient extubé AUJOURD'HUI OUI ☐ NON ☐

Sinon Date de la dernière extubation |__|__|/|__|__|/|__|__| Heure |__|__|:|__|__|

- Extubation (si réalisée AUJOURD'HUI)
  - programmée☐ (selon protocole☐ violation protocole☐)
  - auto-extubation ☐
- VNI prophylactique post-extubation OUI ☐ NON ☐
  - *Si VNI prophylactique*
  - selon protocole ☐ (TVS-TT (-)☐ âge>65ans☐ BPCO☐ Insuffisance cardiaque chronique ☐ Insuffisance respiratoire chronique☐, PaCO2>45 en fin de TVS-TT☐) violation protocole ☐
  - Durée cumulée sur les 24h : |__|__|h
  - *Si absence de VNI prophylactique*
  - selon protocole ☐ violation protocole ☐ contre-indication ☐
- VNI curative post-extubation NON ☐ selon protocole ☐ violation protocole ☐
- Optiflow post-extubation (non recommandé) OUI ☐ NON ☐
- Désencombrement bronchique : NON ☐ Toux manuellement assistée ☐
  Toux assistée instrumentale ☐

Réintubation ce jour : OUI ☐ NON ☐ : si OUI Heure : |__|__|:|__|__|
(critère(s) Neurologique☐ Hémodynamique ☐ autre ☐
Respiratoire ☐ (épuisement ☐ Encombrement ☐ Pneumopathie☐ OAP☐ Dyspnée laryngée☐))
Réintubation pour chirurgie avec intubation <24h OUI☐ NON ☐

A la fin de J30 le patient est :
Intubé ☐ Extubé ☐
Poursuite du protocole de sevrage ☐
Sortie du protocole de sevrage ☐ (remplir la partie « sortie d'essai »)
Poids du jour : |__|__|__| kg

J31 = |__|__|/|__|__/|__|__| de 0h à 23h59

Remplir si patient intubé le matin de J31

- Critères préalables de sevrabilité : Présents (-> Faire TVS) ☐ Absents☐
- TVS : Succès ☐ Echec ☐ (maintien intubation)
- Si succès :
  - Gaz du sang : pH |__|,|__|__|. pCO2 |__|__|__|mmHg pO2 |__|__|__|mmHg FiO2|__|__|__|%
  - Score de toux : 0 ☐ 1 ☐ 2 ☐ 3 ☐ 4 ☐ 5 ☐
  - Score d'encombrement : 0 ☐ 1 ☐ 2 ☐ 3 ☐ 4 ☐
  - Patients du groupe A (assisté) : TVS-TT Succès ☐ Echec ☐ (-> VNI prophylactique)
- Evaluer extubabilité si succès du TVS :
  - Force de toux acceptable (≥3) : Oui ☐ Non ☐
  - Encombrement acceptable (≤2) : Oui ☐ Non ☐
  - Absence d'AG dans les 24h qui suivent : Oui ☐ Non ☐
  - Pas d'argument pour une obstruction laryngée : Oui ☐ Non ☐

➔ Si ≥3 critères « oui » : extubation proposée :

➔ Heure d'extubation (hh :mm): |__|__|:|__|__|

Et raison de la non-extubation le cas échéant : ____________________________

Remplir si le patient n'est plus intubé à un moment de J31 (même quelques minutes ou depuis plusieurs jours)

Patient extubé AUJOURD'HUI OUI ☐ NON ☐

Sinon Date de la dernière extubation |__|__|/|__|__|/|__|__| Heure |__|__|:|__|__|

- Extubation (si réalisée AUJOURD'HUI)
  - programmée☐ (selon protocole☐ violation protocole☐)
  - auto-extubation ☐
- VNI prophylactique post-extubation OUI ☐ NON ☐
  - *Si VNI prophylactique*
  - selon protocole ☐ (TVS-TT (-)☐ âge>65ans☐ BPCO☐ Insuffisance cardiaque chronique ☐ Insuffisance respiratoire chronique☐, PaCO2>45 en fin de TVS-TT☐) violation protocole ☐
  - Durée cumulée sur les 24h : |__|__|h
  - *Si absence de VNI prophylactique*
  - selon protocole ☐ violation protocole ☐ contre-indication ☐
- VNI curative post-extubation NON ☐ selon protocole ☐ violation protocole ☐
- Optiflow post-extubation (non recommandé) OUI ☐ NON ☐
- Désencombrement bronchique : NON ☐ Toux manuellement assistée ☐
  Toux assistée instrumentale ☐

Réintubation ce jour : OUI ☐ NON ☐ : si OUI Heure : |__|__|:|__|__|

(critère(s) Neurologique☐ Hémodynamique ☐ autre ☐

Respiratoire ☐ (épuisement ☐ Encombrement ☐ Pneumopathie☐ OAP☐ Dyspnée laryngée☐))

Réintubation pour chirurgie avec intubation <24h OUI☐ NON ☐

A la fin de J31 le patient est :

Intubé ☐ Extubé ☐

Poursuite du protocole de sevrage ☐

Sortie du protocole de sevrage ☐ (remplir la partie « sortie d'essai »)

Poids du jour : |__|__|__| kg

J32 = |__|__|/|__|__/|__|__| de 0h à 23h59

Remplir si patient intubé le matin de J32

- Critères préalables de sevrabilité : Présents (-> Faire TVS) ☐ Absents☐
- TVS : Succès ☐ Echec ☐ (maintien intubation)
- Si succès :
  - Gaz du sang : pH |__|,|__|__|. pCO2 |__|__|__|mmHg pO2 |__|__|__|mmHg FiO2|__|__|__|%
  - Score de toux : 0 ☐ 1 ☐ 2 ☐ 3 ☐ 4 ☐ 5 ☐
  - Score d'encombrement : 0 ☐ 1 ☐ 2 ☐ 3 ☐ 4 ☐
  - Patients du groupe A (assisté) : TVS-TT Succès ☐ Echec ☐ (-> VNI prophylactique)
- Evaluer extubabilité si succès du TVS :
  - Force de toux acceptable (≥3) : Oui ☐ Non ☐
  - Encombrement acceptable (≤2) : Oui ☐ Non ☐
  - Absence d'AG dans les 24h qui suivent : Oui ☐ Non ☐
  - Pas d'argument pour une obstruction laryngée : Oui ☐ Non ☐

➔ Si ≥3 critères « oui » : extubation proposée :

➔ Heure d'extubation (hh :mm): |__|__|:|__|__|

Et raison de la non-extubation le cas échéant : ______________________________

Remplir si le patient n'est plus intubé à un moment de J32 (même quelques minutes ou depuis plusieurs jours)

Patient extubé AUJOURD'HUI OUI ☐ NON ☐

Sinon Date de la dernière extubation |__|__|/|__|__|/|__|__| Heure |__|__|:|__|__|

- Extubation (si réalisée AUJOURD'HUI)
  - programmée☐ (selon protocole☐ violation protocole☐)
  - auto-extubation ☐
- VNI prophylactique post-extubation OUI ☐ NON ☐
  - *Si VNI prophylactique*
  - selon protocole ☐ (TVS-TT (-)☐ âge>65ans☐ BPCO☐ Insuffisance cardiaque chronique ☐ Insuffisance respiratoire chronique☐, PaCO2>45 en fin de TVS-TT☐) violation protocole ☐
  - Durée cumulée sur les 24h : |__|__|h
  - *Si absence de VNI prophylactique*
  - selon protocole ☐ violation protocole ☐ contre-indication ☐
- VNI curative post-extubation NON ☐ selon protocole ☐ violation protocole ☐
- Optiflow post-extubation (non recommandé) OUI ☐ NON ☐
- Désencombrement bronchique : NON ☐ Toux manuellement assistée ☐
  Toux assistée instrumentale ☐

Réintubation ce jour : OUI ☐ NON ☐ : si OUI Heure : |__|__|:|__|__|
(critère(s) Neurologique☐ Hémodynamique ☐ autre ☐
Respiratoire ☐ (épuisement ☐ Encombrement ☐ Pneumopathie☐ OAP☐ Dyspnée laryngée☐))
Réintubation pour chirurgie avec intubation <24h OUI☐ NON ☐

A la fin de J32 le patient est :
Intubé ☐ Extubé ☐
Poursuite du protocole de sevrage ☐
Sortie du protocole de sevrage ☐ (remplir la partie « sortie d'essai »)
Poids du jour : |__|__|__| kg

J33 = |__|__|/|__|__/|__|__| de 0h à 23h59

Remplir si patient intubé le matin de J33

- Critères préalables de sevrabilité : Présents (-> Faire TVS) ☐ Absents☐
- TVS : Succès ☐ Echec ☐ (maintien intubation)
- Si succès :
  - Gaz du sang : pH |__|,|__|__|. pCO2 |__|__|__|mmHg pO2 |__|__|__|mmHg FiO2|__|__|__|%
  - Score de toux : 0 ☐ 1 ☐ 2 ☐ 3 ☐ 4 ☐ 5 ☐
  - Score d'encombrement : 0 ☐ 1 ☐ 2 ☐ 3 ☐ 4 ☐
  - Patients du groupe A (assisté) : TVS-TT Succès ☐ Echec ☐ (-> VNI prophylactique)
- Evaluer extubabilité si succès du TVS :
  - Force de toux acceptable (≥3) : Oui ☐ Non ☐
  - Encombrement acceptable (≤2) : Oui ☐ Non ☐
  - Absence d'AG dans les 24h qui suivent : Oui ☐ Non ☐
  - Pas d'argument pour une obstruction laryngée : Oui ☐ Non ☐

➔ Si ≥3 critères « oui » : extubation proposée :

➔ Heure d'extubation (hh :mm): |__|__|:|__|__|

Et raison de la non-extubation le cas échéant : ____________________________

Remplir si le patient n'est plus intubé à un moment de J33 (même quelques minutes ou depuis plusieurs jours)

Patient extubé AUJOURD'HUI OUI ☐ NON ☐

Sinon Date de la dernière extubation |__|__|/|__|__|/|__|__| Heure |__|__|:|__|__|

- Extubation (si réalisée AUJOURD'HUI)
  - programmée☐ (selon protocole☐ violation protocole☐)
  - auto-extubation ☐
- VNI prophylactique post-extubation OUI ☐ NON ☐
  - *Si VNI prophylactique*
  - selon protocole ☐ (TVS-TT (-)☐ âge>65ans☐ BPCO☐ Insuffisance cardiaque chronique ☐ Insuffisance respiratoire chronique☐, PaCO2>45 en fin de TVS-TT☐) violation protocole ☐
  - Durée cumulée sur les 24h : |__|__|h
  - *Si absence de VNI prophylactique*
  - selon protocole ☐ violation protocole ☐ contre-indication ☐
- VNI curative post-extubation NON ☐ selon protocole ☐ violation protocole ☐
- Optiflow post-extubation (non recommandé) OUI ☐ NON ☐
- Désencombrement bronchique : NON ☐ Toux manuellement assistée ☐
  Toux assistée instrumentale ☐

Réintubation ce jour : OUI ☐ NON ☐ : si OUI Heure : |__|__|:|__|__|
(critère(s) Neurologique☐ Hémodynamique ☐ autre ☐
Respiratoire ☐ (épuisement ☐ Encombrement ☐ Pneumopathie☐ OAP☐ Dyspnée laryngée☐))
Réintubation pour chirurgie avec intubation <24h OUI☐ NON ☐

A la fin de J33 le patient est :
Intubé ☐ Extubé ☐
Poursuite du protocole de sevrage ☐
Sortie du protocole de sevrage ☐ (remplir la partie « sortie d'essai »)
Poids du jour : |__|__|__| kg

J34 = |__|__|/|__|__/|__|__| de 0h à 23h59

Remplir si patient intubé le matin de J34

- Critères préalables de sevrabilité : Présents (-> Faire TVS) ☐ Absents☐
- TVS : Succès ☐ Echec ☐ (maintien intubation)
- Si succès :
  - Gaz du sang : pH |__|,|__|__|. pCO2 |__|__|__|mmHg pO2 |__|__|__|mmHg FiO2|__|__|__|%
  - Score de toux : 0 ☐ 1 ☐ 2 ☐ 3 ☐ 4 ☐ 5 ☐
  - Score d'encombrement : 0 ☐ 1 ☐ 2 ☐ 3 ☐ 4 ☐
  - Patients du groupe A (assisté) : TVS-TT Succès ☐ Echec ☐ (-> VNI prophylactique)
- Evaluer extubabilité si succès du TVS :
  - Force de toux acceptable (≥3) : Oui ☐ Non ☐
  - Encombrement acceptable (≤2) : Oui ☐ Non ☐
  - Absence d'AG dans les 24h qui suivent : Oui ☐ Non ☐
  - Pas d'argument pour une obstruction laryngée : Oui ☐ Non ☐

➔ Si ≥3 critères « oui » : extubation proposée :

➔ Heure d'extubation (hh :mm): |__|__|:|__|__|

Et raison de la non-extubation le cas échéant : ______________________________

Remplir si le patient n'est plus intubé à un moment de J34 (même quelques minutes ou depuis plusieurs jours)

Patient extubé AUJOURD'HUI OUI ☐ NON ☐

Sinon Date de la dernière extubation |__|__|/|__|__|/|__|__| Heure |__|__|:|__|__|

- Extubation (si réalisée AUJOURD'HUI)
  - programmée☐ (selon protocole☐ violation protocole☐)
  - auto-extubation ☐
- VNI prophylactique post-extubation OUI ☐ NON ☐
  - *Si VNI prophylactique*
  - selon protocole ☐ (TVS-TT (-)☐ âge>65ans☐ BPCO☐ Insuffisance cardiaque chronique ☐ Insuffisance respiratoire chronique☐, PaCO2>45 en fin de TVS-TT☐) violation protocole ☐
  - Durée cumulée sur les 24h : |__|__|h
  - *Si absence de VNI prophylactique*
  - selon protocole ☐ violation protocole ☐ contre-indication ☐
- VNI curative post-extubation NON ☐ selon protocole ☐ violation protocole ☐
- Optiflow post-extubation (non recommandé) OUI ☐ NON ☐
- Désencombrement bronchique : NON ☐ Toux manuellement assistée ☐
  Toux assistée instrumentale ☐

Réintubation ce jour : OUI ☐ NON ☐ : si OUI Heure : |__|__|:|__|__|
(critère(s) Neurologique☐ Hémodynamique ☐ autre ☐
Respiratoire ☐ (épuisement ☐ Encombrement ☐ Pneumopathie☐ OAP☐ Dyspnée laryngée☐))
Réintubation pour chirurgie avec intubation <24h OUI☐ NON ☐

A la fin de J34 le patient est :
Intubé ☐ Extubé ☐
Poursuite du protocole de sevrage ☐
Sortie du protocole de sevrage ☐ (remplir la partie « sortie d'essai »)
Poids du jour : |__|__|__| kg

J35 = |__|__|/|__|__/|__|__| de 0h à 23h59

Remplir si patient intubé le matin de J35

- Critères préalables de sevrabilité : Présents (-> Faire TVS) ☐ Absents☐
- TVS : Succès ☐ Echec ☐ (maintien intubation)
- Si succès :
  - Gaz du sang : pH |__|,|__|__|. pCO2 |__|__|__|mmHg pO2 |__|__|__|mmHg FiO2|__|__|__|%
  - Score de toux : 0 ☐ 1 ☐ 2 ☐ 3 ☐ 4 ☐ 5 ☐
  - Score d'encombrement : 0 ☐ 1 ☐ 2 ☐ 3 ☐ 4 ☐
  - Patients du groupe A (assisté) : TVS-TT Succès ☐ Echec ☐ (-> VNI prophylactique)
- Evaluer extubabilité si succès du TVS :
  - Force de toux acceptable (≥3) : Oui ☐ Non ☐
  - Encombrement acceptable (≤2) : Oui ☐ Non ☐
  - Absence d'AG dans les 24h qui suivent : Oui ☐ Non ☐
  - Pas d'argument pour une obstruction laryngée : Oui ☐ Non ☐

➔ Si ≥3 critères « oui » : extubation proposée :

➔ Heure d'extubation (hh :mm): |__|__|:|__|__|

Et raison de la non-extubation le cas échéant : ____________________________

Remplir si le patient n'est plus intubé à un moment de J35 (même quelques minutes ou depuis plusieurs jours)

Patient extubé AUJOURD'HUI OUI ☐ NON ☐

Sinon Date de la dernière extubation |__|__|/|__|__|/|__|__| Heure |__|__|:|__|__|

- Extubation (si réalisée AUJOURD'HUI)
  - programmée☐ (selon protocole☐ violation protocole☐)
  - auto-extubation ☐
- VNI prophylactique post-extubation OUI ☐ NON ☐
  - *Si VNI prophylactique*
  - selon protocole ☐ (TVS-TT (-)☐ âge>65ans☐ BPCO☐ Insuffisance cardiaque chronique ☐ Insuffisance respiratoire chronique☐, PaCO2>45 en fin de TVS-TT☐) violation protocole ☐
  - Durée cumulée sur les 24h : |__|__|h
  - *Si absence de VNI prophylactique*
  - selon protocole ☐ violation protocole ☐ contre-indication ☐
- VNI curative post-extubation NON ☐ selon protocole ☐ violation protocole ☐
- Optiflow post-extubation (non recommandé) OUI ☐ NON ☐
- Désencombrement bronchique : NON ☐ Toux manuellement assistée ☐
  Toux assistée instrumentale ☐

Réintubation ce jour : OUI ☐ NON ☐ : si OUI Heure : |__|__|:|__|__|
(critère(s) Neurologique☐ Hémodynamique ☐ autre ☐
Respiratoire ☐ (épuisement ☐ Encombrement ☐ Pneumopathie☐ OAP☐ Dyspnée laryngée☐))
Réintubation pour chirurgie avec intubation <24h OUI☐ NON ☐

A la fin de J35 le patient est :
Intubé ☐ Extubé ☐
Poursuite du protocole de sevrage ☐
Sortie du protocole de sevrage ☐ (remplir la partie « sortie d'essai »)
Poids du jour : |__|__|__|kg

J36 = |__|__|/|__|__/|__|__| de 0h à 23h59

Remplir si patient intubé le matin de J36

- Critères préalables de sevrabilité : Présents (-> Faire TVS) ☐ Absents☐
- TVS : Succès ☐ Echec ☐ (maintien intubation)
- Si succès :
  - Gaz du sang : pH |__|,|__|__|. pCO2 |__|__|__|mmHg pO2 |__|__|__|mmHg FiO2|__|__|__|%
  - Score de toux : 0 ☐ 1 ☐ 2 ☐ 3 ☐ 4 ☐ 5 ☐
  - Score d'encombrement : 0 ☐ 1 ☐ 2 ☐ 3 ☐ 4 ☐
  - Patients du groupe A (assisté) : TVS-TT Succès ☐ Echec ☐ (-> VNI prophylactique)
- Evaluer extubabilité si succès du TVS :
  - Force de toux acceptable (≥3) : Oui ☐ Non ☐
  - Encombrement acceptable (≤2) : Oui ☐ Non ☐
  - Absence d'AG dans les 24h qui suivent : Oui ☐ Non ☐
  - Pas d'argument pour une obstruction laryngée : Oui ☐ Non ☐

➔ Si ≥3 critères « oui » : extubation proposée :

➔ Heure d'extubation (hh :mm): |__|__|:|__|__|

Et raison de la non-extubation le cas échéant : ____________________________

Remplir si le patient n'est plus intubé à un moment de J36 (même quelques minutes ou depuis plusieurs jours)

Patient extubé AUJOURD'HUI OUI ☐ NON ☐

Sinon Date de la dernière extubation |__|__|/|__|__|/|__|__| Heure |__|__|:|__|__|

- Extubation (si réalisée AUJOURD'HUI)
  - programmée☐ (selon protocole☐ violation protocole☐)
  - auto-extubation ☐
- VNI prophylactique post-extubation OUI ☐ NON ☐
  - *Si VNI prophylactique*
  - selon protocole ☐ (TVS-TT (-)☐ âge>65ans☐ BPCO☐ Insuffisance cardiaque chronique ☐ Insuffisance respiratoire chronique☐, PaCO2>45 en fin de TVS-TT☐) violation protocole ☐
  - Durée cumulée sur les 24h : |__|__|h
  - *Si absence de VNI prophylactique*
  - selon protocole ☐ violation protocole ☐ contre-indication ☐
- VNI curative post-extubation NON ☐ selon protocole ☐ violation protocole ☐
- Optiflow post-extubation (non recommandé) OUI ☐ NON ☐
- Désencombrement bronchique : NON ☐ Toux manuellement assistée ☐
  Toux assistée instrumentale ☐

Réintubation ce jour : OUI ☐ NON ☐ : si OUI Heure : |__|__|:|__|__|

(critère(s) Neurologique☐ Hémodynamique ☐ autre ☐

Respiratoire ☐ (épuisement ☐ Encombrement ☐ Pneumopathie☐ OAP☐ Dyspnée laryngée☐))

Réintubation pour chirurgie avec intubation <24h OUI☐ NON ☐

A la fin de J36 le patient est :

Intubé ☐ Extubé ☐

Poursuite du protocole de sevrage ☐

Sortie du protocole de sevrage ☐ (remplir la partie « sortie d'essai »)

Poids du jour : |__|__|__| kg

J37 = |__|__|/|__|__/|__|__| de 0h à 23h59

Remplir si patient intubé le matin de J37

- Critères préalables de sevrabilité : Présents (-> Faire TVS) ☐ Absents☐
- TVS : Succès ☐ Echec ☐ (maintien intubation)
- Si succès :
  - Gaz du sang : pH |__|,|__|__|. pCO2 |__|__|__|mmHg pO2 |__|__|__|mmHg FiO2|__|__|__|%
  - Score de toux : 0 ☐ 1 ☐ 2 ☐ 3 ☐ 4 ☐ 5 ☐
  - Score d'encombrement : 0 ☐ 1 ☐ 2 ☐ 3 ☐ 4 ☐
  - Patients du groupe A (assisté) : TVS-TT Succès ☐ Echec ☐ (-> VNI prophylactique)
- Evaluer extubabilité si succès du TVS :
  - Force de toux acceptable (≥3) : Oui ☐ Non ☐
  - Encombrement acceptable (≤2) : Oui ☐ Non ☐
  - Absence d'AG dans les 24h qui suivent : Oui ☐ Non ☐
  - Pas d'argument pour une obstruction laryngée : Oui ☐ Non ☐

➔ Si ≥3 critères « oui » : extubation proposée :

➔ Heure d'extubation (hh :mm): |__|__|:|__|__|

Et raison de la non-extubation le cas échéant : ______________________________

Remplir si le patient n'est plus intubé à un moment de J37 (même quelques minutes ou depuis plusieurs jours)

Patient extubé AUJOURD'HUI OUI ☐ NON ☐

Sinon Date de la dernière extubation |__|__|/|__|__|/|__|__| Heure |__|__|:|__|__|

- Extubation (si réalisée AUJOURD'HUI)
  - programmée☐ (selon protocole☐ violation protocole☐)
  - auto-extubation ☐
- VNI prophylactique post-extubation OUI ☐ NON ☐
  - *Si VNI prophylactique*
  - selon protocole ☐ (TVS-TT (-)☐ âge>65ans☐ BPCO☐ Insuffisance cardiaque chronique ☐ Insuffisance respiratoire chronique☐, PaCO2>45 en fin de TVS-TT☐) violation protocole ☐
  - Durée cumulée sur les 24h : |__|__|h
  - *Si absence de VNI prophylactique*
  - selon protocole ☐ violation protocole ☐ contre-indication ☐
- VNI curative post-extubation NON ☐ selon protocole ☐ violation protocole ☐
- Optiflow post-extubation (non recommandé) OUI ☐ NON ☐
- Désencombrement bronchique : NON ☐ Toux manuellement assistée ☐ Toux assistée instrumentale ☐

Réintubation ce jour : OUI ☐ NON ☐ : si OUI Heure : |__|__|:|__|__|

(critère(s) Neurologique☐ Hémodynamique ☐ autre ☐

Respiratoire ☐ (épuisement ☐ Encombrement ☐ Pneumopathie☐ OAP☐ Dyspnée laryngée☐))

Réintubation pour chirurgie avec intubation <24h OUI☐ NON ☐

A la fin de J37 le patient est :

Intubé ☐ Extubé ☐

Poursuite du protocole de sevrage ☐

Sortie du protocole de sevrage ☐ (remplir la partie « sortie d'essai »)

Poids du jour : |__|__|__| kg

J38 = |__|__|/|__|__/|__|__| de 0h à 23h59

Remplir si patient intubé le matin de J38

- Critères préalables de sevrabilité : Présents (-> Faire TVS) ☐ Absents☐
- TVS : Succès ☐ Echec ☐ (maintien intubation)
- Si succès :
  - Gaz du sang : pH |__|,|__|__|. pCO2 |__|__|__|mmHg pO2 |__|__|__|mmHg FiO2|__|__|__|%
  - Score de toux : 0 ☐ 1 ☐ 2 ☐ 3 ☐ 4 ☐ 5 ☐
  - Score d'encombrement : 0 ☐ 1 ☐ 2 ☐ 3 ☐ 4 ☐
  - Patients du groupe A (assisté) : TVS-TT Succès ☐ Echec ☐ (-> VNI prophylactique)
- Evaluer extubabilité si succès du TVS :
  - Force de toux acceptable (≥3) : Oui ☐ Non ☐
  - Encombrement acceptable (≤2) : Oui ☐ Non ☐
  - Absence d'AG dans les 24h qui suivent : Oui ☐ Non ☐
  - Pas d'argument pour une obstruction laryngée : Oui ☐ Non ☐

➔ Si ≥3 critères « oui » : extubation proposée :

➔ Heure d'extubation (hh :mm): |__|__|:|__|__|

Et raison de la non-extubation le cas échéant : ______________________________

Remplir si le patient n'est plus intubé à un moment de J38 (même quelques minutes ou depuis plusieurs jours)

Patient extubé AUJOURD'HUI OUI ☐ NON ☐

Sinon Date de la dernière extubation |__|__|/|__|__|/|__|__| Heure |__|__|:|__|__|

- Extubation (si réalisée AUJOURD'HUI)
  - programmée☐ (selon protocole☐ violation protocole☐)
  - auto-extubation ☐
- VNI prophylactique post-extubation OUI ☐ NON ☐
  - *Si VNI prophylactique*
  - selon protocole ☐ (TVS-TT (-)☐ âge>65ans☐ BPCO☐ Insuffisance cardiaque chronique ☐ Insuffisance respiratoire chronique☐, PaCO2>45 en fin de TVS-TT☐) violation protocole ☐
  - Durée cumulée sur les 24h : |__|__|h
  - *Si absence de VNI prophylactique*
  - selon protocole ☐ violation protocole ☐ contre-indication ☐
- VNI curative post-extubation NON ☐ selon protocole ☐ violation protocole ☐
- Optiflow post-extubation (non recommandé) OUI ☐ NON ☐
- Désencombrement bronchique : NON ☐ Toux manuellement assistée ☐
  Toux assistée instrumentale ☐

Réintubation ce jour : OUI ☐ NON ☐ : si OUI Heure : |__|__|:|__|__|

(critère(s) Neurologique☐ Hémodynamique ☐ autre ☐

Respiratoire ☐ (épuisement ☐ Encombrement ☐ Pneumopathie☐ OAP☐ Dyspnée laryngée☐))

Réintubation pour chirurgie avec intubation <24h OUI☐ NON ☐

A la fin de J38 le patient est :

Intubé ☐ Extubé ☐

Poursuite du protocole de sevrage ☐

Sortie du protocole de sevrage ☐ (remplir la partie « sortie d'essai »)

Poids du jour : |__|__|__|kg

J39 = |__|__|/|__|__/|__|__| de 0h à 23h59

Remplir si patient intubé le matin de J39

- Critères préalables de sevrabilité : Présents (-> Faire TVS) ☐ Absents☐
- TVS : Succès ☐ Echec ☐ (maintien intubation)
- Si succès :
  - Gaz du sang : pH |__|,|__|__|. pCO2 |__|__|__|mmHg pO2 |__|__|__|mmHg FiO2|__|__|__|%
  - Score de toux : 0 ☐ 1 ☐ 2 ☐ 3 ☐ 4 ☐ 5 ☐
  - Score d'encombrement : 0 ☐ 1 ☐ 2 ☐ 3 ☐ 4 ☐
  - Patients du groupe A (assisté) : TVS-TT Succès ☐ Echec ☐ (-> VNI prophylactique)
- Evaluer extubabilité si succès du TVS :
  - Force de toux acceptable (≥3) : Oui ☐ Non ☐
  - Encombrement acceptable (≤2) : Oui ☐ Non ☐
  - Absence d'AG dans les 24h qui suivent : Oui ☐ Non ☐
  - Pas d'argument pour une obstruction laryngée : Oui ☐ Non ☐

➔ Si ≥3 critères « oui » : extubation proposée :

➔ Heure d'extubation (hh :mm): |__|__|:|__|__|

Et raison de la non-extubation le cas échéant : ______________________________

Remplir si le patient n'est plus intubé à un moment de J39 (même quelques minutes ou depuis plusieurs jours)

Patient extubé AUJOURD'HUI OUI ☐ NON ☐

Sinon Date de la dernière extubation |__|__|/|__|__|/|__|__| Heure |__|__|:|__|__|

- Extubation (si réalisée AUJOURD'HUI)
  - programmée☐ (selon protocole☐ violation protocole☐)
  - auto-extubation ☐
- VNI prophylactique post-extubation OUI ☐ NON ☐
  - *Si VNI prophylactique*
  - selon protocole ☐ (TVS-TT (-)☐ âge>65ans☐ BPCO☐ Insuffisance cardiaque chronique ☐ Insuffisance respiratoire chronique☐, PaCO2>45 en fin de TVS-TT☐) violation protocole ☐
  - Durée cumulée sur les 24h : |__|__|h
  - *Si absence de VNI prophylactique*
  - selon protocole ☐ violation protocole ☐ contre-indication ☐
- VNI curative post-extubation NON ☐ selon protocole ☐ violation protocole ☐
- Optiflow post-extubation (non recommandé) OUI ☐ NON ☐
- Désencombrement bronchique : NON ☐ Toux manuellement assistée ☐
  Toux assistée instrumentale ☐

Réintubation ce jour : OUI ☐ NON ☐ : si OUI Heure : |__|__|:|__|__|
(critère(s) Neurologique☐ Hémodynamique ☐ autre ☐
Respiratoire ☐ (épuisement ☐ Encombrement ☐ Pneumopathie☐ OAP☐ Dyspnée laryngée☐))
Réintubation pour chirurgie avec intubation <24h OUI☐ NON ☐

A la fin de J39 le patient est :
Intubé ☐ Extubé ☐
Poursuite du protocole de sevrage ☐
Sortie du protocole de sevrage ☐ (remplir la partie « sortie d'essai »)
Poids du jour : |__|__|__| kg

J40 = |__|__|/|__|__/|__|__| de 0h à 23h59

Remplir si patient intubé le matin de J40

- Critères préalables de sevrabilité : Présents (-> Faire TVS) ☐ Absents ☐
- TVS : Succès ☐ Echec ☐ (maintien intubation)
- Si succès :
  - Gaz du sang : pH |__|,|__|__|. pCO2 |__|__|__|mmHg pO2 |__|__|__|mmHg FiO2|__|__|__|%
  - Score de toux : 0 ☐ 1 ☐ 2 ☐ 3 ☐ 4 ☐ 5 ☐
  - Score d'encombrement : 0 ☐ 1 ☐ 2 ☐ 3 ☐ 4 ☐
  - Patients du groupe A (assisté) : TVS-TT Succès ☐ Echec ☐ (-> VNI prophylactique)
- Evaluer extubabilité si succès du TVS :
  - Force de toux acceptable (≥3) : Oui ☐ Non ☐
  - Encombrement acceptable (≤2) : Oui ☐ Non ☐
  - Absence d'AG dans les 24h qui suivent : Oui ☐ Non ☐
  - Pas d'argument pour une obstruction laryngée : Oui ☐ Non ☐

➔ Si ≥3 critères « oui » : extubation proposée :

➔ Heure d'extubation (hh :mm): |__|__|:|__|__|

Et raison de la non-extubation le cas échéant : ____________________________

Remplir si le patient n'est plus intubé à un moment de J40 (même quelques minutes ou depuis plusieurs jours)

Patient extubé AUJOURD'HUI OUI ☐ NON ☐

Sinon Date de la dernière extubation |__|__|/|__|__|/|__|__| Heure |__|__|:|__|__|

- Extubation (si réalisée AUJOURD'HUI)
  - programmée ☐ (selon protocole ☐ violation protocole ☐)
  - auto-extubation ☐
- VNI prophylactique post-extubation OUI ☐ NON ☐
  - *Si VNI prophylactique*
  - selon protocole ☐ (TVS-TT (-) ☐ âge>65ans ☐ BPCO ☐ Insuffisance cardiaque chronique ☐ Insuffisance respiratoire chronique ☐, PaCO2>45 en fin de TVS-TT ☐) violation protocole ☐
  - Durée cumulée sur les 24h : |__|__|h
  - *Si absence de VNI prophylactique*
  - selon protocole ☐ violation protocole ☐ contre-indication ☐
- VNI curative post-extubation NON ☐ selon protocole ☐ violation protocole ☐
- Optiflow post-extubation (non recommandé) OUI ☐ NON ☐
- Désencombrement bronchique : NON ☐ Toux manuellement assistée ☐
  Toux assistée instrumentale ☐

Réintubation ce jour : OUI ☐ NON ☐ : si OUI Heure : |__|__|:|__|__|
(critère(s) Neurologique ☐ Hémodynamique ☐ autre ☐
Respiratoire ☐ (épuisement ☐ Encombrement ☐ Pneumopathie ☐ OAP ☐ Dyspnée laryngée ☐))
Réintubation pour chirurgie avec intubation <24h OUI ☐ NON ☐

A la fin de J40 le patient est :
Intubé ☐ Extubé ☐
Poursuite du protocole de sevrage ☐
Sortie du protocole de sevrage ☐ (remplir la partie « sortie d'essai »)
Poids du jour : |__|__|__| kg

J41 = |__|__|/|__|__/|__|__| de 0h à 23h59

Remplir si patient intubé le matin de J41

- Critères préalables de sevrabilité : Présents (-> Faire TVS) ☐ Absents☐
- TVS : Succès ☐ Echec ☐ (maintien intubation)
- Si succès :
  - Gaz du sang : pH |__|,|__|__|. pCO2 |__|__|__|mmHg pO2 |__|__|__|mmHg FiO2|__|__|__|%
  - Score de toux : 0 ☐ 1 ☐ 2 ☐ 3 ☐ 4 ☐ 5 ☐
  - Score d'encombrement : 0 ☐ 1 ☐ 2 ☐ 3 ☐ 4 ☐
  - Patients du groupe A (assisté) : TVS-TT Succès ☐ Echec ☐ (-> VNI prophylactique)
- Evaluer extubabilité si succès du TVS :
  - Force de toux acceptable (≥3) : Oui ☐ Non ☐
  - Encombrement acceptable (≤2) : Oui ☐ Non ☐
  - Absence d'AG dans les 24h qui suivent : Oui ☐ Non ☐
  - Pas d'argument pour une obstruction laryngée : Oui ☐ Non ☐

➔ Si ≥3 critères « oui » : extubation proposée :

➔ Heure d'extubation (hh :mm): |__|__|:|__|__|

Et raison de la non-extubation le cas échéant : ____________________________

Remplir si le patient n'est plus intubé à un moment de J41 (même quelques minutes ou depuis plusieurs jours)

Patient extubé AUJOURD'HUI OUI ☐ NON ☐

Sinon Date de la dernière extubation |__|__|/|__|__|/|__|__| Heure |__|__|:|__|__|

- Extubation (si réalisée AUJOURD'HUI)
  - programmée☐ (selon protocole☐ violation protocole☐)
  - auto-extubation ☐
- VNI prophylactique post-extubation OUI ☐ NON ☐
  - *Si VNI prophylactique*
  - selon protocole ☐ (TVS-TT (-)☐ âge>65ans☐ BPCO☐ Insuffisance cardiaque chronique ☐ Insuffisance respiratoire chronique☐, PaCO2>45 en fin de TVS-TT☐) violation protocole ☐
  - Durée cumulée sur les 24h : |__|__|h
  - *Si absence de VNI prophylactique*
  - selon protocole ☐ violation protocole ☐ contre-indication ☐
- VNI curative post-extubation NON ☐ selon protocole ☐ violation protocole ☐
- Optiflow post-extubation (non recommandé) OUI ☐ NON ☐
- Désencombrement bronchique : NON ☐ Toux manuellement assistée ☐ Toux assistée instrumentale ☐

Réintubation ce jour : OUI ☐ NON ☐ : si OUI Heure : |__|__|:|__|__|

(critère(s) Neurologique☐ Hémodynamique ☐ autre ☐

Respiratoire ☐ (épuisement ☐ Encombrement ☐ Pneumopathie☐ OAP☐ Dyspnée laryngée☐))

Réintubation pour chirurgie avec intubation <24h OUI☐ NON ☐

A la fin de J41 le patient est :

Intubé ☐ Extubé ☐

Poursuite du protocole de sevrage ☐

Sortie du protocole de sevrage ☐ (remplir la partie « sortie d'essai »)

Poids du jour : |__|__|__| kg

J42 = |__|__|/|__|__/|__|__| de 0h à 23h59

Remplir si patient intubé le matin de J42

- Critères préalables de sevrabilité : Présents (-> Faire TVS) ☐ Absents☐
- TVS : Succès ☐ Echec ☐ (maintien intubation)
- Si succès :
  - Gaz du sang : pH |__|,|__|__|. pCO2 |__|__|__|mmHg pO2 |__|__|__|mmHg FiO2|__|__|__|%
  - Score de toux : 0 ☐ 1 ☐ 2 ☐ 3 ☐ 4 ☐ 5 ☐
  - Score d'encombrement : 0 ☐ 1 ☐ 2 ☐ 3 ☐ 4 ☐
  - Patients du groupe A (assisté) : TVS-TT Succès ☐ Echec ☐ (-> VNI prophylactique)
- Evaluer extubabilité si succès du TVS :
  - Force de toux acceptable (≥3) : Oui ☐ Non ☐
  - Encombrement acceptable (≤2) : Oui ☐ Non ☐
  - Absence d'AG dans les 24h qui suivent : Oui ☐ Non ☐
  - Pas d'argument pour une obstruction laryngée : Oui ☐ Non ☐

➔ Si ≥3 critères « oui » : extubation proposée :

➔ Heure d'extubation (hh :mm): |__|__|:|__|__|

Et raison de la non-extubation le cas échéant : ______________________________

Remplir si le patient n'est plus intubé à un moment de J42 (même quelques minutes ou depuis plusieurs jours)

Patient extubé AUJOURD'HUI OUI ☐ NON ☐

Sinon Date de la dernière extubation |__|__|/|__|__|/|__|__| Heure |__|__|:|__|__|

- Extubation (si réalisée AUJOURD'HUI)
  - programmée☐ (selon protocole☐ violation protocole☐)
  - auto-extubation ☐
- VNI prophylactique post-extubation OUI ☐ NON ☐
  - *Si VNI prophylactique*
  - selon protocole ☐ (TVS-TT (-)☐ âge>65ans☐ BPCO☐ Insuffisance cardiaque chronique ☐ Insuffisance respiratoire chronique☐, PaCO2>45 en fin de TVS-TT☐) violation protocole ☐
  - Durée cumulée sur les 24h : |__|__|h
  - *Si absence de VNI prophylactique*
  - selon protocole ☐ violation protocole ☐ contre-indication ☐
- VNI curative post-extubation NON ☐ selon protocole ☐ violation protocole ☐
- Optiflow post-extubation (non recommandé) OUI ☐ NON ☐
- Désencombrement bronchique : NON ☐ Toux manuellement assistée ☐
  Toux assistée instrumentale ☐

Réintubation ce jour : OUI ☐ NON ☐ : si OUI Heure : |__|__|:|__|__|

(critère(s) Neurologique☐ Hémodynamique ☐ autre ☐

Respiratoire ☐ (épuisement ☐ Encombrement ☐ Pneumopathie☐ OAP☐ Dyspnée laryngée☐))

Réintubation pour chirurgie avec intubation <24h OUI☐ NON ☐

A la fin de J42 le patient est :

Intubé ☐ Extubé ☐

Poursuite du protocole de sevrage ☐

Sortie du protocole de sevrage ☐ (remplir la partie « sortie d'essai »)

Poids du jour : |__|__|__| kg

J43 = |__|__|/|__|__/|__|__| de 0h à 23h59

Remplir si patient intubé le matin de J43

- Critères préalables de sevrabilité : Présents (-> Faire TVS) ☐ Absents☐
- TVS : Succès ☐ Echec ☐ (maintien intubation)
- Si succès :
  - Gaz du sang : pH |__|,|__|__|. pCO2 |__|__|__|mmHg pO2 |__|__|__|mmHg FiO2|__|__|__|%
  - Score de toux : 0 ☐ 1 ☐ 2 ☐ 3 ☐ 4 ☐ 5 ☐
  - Score d'encombrement : 0 ☐ 1 ☐ 2 ☐ 3 ☐ 4 ☐
  - Patients du groupe A (assisté) : TVS-TT Succès ☐ Echec ☐ (-> VNI prophylactique)
- Evaluer extubabilité si succès du TVS :
  - Force de toux acceptable (≥3) : Oui ☐ Non ☐
  - Encombrement acceptable (≤2) : Oui ☐ Non ☐
  - Absence d'AG dans les 24h qui suivent : Oui ☐ Non ☐
  - Pas d'argument pour une obstruction laryngée : Oui ☐ Non ☐

➔ Si ≥3 critères « oui » : extubation proposée :

➔ Heure d'extubation (hh :mm): |__|__|:|__|__|

Et raison de la non-extubation le cas échéant : ______________________________

Remplir si le patient n'est plus intubé à un moment de J43 (même quelques minutes ou depuis plusieurs jours)

Patient extubé AUJOURD'HUI OUI ☐ NON ☐

Sinon Date de la dernière extubation |__|__|/|__|__|/|__|__| Heure |__|__|:|__|__|

- Extubation (si réalisée AUJOURD'HUI)
  - programmée☐ (selon protocole☐ violation protocole☐)
  - auto-extubation ☐
- VNI prophylactique post-extubation OUI ☐ NON ☐
  - *Si VNI prophylactique*
  - selon protocole ☐ (TVS-TT (-)☐ âge>65ans☐ BPCO☐ Insuffisance cardiaque chronique ☐ Insuffisance respiratoire chronique☐, PaCO2>45 en fin de TVS-TT☐) violation protocole ☐
  - Durée cumulée sur les 24h : |__|__|h
  - *Si absence de VNI prophylactique*
  - selon protocole ☐ violation protocole ☐ contre-indication ☐
- VNI curative post-extubation NON ☐ selon protocole ☐ violation protocole ☐
- Optiflow post-extubation (non recommandé) OUI ☐ NON ☐
- Désencombrement bronchique : NON ☐ Toux manuellement assistée ☐
  Toux assistée instrumentale ☐

Réintubation ce jour : OUI ☐ NON ☐ : si OUI Heure : |__|__|:|__|__|
(critère(s) Neurologique☐ Hémodynamique ☐ autre ☐
Respiratoire ☐ (épuisement ☐ Encombrement ☐ Pneumopathie☐ OAP☐ Dyspnée laryngée☐))
Réintubation pour chirurgie avec intubation <24h OUI☐ NON ☐

A la fin de J43 le patient est :
Intubé ☐ Extubé ☐
Poursuite du protocole de sevrage ☐
Sortie du protocole de sevrage ☐ (remplir la partie « sortie d'essai »)
Poids du jour : |__|__|__| kg

J44 = |__|__|/|__|__/|__|__| de 0h à 23h59

Remplir si patient intubé le matin de J44

- Critères préalables de sevrabilité : Présents (-> Faire TVS) ☐ Absents☐
- TVS : Succès ☐ Echec ☐ (maintien intubation)
- Si succès :
  - Gaz du sang : pH |__|,|__|__|. pCO2 |__|__|__|mmHg pO2 |__|__|__|mmHg FiO2|__|__|__|%
  - Score de toux : 0 ☐ 1 ☐ 2 ☐ 3 ☐ 4 ☐ 5 ☐
  - Score d'encombrement : 0 ☐ 1 ☐ 2 ☐ 3 ☐ 4 ☐
  - Patients du groupe A (assisté) : TVS-TT Succès ☐ Echec ☐ (-> VNI prophylactique)
- Evaluer extubabilité si succès du TVS :
  - Force de toux acceptable (≥3) : Oui ☐ Non ☐
  - Encombrement acceptable (≤2) : Oui ☐ Non ☐
  - Absence d'AG dans les 24h qui suivent : Oui ☐ Non ☐
  - Pas d'argument pour une obstruction laryngée : Oui ☐ Non ☐

➔ Si ≥3 critères « oui » : extubation proposée :
➔ Heure d'extubation (hh :mm): |__|__|:|__|__|

Et raison de la non-extubation le cas échéant : ____________________________

Remplir si le patient n'est plus intubé à un moment de J44 (même quelques minutes ou depuis plusieurs jours)

Patient extubé AUJOURD'HUI OUI ☐ NON ☐
Sinon Date de la dernière extubation |__|__|/|__|__|/|__|__| Heure |__|__|:|__|__|

- Extubation (si réalisée AUJOURD'HUI)
  - programmée☐ (selon protocole☐ violation protocole☐)
  - auto-extubation ☐
- VNI prophylactique post-extubation OUI ☐ NON ☐
  - *Si VNI prophylactique*
  - selon protocole ☐ (TVS-TT (-)☐ âge>65ans☐ BPCO☐ Insuffisance cardiaque chronique ☐ Insuffisance respiratoire chronique☐, PaCO2>45 en fin de TVS-TT☐) violation protocole ☐
  - Durée cumulée sur les 24h : |__|__|h
  - *Si absence de VNI prophylactique*
  - selon protocole ☐ violation protocole ☐ contre-indication ☐
- VNI curative post-extubation NON ☐ selon protocole ☐ violation protocole ☐
- Optiflow post-extubation (non recommandé) OUI ☐ NON ☐
- Désencombrement bronchique : NON ☐ Toux manuellement assistée ☐ Toux assistée instrumentale ☐

Réintubation ce jour : OUI ☐ NON ☐ : si OUI Heure : |__|__|:|__|__|
(critère(s) Neurologique☐ Hémodynamique ☐ autre ☐
Respiratoire ☐ (épuisement ☐ Encombrement ☐ Pneumopathie☐ OAP☐ Dyspnée laryngée☐))
Réintubation pour chirurgie avec intubation <24h OUI☐ NON ☐

A la fin de J44 le patient est :
Intubé ☐ Extubé ☐
Poursuite du protocole de sevrage ☐
Sortie du protocole de sevrage ☐ (remplir la partie « sortie d'essai »)
Poids du jour : |__|__|__| kg

J45 = |__|__|/|__|__/|__|__| de 0h à 23h59

Remplir si patient intubé le matin de J45

- Critères préalables de sevrabilité : Présents (-> Faire TVS) ☐ Absents☐
- TVS : Succès ☐ Echec ☐ (maintien intubation)
- Si succès :
  - Gaz du sang : pH |__|,|__|__|. pCO2 |__|__|__|mmHg pO2 |__|__|__|mmHg FiO2|__|__|__|%
  - Score de toux : 0 ☐ 1 ☐ 2 ☐ 3 ☐ 4 ☐ 5 ☐
  - Score d'encombrement : 0 ☐ 1 ☐ 2 ☐ 3 ☐ 4 ☐
  - Patients du groupe A (assisté) : TVS-TT Succès ☐ Echec ☐ (-> VNI prophylactique)
- Evaluer extubabilité si succès du TVS :
  - Force de toux acceptable (≥3) : Oui ☐ Non ☐
  - Encombrement acceptable (≤2) : Oui ☐ Non ☐
  - Absence d'AG dans les 24h qui suivent : Oui ☐ Non ☐
  - Pas d'argument pour une obstruction laryngée : Oui ☐ Non ☐

➔ Si ≥3 critères « oui » : extubation proposée :

➔ Heure d'extubation (hh :mm): |__|__|:|__|__|

Et raison de la non-extubation le cas échéant : ____________________________

Remplir si le patient n'est plus intubé à un moment de J45 (même quelques minutes ou depuis plusieurs jours)

Patient extubé AUJOURD'HUI OUI ☐ NON ☐

Sinon Date de la dernière extubation |__|__|/|__|__|/|__|__| Heure |__|__|:|__|__|

- Extubation (si réalisée AUJOURD'HUI)
  - programmée☐ (selon protocole☐ violation protocole☐)
  - auto-extubation ☐
- VNI prophylactique post-extubation OUI ☐ NON ☐
  - *Si VNI prophylactique*
  - selon protocole ☐ (TVS-TT (-)☐ âge>65ans☐ BPCO☐ Insuffisance cardiaque chronique ☐ Insuffisance respiratoire chronique☐, PaCO2>45 en fin de TVS-TT☐) violation protocole ☐
  - Durée cumulée sur les 24h : |__|__|h
  - *Si absence de VNI prophylactique*
  - selon protocole ☐ violation protocole ☐ contre-indication ☐
- VNI curative post-extubation NON ☐ selon protocole ☐ violation protocole ☐
- Optiflow post-extubation (non recommandé) OUI ☐ NON ☐
- Désencombrement bronchique : NON ☐ Toux manuellement assistée ☐ Toux assistée instrumentale ☐

Réintubation ce jour : OUI ☐ NON ☐ : si OUI Heure : |__|__|:|__|__|
(critère(s) Neurologique☐ Hémodynamique ☐ autre ☐
Respiratoire ☐ (épuisement ☐ Encombrement ☐ Pneumopathie☐ OAP☐ Dyspnée laryngée☐))
Réintubation pour chirurgie avec intubation <24h OUI☐ NON ☐

A la fin de J45 le patient est :
Intubé ☐ Extubé ☐
Poursuite du protocole de sevrage ☐
Sortie du protocole de sevrage ☐ (remplir la partie « sortie d'essai »)
Poids du jour : |__|__|__| kg

J46 = |__|__|/|__|__/|__|__| de 0h à 23h59

Remplir si patient intubé le matin de J46

- Critères préalables de sevrabilité : Présents (-> Faire TVS) ☐ Absents☐
- TVS : Succès ☐ Echec ☐ (maintien intubation)
- Si succès :
  - Gaz du sang : pH |__|,|__|__|. pCO2 |__|__|__|mmHg pO2 |__|__|__|mmHg FiO2|__|__|__|%
  - Score de toux : 0 ☐ 1 ☐ 2 ☐ 3 ☐ 4 ☐ 5 ☐
  - Score d'encombrement : 0 ☐ 1 ☐ 2 ☐ 3 ☐ 4 ☐
  - Patients du groupe A (assisté) : TVS-TT Succès ☐ Echec ☐ (-> VNI prophylactique)
- Evaluer extubabilité si succès du TVS :
  - Force de toux acceptable (≥3) : Oui ☐ Non ☐
  - Encombrement acceptable (≤2) : Oui ☐ Non ☐
  - Absence d'AG dans les 24h qui suivent : Oui ☐ Non ☐
  - Pas d'argument pour une obstruction laryngée : Oui ☐ Non ☐

➔ Si ≥3 critères « oui » : extubation proposée :

➔ Heure d'extubation (hh :mm): |__|__|:|__|__|

Et raison de la non-extubation le cas échéant : ____________________________

Remplir si le patient n'est plus intubé à un moment de J46 (même quelques minutes ou depuis plusieurs jours)

Patient extubé AUJOURD'HUI OUI ☐ NON ☐

Sinon Date de la dernière extubation |__|__|/|__|__|/|__|__| Heure |__|__|:|__|__|

- Extubation (si réalisée AUJOURD'HUI)
  - programmée☐ (selon protocole☐ violation protocole☐)
  - auto-extubation ☐
- VNI prophylactique post-extubation OUI ☐ NON ☐
  - *Si VNI prophylactique*
  - selon protocole ☐ (TVS-TT (-)☐ âge>65ans☐ BPCO☐ Insuffisance cardiaque chronique ☐ Insuffisance respiratoire chronique☐, PaCO2>45 en fin de TVS-TT☐) violation protocole ☐
  - Durée cumulée sur les 24h : |__|__|h
  - *Si absence de VNI prophylactique*
  - selon protocole ☐ violation protocole ☐ contre-indication ☐
- VNI curative post-extubation NON ☐ selon protocole ☐ violation protocole ☐
- Optiflow post-extubation (non recommandé) OUI ☐ NON ☐
- Désencombrement bronchique : NON ☐ Toux manuellement assistée ☐
  Toux assistée instrumentale ☐

Réintubation ce jour : OUI ☐ NON ☐ : si OUI Heure : |__|__|:|__|__|
(critère(s) Neurologique☐ Hémodynamique ☐ autre ☐
Respiratoire ☐ (épuisement ☐ Encombrement ☐ Pneumopathie☐ OAP☐ Dyspnée laryngée☐))
Réintubation pour chirurgie avec intubation <24h OUI☐ NON ☐

A la fin de J46 le patient est :
Intubé ☐ Extubé ☐
Poursuite du protocole de sevrage ☐
Sortie du protocole de sevrage ☐ (remplir la partie « sortie d'essai »)
Poids du jour : |__|__|__| kg

J47 = |__|__|/|__|__/|__|__| de 0h à 23h59

Remplir si patient intubé le matin de J47

- Critères préalables de sevrabilité : Présents (-> Faire TVS) ☐ Absents☐
- TVS : Succès ☐ Echec ☐ (maintien intubation)
- Si succès :
  - Gaz du sang : pH |__|,|__|__|. pCO2 |__|__|__|mmHg pO2 |__|__|__|mmHg FiO2|__|__|__|%
  - Score de toux : 0 ☐ 1 ☐ 2 ☐ 3 ☐ 4 ☐ 5 ☐
  - Score d'encombrement : 0 ☐ 1 ☐ 2 ☐ 3 ☐ 4 ☐
  - Patients du groupe A (assisté) : TVS-TT Succès ☐ Echec ☐ (-> VNI prophylactique)
- Evaluer extubabilité si succès du TVS :
  - Force de toux acceptable (≥3) : Oui ☐ Non ☐
  - Encombrement acceptable (≤2) : Oui ☐ Non ☐
  - Absence d'AG dans les 24h qui suivent : Oui ☐ Non ☐
  - Pas d'argument pour une obstruction laryngée : Oui ☐ Non ☐

➔ Si ≥3 critères « oui » : extubation proposée :

➔ Heure d'extubation (hh :mm): |__|__|:|__|__|

Et raison de la non-extubation le cas échéant : ______________________________

Remplir si le patient n'est plus intubé à un moment de J47 (même quelques minutes ou depuis plusieurs jours)

Patient extubé AUJOURD'HUI OUI ☐ NON ☐

Sinon Date de la dernière extubation |__|__|/|__|__|/|__|__| Heure |__|__|:|__|__|

- Extubation (si réalisée AUJOURD'HUI)
  - programmée☐ (selon protocole☐ violation protocole☐)
  - auto-extubation ☐
- VNI prophylactique post-extubation OUI ☐ NON ☐
  - *Si VNI prophylactique*
  - selon protocole ☐ (TVS-TT (-)☐ âge>65ans☐ BPCO☐ Insuffisance cardiaque chronique ☐ Insuffisance respiratoire chronique☐, PaCO2>45 en fin de TVS-TT☐) violation protocole ☐
  - Durée cumulée sur les 24h : |__|__|h
  - *Si absence de VNI prophylactique*
  - selon protocole ☐ violation protocole ☐ contre-indication ☐
- VNI curative post-extubation NON ☐ selon protocole ☐ violation protocole ☐
- Optiflow post-extubation (non recommandé) OUI ☐ NON ☐
- Désencombrement bronchique : NON ☐ Toux manuellement assistée ☐ Toux assistée instrumentale ☐

Réintubation ce jour : OUI ☐ NON ☐ : si OUI Heure : |__|__|:|__|__|

(critère(s) Neurologique☐ Hémodynamique ☐ autre ☐

Respiratoire ☐ (épuisement ☐ Encombrement ☐ Pneumopathie☐ OAP☐ Dyspnée laryngée☐))

Réintubation pour chirurgie avec intubation <24h OUI☐ NON ☐

A la fin de J47 le patient est :

Intubé ☐ Extubé ☐

Poursuite du protocole de sevrage ☐

Sortie du protocole de sevrage ☐ (remplir la partie « sortie d'essai »)

Poids du jour : |__|__|__| kg

J48 = |__|__|/|__|__/|__|__| de 0h à 23h59

Remplir si patient intubé le matin de J48

- Critères préalables de sevrabilité : Présents (-> Faire TVS) ☐ Absents☐
- TVS : Succès ☐ Echec ☐ (maintien intubation)
- Si succès :
  - Gaz du sang : pH |__|,|__|__|. pCO2 |__|__|__|mmHg pO2 |__|__|__|mmHg FiO2|__|__|__|%
  - Score de toux : 0 ☐ 1 ☐ 2 ☐ 3 ☐ 4 ☐ 5 ☐
  - Score d'encombrement : 0 ☐ 1 ☐ 2 ☐ 3 ☐ 4 ☐
  - Patients du groupe A (assisté) : TVS-TT Succès ☐ Echec ☐ (-> VNI prophylactique)
- Evaluer extubabilité si succès du TVS :
  - Force de toux acceptable (≥3) : Oui ☐ Non ☐
  - Encombrement acceptable (≤2) : Oui ☐ Non ☐
  - Absence d'AG dans les 24h qui suivent : Oui ☐ Non ☐
  - Pas d'argument pour une obstruction laryngée : Oui ☐ Non ☐

➔ Si ≥3 critères « oui » : extubation proposée :

➔ Heure d'extubation (hh :mm): |__|__|:|__|__|

Et raison de la non-extubation le cas échéant : ______________________________

Remplir si le patient n'est plus intubé à un moment de J48 (même quelques minutes ou depuis plusieurs jours)

Patient extubé AUJOURD'HUI OUI ☐ NON ☐

Sinon Date de la dernière extubation |__|__|/|__|__|/|__|__| Heure |__|__|:|__|__|

- Extubation (si réalisée AUJOURD'HUI)
  - programmée☐ (selon protocole☐ violation protocole☐)
  - auto-extubation ☐
- VNI prophylactique post-extubation OUI ☐ NON ☐
  - *Si VNI prophylactique*
  - selon protocole ☐ (TVS-TT (-)☐ âge>65ans☐ BPCO☐ Insuffisance cardiaque chronique ☐ Insuffisance respiratoire chronique☐, PaCO2>45 en fin de TVS-TT☐) violation protocole ☐
  - Durée cumulée sur les 24h : |__|__|h
  - *Si absence de VNI prophylactique*
  - selon protocole ☐ violation protocole ☐ contre-indication ☐
- VNI curative post-extubation NON ☐ selon protocole ☐ violation protocole ☐
- Optiflow post-extubation (non recommandé) OUI ☐ NON ☐
- Désencombrement bronchique : NON ☐ Toux manuellement assistée ☐
  Toux assistée instrumentale ☐

Réintubation ce jour : OUI ☐ NON ☐ : si OUI Heure : |__|__|:|__|__|
(critère(s) Neurologique☐ Hémodynamique ☐ autre ☐
Respiratoire ☐ (épuisement ☐ Encombrement ☐ Pneumopathie☐ OAP☐ Dyspnée laryngée☐))
Réintubation pour chirurgie avec intubation <24h OUI☐ NON ☐

A la fin de J48 le patient est :
Intubé ☐ Extubé ☐
Poursuite du protocole de sevrage ☐
Sortie du protocole de sevrage ☐ (remplir la partie « sortie d'essai »)
Poids du jour : |__|__|__| kg

J49 = |__|__|/|__|__/|__|__| de 0h à 23h59

Remplir si patient intubé le matin de J49

- Critères préalables de sevrabilité : Présents (-> Faire TVS) ☐ Absents☐
- TVS : Succès ☐ Echec ☐ (maintien intubation)
- Si succès :
  - Gaz du sang : pH |__|,|__|__|. pCO2 |__|__|__|mmHg pO2 |__|__|__|mmHg FiO2|__|__|__|%
  - Score de toux : 0 ☐ 1 ☐ 2 ☐ 3 ☐ 4 ☐ 5 ☐
  - Score d'encombrement : 0 ☐ 1 ☐ 2 ☐ 3 ☐ 4 ☐
  - Patients du groupe A (assisté) : TVS-TT Succès ☐ Echec ☐ (-> VNI prophylactique)
- Evaluer extubabilité si succès du TVS :
  - Force de toux acceptable (≥3) : Oui ☐ Non ☐
  - Encombrement acceptable (≤2) : Oui ☐ Non ☐
  - Absence d'AG dans les 24h qui suivent : Oui ☐ Non ☐
  - Pas d'argument pour une obstruction laryngée : Oui ☐ Non ☐

➔ Si ≥3 critères « oui » : extubation proposée :
➔ Heure d'extubation (hh :mm): |__|__|:|__|__|

Et raison de la non-extubation le cas échéant : ____________________________

Remplir si le patient n'est plus intubé à un moment de J49 (même quelques minutes ou depuis plusieurs jours)

Patient extubé AUJOURD'HUI OUI ☐ NON ☐
Sinon Date de la dernière extubation |__|__|/|__|__|/|__|__| Heure |__|__|:|__|__|

- Extubation (si réalisée AUJOURD'HUI)
  - programmée☐ (selon protocole☐ violation protocole☐)
  - auto-extubation ☐
- VNI prophylactique post-extubation OUI ☐ NON ☐
  - *Si VNI prophylactique*
  - selon protocole ☐ (TVS-TT (-)☐ âge>65ans☐ BPCO☐ Insuffisance cardiaque chronique ☐ Insuffisance respiratoire chronique☐, PaCO2>45 en fin de TVS-TT☐) violation protocole ☐
  - Durée cumulée sur les 24h : |__|__|h
  - *Si absence de VNI prophylactique*
  - selon protocole ☐ violation protocole ☐ contre-indication ☐
- VNI curative post-extubation NON ☐ selon protocole ☐ violation protocole ☐
- Optiflow post-extubation (non recommandé) OUI ☐ NON ☐
- Désencombrement bronchique : NON ☐ Toux manuellement assistée ☐
  Toux assistée instrumentale ☐

Réintubation ce jour : OUI ☐ NON ☐ : si OUI Heure : |__|__|:|__|__|
(critère(s) Neurologique☐ Hémodynamique ☐ autre ☐
Respiratoire ☐ (épuisement ☐ Encombrement ☐ Pneumopathie☐ OAP☐ Dyspnée laryngée☐))
Réintubation pour chirurgie avec intubation <24h OUI☐ NON ☐

A la fin de J49 le patient est :
Intubé ☐ Extubé ☐
Poursuite du protocole de sevrage ☐
Sortie du protocole de sevrage ☐ (remplir la partie « sortie d'essai »)
Poids du jour : |__|__|__| kg

J50 = |__|__|/|__|__/|__|__| de 0h à 23h59

Remplir si patient intubé le matin de J50

- Critères préalables de sevrabilité : Présents (-> Faire TVS) ☐ Absents ☐
- TVS : Succès ☐ Echec ☐ (maintien intubation)
- Si succès :
  - Gaz du sang : pH |__|,|__|__|. pCO2 |__|__|__|mmHg pO2 |__|__|__|mmHg FiO2|__|__|__|%
  - Score de toux : 0 ☐ 1 ☐ 2 ☐ 3 ☐ 4 ☐ 5 ☐
  - Score d'encombrement : 0 ☐ 1 ☐ 2 ☐ 3 ☐ 4 ☐
  - Patients du groupe A (assisté) : TVS-TT Succès ☐ Echec ☐ (-> VNI prophylactique)
- Evaluer extubabilité si succès du TVS :
  - Force de toux acceptable (≥3) : Oui ☐ Non ☐
  - Encombrement acceptable (≤2) : Oui ☐ Non ☐
  - Absence d'AG dans les 24h qui suivent : Oui ☐ Non ☐
  - Pas d'argument pour une obstruction laryngée : Oui ☐ Non ☐

➔ Si ≥3 critères « oui » : extubation proposée :

➔ Heure d'extubation (hh :mm): |__|__|:|__|__|

Et raison de la non-extubation le cas échéant : ____________________________

Remplir si le patient n'est plus intubé à un moment de J50 (même quelques minutes ou depuis plusieurs jours)

Patient extubé AUJOURD'HUI OUI ☐ NON ☐

Sinon Date de la dernière extubation |__|__|/|__|__|/|__|__| Heure |__|__|:|__|__|

- Extubation (si réalisée AUJOURD'HUI)
  - programmée☐ (selon protocole☐ violation protocole☐)
  - auto-extubation ☐
- VNI prophylactique post-extubation OUI ☐ NON ☐
  - *Si VNI prophylactique*
  - selon protocole ☐ (TVS-TT (-)☐ âge>65ans☐ BPCO☐ Insuffisance cardiaque chronique ☐ Insuffisance respiratoire chronique☐, PaCO2>45 en fin de TVS-TT☐) violation protocole ☐
  - Durée cumulée sur les 24h : |__|__|h
  - *Si absence de VNI prophylactique*
  - selon protocole ☐ violation protocole ☐ contre-indication ☐
- VNI curative post-extubation NON ☐ selon protocole ☐ violation protocole ☐
- Optiflow post-extubation (non recommandé) OUI ☐ NON ☐
- Désencombrement bronchique : NON ☐ Toux manuellement assistée ☐
  Toux assistée instrumentale ☐

Réintubation ce jour : OUI ☐ NON ☐ : si OUI Heure : |__|__|:|__|__|

(critère(s) Neurologique☐ Hémodynamique ☐ autre ☐

Respiratoire ☐ (épuisement ☐ Encombrement ☐ Pneumopathie☐ OAP☐ Dyspnée laryngée☐))

Réintubation pour chirurgie avec intubation <24h OUI☐ NON ☐

A la fin de J50 le patient est :

Intubé ☐ Extubé ☐

Poursuite du protocole de sevrage ☐

Sortie du protocole de sevrage ☐ (remplir la partie « sortie d'essai »)

Poids du jour : |__|__|__| kg

J51 = |__|__|/|__|__/|__|__| de 0h à 23h59

Remplir si patient intubé le matin de J51

- Critères préalables de sevrabilité : Présents (-> Faire TVS) ☐ Absents☐
- TVS : Succès ☐ Echec ☐ (maintien intubation)
- Si succès :
  - Gaz du sang : pH |__|,|__|__|. pCO2 |__|__|__|mmHg pO2 |__|__|__|mmHg FiO2|__|__|__|%
  - Score de toux : 0 ☐ 1 ☐ 2 ☐ 3 ☐ 4 ☐ 5 ☐
  - Score d'encombrement : 0 ☐ 1 ☐ 2 ☐ 3 ☐ 4 ☐
  - Patients du groupe A (assisté) : TVS-TT Succès ☐ Echec ☐ (-> VNI prophylactique)
- Evaluer extubabilité si succès du TVS :
  - Force de toux acceptable (≥3) : Oui ☐ Non ☐
  - Encombrement acceptable (≤2) : Oui ☐ Non ☐
  - Absence d'AG dans les 24h qui suivent : Oui ☐ Non ☐
  - Pas d'argument pour une obstruction laryngée : Oui ☐ Non ☐

➔ Si ≥3 critères « oui » : extubation proposée :

➔ Heure d'extubation (hh :mm): |__|__|:|__|__|

Et raison de la non-extubation le cas échéant : ____________________________

Remplir si le patient n'est plus intubé à un moment de J51 (même quelques minutes ou depuis plusieurs jours)

Patient extubé AUJOURD'HUI OUI ☐ NON ☐

Sinon Date de la dernière extubation |__|__|/|__|__|/|__|__| Heure |__|__|:|__|__|

- Extubation (si réalisée AUJOURD'HUI)
  - programmée☐ (selon protocole☐ violation protocole☐)
  - auto-extubation ☐
- VNI prophylactique post-extubation OUI ☐ NON ☐
  - *Si VNI prophylactique*
  - selon protocole ☐ (TVS-TT (-)☐ âge>65ans☐ BPCO☐ Insuffisance cardiaque chronique ☐ Insuffisance respiratoire chronique☐, PaCO2>45 en fin de TVS-TT☐) violation protocole ☐
  - Durée cumulée sur les 24h : |__|__|h
  - *Si absence de VNI prophylactique*
  - selon protocole ☐ violation protocole ☐ contre-indication ☐
- VNI curative post-extubation NON ☐ selon protocole ☐ violation protocole ☐
- Optiflow post-extubation (non recommandé) OUI ☐ NON ☐
- Désencombrement bronchique : NON ☐ Toux manuellement assistée ☐
  Toux assistée instrumentale ☐

Réintubation ce jour : OUI ☐ NON ☐ : si OUI Heure : |__|__|:|__|__|

(critère(s) Neurologique☐ Hémodynamique ☐ autre ☐

Respiratoire ☐ (épuisement ☐ Encombrement ☐ Pneumopathie☐ OAP☐ Dyspnée laryngée☐))

Réintubation pour chirurgie avec intubation <24h OUI☐ NON ☐

A la fin de J51 le patient est :

Intubé ☐ Extubé ☐

Poursuite du protocole de sevrage ☐

Sortie du protocole de sevrage ☐ (remplir la partie « sortie d'essai »)

Poids du jour : |__|__|__| kg

J52 = |__|__|/|__|__/|__|__| de 0h à 23h59

Remplir si patient intubé le matin de J52

- Critères préalables de sevrabilité : Présents (-> Faire TVS) ☐ Absents☐
- TVS : Succès ☐ Echec ☐ (maintien intubation)
- Si succès :
  - Gaz du sang : pH |__|,|__|__|. pCO2 |__|__|__|mmHg pO2 |__|__|__|mmHg FiO2|__|__|__|%
  - Score de toux : 0 ☐ 1 ☐ 2 ☐ 3 ☐ 4 ☐ 5 ☐
  - Score d'encombrement : 0 ☐ 1 ☐ 2 ☐ 3 ☐ 4 ☐
  - Patients du groupe A (assisté) : TVS-TT Succès ☐ Echec ☐ (-> VNI prophylactique)
- Evaluer extubabilité si succès du TVS :
  - Force de toux acceptable (≥3) : Oui ☐ Non ☐
  - Encombrement acceptable (≤2) : Oui ☐ Non ☐
  - Absence d'AG dans les 24h qui suivent : Oui ☐ Non ☐
  - Pas d'argument pour une obstruction laryngée : Oui ☐ Non ☐

➔ Si ≥3 critères « oui » : extubation proposée :

➔ Heure d'extubation (hh :mm): |__|__|:|__|__|

Et raison de la non-extubation le cas échéant : ___________________________

Remplir si le patient n'est plus intubé à un moment de J52 (même quelques minutes ou depuis plusieurs jours)

Patient extubé AUJOURD'HUI OUI ☐ NON ☐

Sinon Date de la dernière extubation |__|__|/|__|__|/|__|__| Heure |__|__|:|__|__|

- Extubation (si réalisée AUJOURD'HUI)
  - programmée☐ (selon protocole☐ violation protocole☐)
  - auto-extubation ☐
- VNI prophylactique post-extubation OUI ☐ NON ☐
  - *Si VNI prophylactique*
  - selon protocole ☐ (TVS-TT (-)☐ âge>65ans☐ BPCO☐ Insuffisance cardiaque chronique ☐ Insuffisance respiratoire chronique☐, PaCO2>45 en fin de TVS-TT☐) violation protocole ☐
  - Durée cumulée sur les 24h : |__|__|h
  - *Si absence de VNI prophylactique*
  - selon protocole ☐ violation protocole ☐ contre-indication ☐
- VNI curative post-extubation NON ☐ selon protocole ☐ violation protocole ☐
- Optiflow post-extubation (non recommandé) OUI ☐ NON ☐
- Désencombrement bronchique : NON ☐ Toux manuellement assistée ☐
  Toux assistée instrumentale ☐

Réintubation ce jour : OUI ☐ NON ☐ : si OUI Heure : |__|__|:|__|__|
(critère(s) Neurologique☐ Hémodynamique ☐ autre ☐
Respiratoire ☐ (épuisement ☐ Encombrement ☐ Pneumopathie☐ OAP☐ Dyspnée laryngée☐))
Réintubation pour chirurgie avec intubation <24h OUI☐ NON ☐

A la fin de J52 le patient est :
Intubé ☐ Extubé ☐
Poursuite du protocole de sevrage ☐
Sortie du protocole de sevrage ☐ (remplir la partie « sortie d'essai »)
Poids du jour : |__|__|__| kg

J53 = |__|__|/|__|__/|__|__| de 0h à 23h59

Remplir si patient intubé le matin de J53

- Critères préalables de sevrabilité : Présents (-> Faire TVS) ☐ Absents☐
- TVS : Succès ☐ Echec ☐ (maintien intubation)
- Si succès :
  - Gaz du sang : pH |__|,|__|__|. pCO2 |__|__|__|mmHg pO2 |__|__|__|mmHg FiO2|__|__|__|%
  - Score de toux : 0 ☐ 1 ☐ 2 ☐ 3 ☐ 4 ☐ 5 ☐
  - Score d'encombrement : 0 ☐ 1 ☐ 2 ☐ 3 ☐ 4 ☐
  - Patients du groupe A (assisté) : TVS-TT Succès ☐ Echec ☐ (-> VNI prophylactique)
- Evaluer extubabilité si succès du TVS :
  - Force de toux acceptable (≥3) : Oui ☐ Non ☐
  - Encombrement acceptable (≤2) : Oui ☐ Non ☐
  - Absence d'AG dans les 24h qui suivent : Oui ☐ Non ☐
  - Pas d'argument pour une obstruction laryngée : Oui ☐ Non ☐

➔ Si ≥3 critères « oui » : extubation proposée :

➔ Heure d'extubation (hh :mm): |__|__|:|__|__|

Et raison de la non-extubation le cas échéant : ____________________________

Remplir si le patient n'est plus intubé à un moment de J53 (même quelques minutes ou depuis plusieurs jours)

Patient extubé AUJOURD'HUI OUI ☐ NON ☐

Sinon Date de la dernière extubation |__|__|/|__|__|/|__|__| Heure |__|__|:|__|__|

- Extubation (si réalisée AUJOURD'HUI)
  - programmée☐ (selon protocole☐ violation protocole☐)
  - auto-extubation ☐
- VNI prophylactique post-extubation OUI ☐ NON ☐
  - *Si VNI prophylactique*
  - selon protocole ☐ (TVS-TT (-)☐ âge>65ans☐ BPCO☐ Insuffisance cardiaque chronique ☐ Insuffisance respiratoire chronique☐, PaCO2>45 en fin de TVS-TT☐) violation protocole ☐
  - Durée cumulée sur les 24h : |__|__|h
  - *Si absence de VNI prophylactique*
  - selon protocole ☐ violation protocole ☐ contre-indication ☐
- VNI curative post-extubation NON ☐ selon protocole ☐ violation protocole ☐
- Optiflow post-extubation (non recommandé) OUI ☐ NON ☐
- Désencombrement bronchique : NON ☐ Toux manuellement assistée ☐ Toux assistée instrumentale ☐

Réintubation ce jour : OUI ☐ NON ☐ : si OUI Heure : |__|__|:|__|__|
(critère(s) Neurologique☐ Hémodynamique ☐ autre ☐
Respiratoire ☐ (épuisement ☐ Encombrement ☐ Pneumopathie☐ OAP☐ Dyspnée laryngée☐))
Réintubation pour chirurgie avec intubation <24h OUI☐ NON ☐

A la fin de J53 le patient est :
Intubé ☐ Extubé ☐
Poursuite du protocole de sevrage ☐
Sortie du protocole de sevrage ☐ (remplir la partie « sortie d'essai »)
Poids du jour : |__|__|__| kg

J54 = |__|__|/|__|__/|__|__| de 0h à 23h59

Remplir si patient intubé le matin de J54

- Critères préalables de sevrabilité : Présents (-> Faire TVS) ☐ Absents☐
- TVS : Succès ☐ Echec ☐ (maintien intubation)
- Si succès :
  - Gaz du sang : pH |__|,|__|__|. pCO2 |__|__|__|mmHg pO2 |__|__|__|mmHg FiO2|__|__|__|%
  - Score de toux : 0 ☐ 1 ☐ 2 ☐ 3 ☐ 4 ☐ 5 ☐
  - Score d'encombrement : 0 ☐ 1 ☐ 2 ☐ 3 ☐ 4 ☐
  - Patients du groupe A (assisté) : TVS-TT Succès ☐ Echec ☐ (-> VNI prophylactique)
- Evaluer extubabilité si succès du TVS :
  - Force de toux acceptable (≥3) : Oui ☐ Non ☐
  - Encombrement acceptable (≤2) : Oui ☐ Non ☐
  - Absence d'AG dans les 24h qui suivent : Oui ☐ Non ☐
  - Pas d'argument pour une obstruction laryngée : Oui ☐ Non ☐

➔ Si ≥3 critères « oui » : extubation proposée :

➔ Heure d'extubation (hh :mm): |__|__|:|__|__|

Et raison de la non-extubation le cas échéant : ______________________________

Remplir si le patient n'est plus intubé à un moment de J54 (même quelques minutes ou depuis plusieurs jours)

Patient extubé AUJOURD'HUI OUI ☐ NON ☐

Sinon Date de la dernière extubation |__|__|/|__|__|/|__|__| Heure |__|__|:|__|__|

- Extubation (si réalisée AUJOURD'HUI)
  - programmée☐ (selon protocole☐ violation protocole☐)
  - auto-extubation ☐
- VNI prophylactique post-extubation OUI ☐ NON ☐
  - *Si VNI prophylactique*
  - selon protocole ☐ (TVS-TT (-)☐ âge>65ans☐ BPCO☐ Insuffisance cardiaque chronique ☐ Insuffisance respiratoire chronique☐, PaCO2>45 en fin de TVS-TT☐) violation protocole ☐
  - Durée cumulée sur les 24h : |__|__|h
  - *Si absence de VNI prophylactique*
  - selon protocole ☐ violation protocole ☐ contre-indication ☐
- VNI curative post-extubation NON ☐ selon protocole ☐ violation protocole ☐
- Optiflow post-extubation (non recommandé) OUI ☐ NON ☐
- Désencombrement bronchique : NON ☐ Toux manuellement assistée ☐
  Toux assistée instrumentale ☐

Réintubation ce jour : OUI ☐ NON ☐ : si OUI Heure : |__|__|:|__|__|

(critère(s) Neurologique☐ Hémodynamique ☐ autre ☐

Respiratoire ☐ (épuisement ☐ Encombrement ☐ Pneumopathie☐ OAP☐ Dyspnée laryngée☐))

Réintubation pour chirurgie avec intubation <24h OUI☐ NON ☐

A la fin de J54 le patient est :

Intubé ☐ Extubé ☐

Poursuite du protocole de sevrage ☐

Sortie du protocole de sevrage ☐ (remplir la partie « sortie d'essai »)

Poids du jour : |__|__|__| kg

J55 = |__|__|/|__|__/|__|__| de 0h à 23h59

Remplir si patient intubé le matin de J55

- Critères préalables de sevrabilité : Présents (-> Faire TVS) ☐ Absents☐
- TVS : Succès ☐ Echec ☐ (maintien intubation)
- Si succès :
  - Gaz du sang : pH |__|,|__|__|. pCO2 |__|__|__|mmHg pO2 |__|__|__|mmHg FiO2|__|__|__|%
  - Score de toux : 0 ☐ 1 ☐ 2 ☐ 3 ☐ 4 ☐ 5 ☐
  - Score d'encombrement : 0 ☐ 1 ☐ 2 ☐ 3 ☐ 4 ☐
  - Patients du groupe A (assisté) : TVS-TT Succès ☐ Echec ☐ (-> VNI prophylactique)
- Evaluer extubabilité si succès du TVS :
  - Force de toux acceptable (≥3) : Oui ☐ Non ☐
  - Encombrement acceptable (≤2) : Oui ☐ Non ☐
  - Absence d'AG dans les 24h qui suivent : Oui ☐ Non ☐
  - Pas d'argument pour une obstruction laryngée : Oui ☐ Non ☐

➔ Si ≥3 critères « oui » : extubation proposée :

➔ Heure d'extubation (hh :mm): |__|__|:|__|__|

Et raison de la non-extubation le cas échéant : ____________________________

Remplir si le patient n'est plus intubé à un moment de J55 (même quelques minutes ou depuis plusieurs jours)

Patient extubé AUJOURD'HUI OUI ☐ NON ☐

Sinon Date de la dernière extubation |__|__|/|__|__|/|__|__| Heure |__|__|:|__|__|

- Extubation (si réalisée AUJOURD'HUI)
  - programmée☐ (selon protocole☐ violation protocole☐)
  - auto-extubation ☐
- VNI prophylactique post-extubation OUI ☐ NON ☐
  - *Si VNI prophylactique*
  - selon protocole ☐ (TVS-TT (-)☐ âge>65ans☐ BPCO☐ Insuffisance cardiaque chronique ☐ Insuffisance respiratoire chronique☐, PaCO2>45 en fin de TVS-TT☐) violation protocole ☐
  - Durée cumulée sur les 24h : |__|__|h
  - *Si absence de VNI prophylactique*
  - selon protocole ☐ violation protocole ☐ contre-indication ☐
- VNI curative post-extubation NON ☐ selon protocole ☐ violation protocole ☐
- Optiflow post-extubation (non recommandé) OUI ☐ NON ☐
- Désencombrement bronchique : NON ☐ Toux manuellement assistée ☐
  Toux assistée instrumentale ☐

Réintubation ce jour : OUI ☐ NON ☐ : si OUI Heure : |__|__|:|__|__|
(critère(s) Neurologique☐ Hémodynamique ☐ autre ☐
Respiratoire ☐ (épuisement ☐ Encombrement ☐ Pneumopathie☐ OAP☐ Dyspnée laryngée☐))
Réintubation pour chirurgie avec intubation <24h OUI☐ NON ☐

A la fin de J55 le patient est :
Intubé ☐ Extubé ☐
Poursuite du protocole de sevrage ☐
Sortie du protocole de sevrage ☐ (remplir la partie « sortie d'essai »)
Poids du jour : |__|__|__| kg

J56 = |__|__|/|__|__/|__|__| de 0h à 23h59

Remplir si patient intubé le matin de J56

- Critères préalables de sevrabilité : Présents (-> Faire TVS) ☐ Absents☐
- TVS : Succès ☐ Echec ☐ (maintien intubation)
- Si succès :
  - Gaz du sang : pH |__|,|__|__|. pCO2 |__|__|__|mmHg pO2 |__|__|__|mmHg FiO2|__|__|__|%
  - Score de toux : 0 ☐ 1 ☐ 2 ☐ 3 ☐ 4 ☐ 5 ☐
  - Score d'encombrement : 0 ☐ 1 ☐ 2 ☐ 3 ☐ 4 ☐
  - Patients du groupe A (assisté) : TVS-TT Succès ☐ Echec ☐ (-> VNI prophylactique)
- Evaluer extubabilité si succès du TVS :
  - Force de toux acceptable (≥3) : Oui ☐ Non ☐
  - Encombrement acceptable (≤2) : Oui ☐ Non ☐
  - Absence d'AG dans les 24h qui suivent : Oui ☐ Non ☐
  - Pas d'argument pour une obstruction laryngée : Oui ☐ Non ☐

➔ Si ≥3 critères « oui » : extubation proposée :

➔ Heure d'extubation (hh :mm): |__|__|:|__|__|

Et raison de la non-extubation le cas échéant : ______________________________

Remplir si le patient n'est plus intubé à un moment de J56 (même quelques minutes ou depuis plusieurs jours)

Patient extubé AUJOURD'HUI OUI ☐ NON ☐

Sinon Date de la dernière extubation |__|__|/|__|__|/|__|__| Heure |__|__|:|__|__|

- Extubation (si réalisée AUJOURD'HUI)
  - programmée☐ (selon protocole☐ violation protocole☐)
  - auto-extubation ☐
- VNI prophylactique post-extubation OUI ☐ NON ☐
  - *Si VNI prophylactique*
  - selon protocole ☐ (TVS-TT (-)☐ âge>65ans☐ BPCO☐ Insuffisance cardiaque chronique ☐ Insuffisance respiratoire chronique☐, PaCO2>45 en fin de TVS-TT☐) violation protocole ☐
  - Durée cumulée sur les 24h : |__|__|h
  - *Si absence de VNI prophylactique*
  - selon protocole ☐ violation protocole ☐ contre-indication ☐
- VNI curative post-extubation NON ☐ selon protocole ☐ violation protocole ☐
- Optiflow post-extubation (non recommandé) OUI ☐ NON ☐
- Désencombrement bronchique : NON ☐ Toux manuellement assistée ☐
  Toux assistée instrumentale ☐

Réintubation ce jour : OUI ☐ NON ☐ : si OUI Heure : |__|__|:|__|__|

(critère(s) Neurologique☐ Hémodynamique ☐ autre ☐

Respiratoire ☐ (épuisement ☐ Encombrement ☐ Pneumopathie☐ OAP☐ Dyspnée laryngée☐))

Réintubation pour chirurgie avec intubation <24h OUI☐ NON ☐

A la fin de J56 le patient est :

Intubé ☐ Extubé ☐

Poursuite du protocole de sevrage ☐

Sortie du protocole de sevrage ☐ (remplir la partie « sortie d'essai »)

Poids du jour : |__|__|__|kg

J57 = |__|__|/|__|__/|__|__| de 0h à 23h59

Remplir si patient intubé le matin de J57

- Critères préalables de sevrabilité : Présents (-> Faire TVS) ☐ Absents☐
- TVS : Succès ☐ Echec ☐ (maintien intubation)
- Si succès :
  - Gaz du sang : pH |__|,|__|__|. pCO2 |__|__|__|mmHg pO2 |__|__|__|mmHg FiO2|__|__|__|%
  - Score de toux : 0 ☐ 1 ☐ 2 ☐ 3 ☐ 4 ☐ 5 ☐
  - Score d'encombrement : 0 ☐ 1 ☐ 2 ☐ 3 ☐ 4 ☐
  - Patients du groupe A (assisté) : TVS-TT Succès ☐ Echec ☐ (-> VNI prophylactique)
- Evaluer extubabilité si succès du TVS :
  - Force de toux acceptable (≥3) : Oui ☐ Non ☐
  - Encombrement acceptable (≤2) : Oui ☐ Non ☐
  - Absence d'AG dans les 24h qui suivent : Oui ☐ Non ☐
  - Pas d'argument pour une obstruction laryngée : Oui ☐ Non ☐

➔ Si ≥3 critères « oui » : extubation proposée :

➔ Heure d'extubation (hh :mm): |__|__|:|__|__|

Et raison de la non-extubation le cas échéant : ____________________________

Remplir si le patient n'est plus intubé à un moment de J57 (même quelques minutes ou depuis plusieurs jours)

Patient extubé AUJOURD'HUI OUI ☐ NON ☐

Sinon Date de la dernière extubation |__|__|/|__|__|/|__|__| Heure |__|__|:|__|__|

- Extubation (si réalisée AUJOURD'HUI)
  - programmée☐ (selon protocole☐ violation protocole☐)
  - auto-extubation ☐
- VNI prophylactique post-extubation OUI ☐ NON ☐
  - *Si VNI prophylactique*
  - selon protocole ☐ (TVS-TT (-)☐ âge>65ans☐ BPCO☐ Insuffisance cardiaque chronique ☐ Insuffisance respiratoire chronique☐, PaCO2>45 en fin de TVS-TT☐) violation protocole ☐
  - Durée cumulée sur les 24h : |__|__|h
  - *Si absence de VNI prophylactique*
  - selon protocole ☐ violation protocole ☐ contre-indication ☐
- VNI curative post-extubation NON ☐ selon protocole ☐ violation protocole ☐
- Optiflow post-extubation (non recommandé) OUI ☐ NON ☐
- Désencombrement bronchique : NON ☐ Toux manuellement assistée ☐
  Toux assistée instrumentale ☐

Réintubation ce jour : OUI ☐ NON ☐ : si OUI Heure : |__|__|:|__|__|
(critère(s) Neurologique☐ Hémodynamique ☐ autre ☐
Respiratoire ☐ (épuisement ☐ Encombrement ☐ Pneumopathie☐ OAP☐ Dyspnée laryngée☐))
Réintubation pour chirurgie avec intubation <24h OUI☐ NON ☐

A la fin de J57 le patient est :
Intubé ☐ Extubé ☐
Poursuite du protocole de sevrage ☐
Sortie du protocole de sevrage ☐ (remplir la partie « sortie d'essai »)
Poids du jour : |__|__|__| kg

J58 = |__|__|/|__|__/|__|__| de 0h à 23h59

Remplir si patient intubé le matin de J58

- Critères préalables de sevrabilité : Présents (-> Faire TVS) ☐ Absents☐
- TVS : Succès ☐ Echec ☐ (maintien intubation)
- Si succès :
  - Gaz du sang : pH |__|,|__|__|. pCO2 |__|__|__|mmHg pO2 |__|__|__|mmHg FiO2|__|__|__|%
  - Score de toux : 0 ☐ 1 ☐ 2 ☐ 3 ☐ 4 ☐ 5 ☐
  - Score d'encombrement : 0 ☐ 1 ☐ 2 ☐ 3 ☐ 4 ☐
  - Patients du groupe A (assisté) : TVS-TT Succès ☐ Echec ☐ (-> VNI prophylactique)
- Evaluer extubabilité si succès du TVS :
  - Force de toux acceptable (≥3) : Oui ☐ Non ☐
  - Encombrement acceptable (≤2) : Oui ☐ Non ☐
  - Absence d'AG dans les 24h qui suivent : Oui ☐ Non ☐
  - Pas d'argument pour une obstruction laryngée : Oui ☐ Non ☐

➔ Si ≥3 critères « oui » : extubation proposée :

➔ Heure d'extubation (hh :mm): |__|__|:|__|__|

Et raison de la non-extubation le cas échéant : ____________________________

Remplir si le patient n'est plus intubé à un moment de J58 (même quelques minutes ou depuis plusieurs jours)

Patient extubé AUJOURD'HUI OUI ☐ NON ☐

Sinon Date de la dernière extubation |__|__|/|__|__|/|__|__| Heure |__|__|:|__|__|

- Extubation (si réalisée AUJOURD'HUI)
  - programmée☐ (selon protocole☐ violation protocole☐)
  - auto-extubation ☐
- VNI prophylactique post-extubation OUI ☐ NON ☐
  - *Si VNI prophylactique*
  - selon protocole ☐ (TVS-TT (-)☐ âge>65ans☐ BPCO☐ Insuffisance cardiaque chronique ☐ Insuffisance respiratoire chronique☐, PaCO2>45 en fin de TVS-TT☐) violation protocole ☐
  - Durée cumulée sur les 24h : |__|__|h
  - *Si absence de VNI prophylactique*
  - selon protocole ☐ violation protocole ☐ contre-indication ☐
- VNI curative post-extubation NON ☐ selon protocole ☐ violation protocole ☐
- Optiflow post-extubation (non recommandé) OUI ☐ NON ☐
- Désencombrement bronchique : NON ☐ Toux manuellement assistée ☐
  Toux assistée instrumentale ☐

Réintubation ce jour : OUI ☐ NON ☐ : si OUI Heure : |__|__|:|__|__|

(critère(s) Neurologique☐ Hémodynamique ☐ autre ☐

Respiratoire ☐ (épuisement ☐ Encombrement ☐ Pneumopathie☐ OAP☐ Dyspnée laryngée☐))

Réintubation pour chirurgie avec intubation <24h OUI☐ NON ☐

A la fin de J58 le patient est :

Intubé ☐ Extubé ☐

Poursuite du protocole de sevrage ☐

Sortie du protocole de sevrage ☐ (remplir la partie « sortie d'essai »)

Poids du jour : |__|__|__| kg

J59 = |__|__|/|__|__/|__|__| de 0h à 23h59

Remplir si patient intubé le matin de J59

- Critères préalables de sevrabilité : Présents (-> Faire TVS) ☐ Absents☐
- TVS : Succès ☐ Echec ☐ (maintien intubation)
- Si succès :
  - Gaz du sang : pH |__|,|__|__|. pCO2 |__|__|__|mmHg pO2 |__|__|__|mmHg FiO2|__|__|__|%
  - Score de toux : 0 ☐ 1 ☐ 2 ☐ 3 ☐ 4 ☐ 5 ☐
  - Score d'encombrement : 0 ☐ 1 ☐ 2 ☐ 3 ☐ 4 ☐
  - Patients du groupe A (assisté) : TVS-TT Succès ☐ Echec ☐ (-> VNI prophylactique)
- Evaluer extubabilité si succès du TVS :
  - Force de toux acceptable (≥3) : Oui ☐ Non ☐
  - Encombrement acceptable (≤2) : Oui ☐ Non ☐
  - Absence d'AG dans les 24h qui suivent : Oui ☐ Non ☐
  - Pas d'argument pour une obstruction laryngée : Oui ☐ Non ☐

➔ Si ≥3 critères « oui » : extubation proposée :

➔ Heure d'extubation (hh :mm): |__|__|:|__|__|

Et raison de la non-extubation le cas échéant : ______________________________

Remplir si le patient n'est plus intubé à un moment de J59 (même quelques minutes ou depuis plusieurs jours)

Patient extubé AUJOURD'HUI OUI ☐ NON ☐

Sinon Date de la dernière extubation |__|__|/|__|__|/|__|__| Heure |__|__|:|__|__|

- Extubation (si réalisée AUJOURD'HUI)
  - programmée☐ (selon protocole☐ violation protocole☐)
  - auto-extubation ☐
- VNI prophylactique post-extubation OUI ☐ NON ☐
  - *Si VNI prophylactique*
  - selon protocole ☐ (TVS-TT (-)☐ âge>65ans☐ BPCO☐ Insuffisance cardiaque chronique ☐ Insuffisance respiratoire chronique☐, PaCO2>45 en fin de TVS-TT☐) violation protocole ☐
  - Durée cumulée sur les 24h : |__|__|h
  - *Si absence de VNI prophylactique*
  - selon protocole ☐ violation protocole ☐ contre-indication ☐
- VNI curative post-extubation NON ☐ selon protocole ☐ violation protocole ☐
- Optiflow post-extubation (non recommandé) OUI ☐ NON ☐
- Désencombrement bronchique : NON ☐ Toux manuellement assistée ☐
  Toux assistée instrumentale ☐

Réintubation ce jour : OUI ☐ NON ☐ : si OUI Heure : |__|__|:|__|__|
(critère(s) Neurologique☐ Hémodynamique ☐ autre ☐
Respiratoire ☐ (épuisement ☐ Encombrement ☐ Pneumopathie☐ OAP☐ Dyspnée laryngée☐))
Réintubation pour chirurgie avec intubation <24h OUI☐ NON ☐

A la fin de J59 le patient est :
Intubé ☐ Extubé ☐
Poursuite du protocole de sevrage ☐
Sortie du protocole de sevrage ☐ (remplir la partie « sortie d'essai »)
Poids du jour : |__|__|__| kg

J60 = |__|__|/|__|__/|__|__| de 0h à 23h59

Remplir si patient intubé le matin de J60

- Critères préalables de sevrabilité : Présents (-> Faire TVS) ☐ Absents☐
- TVS : Succès ☐ Echec ☐ (maintien intubation)
- Si succès :
    - Gaz du sang : pH |__|,|__|__|. pCO2 |__|__|__|mmHg pO2 |__|__|__|mmHg FiO2|__|__|__|%
    - Score de toux : 0 ☐ 1 ☐ 2 ☐ 3 ☐ 4 ☐ 5 ☐
    - Score d'encombrement : 0 ☐ 1 ☐ 2 ☐ 3 ☐ 4 ☐
    - Patients du groupe A (assisté) : TVS-TT Succès ☐ Echec ☐ (-> VNI prophylactique)
- Evaluer extubabilité si succès du TVS :
    - Force de toux acceptable (≥3) : Oui ☐ Non ☐
    - Encombrement acceptable (≤2) : Oui ☐ Non ☐
    - Absence d'AG dans les 24h qui suivent : Oui ☐ Non ☐
    - Pas d'argument pour une obstruction laryngée : Oui ☐ Non ☐

➔ Si ≥3 critères « oui » : extubation proposée :

➔ Heure d'extubation (hh :mm): |__|__|:|__|__|

Et raison de la non-extubation le cas échéant : ____________________________

Remplir si le patient n'est plus intubé à un moment de J60 (même quelques minutes ou depuis plusieurs jours)

Patient extubé AUJOURD'HUI OUI ☐ NON ☐

Sinon Date de la dernière extubation |__|__|/|__|__|/|__|__| Heure |__|__|:|__|__|

- Extubation (si réalisée AUJOURD'HUI)
    - programmée☐ (selon protocole☐ violation protocole☐)
    - auto-extubation ☐
- VNI prophylactique post-extubation OUI ☐ NON ☐
    - *Si VNI prophylactique*
    - selon protocole ☐ (TVS-TT (-)☐ âge>65ans☐ BPCO☐ Insuffisance cardiaque chronique ☐ Insuffisance respiratoire chronique☐, PaCO2>45 en fin de TVS-TT☐) violation protocole ☐
    - Durée cumulée sur les 24h : |__|__|h
    - *Si absence de VNI prophylactique*
    - selon protocole ☐ violation protocole ☐ contre-indication ☐
- VNI curative post-extubation NON ☐ selon protocole ☐ violation protocole ☐
- Optiflow post-extubation (non recommandé) OUI ☐ NON ☐
- Désencombrement bronchique : NON ☐ Toux manuellement assistée ☐
  Toux assistée instrumentale ☐

Réintubation ce jour : OUI ☐ NON ☐ : si OUI Heure : |__|__|:|__|__|

(critère(s) Neurologique☐ Hémodynamique ☐ autre ☐

Respiratoire ☐ (épuisement ☐ Encombrement ☐ Pneumopathie☐ OAP☐ Dyspnée laryngée☐))

Réintubation pour chirurgie avec intubation <24h OUI☐ NON ☐

A la fin de J60 le patient est :

Intubé ☐ Extubé ☐

Poursuite du protocole de sevrage ☐

Sortie du protocole de sevrage ☐ (remplir la partie « sortie d'essai »)

Poids du jour : |__|__|__| kg

J61 = |__|__|/|__|__/|__|__| de 0h à 23h59

Remplir si patient intubé le matin de J61

- Critères préalables de sevrabilité : Présents (-> Faire TVS) ☐ Absents☐
- TVS : Succès ☐ Echec ☐ (maintien intubation)
- Si succès :
  - Gaz du sang : pH |__|,|__|__|. pCO2 |__|__|__|mmHg pO2 |__|__|__|mmHg FiO2|__|__|__|%
  - Score de toux : 0 ☐ 1 ☐ 2 ☐ 3 ☐ 4 ☐ 5 ☐
  - Score d'encombrement : 0 ☐ 1 ☐ 2 ☐ 3 ☐ 4 ☐
  - Patients du groupe A (assisté) : TVS-TT Succès ☐ Echec ☐ (-> VNI prophylactique)
- Evaluer extubabilité si succès du TVS :
  - Force de toux acceptable (≥3) : Oui ☐ Non ☐
  - Encombrement acceptable (≤2) : Oui ☐ Non ☐
  - Absence d'AG dans les 24h qui suivent : Oui ☐ Non ☐
  - Pas d'argument pour une obstruction laryngée : Oui ☐ Non ☐

➔ Si ≥3 critères « oui » : extubation proposée :

➔ Heure d'extubation (hh :mm): |__|__|:|__|__|

Et raison de la non-extubation le cas échéant : ______________________________

Remplir si le patient n'est plus intubé à un moment de J61 (même quelques minutes ou depuis plusieurs jours)

Patient extubé AUJOURD'HUI OUI ☐ NON ☐

Sinon Date de la dernière extubation |__|__|/|__|__|/|__|__| Heure |__|__|:|__|__|

- Extubation (si réalisée AUJOURD'HUI)
  - programmée☐ (selon protocole☐ violation protocole☐)
  - auto-extubation ☐
- VNI prophylactique post-extubation OUI ☐ NON ☐
  - *Si VNI prophylactique*
  - selon protocole ☐ (TVS-TT (-)☐ âge>65ans☐ BPCO☐ Insuffisance cardiaque chronique ☐ Insuffisance respiratoire chronique☐, PaCO2>45 en fin de TVS-TT☐) violation protocole ☐
  - Durée cumulée sur les 24h : |__|__|h
  - *Si absence de VNI prophylactique*
  - selon protocole ☐ violation protocole ☐ contre-indication ☐
- VNI curative post-extubation NON ☐ selon protocole ☐ violation protocole ☐
- Optiflow post-extubation (non recommandé) OUI ☐ NON ☐
- Désencombrement bronchique : NON ☐ Toux manuellement assistée ☐
  Toux assistée instrumentale ☐

Réintubation ce jour : OUI ☐ NON ☐ : si OUI Heure : |__|__|:|__|__|

(critère(s) Neurologique☐ Hémodynamique ☐ autre ☐

Respiratoire ☐ (épuisement ☐ Encombrement ☐ Pneumopathie☐ OAP☐ Dyspnée laryngée☐))

Réintubation pour chirurgie avec intubation <24h OUI☐ NON ☐

A la fin de J61 le patient est :

Intubé ☐ Extubé ☐

Poursuite du protocole de sevrage ☐

Sortie du protocole de sevrage ☐ (remplir la partie « sortie d'essai »)

Poids du jour : |__|__|__| kg

J62 = |__|__|/|__|__/|__|__| de 0h à 23h59

Remplir si patient intubé le matin de J62

- Critères préalables de sevrabilité : Présents (-> Faire TVS) ☐ Absents☐
- TVS : Succès ☐ Echec ☐ (maintien intubation)
- Si succès :
  - Gaz du sang : pH |__|,|__|__|. pCO2 |__|__|__|mmHg pO2 |__|__|__|mmHg FiO2|__|__|__|%
  - Score de toux : 0 ☐ 1 ☐ 2 ☐ 3 ☐ 4 ☐ 5 ☐
  - Score d'encombrement : 0 ☐ 1 ☐ 2 ☐ 3 ☐ 4 ☐
  - Patients du groupe A (assisté) : TVS-TT Succès ☐ Echec ☐ (-> VNI prophylactique)
- Evaluer extubabilité si succès du TVS :
  - Force de toux acceptable (≥3) : Oui ☐ Non ☐
  - Encombrement acceptable (≤2) : Oui ☐ Non ☐
  - Absence d'AG dans les 24h qui suivent : Oui ☐ Non ☐
  - Pas d'argument pour une obstruction laryngée : Oui ☐ Non ☐

➔ Si ≥3 critères « oui » : extubation proposée :

➔ Heure d'extubation (hh :mm): |__|__|:|__|__|

Et raison de la non-extubation le cas échéant : ____________________________

Remplir si le patient n'est plus intubé à un moment de J62 (même quelques minutes ou depuis plusieurs jours)

Patient extubé AUJOURD'HUI OUI ☐ NON ☐

Sinon Date de la dernière extubation |__|__|/|__|__|/|__|__| Heure |__|__|:|__|__|

- Extubation (si réalisée AUJOURD'HUI)
  - programmée☐ (selon protocole☐ violation protocole☐)
  - auto-extubation ☐
- VNI prophylactique post-extubation OUI ☐ NON ☐
  - *Si VNI prophylactique*
  - selon protocole ☐ (TVS-TT (-)☐ âge>65ans☐ BPCO☐ Insuffisance cardiaque chronique ☐ Insuffisance respiratoire chronique☐, PaCO2>45 en fin de TVS-TT☐) violation protocole ☐
  - Durée cumulée sur les 24h : |__|__|h
  - *Si absence de VNI prophylactique*
  - selon protocole ☐ violation protocole ☐ contre-indication ☐
- VNI curative post-extubation NON ☐ selon protocole ☐ violation protocole ☐
- Optiflow post-extubation (non recommandé) OUI ☐ NON ☐
- Désencombrement bronchique : NON ☐ Toux manuellement assistée ☐
  Toux assistée instrumentale ☐

Réintubation ce jour : OUI ☐ NON ☐ : si OUI Heure : |__|__|:|__|__|
(critère(s) Neurologique☐ Hémodynamique ☐ autre ☐
Respiratoire ☐ (épuisement ☐ Encombrement ☐ Pneumopathie☐ OAP☐ Dyspnée laryngée☐))
Réintubation pour chirurgie avec intubation <24h OUI☐ NON ☐

A la fin de J62 le patient est :
Intubé ☐ Extubé ☐
Poursuite du protocole de sevrage ☐
Sortie du protocole de sevrage ☐ (remplir la partie « sortie d'essai »)
Poids du jour : |__|__|__| kg

J63 = |__|__|/|__|__/|__|__| de 0h à 23h59

Remplir si patient intubé le matin de J63

- Critères préalables de sevrabilité : Présents (-> Faire TVS) ☐ Absents☐
- TVS : Succès ☐ Echec ☐ (maintien intubation)
- Si succès :
  - Gaz du sang : pH |__|,|__|__|. pCO2 |__|__|__|mmHg pO2 |__|__|__|mmHg FiO2|__|__|__|%
  - Score de toux : 0 ☐ 1 ☐ 2 ☐ 3 ☐ 4 ☐ 5 ☐
  - Score d'encombrement : 0 ☐ 1 ☐ 2 ☐ 3 ☐ 4 ☐
  - Patients du groupe A (assisté) : TVS-TT Succès ☐ Echec ☐ (-> VNI prophylactique)
- Evaluer extubabilité si succès du TVS :
  - Force de toux acceptable (≥3) : Oui ☐ Non ☐
  - Encombrement acceptable (≤2) : Oui ☐ Non ☐
  - Absence d'AG dans les 24h qui suivent : Oui ☐ Non ☐
  - Pas d'argument pour une obstruction laryngée : Oui ☐ Non ☐

➔ Si ≥3 critères « oui » : extubation proposée :

➔ Heure d'extubation (hh :mm): |__|__|:|__|__|

Et raison de la non-extubation le cas échéant : ____________________________

Remplir si le patient n'est plus intubé à un moment de J63 (même quelques minutes ou depuis plusieurs jours)

Patient extubé AUJOURD'HUI OUI ☐ NON ☐

Sinon Date de la dernière extubation |__|__|/|__|__|/|__|__| Heure |__|__|:|__|__|

- Extubation (si réalisée AUJOURD'HUI)
  - programmée☐ (selon protocole☐ violation protocole☐)
  - auto-extubation ☐
- VNI prophylactique post-extubation OUI ☐ NON ☐
  - *Si VNI prophylactique*
  - selon protocole ☐ (TVS-TT (-)☐ âge>65ans☐ BPCO☐ Insuffisance cardiaque chronique ☐ Insuffisance respiratoire chronique☐, PaCO2>45 en fin de TVS-TT☐) violation protocole ☐
  - Durée cumulée sur les 24h : |__|__|h
  - *Si absence de VNI prophylactique*
  - selon protocole ☐ violation protocole ☐ contre-indication ☐
- VNI curative post-extubation NON ☐ selon protocole ☐ violation protocole ☐
- Optiflow post-extubation (non recommandé) OUI ☐ NON ☐
- Désencombrement bronchique : NON ☐ Toux manuellement assistée ☐
  Toux assistée instrumentale ☐

Réintubation ce jour : OUI ☐ NON ☐ : si OUI Heure : |__|__|:|__|__|

(critère(s) Neurologique☐ Hémodynamique ☐ autre ☐

Respiratoire ☐ (épuisement ☐ Encombrement ☐ Pneumopathie☐ OAP☐ Dyspnée laryngée☐))

Réintubation pour chirurgie avec intubation <24h OUI☐ NON ☐

A la fin de J63 le patient est :

Intubé ☐ Extubé ☐

Poursuite du protocole de sevrage ☐

Sortie du protocole de sevrage ☐ (remplir la partie « sortie d'essai »)

Poids du jour : |__|__|__| kg

J64 = |__|__|/|__|__/|__|__| de 0h à 23h59

Remplir si patient intubé le matin de J64

- Critères préalables de sevrabilité : Présents (-> Faire TVS) ☐ Absents☐
- TVS : Succès ☐ Echec ☐ (maintien intubation)
- Si succès :
  - Gaz du sang : pH |__|,|__|__|. pCO2 |__|__|__|mmHg pO2 |__|__|__|mmHg FiO2|__|__|__|%
  - Score de toux : 0 ☐ 1 ☐ 2 ☐ 3 ☐ 4 ☐ 5 ☐
  - Score d'encombrement : 0 ☐ 1 ☐ 2 ☐ 3 ☐ 4 ☐
  - Patients du groupe A (assisté) : TVS-TT Succès ☐ Echec ☐ (-> VNI prophylactique)
- Evaluer extubabilité si succès du TVS :
  - Force de toux acceptable (≥3) : Oui ☐ Non ☐
  - Encombrement acceptable (≤2) : Oui ☐ Non ☐
  - Absence d'AG dans les 24h qui suivent : Oui ☐ Non ☐
  - Pas d'argument pour une obstruction laryngée : Oui ☐ Non ☐

➔ Si ≥3 critères « oui » : extubation proposée :

➔ Heure d'extubation (hh :mm): |__|__|:|__|__|

Et raison de la non-extubation le cas échéant : ______________________________

Remplir si le patient n'est plus intubé à un moment de J64 (même quelques minutes ou depuis plusieurs jours)

Patient extubé AUJOURD'HUI OUI ☐ NON ☐

Sinon Date de la dernière extubation |__|__|/|__|__|/|__|__| Heure |__|__|:|__|__|

- Extubation (si réalisée AUJOURD'HUI)
  - programmée☐ (selon protocole☐ violation protocole☐)
  - auto-extubation ☐
- VNI prophylactique post-extubation OUI ☐ NON ☐
  - *Si VNI prophylactique*
  - selon protocole ☐ (TVS-TT (-)☐ âge>65ans☐ BPCO☐ Insuffisance cardiaque chronique ☐ Insuffisance respiratoire chronique☐, PaCO2>45 en fin de TVS-TT☐) violation protocole ☐
  - Durée cumulée sur les 24h : |__|__|h
  - *Si absence de VNI prophylactique*
  - selon protocole ☐ violation protocole ☐ contre-indication ☐
- VNI curative post-extubation NON ☐ selon protocole ☐ violation protocole ☐
- Optiflow post-extubation (non recommandé) OUI ☐ NON ☐
- Désencombrement bronchique : NON ☐ Toux manuellement assistée ☐
  Toux assistée instrumentale ☐

Réintubation ce jour : OUI ☐ NON ☐ : si OUI Heure : |__|__|:|__|__|
(critère(s) Neurologique☐ Hémodynamique ☐ autre ☐
Respiratoire ☐ (épuisement ☐ Encombrement ☐ Pneumopathie☐ OAP☐ Dyspnée laryngée☐))
Réintubation pour chirurgie avec intubation <24h OUI☐ NON ☐

A la fin de J64 le patient est :
Intubé ☐ Extubé ☐
Poursuite du protocole de sevrage ☐
Sortie du protocole de sevrage ☐ (remplir la partie « sortie d'essai »)
Poids du jour : |__|__|__| kg

J65 = |__|__|/|__|__/|__|__| de 0h à 23h59

Remplir si patient intubé le matin de J65

- Critères préalables de sevrabilité : Présents (-> Faire TVS) ☐ Absents☐
- TVS : Succès ☐ Echec ☐ (maintien intubation)
- Si succès :
  - Gaz du sang : pH |__|,|__|__|. pCO2 |__|__|__|mmHg pO2 |__|__|__|mmHg FiO2|__|__|__|%
  - Score de toux : 0 ☐ 1 ☐ 2 ☐ 3 ☐ 4 ☐ 5 ☐
  - Score d'encombrement : 0 ☐ 1 ☐ 2 ☐ 3 ☐ 4 ☐
  - Patients du groupe A (assisté) : TVS-TT Succès ☐ Echec ☐ (-> VNI prophylactique)
- Evaluer extubabilité si succès du TVS :
  - Force de toux acceptable (≥3) : Oui ☐ Non ☐
  - Encombrement acceptable (≤2) : Oui ☐ Non ☐
  - Absence d'AG dans les 24h qui suivent : Oui ☐ Non ☐
  - Pas d'argument pour une obstruction laryngée : Oui ☐ Non ☐

➔ Si ≥3 critères « oui » : extubation proposée :

➔ Heure d'extubation (hh :mm): |__|__|:|__|__|

Et raison de la non-extubation le cas échéant : ____________________________

Remplir si le patient n'est plus intubé à un moment de J65 (même quelques minutes ou depuis plusieurs jours)

Patient extubé AUJOURD'HUI OUI ☐ NON ☐

Sinon Date de la dernière extubation |__|__|/|__|__|/|__|__| Heure |__|__|:|__|__|

- Extubation (si réalisée AUJOURD'HUI)
  - programmée☐ (selon protocole☐ violation protocole☐)
  - auto-extubation ☐
- VNI prophylactique post-extubation OUI ☐ NON ☐
  - *Si VNI prophylactique*
  - selon protocole ☐ (TVS-TT (-)☐ âge>65ans☐ BPCO☐ Insuffisance cardiaque chronique ☐ Insuffisance respiratoire chronique☐, PaCO2>45 en fin de TVS-TT☐) violation protocole ☐
  - Durée cumulée sur les 24h : |__|__|h
  - *Si absence de VNI prophylactique*
  - selon protocole ☐ violation protocole ☐ contre-indication ☐
- VNI curative post-extubation NON ☐ selon protocole ☐ violation protocole ☐
- Optiflow post-extubation (non recommandé) OUI ☐ NON ☐
- Désencombrement bronchique : NON ☐ Toux manuellement assistée ☐
  Toux assistée instrumentale ☐

Réintubation ce jour : OUI ☐ NON ☐ : si OUI Heure : |__|__|:|__|__|
(critère(s) Neurologique☐ Hémodynamique ☐ autre ☐
Respiratoire ☐ (épuisement ☐ Encombrement ☐ Pneumopathie☐ OAP☐ Dyspnée laryngée☐))
Réintubation pour chirurgie avec intubation <24h OUI☐ NON ☐

A la fin de J65 le patient est :
Intubé ☐ Extubé ☐
Poursuite du protocole de sevrage ☐
Sortie du protocole de sevrage ☐ (remplir la partie « sortie d'essai »)
Poids du jour : |__|__|__|kg

J66 = |__|__|/|__|__/|__|__| de 0h à 23h59

Remplir si patient intubé le matin de J66

- Critères préalables de sevrabilité : Présents (-> Faire TVS) ☐ Absents☐
- TVS : Succès ☐ Echec ☐ (maintien intubation)
- Si succès :
  - Gaz du sang : pH |__|,|__|__|. pCO2 |__|__|__|mmHg pO2 |__|__|__|mmHg FiO2|__|__|__|%
  - Score de toux : 0 ☐ 1 ☐ 2 ☐ 3 ☐ 4 ☐ 5 ☐
  - Score d'encombrement : 0 ☐ 1 ☐ 2 ☐ 3 ☐ 4 ☐
  - Patients du groupe A (assisté) : TVS-TT Succès ☐ Echec ☐ (-> VNI prophylactique)
- Evaluer extubabilité si succès du TVS :
  - Force de toux acceptable (≥3) : Oui ☐ Non ☐
  - Encombrement acceptable (≤2) : Oui ☐ Non ☐
  - Absence d'AG dans les 24h qui suivent : Oui ☐ Non ☐
  - Pas d'argument pour une obstruction laryngée : Oui ☐ Non ☐

➔ Si ≥3 critères « oui » : extubation proposée :

➔ Heure d'extubation (hh :mm): |__|__|:|__|__|

Et raison de la non-extubation le cas échéant : ______________________________

Remplir si le patient n'est plus intubé à un moment de J66 (même quelques minutes ou depuis plusieurs jours)

Patient extubé AUJOURD'HUI OUI ☐ NON ☐

Sinon Date de la dernière extubation |__|__|/|__|__|/|__|__| Heure |__|__|:|__|__|

- Extubation (si réalisée AUJOURD'HUI)
  - programmée☐ (selon protocole☐ violation protocole☐)
  - auto-extubation ☐
- VNI prophylactique post-extubation OUI ☐ NON ☐
  - *Si VNI prophylactique*
  - selon protocole ☐ (TVS-TT (-)☐ âge>65ans☐ BPCO☐ Insuffisance cardiaque chronique ☐ Insuffisance respiratoire chronique☐, PaCO2>45 en fin de TVS-TT☐) violation protocole ☐
  - Durée cumulée sur les 24h : |__|__|h
  - *Si absence de VNI prophylactique*
  - selon protocole ☐ violation protocole ☐ contre-indication ☐
- VNI curative post-extubation NON ☐ selon protocole ☐ violation protocole ☐
- Optiflow post-extubation (non recommandé) OUI ☐ NON ☐
- Désencombrement bronchique : NON ☐ Toux manuellement assistée ☐
  Toux assistée instrumentale ☐

Réintubation ce jour : OUI ☐ NON ☐ : si OUI Heure : |__|__|:|__|__|
(critère(s) Neurologique☐ Hémodynamique ☐ autre ☐
Respiratoire ☐ (épuisement ☐ Encombrement ☐ Pneumopathie☐ OAP☐ Dyspnée laryngée☐))
Réintubation pour chirurgie avec intubation <24h OUI☐ NON ☐

A la fin de J66 le patient est :
Intubé ☐ Extubé ☐
Poursuite du protocole de sevrage ☐
Sortie du protocole de sevrage ☐ (remplir la partie « sortie d'essai »)
Poids du jour : |__|__|__| kg

J67 = |__|__|/|__|__/|__|__| de 0h à 23h59

Remplir si patient intubé le matin de J67

- Critères préalables de sevrabilité : Présents (-> Faire TVS) ☐ Absents☐
- TVS : Succès ☐ Echec ☐ (maintien intubation)
- Si succès :
  - Gaz du sang : pH |__|,|__|__|. pCO2 |__|__|__|mmHg pO2 |__|__|__|mmHg FiO2|__|__|__|%
  - Score de toux : 0 ☐ 1 ☐ 2 ☐ 3 ☐ 4 ☐ 5 ☐
  - Score d'encombrement : 0 ☐ 1 ☐ 2 ☐ 3 ☐ 4 ☐
  - Patients du groupe A (assisté) : TVS-TT Succès ☐ Echec ☐ (-> VNI prophylactique)
- Evaluer extubabilité si succès du TVS :
  - Force de toux acceptable (≥3) : Oui ☐ Non ☐
  - Encombrement acceptable (≤2) : Oui ☐ Non ☐
  - Absence d'AG dans les 24h qui suivent : Oui ☐ Non ☐
  - Pas d'argument pour une obstruction laryngée : Oui ☐ Non ☐

➔ Si ≥3 critères « oui » : extubation proposée :

➔ Heure d'extubation (hh :mm): |__|__|:|__|__|

Et raison de la non-extubation le cas échéant : ____________________________

Remplir si le patient n'est plus intubé à un moment de J67 (même quelques minutes ou depuis plusieurs jours)

Patient extubé AUJOURD'HUI OUI ☐ NON ☐

Sinon Date de la dernière extubation |__|__|/|__|__|/|__|__| Heure |__|__|:|__|__|

- Extubation (si réalisée AUJOURD'HUI)
  - programmée☐ (selon protocole☐ violation protocole☐)
  - auto-extubation ☐
- VNI prophylactique post-extubation OUI ☐ NON ☐
  - *Si VNI prophylactique*
  - selon protocole ☐ (TVS-TT (-)☐ âge>65ans☐ BPCO☐ Insuffisance cardiaque chronique ☐ Insuffisance respiratoire chronique☐, PaCO2>45 en fin de TVS-TT☐) violation protocole ☐
  - Durée cumulée sur les 24h : |__|__|h
  - *Si absence de VNI prophylactique*
  - selon protocole ☐ violation protocole ☐ contre-indication ☐
- VNI curative post-extubation NON ☐ selon protocole ☐ violation protocole ☐
- Optiflow post-extubation (non recommandé) OUI ☐ NON ☐
- Désencombrement bronchique : NON ☐ Toux manuellement assistée ☐
  Toux assistée instrumentale ☐

Réintubation ce jour : OUI ☐ NON ☐ : si OUI Heure : |__|__|:|__|__|

(critère(s) Neurologique☐ Hémodynamique ☐ autre ☐

Respiratoire ☐ (épuisement ☐ Encombrement ☐ Pneumopathie☐ OAP☐ Dyspnée laryngée☐))

Réintubation pour chirurgie avec intubation <24h OUI☐ NON ☐

A la fin de J67 le patient est :

Intubé ☐ Extubé ☐

Poursuite du protocole de sevrage ☐

Sortie du protocole de sevrage ☐ (remplir la partie « sortie d'essai »)

Poids du jour : |__|__|__| kg

J68 = |__|__|/|__|__/|__|__| de 0h à 23h59

Remplir si patient intubé le matin de J68

- Critères préalables de sevrabilité : Présents (-> Faire TVS) ☐ Absents☐
- TVS : Succès ☐ Echec ☐ (maintien intubation)
- Si succès :
  - Gaz du sang : pH |__|,|__|__|. pCO2 |__|__|__|mmHg pO2 |__|__|__|mmHg FiO2|__|__|__|%
  - Score de toux : 0 ☐ 1 ☐ 2 ☐ 3 ☐ 4 ☐ 5 ☐
  - Score d'encombrement : 0 ☐ 1 ☐ 2 ☐ 3 ☐ 4 ☐
  - Patients du groupe A (assisté) : TVS-TT Succès ☐ Echec ☐ (-> VNI prophylactique)
- Evaluer extubabilité si succès du TVS :
  - Force de toux acceptable (≥3) : Oui ☐ Non ☐
  - Encombrement acceptable (≤2) : Oui ☐ Non ☐
  - Absence d'AG dans les 24h qui suivent : Oui ☐ Non ☐
  - Pas d'argument pour une obstruction laryngée : Oui ☐ Non ☐

➔ Si ≥3 critères « oui » : extubation proposée :

➔ Heure d'extubation (hh :mm): |__|__|:|__|__|

Et raison de la non-extubation le cas échéant : ____________________________

Remplir si le patient n'est plus intubé à un moment de J68 (même quelques minutes ou depuis plusieurs jours)

Patient extubé AUJOURD'HUI OUI ☐ NON ☐

Sinon Date de la dernière extubation |__|__|/|__|__|/|__|__| Heure |__|__|:|__|__|

- Extubation (si réalisée AUJOURD'HUI)
  - programmée☐ (selon protocole☐ violation protocole☐)
  - auto-extubation ☐
- VNI prophylactique post-extubation OUI ☐ NON ☐
  - *Si VNI prophylactique*
  - selon protocole ☐ (TVS-TT (-)☐ âge>65ans☐ BPCO☐ Insuffisance cardiaque chronique ☐ Insuffisance respiratoire chronique☐, PaCO2>45 en fin de TVS-TT☐) violation protocole ☐
  - Durée cumulée sur les 24h : |__|__|h
  - *Si absence de VNI prophylactique*
  - selon protocole ☐ violation protocole ☐ contre-indication ☐
- VNI curative post-extubation NON ☐ selon protocole ☐ violation protocole ☐
- Optiflow post-extubation (non recommandé) OUI ☐ NON ☐
- Désencombrement bronchique : NON ☐ Toux manuellement assistée ☐
  Toux assistée instrumentale ☐

Réintubation ce jour : OUI ☐ NON ☐ : si OUI Heure : |__|__|:|__|__|
(critère(s) Neurologique☐ Hémodynamique ☐ autre ☐
Respiratoire ☐ (épuisement ☐ Encombrement ☐ Pneumopathie☐ OAP☐ Dyspnée laryngée☐))
Réintubation pour chirurgie avec intubation <24h OUI☐ NON ☐

A la fin de J68 le patient est :
Intubé ☐ Extubé ☐
Poursuite du protocole de sevrage ☐
Sortie du protocole de sevrage ☐ (remplir la partie « sortie d'essai »)
Poids du jour : |__|__|__| kg

J69 = |__|__|/|__|__/|__|__| de 0h à 23h59

Remplir si patient intubé le matin de J69

- Critères préalables de sevrabilité : Présents (-> Faire TVS) ☐ Absents☐
- TVS : Succès ☐ Echec ☐ (maintien intubation)
- Si succès :
  - Gaz du sang : pH |__|,|__|__|. pCO2 |__|__|__|mmHg pO2 |__|__|__|mmHg FiO2|__|__|__|%
  - Score de toux : 0 ☐ 1 ☐ 2 ☐ 3 ☐ 4 ☐ 5 ☐
  - Score d'encombrement : 0 ☐ 1 ☐ 2 ☐ 3 ☐ 4 ☐
  - Patients du groupe A (assisté) : TVS-TT Succès ☐ Echec ☐ (-> VNI prophylactique)
- Evaluer extubabilité si succès du TVS :
  - Force de toux acceptable (≥3) : Oui ☐ Non ☐
  - Encombrement acceptable (≤2) : Oui ☐ Non ☐
  - Absence d'AG dans les 24h qui suivent : Oui ☐ Non ☐
  - Pas d'argument pour une obstruction laryngée : Oui ☐ Non ☐

➔ Si ≥3 critères « oui » : extubation proposée :

➔ Heure d'extubation (hh :mm): |__|__|:|__|__|

Et raison de la non-extubation le cas échéant : ____________________________

Remplir si le patient n'est plus intubé à un moment de J69 (même quelques minutes ou depuis plusieurs jours)

Patient extubé AUJOURD'HUI OUI ☐ NON ☐

Sinon Date de la dernière extubation |__|__|/|__|__|/|__|__| Heure |__|__|:|__|__|

- Extubation (si réalisée AUJOURD'HUI)
  - programmée☐ (selon protocole☐ violation protocole☐)
  - auto-extubation ☐
- VNI prophylactique post-extubation OUI ☐ NON ☐
  - *Si VNI prophylactique*
  - selon protocole ☐ (TVS-TT (-)☐ âge>65ans☐ BPCO☐ Insuffisance cardiaque chronique ☐ Insuffisance respiratoire chronique☐, PaCO2>45 en fin de TVS-TT☐) violation protocole ☐
  - Durée cumulée sur les 24h : |__|__|h
  - *Si absence de VNI prophylactique*
  - selon protocole ☐ violation protocole ☐ contre-indication ☐
- VNI curative post-extubation NON ☐ selon protocole ☐ violation protocole ☐
- Optiflow post-extubation (non recommandé) OUI ☐ NON ☐
- Désencombrement bronchique : NON ☐ Toux manuellement assistée ☐
  Toux assistée instrumentale ☐

Réintubation ce jour : OUI ☐ NON ☐ : si OUI Heure : |__|__|:|__|__|
(critère(s) Neurologique☐ Hémodynamique ☐ autre ☐
Respiratoire ☐ (épuisement ☐ Encombrement ☐ Pneumopathie☐ OAP☐ Dyspnée laryngée☐))
Réintubation pour chirurgie avec intubation <24h OUI☐ NON ☐

A la fin de J69 le patient est :
Intubé ☐ Extubé ☐
Poursuite du protocole de sevrage ☐
Sortie du protocole de sevrage ☐ (remplir la partie « sortie d'essai »)
Poids du jour : |__|__|__|kg

J70 = |__|__|/|__|__/|__|__| de 0h à 23h59

Remplir si patient intubé le matin de J70

- Critères préalables de sevrabilité : Présents (-> Faire TVS) ☐ Absents☐
- TVS : Succès ☐ Echec ☐ (maintien intubation)
- Si succès :
  - Gaz du sang : pH |__|,|__|__|. pCO2 |__|__|__|mmHg pO2 |__|__|__|mmHg FiO2|__|__|__|%
  - Score de toux : 0 ☐ 1 ☐ 2 ☐ 3 ☐ 4 ☐ 5 ☐
  - Score d'encombrement : 0 ☐ 1 ☐ 2 ☐ 3 ☐ 4 ☐
  - Patients du groupe A (assisté) : TVS-TT Succès ☐ Echec ☐ (-> VNI prophylactique)
- Evaluer extubabilité si succès du TVS :
  - Force de toux acceptable (≥3) : Oui ☐ Non ☐
  - Encombrement acceptable (≤2) : Oui ☐ Non ☐
  - Absence d'AG dans les 24h qui suivent : Oui ☐ Non ☐
  - Pas d'argument pour une obstruction laryngée : Oui ☐ Non ☐

➔ Si ≥3 critères « oui » : extubation proposée :

➔ Heure d'extubation (hh :mm): |__|__|:|__|__|

Et raison de la non-extubation le cas échéant : ____________________________

Remplir si le patient n'est plus intubé à un moment de J70 (même quelques minutes ou depuis plusieurs jours)

Patient extubé AUJOURD'HUI OUI ☐ NON ☐

Sinon Date de la dernière extubation |__|__|/|__|__|/|__|__| Heure |__|__|:|__|__|

- Extubation (si réalisée AUJOURD'HUI)
  - programmée☐ (selon protocole☐ violation protocole☐)
  - auto-extubation ☐
- VNI prophylactique post-extubation OUI ☐ NON ☐
  - *Si VNI prophylactique*
  - selon protocole ☐ (TVS-TT (-)☐ âge>65ans☐ BPCO☐ Insuffisance cardiaque chronique ☐ Insuffisance respiratoire chronique☐, PaCO2>45 en fin de TVS-TT☐) violation protocole ☐
  - Durée cumulée sur les 24h : |__|__|h
  - *Si absence de VNI prophylactique*
  - selon protocole ☐ violation protocole ☐ contre-indication ☐
- VNI curative post-extubation NON ☐ selon protocole ☐ violation protocole ☐
- Optiflow post-extubation (non recommandé) OUI ☐ NON ☐
- Désencombrement bronchique : NON ☐ Toux manuellement assistée ☐
  Toux assistée instrumentale ☐

Réintubation ce jour : OUI ☐ NON ☐ : si OUI Heure : |__|__|:|__|__|
(critère(s) Neurologique☐ Hémodynamique ☐ autre ☐
Respiratoire ☐ (épuisement ☐ Encombrement ☐ Pneumopathie☐ OAP☐ Dyspnée laryngée☐))
Réintubation pour chirurgie avec intubation <24h OUI☐ NON ☐

A la fin de J70 le patient est :
Intubé ☐ Extubé ☐
Poursuite du protocole de sevrage ☐
Sortie du protocole de sevrage ☐ (remplir la partie « sortie d'essai »)
Poids du jour : |__|__|__| kg

J71 = |__|__|/|__|__/|__|__| de 0h à 23h59

Remplir si patient intubé le matin de J71

- Critères préalables de sevrabilité : Présents (-> Faire TVS) ☐ Absents☐
- TVS : Succès ☐ Echec ☐ (maintien intubation)
- Si succès :
  - Gaz du sang : pH |__|,|__|__|. pCO2 |__|__|__|mmHg pO2 |__|__|__|mmHg FiO2|__|__|__|%
  - Score de toux : 0 ☐ 1 ☐ 2 ☐ 3 ☐ 4 ☐ 5 ☐
  - Score d'encombrement : 0 ☐ 1 ☐ 2 ☐ 3 ☐ 4 ☐
  - Patients du groupe A (assisté) : TVS-TT Succès ☐ Echec ☐ (-> VNI prophylactique)
- Evaluer extubabilité si succès du TVS :
  - Force de toux acceptable (≥3) : Oui ☐ Non ☐
  - Encombrement acceptable (≤2) : Oui ☐ Non ☐
  - Absence d'AG dans les 24h qui suivent : Oui ☐ Non ☐
  - Pas d'argument pour une obstruction laryngée : Oui ☐ Non ☐

➔ Si ≥3 critères « oui » : extubation proposée :

➔ Heure d'extubation (hh :mm): |__|__|:|__|__|

Et raison de la non-extubation le cas échéant : ____________________________

Remplir si le patient n'est plus intubé à un moment de J71 (même quelques minutes ou depuis plusieurs jours)

Patient extubé AUJOURD'HUI OUI ☐ NON ☐

Sinon Date de la dernière extubation |__|__|/|__|__|/|__|__| Heure |__|__|:|__|__|

- Extubation (si réalisée AUJOURD'HUI)
  - programmée☐ (selon protocole☐ violation protocole☐)
  - auto-extubation ☐
- VNI prophylactique post-extubation OUI ☐ NON ☐
  - *Si VNI prophylactique*
  - selon protocole ☐ (TVS-TT (-)☐ âge>65ans☐ BPCO☐ Insuffisance cardiaque chronique ☐ Insuffisance respiratoire chronique☐, PaCO2>45 en fin de TVS-TT☐) violation protocole ☐
  - Durée cumulée sur les 24h : |__|__|h
  - *Si absence de VNI prophylactique*
  - selon protocole ☐ violation protocole ☐ contre-indication ☐
- VNI curative post-extubation NON ☐ selon protocole ☐ violation protocole ☐
- Optiflow post-extubation (non recommandé) OUI ☐ NON ☐
- Désencombrement bronchique : NON ☐ Toux manuellement assistée ☐ Toux assistée instrumentale ☐

Réintubation ce jour : OUI ☐ NON ☐ : si OUI Heure : |__|__|:|__|__|
(critère(s) Neurologique☐ Hémodynamique ☐ autre ☐
Respiratoire ☐ (épuisement ☐ Encombrement ☐ Pneumopathie☐ OAP☐ Dyspnée laryngée☐))
Réintubation pour chirurgie avec intubation <24h OUI☐ NON ☐

A la fin de J71 le patient est :
Intubé ☐ Extubé ☐
Poursuite du protocole de sevrage ☐
Sortie du protocole de sevrage ☐ (remplir la partie « sortie d'essai »)
Poids du jour : |__|__|__| kg

J72 = |__|__|/|__|__/|__|__| de 0h à 23h59

Remplir si patient intubé le matin de J72

- Critères préalables de sevrabilité : Présents (-> Faire TVS) ☐ Absents☐
- TVS : Succès ☐ Echec ☐ (maintien intubation)
- Si succès :
  - Gaz du sang : pH |__|,|__|__|. pCO2 |__|__|__|mmHg pO2 |__|__|__|mmHg FiO2|__|__|__|%
  - Score de toux : 0 ☐ 1 ☐ 2 ☐ 3 ☐ 4 ☐ 5 ☐
  - Score d'encombrement : 0 ☐ 1 ☐ 2 ☐ 3 ☐ 4 ☐
  - Patients du groupe A (assisté) : TVS-TT Succès ☐ Echec ☐ (-> VNI prophylactique)
- Evaluer extubabilité si succès du TVS :
  - Force de toux acceptable (≥3) : Oui ☐ Non ☐
  - Encombrement acceptable (≤2) : Oui ☐ Non ☐
  - Absence d'AG dans les 24h qui suivent : Oui ☐ Non ☐
  - Pas d'argument pour une obstruction laryngée : Oui ☐ Non ☐

➔ Si ≥3 critères « oui » : extubation proposée :

➔ Heure d'extubation (hh :mm): |__|__|:|__|__|

Et raison de la non-extubation le cas échéant : ______________________________

Remplir si le patient n'est plus intubé à un moment de J72 (même quelques minutes ou depuis plusieurs jours)

Patient extubé AUJOURD'HUI OUI ☐ NON ☐

Sinon Date de la dernière extubation |__|__|/|__|__|/|__|__| Heure |__|__|:|__|__|

- Extubation (si réalisée AUJOURD'HUI)
  - programmée☐ (selon protocole☐ violation protocole☐)
  - auto-extubation ☐
- VNI prophylactique post-extubation OUI ☐ NON ☐
  - *Si VNI prophylactique*
  - selon protocole ☐ (TVS-TT (-)☐ âge>65ans☐ BPCO☐ Insuffisance cardiaque chronique ☐ Insuffisance respiratoire chronique☐, PaCO2>45 en fin de TVS-TT☐) violation protocole ☐
  - Durée cumulée sur les 24h : |__|__|h
  - *Si absence de VNI prophylactique*
  - selon protocole ☐ violation protocole ☐ contre-indication ☐
- VNI curative post-extubation NON ☐ selon protocole ☐ violation protocole ☐
- Optiflow post-extubation (non recommandé) OUI ☐ NON ☐
- Désencombrement bronchique : NON ☐ Toux manuellement assistée ☐
  Toux assistée instrumentale ☐

Réintubation ce jour : OUI ☐ NON ☐ : si OUI Heure : |__|__|:|__|__|

(critère(s) Neurologique☐ Hémodynamique ☐ autre ☐

Respiratoire ☐ (épuisement ☐ Encombrement ☐ Pneumopathie☐ OAP☐ Dyspnée laryngée☐))

Réintubation pour chirurgie avec intubation <24h OUI☐ NON ☐

A la fin de J72 le patient est :

Intubé ☐ Extubé ☐

Poursuite du protocole de sevrage ☐

Sortie du protocole de sevrage ☐ (remplir la partie « sortie d'essai »)

Poids du jour : |__|__|__|kg

J73 = |__|__|/|__|__/|__|__| de 0h à 23h59

Remplir si patient intubé le matin de J73

- Critères préalables de sevrabilité : Présents (-> Faire TVS) ☐ Absents☐
- TVS : Succès ☐ Echec ☐ (maintien intubation)
- Si succès :
  - Gaz du sang : pH |__|,|__|__|. pCO2 |__|__|__|mmHg pO2 |__|__|__|mmHg FiO2|__|__|__|%
  - Score de toux : 0 ☐ 1 ☐ 2 ☐ 3 ☐ 4 ☐ 5 ☐
  - Score d'encombrement : 0 ☐ 1 ☐ 2 ☐ 3 ☐ 4 ☐
  - Patients du groupe A (assisté) : TVS-TT Succès ☐ Echec ☐ (-> VNI prophylactique)
- Evaluer extubabilité si succès du TVS :
  - Force de toux acceptable (≥3) : Oui ☐ Non ☐
  - Encombrement acceptable (≤2) : Oui ☐ Non ☐
  - Absence d'AG dans les 24h qui suivent : Oui ☐ Non ☐
  - Pas d'argument pour une obstruction laryngée : Oui ☐ Non ☐

➔ Si ≥3 critères « oui » : extubation proposée :

➔ Heure d'extubation (hh :mm): |__|__|:|__|__|

Et raison de la non-extubation le cas échéant : ____________________________

Remplir si le patient n'est plus intubé à un moment de J73 (même quelques minutes ou depuis plusieurs jours)

Patient extubé AUJOURD'HUI OUI ☐ NON ☐

Sinon Date de la dernière extubation |__|__|/|__|__|/|__|__| Heure |__|__|:|__|__|

- Extubation (si réalisée AUJOURD'HUI)
  - programmée☐ (selon protocole☐ violation protocole☐)
  - auto-extubation ☐
- VNI prophylactique post-extubation OUI ☐ NON ☐
  - *Si VNI prophylactique*
  - selon protocole ☐ (TVS-TT (-)☐ âge>65ans☐ BPCO☐ Insuffisance cardiaque chronique ☐ Insuffisance respiratoire chronique☐, PaCO2>45 en fin de TVS-TT☐) violation protocole ☐
  - Durée cumulée sur les 24h : |__|__|h
  - *Si absence de VNI prophylactique*
  - selon protocole ☐ violation protocole ☐ contre-indication ☐
- VNI curative post-extubation NON ☐ selon protocole ☐ violation protocole ☐
- Optiflow post-extubation (non recommandé) OUI ☐ NON ☐
- Désencombrement bronchique : NON ☐ Toux manuellement assistée ☐
  Toux assistée instrumentale ☐

Réintubation ce jour : OUI ☐ NON ☐ : si OUI Heure : |__|__|:|__|__|
(critère(s) Neurologique☐ Hémodynamique ☐ autre ☐
Respiratoire ☐ (épuisement ☐ Encombrement ☐ Pneumopathie☐ OAP☐ Dyspnée laryngée☐))
Réintubation pour chirurgie avec intubation <24h OUI☐ NON ☐

A la fin de J73 le patient est :
Intubé ☐ Extubé ☐
Poursuite du protocole de sevrage ☐
Sortie du protocole de sevrage ☐ (remplir la partie « sortie d'essai »)
Poids du jour : |__|__|__| kg

J74 = |__|__|/|__|__/|__|__| de 0h à 23h59

Remplir si patient intubé le matin de J74

- Critères préalables de sevrabilité : Présents (-> Faire TVS) ☐ Absents☐
- TVS : Succès ☐ Echec ☐ (maintien intubation)
- Si succès :
  - Gaz du sang : pH |__|,|__|__|. pCO2 |__|__|__|mmHg pO2 |__|__|__|mmHg FiO2|__|__|__|%
  - Score de toux : 0 ☐ 1 ☐ 2 ☐ 3 ☐ 4 ☐ 5 ☐
  - Score d'encombrement : 0 ☐ 1 ☐ 2 ☐ 3 ☐ 4 ☐
  - Patients du groupe A (assisté) : TVS-TT Succès ☐ Echec ☐ (-> VNI prophylactique)
- Evaluer extubabilité si succès du TVS :
  - Force de toux acceptable (≥3) : Oui ☐ Non ☐
  - Encombrement acceptable (≤2) : Oui ☐ Non ☐
  - Absence d'AG dans les 24h qui suivent : Oui ☐ Non ☐
  - Pas d'argument pour une obstruction laryngée : Oui ☐ Non ☐

➔ Si ≥3 critères « oui » : extubation proposée :

➔ Heure d'extubation (hh :mm): |__|__|:|__|__|

Et raison de la non-extubation le cas échéant : ____________________________

Remplir si le patient n'est plus intubé à un moment de J74 (même quelques minutes ou depuis plusieurs jours)

Patient extubé AUJOURD'HUI OUI ☐ NON ☐

Sinon Date de la dernière extubation |__|__|/|__|__|/|__|__| Heure |__|__|:|__|__|

- Extubation (si réalisée AUJOURD'HUI)
  - programmée☐ (selon protocole☐ violation protocole☐)
  - auto-extubation ☐
- VNI prophylactique post-extubation OUI ☐ NON ☐
  - *Si VNI prophylactique*
  - selon protocole ☐ (TVS-TT (-)☐ âge>65ans☐ BPCO☐ Insuffisance cardiaque chronique ☐ Insuffisance respiratoire chronique☐, PaCO2>45 en fin de TVS-TT☐) violation protocole ☐
  - Durée cumulée sur les 24h : |__|__|h
  - *Si absence de VNI prophylactique*
  - selon protocole ☐ violation protocole ☐ contre-indication ☐
- VNI curative post-extubation NON ☐ selon protocole ☐ violation protocole ☐
- Optiflow post-extubation (non recommandé) OUI ☐ NON ☐
- Désencombrement bronchique : NON ☐ Toux manuellement assistée ☐
  Toux assistée instrumentale ☐

Réintubation ce jour : OUI ☐ NON ☐ : si OUI Heure : |__|__|:|__|__|
(critère(s) Neurologique☐ Hémodynamique ☐ autre ☐
Respiratoire ☐ (épuisement ☐ Encombrement ☐ Pneumopathie☐ OAP☐ Dyspnée laryngée☐))
Réintubation pour chirurgie avec intubation <24h OUI☐ NON ☐

A la fin de J74 le patient est :
Intubé ☐ Extubé ☐
Poursuite du protocole de sevrage ☐
Sortie du protocole de sevrage ☐ (remplir la partie « sortie d'essai »)
Poids du jour : |__|__|__|kg

J75 = |__|__|/|__|__/|__|__| de 0h à 23h59

Remplir si patient intubé le matin de J75

- Critères préalables de sevrabilité : Présents (-> Faire TVS) ☐ Absents☐
- TVS : Succès ☐ Echec ☐ (maintien intubation)
- Si succès :
  - Gaz du sang : pH |__|,|__|__|. pCO2 |__|__|__|mmHg pO2 |__|__|__|mmHg FiO2|__|__|__|%
  - Score de toux : 0 ☐ 1 ☐ 2 ☐ 3 ☐ 4 ☐ 5 ☐
  - Score d'encombrement : 0 ☐ 1 ☐ 2 ☐ 3 ☐ 4 ☐
  - Patients du groupe A (assisté) : TVS-TT Succès ☐ Echec ☐ (-> VNI prophylactique)
- Evaluer extubabilité si succès du TVS :
  - Force de toux acceptable (≥3) : Oui ☐ Non ☐
  - Encombrement acceptable (≤2) : Oui ☐ Non ☐
  - Absence d'AG dans les 24h qui suivent : Oui ☐ Non ☐
  - Pas d'argument pour une obstruction laryngée : Oui ☐ Non ☐

➔ Si ≥3 critères « oui » : extubation proposée :

➔ Heure d'extubation (hh :mm): |__|__|:|__|__|

Et raison de la non-extubation le cas échéant : ____________________________

Remplir si le patient n'est plus intubé à un moment de J75 (même quelques minutes ou depuis plusieurs jours)

Patient extubé AUJOURD'HUI OUI ☐ NON ☐

Sinon Date de la dernière extubation |__|__|/|__|__|/|__|__| Heure |__|__|:|__|__|

- Extubation (si réalisée AUJOURD'HUI)
  - programmée☐ (selon protocole☐ violation protocole☐)
  - auto-extubation ☐
- VNI prophylactique post-extubation OUI ☐ NON ☐
  - *Si VNI prophylactique*
  - selon protocole ☐ (TVS-TT (-)☐ âge>65ans☐ BPCO☐ Insuffisance cardiaque chronique ☐ Insuffisance respiratoire chronique☐, PaCO2>45 en fin de TVS-TT☐) violation protocole ☐
  - Durée cumulée sur les 24h : |__|__|h
  - *Si absence de VNI prophylactique*
  - selon protocole ☐ violation protocole ☐ contre-indication ☐
- VNI curative post-extubation NON ☐ selon protocole ☐ violation protocole ☐
- Optiflow post-extubation (non recommandé) OUI ☐ NON ☐
- Désencombrement bronchique : NON ☐ Toux manuellement assistée ☐
  Toux assistée instrumentale ☐

Réintubation ce jour : OUI ☐ NON ☐ : si OUI Heure : |__|__|:|__|__|

(critère(s) Neurologique☐ Hémodynamique ☐ autre ☐

Respiratoire ☐ (épuisement ☐ Encombrement ☐ Pneumopathie☐ OAP☐ Dyspnée laryngée☐))

Réintubation pour chirurgie avec intubation <24h OUI☐ NON ☐

A la fin de J75 le patient est :

Intubé ☐ Extubé ☐

Poursuite du protocole de sevrage ☐

Sortie du protocole de sevrage ☐ (remplir la partie « sortie d'essai »)

Poids du jour : |__|__|__| kg

J76 = |__|__|/|__|__/|__|__| de 0h à 23h59

Remplir si patient intubé le matin de J76

- Critères préalables de sevrabilité : Présents (-> Faire TVS) ☐ Absents ☐
- TVS : Succès ☐ Echec ☐ (maintien intubation)
- Si succès :
  - Gaz du sang : pH |__|,|__|__|. pCO2 |__|__|__|mmHg pO2 |__|__|__|mmHg FiO2|__|__|__|%
  - Score de toux : 0 ☐ 1 ☐ 2 ☐ 3 ☐ 4 ☐ 5 ☐
  - Score d'encombrement : 0 ☐ 1 ☐ 2 ☐ 3 ☐ 4 ☐
  - Patients du groupe A (assisté) : TVS-TT Succès ☐ Echec ☐ (-> VNI prophylactique)
- Evaluer extubabilité si succès du TVS :
  - Force de toux acceptable (≥3) : Oui ☐ Non ☐
  - Encombrement acceptable (≤2) : Oui ☐ Non ☐
  - Absence d'AG dans les 24h qui suivent : Oui ☐ Non ☐
  - Pas d'argument pour une obstruction laryngée : Oui ☐ Non ☐

➔ Si ≥3 critères « oui » : extubation proposée :

➔ Heure d'extubation (hh :mm): |__|__|:|__|__|

Et raison de la non-extubation le cas échéant : ____________________________

Remplir si le patient n'est plus intubé à un moment de J76 (même quelques minutes ou depuis plusieurs jours)

Patient extubé AUJOURD'HUI OUI ☐ NON ☐

Sinon Date de la dernière extubation |__|__|/|__|__|/|__|__| Heure |__|__|:|__|__|

- Extubation (si réalisée AUJOURD'HUI)
  - programmée☐ (selon protocole☐ violation protocole☐)
  - auto-extubation ☐
- VNI prophylactique post-extubation OUI ☐ NON ☐
  - *Si VNI prophylactique*
  - selon protocole ☐ (TVS-TT (-)☐ âge>65ans☐ BPCO☐ Insuffisance cardiaque chronique ☐ Insuffisance respiratoire chronique☐, PaCO2>45 en fin de TVS-TT☐) violation protocole ☐
  - Durée cumulée sur les 24h : |__|__|h
  - *Si absence de VNI prophylactique*
  - selon protocole ☐ violation protocole ☐ contre-indication ☐
- VNI curative post-extubation NON ☐ selon protocole ☐ violation protocole ☐
- Optiflow post-extubation (non recommandé) OUI ☐ NON ☐
- Désencombrement bronchique : NON ☐ Toux manuellement assistée ☐ Toux assistée instrumentale ☐

Réintubation ce jour : OUI ☐ NON ☐ : si OUI Heure : |__|__|:|__|__|
(critère(s) Neurologique☐ Hémodynamique ☐ autre ☐
Respiratoire ☐ (épuisement ☐ Encombrement ☐ Pneumopathie☐ OAP☐ Dyspnée laryngée☐))
Réintubation pour chirurgie avec intubation <24h OUI☐ NON ☐

A la fin de J76 le patient est :
Intubé ☐ Extubé ☐
Poursuite du protocole de sevrage ☐
Sortie du protocole de sevrage ☐ (remplir la partie « sortie d'essai »)
Poids du jour : |__|__|__|kg

J77 = |__|__|/|__|__/|__|__| de 0h à 23h59

Remplir si patient intubé le matin de J77

- Critères préalables de sevrabilité : Présents (-> Faire TVS) ☐ Absents ☐
- TVS : Succès ☐ Echec ☐ (maintien intubation)
- Si succès :
  - Gaz du sang : pH |__|,|__|__|. pCO2 |__|__|__|mmHg pO2 |__|__|__|mmHg FiO2|__|__|__|%
  - Score de toux : 0 ☐ 1 ☐ 2 ☐ 3 ☐ 4 ☐ 5 ☐
  - Score d'encombrement : 0 ☐ 1 ☐ 2 ☐ 3 ☐ 4 ☐
  - Patients du groupe A (assisté) : TVS-TT Succès ☐ Echec ☐ (-> VNI prophylactique)
- Evaluer extubabilité si succès du TVS :
  - Force de toux acceptable (≥3) : Oui ☐ Non ☐
  - Encombrement acceptable (≤2) : Oui ☐ Non ☐
  - Absence d'AG dans les 24h qui suivent : Oui ☐ Non ☐
  - Pas d'argument pour une obstruction laryngée : Oui ☐ Non ☐

➔ Si ≥3 critères « oui » : extubation proposée :

➔ Heure d'extubation (hh :mm): |__|__|:|__|__|

Et raison de la non-extubation le cas échéant : ____________________________

Remplir si le patient n'est plus intubé à un moment de J77 (même quelques minutes ou depuis plusieurs jours)

Patient extubé AUJOURD'HUI OUI ☐ NON ☐

Sinon Date de la dernière extubation |__|__|/|__|__|/|__|__| Heure |__|__|:|__|__|

- Extubation (si réalisée AUJOURD'HUI)
  - programmée ☐ (selon protocole ☐ violation protocole ☐)
  - auto-extubation ☐
- VNI prophylactique post-extubation OUI ☐ NON ☐
  - *Si VNI prophylactique*
  - selon protocole ☐ (TVS-TT (-) ☐ âge>65ans ☐ BPCO ☐ Insuffisance cardiaque chronique ☐ Insuffisance respiratoire chronique ☐, PaCO2>45 en fin de TVS-TT ☐) violation protocole ☐
  - Durée cumulée sur les 24h : |__|__|h
  - *Si absence de VNI prophylactique*
  - selon protocole ☐ violation protocole ☐ contre-indication ☐
- VNI curative post-extubation NON ☐ selon protocole ☐ violation protocole ☐
- Optiflow post-extubation (non recommandé) OUI ☐ NON ☐
- Désencombrement bronchique : NON ☐ Toux manuellement assistée ☐
  Toux assistée instrumentale ☐

Réintubation ce jour : OUI ☐ NON ☐ : si OUI Heure : |__|__|:|__|__|

(critère(s) Neurologique ☐ Hémodynamique ☐ autre ☐

Respiratoire ☐ (épuisement ☐ Encombrement ☐ Pneumopathie ☐ OAP ☐ Dyspnée laryngée ☐))

Réintubation pour chirurgie avec intubation <24h OUI ☐ NON ☐

A la fin de J77 le patient est :

Intubé ☐ Extubé ☐

Poursuite du protocole de sevrage ☐

Sortie du protocole de sevrage ☐ (remplir la partie « sortie d'essai »)

Poids du jour : |__|__|__| kg

J78 = |__|__|/|__|__/|__|__| de 0h à 23h59

Remplir si patient intubé le matin de J78

- Critères préalables de sevrabilité : Présents (-> Faire TVS) ☐ Absents☐
- TVS : Succès ☐ Echec ☐ (maintien intubation)
- Si succès :
  - Gaz du sang : pH |__|,|__|__|. pCO2 |__|__|__|mmHg pO2 |__|__|__|mmHg FiO2|__|__|__|%
  - Score de toux : 0 ☐ 1 ☐ 2 ☐ 3 ☐ 4 ☐ 5 ☐
  - Score d'encombrement : 0 ☐ 1 ☐ 2 ☐ 3 ☐ 4 ☐
  - Patients du groupe A (assisté) : TVS-TT Succès ☐ Echec ☐ (-> VNI prophylactique)
- Evaluer extubabilité si succès du TVS :
  - Force de toux acceptable (≥3) : Oui ☐ Non ☐
  - Encombrement acceptable (≤2) : Oui ☐ Non ☐
  - Absence d'AG dans les 24h qui suivent : Oui ☐ Non ☐
  - Pas d'argument pour une obstruction laryngée : Oui ☐ Non ☐

➔ Si ≥3 critères « oui » : extubation proposée :

➔ Heure d'extubation (hh :mm): |__|__|:|__|__|

Et raison de la non-extubation le cas échéant : ____________________________

Remplir si le patient n'est plus intubé à un moment de J78 (même quelques minutes ou depuis plusieurs jours)

Patient extubé AUJOURD'HUI OUI ☐ NON ☐

Sinon Date de la dernière extubation |__|__|/|__|__|/|__|__| Heure |__|__|:|__|__|

- Extubation (si réalisée AUJOURD'HUI)
  - programmée☐ (selon protocole☐ violation protocole☐)
  - auto-extubation ☐
- VNI prophylactique post-extubation OUI ☐ NON ☐
  - *Si VNI prophylactique*
  - selon protocole ☐ (TVS-TT (-)☐ âge>65ans☐ BPCO☐ Insuffisance cardiaque chronique ☐ Insuffisance respiratoire chronique☐, PaCO2>45 en fin de TVS-TT☐) violation protocole ☐
  - Durée cumulée sur les 24h : |__|__|h
  - *Si absence de VNI prophylactique*
  - selon protocole ☐ violation protocole ☐ contre-indication ☐
- VNI curative post-extubation NON ☐ selon protocole ☐ violation protocole ☐
- Optiflow post-extubation (non recommandé) OUI ☐ NON ☐
- Désencombrement bronchique : NON ☐ Toux manuellement assistée ☐ Toux assistée instrumentale ☐

Réintubation ce jour : OUI ☐ NON ☐ : si OUI Heure : |__|__|:|__|__|
(critère(s) Neurologique☐ Hémodynamique ☐ autre ☐
Respiratoire ☐ (épuisement ☐ Encombrement ☐ Pneumopathie☐ OAP☐ Dyspnée laryngée☐))
Réintubation pour chirurgie avec intubation <24h OUI☐ NON ☐

A la fin de J78 le patient est :
Intubé ☐ Extubé ☐
Poursuite du protocole de sevrage ☐
Sortie du protocole de sevrage ☐ (remplir la partie « sortie d'essai »)
Poids du jour : |__|__|__| kg

J79 = |__|__|/|__|__/|__|__| de 0h à 23h59

Remplir si patient intubé le matin de J79

- Critères préalables de sevrabilité : Présents (-> Faire TVS) ☐ Absents ☐
- TVS : Succès ☐ Echec ☐ (maintien intubation)
- Si succès :
  - Gaz du sang : pH |__|,|__|__|. pCO2 |__|__|__|mmHg pO2 |__|__|__|mmHg FiO2|__|__|__|%
  - Score de toux : 0 ☐ 1 ☐ 2 ☐ 3 ☐ 4 ☐ 5 ☐
  - Score d'encombrement : 0 ☐ 1 ☐ 2 ☐ 3 ☐ 4 ☐
  - Patients du groupe A (assisté) : TVS-TT Succès ☐ Echec ☐ (-> VNI prophylactique)
- Evaluer extubabilité si succès du TVS :
  - Force de toux acceptable (≥3) : Oui ☐ Non ☐
  - Encombrement acceptable (≤2) : Oui ☐ Non ☐
  - Absence d'AG dans les 24h qui suivent : Oui ☐ Non ☐
  - Pas d'argument pour une obstruction laryngée : Oui ☐ Non ☐

➔ Si ≥3 critères « oui » : extubation proposée :

➔ Heure d'extubation (hh :mm): |__|__|:|__|__|

Et raison de la non-extubation le cas échéant : ____________________________

Remplir si le patient n'est plus intubé à un moment de J79 (même quelques minutes ou depuis plusieurs jours)

Patient extubé AUJOURD'HUI OUI ☐ NON ☐

Sinon Date de la dernière extubation |__|__|/|__|__|/|__|__| Heure |__|__|:|__|__|

- Extubation (si réalisée AUJOURD'HUI)
  - programmée☐ (selon protocole☐ violation protocole☐)
  - auto-extubation ☐
- VNI prophylactique post-extubation OUI ☐ NON ☐
  - *Si VNI prophylactique*
  - selon protocole ☐ (TVS-TT (-)☐ âge>65ans☐ BPCO☐ Insuffisance cardiaque chronique ☐ Insuffisance respiratoire chronique☐, PaCO2>45 en fin de TVS-TT☐) violation protocole ☐
  - Durée cumulée sur les 24h : |__|__|h
  - *Si absence de VNI prophylactique*
  - selon protocole ☐ violation protocole ☐ contre-indication ☐
- VNI curative post-extubation NON ☐ selon protocole ☐ violation protocole ☐
- Optiflow post-extubation (non recommandé) OUI ☐ NON ☐
- Désencombrement bronchique : NON ☐ Toux manuellement assistée ☐
  Toux assistée instrumentale ☐

Réintubation ce jour : OUI ☐ NON ☐ : si OUI Heure : |__|__|:|__|__|
(critère(s) Neurologique☐ Hémodynamique ☐ autre ☐
Respiratoire ☐ (épuisement ☐ Encombrement ☐ Pneumopathie☐ OAP☐ Dyspnée laryngée☐))
Réintubation pour chirurgie avec intubation <24h OUI☐ NON ☐

A la fin de J79 le patient est :
Intubé ☐ Extubé ☐
Poursuite du protocole de sevrage ☐
Sortie du protocole de sevrage ☐ (remplir la partie « sortie d'essai »)
Poids du jour : |__|__|__| kg

J80 = |__|__|/|__|__/|__|__| de 0h à 23h59

Remplir si patient intubé le matin de J80

- Critères préalables de sevrabilité : Présents (-> Faire TVS) ☐ Absents☐
- TVS : Succès ☐ Echec ☐ (maintien intubation)
- Si succès :
  - Gaz du sang : pH |__|,|__|__| . pCO2 |__|__|__|mmHg pO2 |__|__|__|mmHg FiO2|__|__|__|%
  - Score de toux : 0 ☐ 1 ☐ 2 ☐ 3 ☐ 4 ☐ 5 ☐
  - Score d'encombrement : 0 ☐ 1 ☐ 2 ☐ 3 ☐ 4 ☐
  - Patients du groupe A (assisté) : TVS-TT Succès ☐ Echec ☐ (-> VNI prophylactique)
- Evaluer extubabilité si succès du TVS :
  - Force de toux acceptable (≥3) : Oui ☐ Non ☐
  - Encombrement acceptable (≤2) : Oui ☐ Non ☐
  - Absence d'AG dans les 24h qui suivent : Oui ☐ Non ☐
  - Pas d'argument pour une obstruction laryngée : Oui ☐ Non ☐

➔ Si ≥3 critères « oui » : extubation proposée :
➔ Heure d'extubation (hh :mm): |__|__|:|__|__|

Et raison de la non-extubation le cas échéant : ____________________________

Remplir si le patient n'est plus intubé à un moment de J80 (même quelques minutes ou depuis plusieurs jours)

Patient extubé AUJOURD'HUI OUI ☐ NON ☐
Sinon Date de la dernière extubation |__|__|/|__|__|/|__|__| Heure |__|__|:|__|__|

- Extubation (si réalisée AUJOURD'HUI)
  - programmée☐ (selon protocole☐ violation protocole☐)
  - auto-extubation ☐
- VNI prophylactique post-extubation OUI ☐ NON ☐
  - *Si VNI prophylactique*
  - selon protocole ☐ (TVS-TT (-)☐ âge>65ans☐ BPCO☐ Insuffisance cardiaque chronique ☐ Insuffisance respiratoire chronique☐, PaCO2>45 en fin de TVS-TT☐) violation protocole ☐
  - Durée cumulée sur les 24h : |__|__|h
  - *Si absence de VNI prophylactique*
  - selon protocole ☐ violation protocole ☐ contre-indication ☐
- VNI curative post-extubation NON ☐ selon protocole ☐ violation protocole ☐
- Optiflow post-extubation (non recommandé) OUI ☐ NON ☐
- Désencombrement bronchique : NON ☐ Toux manuellement assistée ☐ Toux assistée instrumentale ☐

Réintubation ce jour : OUI ☐ NON ☐ : si OUI Heure : |__|__|:|__|__|
(critère(s) Neurologique☐ Hémodynamique ☐ autre ☐
Respiratoire ☐ (épuisement ☐ Encombrement ☐ Pneumopathie☐ OAP☐ Dyspnée laryngée☐))
Réintubation pour chirurgie avec intubation <24h OUI☐ NON ☐

A la fin de J80 le patient est :
Intubé ☐ Extubé ☐
Poursuite du protocole de sevrage ☐
Sortie du protocole de sevrage ☐ (remplir la partie « sortie d'essai »)
Poids du jour : |__|__|__|kg

J81 = |__|__|/|__|__/|__|__| de 0h à 23h59

Remplir si patient intubé le matin de J81

- Critères préalables de sevrabilité : Présents (-> Faire TVS) ☐ Absents ☐
- TVS : Succès ☐ Echec ☐ (maintien intubation)
- Si succès :
  - Gaz du sang : pH |__|,|__|__|. pCO2 |__|__|__| mmHg pO2 |__|__|__| mmHg FiO2 |__|__|__| %
  - Score de toux : 0 ☐ 1 ☐ 2 ☐ 3 ☐ 4 ☐ 5 ☐
  - Score d'encombrement : 0 ☐ 1 ☐ 2 ☐ 3 ☐ 4 ☐
  - Patients du groupe A (assisté) : TVS-TT Succès ☐ Echec ☐ (-> VNI prophylactique)
- Evaluer extubabilité si succès du TVS :
  - Force de toux acceptable (≥3) : Oui ☐ Non ☐
  - Encombrement acceptable (≤2) : Oui ☐ Non ☐
  - Absence d'AG dans les 24h qui suivent : Oui ☐ Non ☐
  - Pas d'argument pour une obstruction laryngée : Oui ☐ Non ☐

➔ Si ≥3 critères « oui » : extubation proposée :
➔ Heure d'extubation (hh :mm): |__|__|:|__|__|

Et raison de la non-extubation le cas échéant : ____________________________

Remplir si le patient n'est plus intubé à un moment de J81 (même quelques minutes ou depuis plusieurs jours)

Patient extubé AUJOURD'HUI OUI ☐ NON ☐
Sinon Date de la dernière extubation |__|__|/|__|__|/|__|__| Heure |__|__|:|__|__|

- Extubation (si réalisée AUJOURD'HUI)
  - programmée ☐ (selon protocole ☐ violation protocole ☐)
  - auto-extubation ☐
- VNI prophylactique post-extubation OUI ☐ NON ☐
  - *Si VNI prophylactique*
  - selon protocole ☐ (TVS-TT (-) ☐ âge>65ans ☐ BPCO ☐ Insuffisance cardiaque chronique ☐ Insuffisance respiratoire chronique ☐, PaCO2>45 en fin de TVS-TT ☐) violation protocole ☐
  - Durée cumulée sur les 24h : |__|__| h
  - *Si absence de VNI prophylactique*
  - selon protocole ☐ violation protocole ☐ contre-indication ☐
- VNI curative post-extubation NON ☐ selon protocole ☐ violation protocole ☐
- Optiflow post-extubation (non recommandé) OUI ☐ NON ☐
- Désencombrement bronchique : NON ☐ Toux manuellement assistée ☐
  Toux assistée instrumentale ☐

Réintubation ce jour : OUI ☐ NON ☐ : si OUI Heure : |__|__|:|__|__|
(critère(s) Neurologique ☐ Hémodynamique ☐ autre ☐
Respiratoire ☐ (épuisement ☐ Encombrement ☐ Pneumopathie ☐ OAP ☐ Dyspnée laryngée ☐))
Réintubation pour chirurgie avec intubation <24h OUI ☐ NON ☐

A la fin de J81 le patient est :
Intubé ☐ Extubé ☐
Poursuite du protocole de sevrage ☐
Sortie du protocole de sevrage ☐ (remplir la partie « sortie d'essai »)
Poids du jour : |__|__|__| kg

J82 = |__|__|/|__|__/|__|__| de 0h à 23h59

Remplir si patient intubé le matin de J82

- Critères préalables de sevrabilité : Présents (-> Faire TVS) ☐ Absents☐
- TVS : Succès ☐ Echec ☐ (maintien intubation)
- Si succès :
  - Gaz du sang : pH |__|,|__|__|. pCO2 |__|__|__|mmHg pO2 |__|__|__|mmHg FiO2|__|__|__|%
  - Score de toux : 0 ☐ 1 ☐ 2 ☐ 3 ☐ 4 ☐ 5 ☐
  - Score d'encombrement : 0 ☐ 1 ☐ 2 ☐ 3 ☐ 4 ☐
  - Patients du groupe A (assisté) : TVS-TT Succès ☐ Echec ☐ (-> VNI prophylactique)
- Evaluer extubabilité si succès du TVS :
  - Force de toux acceptable (≥3) : Oui ☐ Non ☐
  - Encombrement acceptable (≤2) : Oui ☐ Non ☐
  - Absence d'AG dans les 24h qui suivent : Oui ☐ Non ☐
  - Pas d'argument pour une obstruction laryngée : Oui ☐ Non ☐

➔ Si ≥3 critères « oui » : extubation proposée :

➔ Heure d'extubation (hh :mm): |__|__|:|__|__|

Et raison de la non-extubation le cas échéant : ____________________________

Remplir si le patient n'est plus intubé à un moment de J82 (même quelques minutes ou depuis plusieurs jours)

Patient extubé AUJOURD'HUI OUI ☐ NON ☐

Sinon Date de la dernière extubation |__|__|/|__|__|/|__|__| Heure |__|__|:|__|__|

- Extubation (si réalisée AUJOURD'HUI)
  - programmée☐ (selon protocole☐ violation protocole☐)
  - auto-extubation ☐
- VNI prophylactique post-extubation OUI ☐ NON ☐
  - *Si VNI prophylactique*
  - selon protocole ☐ (TVS-TT (-)☐ âge>65ans☐ BPCO☐ Insuffisance cardiaque chronique ☐ Insuffisance respiratoire chronique☐, PaCO2>45 en fin de TVS-TT☐) violation protocole ☐
  - Durée cumulée sur les 24h : |__|__|h
  - *Si absence de VNI prophylactique*
  - selon protocole ☐ violation protocole ☐ contre-indication ☐
- VNI curative post-extubation NON ☐ selon protocole ☐ violation protocole ☐
- Optiflow post-extubation (non recommandé) OUI ☐ NON ☐
- Désencombrement bronchique : NON ☐ Toux manuellement assistée ☐
  Toux assistée instrumentale ☐

Réintubation ce jour : OUI ☐ NON ☐ : si OUI Heure : |__|__|:|__|__|
(critère(s) Neurologique☐ Hémodynamique ☐ autre ☐
Respiratoire ☐ (épuisement ☐ Encombrement ☐ Pneumopathie☐ OAP☐ Dyspnée laryngée☐))
Réintubation pour chirurgie avec intubation <24h OUI☐ NON ☐

A la fin de J82 le patient est :
Intubé ☐ Extubé ☐
Poursuite du protocole de sevrage ☐
Sortie du protocole de sevrage ☐ (remplir la partie « sortie d'essai »)
Poids du jour : |__|__|__|kg

J83 = |__|__|/|__|__/|__|__| de 0h à 23h59

Remplir si patient intubé le matin de J83

- Critères préalables de sevrabilité : Présents (-> Faire TVS) ☐ Absents☐
- TVS : Succès ☐ Echec ☐ (maintien intubation)
- Si succès :
  - Gaz du sang : pH |__|,|__|__|. pCO2 |__|__|__|mmHg pO2 |__|__|__|mmHg FiO2|__|__|__|%
  - Score de toux : 0 ☐ 1 ☐ 2 ☐ 3 ☐ 4 ☐ 5 ☐
  - Score d'encombrement : 0 ☐ 1 ☐ 2 ☐ 3 ☐ 4 ☐
  - Patients du groupe A (assisté) : TVS-TT Succès ☐ Echec ☐ (-> VNI prophylactique)
- Evaluer extubabilité si succès du TVS :
  - Force de toux acceptable (≥3) : Oui ☐ Non ☐
  - Encombrement acceptable (≤2) : Oui ☐ Non ☐
  - Absence d'AG dans les 24h qui suivent : Oui ☐ Non ☐
  - Pas d'argument pour une obstruction laryngée : Oui ☐ Non ☐

➔ Si ≥3 critères « oui » : extubation proposée :

➔ Heure d'extubation (hh :mm): |__|__|:|__|__|

Et raison de la non-extubation le cas échéant : ____________________________

Remplir si le patient n'est plus intubé à un moment de J83 (même quelques minutes ou depuis plusieurs jours)

Patient extubé AUJOURD'HUI OUI ☐ NON ☐

Sinon Date de la dernière extubation |__|__|/|__|__|/|__|__| Heure |__|__|:|__|__|

- Extubation (si réalisée AUJOURD'HUI)
  - programmée☐ (selon protocole☐ violation protocole☐)
  - auto-extubation ☐
- VNI prophylactique post-extubation OUI ☐ NON ☐
  - *Si VNI prophylactique*
  - selon protocole ☐ (TVS-TT (-)☐ âge>65ans☐ BPCO☐ Insuffisance cardiaque chronique ☐ Insuffisance respiratoire chronique☐, PaCO2>45 en fin de TVS-TT☐) violation protocole ☐
  - Durée cumulée sur les 24h : |__|__|h
  - *Si absence de VNI prophylactique*
  - selon protocole ☐ violation protocole ☐ contre-indication ☐
- VNI curative post-extubation NON ☐ selon protocole ☐ violation protocole ☐
- Optiflow post-extubation (non recommandé) OUI ☐ NON ☐
- Désencombrement bronchique : NON ☐ Toux manuellement assistée ☐
  Toux assistée instrumentale ☐

Réintubation ce jour : OUI ☐ NON ☐ : si OUI Heure : |__|__|:|__|__|
(critère(s) Neurologique☐ Hémodynamique ☐ autre ☐
Respiratoire ☐ (épuisement ☐ Encombrement ☐ Pneumopathie☐ OAP☐ Dyspnée laryngée☐))
Réintubation pour chirurgie avec intubation <24h OUI☐ NON ☐

A la fin de J83 le patient est :
Intubé ☐ Extubé ☐
Poursuite du protocole de sevrage ☐
Sortie du protocole de sevrage ☐ (remplir la partie « sortie d'essai »)
Poids du jour : |__|__|__| kg

J84 = |__|__|/|__|__/|__|__| de 0h à 23h59

Remplir si patient intubé le matin de J84

- Critères préalables de sevrabilité : Présents (-> Faire TVS) ☐ Absents ☐
- TVS : Succès ☐ Echec ☐ (maintien intubation)
- Si succès :
  - Gaz du sang : pH |__|,|__|__| . pCO2 |__|__|__| mmHg pO2 |__|__|__| mmHg FiO2 |__|__|__| %
  - Score de toux : 0 ☐ 1 ☐ 2 ☐ 3 ☐ 4 ☐ 5 ☐
  - Score d'encombrement : 0 ☐ 1 ☐ 2 ☐ 3 ☐ 4 ☐
  - Patients du groupe A (assisté) : TVS-TT Succès ☐ Echec ☐ (-> VNI prophylactique)
- Evaluer extubabilité si succès du TVS :
  - Force de toux acceptable (≥3) : Oui ☐ Non ☐
  - Encombrement acceptable (≤2) : Oui ☐ Non ☐
  - Absence d'AG dans les 24h qui suivent : Oui ☐ Non ☐
  - Pas d'argument pour une obstruction laryngée : Oui ☐ Non ☐

➔ Si ≥3 critères « oui » : extubation proposée :

➔ Heure d'extubation (hh :mm): |__|__|:|__|__|

Et raison de la non-extubation le cas échéant : ____________________________

Remplir si le patient n'est plus intubé à un moment de J84 (même quelques minutes ou depuis plusieurs jours)

Patient extubé AUJOURD'HUI OUI ☐ NON ☐

Sinon Date de la dernière extubation |__|__|/|__|__|/|__|__| Heure |__|__|:|__|__|

- Extubation (si réalisée AUJOURD'HUI)
  - programmée ☐ (selon protocole ☐ violation protocole ☐)
  - auto-extubation ☐
- VNI prophylactique post-extubation OUI ☐ NON ☐
  - *Si VNI prophylactique*
  - selon protocole ☐ (TVS-TT (-) ☐ âge>65ans ☐ BPCO ☐ Insuffisance cardiaque chronique ☐ Insuffisance respiratoire chronique ☐, PaCO2>45 en fin de TVS-TT ☐) violation protocole ☐
  - Durée cumulée sur les 24h : |__|__| h
  - *Si absence de VNI prophylactique*
  - selon protocole ☐ violation protocole ☐ contre-indication ☐
- VNI curative post-extubation NON ☐ selon protocole ☐ violation protocole ☐
- Optiflow post-extubation (non recommandé) OUI ☐ NON ☐
- Désencombrement bronchique : NON ☐ Toux manuellement assistée ☐
  Toux assistée instrumentale ☐

Réintubation ce jour : OUI ☐ NON ☐ : si OUI Heure : |__|__|:|__|__|
(critère(s) Neurologique ☐ Hémodynamique ☐ autre ☐
Respiratoire ☐ (épuisement ☐ Encombrement ☐ Pneumopathie ☐ OAP ☐ Dyspnée laryngée ☐))
Réintubation pour chirurgie avec intubation <24h OUI ☐ NON ☐

A la fin de J84 le patient est :
Intubé ☐ Extubé ☐
Poursuite du protocole de sevrage ☐
Sortie du protocole de sevrage ☐ (remplir la partie « sortie d'essai »)
Poids du jour : |__|__|__| kg

J85 = |__|__|/|__|__/|__|__| de 0h à 23h59

Remplir si patient intubé le matin de J85

- Critères préalables de sevrabilité : Présents (-> Faire TVS) ☐ Absents☐
- TVS : Succès ☐ Echec ☐ (maintien intubation)
- Si succès :
  - Gaz du sang : pH |__|,|__|__|. pCO2 |__|__|__|mmHg pO2 |__|__|__|mmHg FiO2|__|__|__|%
  - Score de toux : 0 ☐ 1 ☐ 2 ☐ 3 ☐ 4 ☐ 5 ☐
  - Score d'encombrement : 0 ☐ 1 ☐ 2 ☐ 3 ☐ 4 ☐
  - Patients du groupe A (assisté) : TVS-TT Succès ☐ Echec ☐ (-> VNI prophylactique)
- Evaluer extubabilité si succès du TVS :
  - Force de toux acceptable (≥3) : Oui ☐ Non ☐
  - Encombrement acceptable (≤2) : Oui ☐ Non ☐
  - Absence d'AG dans les 24h qui suivent : Oui ☐ Non ☐
  - Pas d'argument pour une obstruction laryngée : Oui ☐ Non ☐

➔ Si ≥3 critères « oui » : extubation proposée :
➔ Heure d'extubation (hh :mm): |__|__|:|__|__|

Et raison de la non-extubation le cas échéant : ______________________________

Remplir si le patient n'est plus intubé à un moment de J85 (même quelques minutes ou depuis plusieurs jours)

Patient extubé AUJOURD'HUI OUI ☐ NON ☐
Sinon Date de la dernière extubation |__|__|/|__|__|/|__|__| Heure |__|__|:|__|__|

- Extubation (si réalisée AUJOURD'HUI)
  - programmée☐ (selon protocole☐ violation protocole☐)
  - auto-extubation ☐
- VNI prophylactique post-extubation OUI ☐ NON ☐
  - *Si VNI prophylactique*
  - selon protocole ☐ (TVS-TT (-)☐ âge>65ans☐ BPCO☐ Insuffisance cardiaque chronique ☐ Insuffisance respiratoire chronique☐, PaCO2>45 en fin de TVS-TT☐) violation protocole ☐
  - Durée cumulée sur les 24h : |__|__|h
  - *Si absence de VNI prophylactique*
  - selon protocole ☐ violation protocole ☐ contre-indication ☐
- VNI curative post-extubation NON ☐ selon protocole ☐ violation protocole ☐
- Optiflow post-extubation (non recommandé) OUI ☐ NON ☐
- Désencombrement bronchique : NON ☐ Toux manuellement assistée ☐
  Toux assistée instrumentale ☐

Réintubation ce jour : OUI ☐ NON ☐ : si OUI Heure : |__|__|:|__|__|
(critère(s) Neurologique☐ Hémodynamique ☐ autre ☐
Respiratoire ☐ (épuisement ☐ Encombrement ☐ Pneumopathie☐ OAP☐ Dyspnée laryngée☐))
Réintubation pour chirurgie avec intubation <24h OUI☐ NON ☐

A la fin de J85 le patient est :
Intubé ☐ Extubé ☐
Poursuite du protocole de sevrage ☐
Sortie du protocole de sevrage ☐ (remplir la partie « sortie d'essai »)
Poids du jour : |__|__|__| kg

J86 = |__|__|/|__|__/|__|__| de 0h à 23h59

Remplir si patient intubé le matin de J86

- Critères préalables de sevrabilité : Présents (-> Faire TVS) ☐ Absents☐
- TVS : Succès ☐ Echec ☐ (maintien intubation)
- Si succès :
  - Gaz du sang : pH |__|,|__|__|. pCO2 |__|__|__|mmHg pO2 |__|__|__|mmHg FiO2|__|__|__|%
  - Score de toux : 0 ☐ 1 ☐ 2 ☐ 3 ☐ 4 ☐ 5 ☐
  - Score d'encombrement : 0 ☐ 1 ☐ 2 ☐ 3 ☐ 4 ☐
  - Patients du groupe A (assisté) : TVS-TT Succès ☐ Echec ☐ (-> VNI prophylactique)
- Evaluer extubabilité si succès du TVS :
  - Force de toux acceptable (≥3) : Oui ☐ Non ☐
  - Encombrement acceptable (≤2) : Oui ☐ Non ☐
  - Absence d'AG dans les 24h qui suivent : Oui ☐ Non ☐
  - Pas d'argument pour une obstruction laryngée : Oui ☐ Non ☐

➔ Si ≥3 critères « oui » : extubation proposée :

➔ Heure d'extubation (hh :mm): |__|__|:|__|__|

Et raison de la non-extubation le cas échéant : ____________________________

Remplir si le patient n'est plus intubé à un moment de J86 (même quelques minutes ou depuis plusieurs jours)

Patient extubé AUJOURD'HUI OUI ☐ NON ☐

Sinon Date de la dernière extubation |__|__|/|__|__|/|__|__| Heure |__|__|:|__|__|

- Extubation (si réalisée AUJOURD'HUI)
  - programmée☐ (selon protocole☐ violation protocole☐)
  - auto-extubation ☐
- VNI prophylactique post-extubation OUI ☐ NON ☐
  - *Si VNI prophylactique*
  - selon protocole ☐ (TVS-TT (-)☐ âge>65ans☐ BPCO☐ Insuffisance cardiaque chronique ☐ Insuffisance respiratoire chronique☐, PaCO2>45 en fin de TVS-TT☐) violation protocole ☐
  - Durée cumulée sur les 24h : |__|__|h
  - *Si absence de VNI prophylactique*
  - selon protocole ☐ violation protocole ☐ contre-indication ☐
- VNI curative post-extubation NON ☐ selon protocole ☐ violation protocole ☐
- Optiflow post-extubation (non recommandé) OUI ☐ NON ☐
- Désencombrement bronchique : NON ☐ Toux manuellement assistée ☐
  Toux assistée instrumentale ☐

Réintubation ce jour : OUI ☐ NON ☐ : si OUI Heure : |__|__|:|__|__|

(critère(s) Neurologique☐ Hémodynamique ☐ autre ☐

Respiratoire ☐ (épuisement ☐ Encombrement ☐ Pneumopathie☐ OAP☐ Dyspnée laryngée☐))

Réintubation pour chirurgie avec intubation <24h OUI☐ NON ☐

A la fin de J86 le patient est :

Intubé ☐ Extubé ☐

Poursuite du protocole de sevrage ☐

Sortie du protocole de sevrage ☐ (remplir la partie « sortie d'essai »)

Poids du jour : |__|__|__|kg

J87 = |__|__|/|__|__/|__|__| de 0h à 23h59

Remplir si patient intubé le matin de J87

- Critères préalables de sevrabilité : Présents (-> Faire TVS) ☐ Absents ☐
- TVS : Succès ☐ Echec ☐ (maintien intubation)
- Si succès :
  - Gaz du sang : pH |__|,|__|__|. pCO2 |__|__|__|mmHg pO2 |__|__|__|mmHg FiO2|__|__|__|%
  - Score de toux : 0 ☐ 1 ☐ 2 ☐ 3 ☐ 4 ☐ 5 ☐
  - Score d'encombrement : 0 ☐ 1 ☐ 2 ☐ 3 ☐ 4 ☐
  - Patients du groupe A (assisté) : TVS-TT Succès ☐ Echec ☐ (-> VNI prophylactique)
- Evaluer extubabilité si succès du TVS :
  - Force de toux acceptable (≥3) : Oui ☐ Non ☐
  - Encombrement acceptable (≤2) : Oui ☐ Non ☐
  - Absence d'AG dans les 24h qui suivent : Oui ☐ Non ☐
  - Pas d'argument pour une obstruction laryngée : Oui ☐ Non ☐

➔ Si ≥3 critères « oui » : extubation proposée :

➔ Heure d'extubation (hh :mm): |__|__|:|__|__|

Et raison de la non-extubation le cas échéant : ______________________________

Remplir si le patient n'est plus intubé à un moment de J87 (même quelques minutes ou depuis plusieurs jours)

Patient extubé AUJOURD'HUI OUI ☐ NON ☐

Sinon Date de la dernière extubation |__|__|/|__|__|/|__|__| Heure |__|__|:|__|__|

- Extubation (si réalisée AUJOURD'HUI)
  - programmée☐ (selon protocole☐ violation protocole☐)
  - auto-extubation ☐
- VNI prophylactique post-extubation OUI ☐ NON ☐
  - *Si VNI prophylactique*
  - selon protocole ☐ (TVS-TT (-)☐ âge>65ans☐ BPCO☐ Insuffisance cardiaque chronique ☐ Insuffisance respiratoire chronique☐, PaCO2>45 en fin de TVS-TT☐) violation protocole ☐
  - Durée cumulée sur les 24h : |__|__|h
  - *Si absence de VNI prophylactique*
  - selon protocole ☐ violation protocole ☐ contre-indication ☐
- VNI curative post-extubation NON ☐ selon protocole ☐ violation protocole ☐
- Optiflow post-extubation (non recommandé) OUI ☐ NON ☐
- Désencombrement bronchique : NON ☐ Toux manuellement assistée ☐ Toux assistée instrumentale ☐

Réintubation ce jour : OUI ☐ NON ☐ : si OUI Heure : |__|__|:|__|__|
(critère(s) Neurologique☐ Hémodynamique ☐ autre ☐
Respiratoire ☐ (épuisement ☐ Encombrement ☐ Pneumopathie☐ OAP☐ Dyspnée laryngée☐))
Réintubation pour chirurgie avec intubation <24h OUI☐ NON ☐

A la fin de J87 le patient est :
Intubé ☐ Extubé ☐
Poursuite du protocole de sevrage ☐
Sortie du protocole de sevrage ☐ (remplir la partie « sortie d'essai »)
Poids du jour : |__|__|__| kg

J88 = |__|__|/|__|__/|__|__| de 0h à 23h59

Remplir si patient intubé le matin de J88

- Critères préalables de sevrabilité : Présents (-> Faire TVS) ☐ Absents ☐
- TVS : Succès ☐ Echec ☐ (maintien intubation)
- Si succès :
  - Gaz du sang : pH |__|,|__|__|. pCO2 |__|__|__| mmHg pO2 |__|__|__| mmHg FiO2 |__|__|__| %
  - Score de toux : 0 ☐ 1 ☐ 2 ☐ 3 ☐ 4 ☐ 5 ☐
  - Score d'encombrement : 0 ☐ 1 ☐ 2 ☐ 3 ☐ 4 ☐
  - Patients du groupe A (assisté) : TVS-TT Succès ☐ Echec ☐ (-> VNI prophylactique)
- Evaluer extubabilité si succès du TVS :
  - Force de toux acceptable (≥3) : Oui ☐ Non ☐
  - Encombrement acceptable (≤2) : Oui ☐ Non ☐
  - Absence d'AG dans les 24h qui suivent : Oui ☐ Non ☐
  - Pas d'argument pour une obstruction laryngée : Oui ☐ Non ☐

➔ Si ≥3 critères « oui » : extubation proposée :

➔ Heure d'extubation (hh :mm): |__|__|:|__|__|

Et raison de la non-extubation le cas échéant : ____________________________

Remplir si le patient n'est plus intubé à un moment de J88 (même quelques minutes ou depuis plusieurs jours)

Patient extubé AUJOURD'HUI OUI ☐ NON ☐

Sinon Date de la dernière extubation |__|__|/|__|__|/|__|__| Heure |__|__|:|__|__|

- Extubation (si réalisée AUJOURD'HUI)
  - programmée ☐ (selon protocole ☐ violation protocole ☐)
  - auto-extubation ☐
- VNI prophylactique post-extubation OUI ☐ NON ☐
  - *Si VNI prophylactique*
  - selon protocole ☐ (TVS-TT (-) ☐ âge>65ans ☐ BPCO ☐ Insuffisance cardiaque chronique ☐ Insuffisance respiratoire chronique ☐, PaCO2>45 en fin de TVS-TT ☐) violation protocole ☐
  - Durée cumulée sur les 24h : |__|__| h
  - *Si absence de VNI prophylactique*
  - selon protocole ☐ violation protocole ☐ contre-indication ☐
- VNI curative post-extubation NON ☐ selon protocole ☐ violation protocole ☐
- Optiflow post-extubation (non recommandé) OUI ☐ NON ☐
- Désencombrement bronchique : NON ☐ Toux manuellement assistée ☐
  Toux assistée instrumentale ☐

Réintubation ce jour : OUI ☐ NON ☐ : si OUI Heure : |__|__|:|__|__|

(critère(s) Neurologique ☐ Hémodynamique ☐ autre ☐

Respiratoire ☐ (épuisement ☐ Encombrement ☐ Pneumopathie ☐ OAP ☐ Dyspnée laryngée ☐))

Réintubation pour chirurgie avec intubation <24h OUI ☐ NON ☐

A la fin de J88 le patient est :

Intubé ☐ Extubé ☐

Poursuite du protocole de sevrage ☐

Sortie du protocole de sevrage ☐ (remplir la partie « sortie d'essai »)

Poids du jour : |__|__|__| kg

J89 = |__|__|/|__|__/|__|__| de 0h à 23h59

Remplir si patient intubé le matin de J89

- Critères préalables de sevrabilité : Présents (-> Faire TVS) ☐ Absents☐
- TVS : Succès ☐ Echec ☐ (maintien intubation)
- Si succès :
  - Gaz du sang : pH |__|,|__|__|. pCO2 |__|__|__|mmHg pO2 |__|__|__|mmHg FiO2|__|__|__|%
  - Score de toux : 0 ☐ 1 ☐ 2 ☐ 3 ☐ 4 ☐ 5 ☐
  - Score d'encombrement : 0 ☐ 1 ☐ 2 ☐ 3 ☐ 4 ☐
  - Patients du groupe A (assisté) : TVS-TT Succès ☐ Echec ☐ (-> VNI prophylactique)
- Evaluer extubabilité si succès du TVS :
  - Force de toux acceptable (≥3) : Oui ☐ Non ☐
  - Encombrement acceptable (≤2) : Oui ☐ Non ☐
  - Absence d'AG dans les 24h qui suivent : Oui ☐ Non ☐
  - Pas d'argument pour une obstruction laryngée : Oui ☐ Non ☐

➔ Si ≥3 critères « oui » : extubation proposée :

➔ Heure d'extubation (hh :mm): |__|__|:|__|__|

Et raison de la non-extubation le cas échéant : ____________________________

Remplir si le patient n'est plus intubé à un moment de J89 (même quelques minutes ou depuis plusieurs jours)

Patient extubé AUJOURD'HUI OUI ☐ NON ☐

Sinon Date de la dernière extubation |__|__|/|__|__|/|__|__| Heure |__|__|:|__|__|

- Extubation (si réalisée AUJOURD'HUI)
  - programmée☐ (selon protocole☐ violation protocole☐)
  - auto-extubation ☐
- VNI prophylactique post-extubation OUI ☐ NON ☐
  - *Si VNI prophylactique*
  - selon protocole ☐ (TVS-TT (-)☐ âge>65ans☐ BPCO☐ Insuffisance cardiaque chronique ☐ Insuffisance respiratoire chronique☐, PaCO2>45 en fin de TVS-TT☐) violation protocole ☐
  - Durée cumulée sur les 24h : |__|__|h
  - *Si absence de VNI prophylactique*
  - selon protocole ☐ violation protocole ☐ contre-indication ☐
- VNI curative post-extubation NON ☐ selon protocole ☐ violation protocole ☐
- Optiflow post-extubation (non recommandé) OUI ☐ NON ☐
- Désencombrement bronchique : NON ☐ Toux manuellement assistée ☐ Toux assistée instrumentale ☐

Réintubation ce jour : OUI ☐ NON ☐ : si OUI Heure : |__|__|:|__|__|
(critère(s) Neurologique☐ Hémodynamique ☐ autre ☐
Respiratoire ☐ (épuisement ☐ Encombrement ☐ Pneumopathie☐ OAP☐ Dyspnée laryngée☐))
Réintubation pour chirurgie avec intubation <24h OUI☐ NON ☐

A la fin de J89 le patient est :
Intubé ☐ Extubé ☐
Poursuite du protocole de sevrage ☐
Sortie du protocole de sevrage ☐ (remplir la partie « sortie d'essai »)
Poids du jour : |__|__|__| kg

J90 = |__|__|/|__|__/|__|__| de 0h à 23h59

Remplir si patient intubé le matin de J90

- Critères préalables de sevrabilité : Présents (-> Faire TVS) ☐ Absents☐
- TVS : Succès ☐ Echec ☐ (maintien intubation)
- Si succès :
  - Gaz du sang : pH |__|,|__|__|. pCO2 |__|__|__|mmHg pO2 |__|__|__|mmHg FiO2|__|__|__|%
  - Score de toux : 0 ☐ 1 ☐ 2 ☐ 3 ☐ 4 ☐ 5 ☐
  - Score d'encombrement : 0 ☐ 1 ☐ 2 ☐ 3 ☐ 4 ☐
  - Patients du groupe A (assisté) : TVS-TT Succès ☐ Echec ☐ (-> VNI prophylactique)
- Evaluer extubabilité si succès du TVS :
  - Force de toux acceptable (≥3) : Oui ☐ Non ☐
  - Encombrement acceptable (≤2) : Oui ☐ Non ☐
  - Absence d'AG dans les 24h qui suivent : Oui ☐ Non ☐
  - Pas d'argument pour une obstruction laryngée : Oui ☐ Non ☐

➔ Si ≥3 critères « oui » : extubation proposée :
➔ Heure d'extubation (hh :mm): |__|__|:|__|__|

Et raison de la non-extubation le cas échéant : ____________________________

Remplir si le patient n'est plus intubé à un moment de J90 (même quelques minutes ou depuis plusieurs jours)

Patient extubé AUJOURD'HUI OUI ☐ NON ☐
Sinon Date de la dernière extubation |__|__|/|__|__|/|__|__| Heure |__|__|:|__|__|

- Extubation (si réalisée AUJOURD'HUI)
  - programmée☐ (selon protocole☐ violation protocole☐)
  - auto-extubation ☐
- VNI prophylactique post-extubation OUI ☐ NON ☐
  - *Si VNI prophylactique*
  - selon protocole ☐ (TVS-TT (-)☐ âge>65ans☐ BPCO☐ Insuffisance cardiaque chronique ☐ Insuffisance respiratoire chronique☐, PaCO2>45 en fin de TVS-TT☐) violation protocole ☐
  - Durée cumulée sur les 24h : |__|__|h
  - *Si absence de VNI prophylactique*
  - selon protocole ☐ violation protocole ☐ contre-indication ☐
- VNI curative post-extubation NON ☐ selon protocole ☐ violation protocole ☐
- Optiflow post-extubation (non recommandé) OUI ☐ NON ☐
- Désencombrement bronchique : NON ☐ Toux manuellement assistée ☐
  Toux assistée instrumentale ☐

Réintubation ce jour : OUI ☐ NON ☐ : si OUI Heure : |__|__|:|__|__|
(critère(s) Neurologique☐ Hémodynamique ☐ autre ☐
Respiratoire ☐ (épuisement ☐ Encombrement ☐ Pneumopathie☐ OAP☐ Dyspnée laryngée☐))
Réintubation pour chirurgie avec intubation <24h OUI☐ NON ☐

A la fin de J90 le patient est :
Intubé ☐ Extubé ☐
Poursuite du protocole de sevrage ☐
Sortie du protocole de sevrage ☐ (remplir la partie « sortie d'essai »)
Poids du jour : |__|__|__| kg
