# Supplementary material for: Pressure support and positive end-expiratory pressure versus T-piece during spontaneous breathing trial in difficult weaning from mechanical ventilation: study protocol for the SBT-ICU study
Source: Trials. 2022 Dec 12;23:993. doi: 10.1186/s13063-022-06896-4 (PMC9742015; doi:10.1186/s13063-022-06896-4)
Supplement: Supplementary file 8 — Additional file 8. [file 13063_2022_6896_MOESM8_ESM.docx]

**Protocol Version 1**

**Pressure Support and Positive End Expiratory Pressure versus T-piece during Spontaneous Breathing Trial in difficult weaning: a randomized controlled trial. SBT-ICU study**

Document Version History

| Version Date | Version | Author | Signature | Change Description | Reason/Comment |
| --- | --- | --- | --- | --- | --- |
| 290/06/2022 | 1 | MM/JCR |  | Initial release. | Not applicable. |
|  |  |  |  |  |  |
|  |  |  |  |  |  |

**TABLE OF CONTENTS**

[LIST OF ABBREVIATIONS 3](#_Toc107487441)

[1. Study Objectives 4](#_Toc107487442)

[1.1. PRIMARY OBJECTIVE 4](#_Toc107487443)

[1.2. SECONDARY OBJECTIVES 4](#_Toc107487444)

[2. Background/Introduction 4](#_Toc107487445)

[2.1. STUDY DESIGN 4](#_Toc107487446)

[2.2. TREATMENT GROUPS 5](#_Toc107487447)

[2.3. STUDY POPULATION 5](#_Toc107487448)

[2.4. INTERVENTION 6](#_Toc107487449)

[2.5. SAMPLE SIZE 6](#_Toc107487450)

[2.6. STUDY PROCEDURE 6](#_Toc107487451)

[3. Population of Analysis 8](#_Toc107487452)

[4. Outcome Variables 8](#_Toc107487453)

[4.1. PRIMARY OUTCOME 8](#_Toc107487454)

[4.2. SECONDARY PARAMETERS OUTCOMES 8](#_Toc107487455)

[4.3. OTHER PARAMETERS 8](#_Toc107487456)

[5. Statistical Methodology 12](#_Toc107487457)

[5.1. GENERAL METHODOLOGY 12](#_Toc107487458)

[5.2. PRIMARY DATA ANALYSES 13](#_Toc107487459)

LIST OF ABBREVIATIONS

CHF: chronic heart failure

COPD: chronic obstructive pulmonary disease

ICU: intensive care unit

PEEP: positive end-expiratory pressure

SBT: spontaneous breathing trial

SBT-PS: spontaneous breathing trial with pressure support

SBT-TP: spontaneous breathing trial with T-piece

VFD: ventilator-free days

WIPO: weaning-induced pulmonary oedema

1. Study Objectives
   1. PRIMARY OBJECTIVE

Main objective: to evaluate whether if an assisted weaning strategy based on SBT-PS with PEEP followed by an SBT-TP to identify patients at high risk of extubation failure and trigger the use of prophylactic post-extubation NIV (assisted weaning strategy, intervention group) leads to a **shorter time to successful extubation** in difficult to wean patients as compared to a weaning strategy based solely on SBT-TP (unassisted weaning strategy, control group) and prophylactic post-extubation NIV based on international guidelines

- 1. SECONDARY OBJECTIVES

1. To evaluate whether if an assisted weaning strategy leads to a higher rate of first successful extubation in difficult to wean patients as compared to an unassisted weaning strategy.
2. To evaluate whether if an assisted weaning strategy leads to a shorter duration of invasive mechanical ventilation in difficult to wean patients as compared to an unassisted weaning strategy.
3. To evaluate whether if an assisted weaning strategy leads to a shorter duration of mechanical ventilation (i.e., invasive and non-invasive) in difficult to wean patients as compared to an unassisted weaning strategy.
4. To evaluate whether if an assisted weaning strategy leads to more ventilator-free days (VFD) at day-28 and at day-90, respectively, in difficult to wean patients as compared to an unassisted weaning strategy.
5. To evaluate whether if an assisted weaning strategy leads to a shorter intensive care unit stay in difficult to wean patients as compared to an unassisted weaning strategy.
6. To evaluate whether if an assisted weaning strategy leads to a shorter hospital stay in difficult to wean patients as compared to an unassisted weaning strategy.
7. To evaluate whether if an assisted weaning strategy leads to a lower mortality in intensive care unit (ICU), mortality at day-28 and mortality at day-90, respectively, in difficult to wean patients as compared to an unassisted weaning strategy.
8. To evaluate whether if an assisted weaning strategy leads to a higher reintubation rate in difficult to wean patients as compared to an unassisted weaning strategy.
9. Background/Introduction
   1. STUDY DESIGN

- The study is a monocentric prospective open labelled, randomized controlled superiority trial, with two parallel groups and balanced randomization stratified into 3 groups (according to patients prior diseases), with a 1:1 ratio, using random blocks of size 4, 6 and 8.
- Type of control : alternative strategy
- Level and method of blinding : none (open labelled study)
- Method of treatment assignment: randomization with stratification
- Number of subject : 94 patients (47 per group)
- Duration of study : 90 days
  1. TREATMENT GROUPS

In this study, two strategies of weaning are compared: “assisted strategy” (intervention group) and “unassisted strategy” (control group).

Allocation sequence will be computer generated with stratification into 3 groups:

- Patients with chronic heart failure (CHF) (defined by an ejection fraction <45%)

- Patient with COPD or chronic respiratory failure

- Other patients

Randomization was done in each stratum with random blocks of size 4, 6 and 8.

Allocation sequence was concealed in a sealed opaque envelope with sequential number in each stratum. Allocation sequence was generated by a person independent from the investigator and not enrolling nor assigning patients to intervention.

Clinicians and investigators are enrolling patients and assigning patients to intervention according to the allocation.

Due to the type of intervention, no blinding is possible for the clinicians and the patients. During the database cleaning process, the statistician will be blind of the treatment allocation.

Patients are secondary excluded of the study if:

- Patient is transferred to another ICU
- Consent withdrawal
- Severe adverse event due to the study (on clinician decision)

These patients will be replaced.

- 1. STUDY POPULATION

Inclusion criteria:

• Patient 18 years old or more

• Intubated and ventilated in intensive care unit for more than 24 hours

• Patient ready for weaning evaluation

• Failure of a first SBT-TP

Exclusion criteria:

• Chronic neuromuscular disease

• Guillain-Barré Syndrome

• Central nervous system disease with consciousness disorder (i.e inability to obey a simple command)

• Tracheostomy

• Chronic disease with life expectancy less than 1 year

• Pregnancy, breast feeding

• Withholding life support regarding a reintubation

• Prisoner or patient interned in psychiatric hospital

• Under legal protective measures

• Language barrier

• Lack of social security

• Lack of the patient's consent (or of the next of kin where appropriate)

• Patient under an exclusion period after enrolment in another research study

- 1. INTERVENTION

The interventions will be started within the 6hr following inclusion.

Intervention group (assisted weaning strategy)

While intubated and being able to perform a SBT, patients will perform a daily SBT with pressure support 7 cmH2O and PEEP 5 cmH2O during at most 30 min to assess readiness to be weaned from mechanical ventilation. Inspired oxygen fraction (FiO2) is set between 21% to 50% in order to target a peripheral oxygen saturation (SpO2) between 94 and 98% (or between 88 and 92% for chronic obstructive pulmonary disease (COPD)/chronic respiratory failure (CRF) patients). SBT success is defined by the ability to perform the SBT-PS during 30 min without failure criteria. In case of SBT failure, patient is switch back to previous respiratory parameters. In case of SBT success, an additional SBT-TP will be performed after 30 min of rest, for a maximal duration of 30 min, to further assess the risk of respiratory failure at atmospheric pressure. The modalities of the SBT-TP are the same as the control group (see below). If the patient meets extubability criteria on SBT-PS (see below), he will be extubated 2 to 3 h after resuming ventilation and will receive post-extubation NIV unless contraindicated if SBT-TP was a failure or if meeting at least one of the following post-extubation prophylactic NIV criteria: age > 65 yrs., chronic heart failure (CHF), CRF, carbon dioxide partial pressure in arterial blood (PaCO2) > 45 mmHg during SBT, or COPD.

Control group (unassisted weaning strategy)

While intubated and being able to perform a SBT (Table 2), patients will perform daily a 30 min SBT by disconnecting the patient from the ventilator and using a T-piece to administer 0 to 10 L/min oxygen targeting a peripheral oxygen saturation (SpO2) between 94 and 98% (or between 88 and 92% for COPD/CRF patients). SBT success is defined by the ability to perform the SBT-TP during 30 min without failure criteria. In case of SBT failure, patient is switch back to previous respiratory parameters. If the patient succeeds the SBT-TP and meets extubability criteria, he will be extubated 2 to 3 h after resuming ventilation and will receive post-extubation NIV if meeting post-extubation NIV criteria (cf. supra) unless contraindicated.

- 1. SAMPLE SIZE

In a pilot observational study on 88 patients, median time between first SBT and extubation was 22h with a strategy using SBT-PS and PEEP. We hypothesized that a between-arm difference of at least 24 hours would be clinically relevant to prefer a SBT strategy over one another. With an alpha risk of 5% and a bilateral hypothesis, a power of 80% and a hazard ratio of 2 (based on the ratio of median duration of time between first SBT and successful extubation), a total of 66 successful extubation would be needed. Accounting for both potential deaths before successful weaning and weaning failure (assumed to amount to 30%), we plan to include 94 patients (47 per group).

- 1. STUDY PROCEDURE

|  | Inclusion (day-1) | Day-2 to day-90 | ICU discharge / Death | Day-28 | Day-90 |
| --- | --- | --- | --- | --- | --- |
| Demographic data | X |  |  |  |  |
| Patient weaning readiness | X | X* |  |  |  |
| SBT result | X | X* |  |  |  |
| Arterial blood gas | X | X* |  |  |  |
| *If SBT success* |  |  |  |  |  |
| Cough score | X | X* |  |  |  |
| Abundancy of respiratory secretions | X | X* |  |  |  |
| Extubability criteria | X | X* |  |  |  |
| *If extubation* |  |  |  |  |  |
| Extubation date and time | X** | X** |  |  |  |
| Extubation type (protocolized/self-extubation) | X** | X** |  |  |  |
| Post-extubation NIV characteristics | X** | X** |  |  |  |
| Respiratory physiotherapy characteristics | X*** | X*** |  |  |  |
| High-flow nasal oxygen use | X | X*** |  |  |  |
| Invasive mechanical ventilation status | X | X*** |  |  |  |
| *If reintubation* |  |  |  |  |  |
| Reintubation criteria | X | X |  |  |  |
| *If tracheostomy performed* |  |  |  |  |  |
| Characteristics of tracheostomy |  |  | X |  |  |
| *Follow-up data* |  |  |  |  |  |
| Date/hour of cessation of invasive ventilation and NIV |  |  | X |  |  |
| End of study |  |  |  |  |  |
| Respiratory status |  |  | X | X | X |
| Mortality |  |  | X | X | X |
| *As long as the patient is intubated or tracheostomized  **On the day of extubation and the day after  ***during the 7 days following extubation  NIV denotes non-invasive ventilation, and SBT spontaneous breathing trial. | | | | | |

1. Population of Analysis (intention to treat population)

All subjects who were randomized except patients secondary excluded from the study due to:

- Transfer to another ICU not participating to the study
- Consent withdrawal
- Lack of inclusion criteria or presence of exclusion criteria

All the analyses (including primary efficacy analysis) will be performed on this population.

1. Outcome Variables
   1. PRIMARY OUTCOME

The primary outcome will be duration (in hours) between study inclusion and successful extubation (defined by the absence of reintubation or death within the 7 days (168 hours) following extubation). Reintubation and resuming of mechanical ventilation for less than 24h related to unplanned surgical procedure will not be considered as extubation failure. Patients extubated but exiting ICU within the 7 days following extubation will be considered as successful extubation. In patients not meeting extubation success criteria, data will be censored at day-90 or date of death whichever comes first.

- 1. SECONDARY PARAMETERS OUTCOMES

1. Rate of first successful extubation: number of patients successfully extubated after their first extubation/total number of patients per group
2. Invasive mechanical ventilation duration (expressed in hours): total cumulative time spent on invasive mechanical ventilation since inclusion per group. Each time period spent on invasive mechanical ventilation from inclusion until successful extubation are summed up. Patients not experiencing successful extubation will be censored at Day 90 or time of death (if earlier than Day 90).
3. Mechanical (invasive and non-invasive) ventilation duration (expressed in hours): total cumulative time spent on invasive and non-invasive mechanical ventilation since inclusion per group.
   - If the patient is successfully extubated with post-extubation NIV: date/time of NIV cessation minus date/time of inclusion. (or date/time of ICU discharge If NIV not ceased at ICU discharge)
   - If the patient is successfully extubated without post-extubation NIV: date/time of successful extubation minus date/time of inclusion.
   - Patients not experiencing successful extubation will be censored at Day 90 or time of death (if earlier than Day 90).
4. Ventilator-free days (VFD) at Day-28 and Day-90. VFD will be computed as follows from the day of inclusion:
   - VFD = 0 if the patient dies between inclusion and day-28 for VFD at day-28, or if the patient dies between inclusion and day-90 for VFD at day-90
   - VFD = 28-x for VFD at day-28 or VFD = 90-x for VFD at day-90 if the patient is successfully weaned from invasive mechanical ventilation, with x being the number of days from inclusion to last successful extubation. Successful weaning from mechanical ventilation will be defined as extubation without reintubation within at least 7 days (or weaning from mechanical ventilation for at least 7 days for patients with tracheostomy)
   - VFD = 0 if the patient is mechanically ventilated for more than 28 days after inclusion for VFD at day-28, or more than 90 days after inclusion for VFD at day-90
5. ICU stay (days): ICU length of stay between inclusion and ICU discharge
6. Hospital stay (days): hospital length of stay between inclusion and hospital discharge (to home or rehabilitation facility).
7. ICU, day-28, and day-90 mortality: defined as the ratio of the number of death over each period over the total number of patients per group.
8. Reintubation rate: number of patients with any reintubation divided by the number of patients per group
   1. OTHER PARAMETERS

**DEMOGRAPHY AND BASELINE**

**Collected variables**

- Randomization group: categorical variable. Intervention or control group.
- Stratification group: categorical variable. COPD or Chronic heart failure or Other
- Time of inclusion: date variable. Expressed as date (DD/MM/YYYY) and hour (HH:MM)
- Sex: categorical variable. Male or female
- Age: continuous variable. Expressed in years
- Height: continuous variable. Expressed in years, rounded to the nearest integer
- Weight: continuous variable. Expressed in kilos, with a precision of 0.5.
- Body mass index: continuous variable. Expressed in kilos, rounded to the nearest integer
- Hospital admission date: date variable. Expressed as date (DD/MM/YYYY). In case of referral from another hospital, hospital admission date is the date of admission in the previous hospital.
- ICU admission date: date variable. Expressed as date (DD/MM/YYYY). In case of referral from another ICU, ICU admission date is the date of admission in the previous ICU.
- Intubation date: date variable. Expressed as date (DD/MM/YYYY). In case of multiple intubation during ICU stay, the date corresponds the most recent intubation.
- Intubation due to respiratory failure: categorical variable (yes or no)
- Intubation due to hemodynamic failure: categorical variable (yes or no)
- Intubation due to neurological failure: categorical variable (yes or no)
- Intubation due to major surgery: categorical variable (yes or no)
- Intubation due to other reason: categorical variable (yes or no)
- Admission context: categorical variable (medical, planned surgery, emergent surgery, trauma)
- SAPSII score: continuous variable, ranging from 0 to 163
- Respiratory SOFA score, discrete variable, ranging from 0 to 4
- Hemodynamic SOFA score, discrete variable, ranging from 0 to 4
- Hepatic SOFA score, discrete variable, ranging from 0 to 4
- Renal SOFA score, discrete variable, ranging from 0 to 4
- Neurological SOFA score, discrete variable, ranging from 0 to 4
- Hematologic SOFA score, discrete variable, ranging from 0 to 4
- COPD status: categorical variable. None or suspected or proven
- Gold stage: categorical variable. Stage 1, 2,3 or 4. Only for COPD patients.
- Chronic respiratory failure: categorical variable (yes or no)
- Chronic respiratory failure type: categorical variable (obstructive, restrictive or mixed). Only for chronic respiratory failure patients.
- Chronic respiratory support: categorical variable (oxygen, NIV, both or none). Only for chronic respiratory failure patients.
- Chronic heart failure: categorical variable (yes or no)
- Charlson score: continuous variable, ranging from 0 to 43
- Baseline ventilator parameters:
  - Ventilation mode: categorical variable. Pressure support, volume controlled or other
  - Minute ventilation: continuous variable. Expressed in liters/min, with a precision of 0.1
  - Respiratory rate: continuous variable. Expressed in /min, as an integer
  - PEEP: continuous variable. Expressed in cmH2O, as an integer
  - FiO2: continuous variable. Expressed in %, as an integer
- Baseline arterial blood gas:
  - pH: continuous variable. Expressed without unit, with a precision of 0.01
  - PaCO2: continuous variable. Expressed in mmHg, as an integer
  - PaO2: continuous variable. Expressed in mmHg, as an integer
  - Bicarbonates: continuous variable. Expressed in mmol/L, as an integer
  - Base excess: continuous variable. Expressed in mmol/L, as an integer
  - Lactate: continuous variable. Expressed in mmol/L, as an integer

**Computed variables**

- Hospital length of stay at inclusion: continuous variable. Expressed in days, equal to day of inclusion minus day of hospital admission
- ICU length of stay at inclusion: continuous variable. Expressed in days, equal to day of inclusion minus day of ICU admission
- Intubation duration at inclusion: continuous variable. Expressed in days, equal to day of inclusion minus day of intubation
- Total SOFA score, computed as the sum of the respiratory, hemodynamic, hepatic, renal, neurological and hematologic SOFA, discrete variable, ranging from 0 to 24

**PROTOCOL VIOLATIONS**

Five protocol violations will be assessed, categorical variable (yes or no):

- 1. Patients not extubated despite the presence of both weaning and extubability criteria
  2. Patients extubated despite lack of weaning or extubability criteria
  3. Post-extubation NIV performed despite not required per protocol
  4. Rescue NIV for post-extubation respiratory distress (except for WIPO or hypercapnic failure for COPD/chronic respiratory failure patients)
  5. Post-extubation NIV not done despite required per protocol

**DAILY ASSESSMENTS**

These variables will be collected from Day 1 until Day 90:

- If the patient is intubated (SBT data):
  - Preliminary weaning criteria: categorical variable. Present or absent.
  - SBT result: categorical variable. Success or failure.
  - Arterial blood gas in case of SBT success:
    - pH: continuous variable. Expressed without unit, with a precision of 0.01
    - PaCO2: continuous variable. Expressed in mmHg, as an integer
    - PaO2: continuous variable. Expressed in mmHg, as an integer
    - FiO2: continuous variable. Expressed in %, as an integer. For control group, FiO2 is equal to 21+ 3*O2 flow on T-piece
  - Cough score: categorical variable. 0, 1, 2, 3, 4, 5
  - Abundance of respiratory secretions: categorical variable. 0, 1, 2, 3, 4
  - For intervention group: result of SBT-TP, categorical variable. Success or failure.
  - Summary of extubability criteria
    - Acceptable cough (cough score ≥3): categorical variable. Yes or No
    - Acceptable respiratory secretions (abundance of respiratory secretions ≤2): categorical variable. Yes or No
    - No general anaesthesia planned on the next 24h: categorical variable. Yes or No
    - No argument for laryngeal obstruction: categorical variable. Yes or No
    - Number of positive extubability criteria: numerical variable. Ranging from 0 to 4
  - Date (JJ/MM/AAAA)
  - Hour of extubation (HH:MM)
- If the patient is extubated (extubation data):
  - Patient extubated today: categorical variable. Yes or no
  - Extubation type: categorical variable. Programmed extubation per protocol; programmed extubation (protocol violation) or auto-extubation
  - Post-extubation prophylactic NIV (on the day of extubation and the following day): categorical variable. Yes or no
    - In case post-extubation prophylactic NIV:
      - Per-protocol: categorical variable. Yes or no
        - If per protocol, reason(s) for NIV

SBT-TP failure (for intervention group): categorical variable. Yes or no

Age>65 years old: categorical variable. Yes or no

COPD: categorical variable. Yes or no

Chronic heart failure: categorical variable. Yes or no

Chronic respiratory failure: categorical variable. Yes or no

PaCO2>45mmHg at the end of SBT: categorical variable. Yes or no

- - - - - Duration of NIV: continuous variable. Expressed in hours, as an integer
    - In case of no prophylactic NIV:
      - Reason: categorical variable. Per protocol, violation of protocol or contra-indication
  - Post-extubation rescue NIV (during the 7 days following extubation): categorical variable. No, per protocol, protocol violation
  - Post-extubation high-flow nasal oxygen (during the 7 days following extubation): categorical variable. No, Yes
  - Respiratory physiotherapy (during the 7 days following extubation): categorical variable. No, manual therapy, instrumental therapy
- Reintubation (reintubation data): categorical variable. Yes or no
  - Reintubation criteria: categorical variable. Neurological, Hemodynamic, other, Respiratory
  - In case of respiratory criteria reintubation, aetiology: categorical variable:
    - Respiratory exhaustion: Yes or no
    - Abundant respiratory secretion: Yes or no
    - Pneumonia: Yes or no
    - Cardiac pulmonary edema: yes or no
    - Laryngeal dyspnea: yes or no
  - Reintubation for general anaesthesia lasting less than 24h: categorical variable. Yes or no
- Status at the end of the day:
  - Respiratory status: categorical variable: yes or no
  - Weaning protocol: categorical variable: pursuit or end
  - Weight: continuous variable. Expressed in kg, with a precision of 0.5

**TERTIARY OUTCOMES**

- Tracheostomy during ICU stay: categorical variable: Yes or no
  - Time of tracheostomy: date variable. Expressed as date (DD/MM/YYYY) and hour (HH:MM)
  - Method of tracheostomy: categorical variable. Percutaneous or surgical
  - Indication(s) for tracheostomy: categorical variable:
    - Difficult weaning: yes or no
    - Swallowing disorder/abundant bronchial secretions: yes or no
    - Other: yes or no
  - Patient successfully weaned from ventilation: yes or no
    - If yes: Time of successful weaning: date variable. Expressed as date (DD/MM/YYYY) and hour (HH:MM)
- End of protocol: categorical variable. Secondary exclusion or end of protocol
  - Date of study end: date variable. Expressed as date (DD/MM/YYYY)
  - In case of secondary exclusion: categorical variable. Transfer to another ICU not participating to the study; Consent withdrawal; In case of serious adverse event, according to the clinician in charge.
  - In case of end of protocol (Cause of end of protocol): categorical variable. No reintubation in the 7 days following extubation; Withdrawing of care or withholding of reintubation decision; ICU discharge ; death ; patient still intubated at Day 90.
- Severe adverse event: categorical variable. Yes or no.
- Respiratory status at ICU discharge (Day 28, Day 90): categorical variable. Not ventilated, non-invasive ventilation (except CPAP), invasive ventilation (tracheostomy), invasive ventilation (intubated), tracheostomy (without ventilation), dead, lost to follow up
- Date of ICU discharge: date variable. Expressed as date (DD/MM/YYYY)
- Date of hospital discharge: date variable. Expressed as date (DD/MM/YYYY)
- Successful extubation: categorical variable. Yes, death or weaning failure
- Successful extubation rank. Discrete variable. NA in case of death
- Time of successful extubation: date variable. Expressed as date (DD/MM/YYYY) and hour (HH:MM).
- Number of reintubation: Discrete variable

1. Statistical Methodology
   1. GENERAL METHODOLOGY

Quantitative variables will be will be described using the following statistics: number of missing data, mean, standard deviation, quartiles, minimum and maximum values, number and rate of missing variables, and compared between groups with the Mann-Whitney test. No normality check will be performed. Qualitative variables will be reported as absolute and relative frequencies, number and rate of missing variables, and compared between groups with the chi-2 test or the Fisher exact test. Variables will be compared between both groups, except for specific analysis described in 5.2. We will not adjust p-value for multiple comparison. Univariable absolute difference will be reported for each variable as difference [95% confidence interval]. The Hodges-Lehmann method will be used to compute unbiased median differences for outcomes and their CI95%. For qualitative variables, 95% confidence interval will be computed through bootstrapping.

P-value <0,05 with a bilateral hypothesis will be considered as significant. All the analyses will be carried out using R for Windows.

**HANDLING OF MISSING DATA**

Missing data are reported as NA and will not be imputed. Number of missing observations will be reported in tables. A summary of missing observations will be included in the supplementary material.

**SENSITIVITY ANALYSES**

A sensitivity analysis will be performed for the main outcome (cf. infra).

**SUBGROUP ANALYSIS**

An exploratory analysis will be performed on the following subgroups, on the primary judgment criterion.

- - - - Randomization strata
      - COVID status (COVID positive vs COVID negative)
      - Median bicarbonates on the day of extubation (lower than median vs. higher than median)
      - PaCO_2_ at the end of SBT ≥ 45 mmHg vs < 45 mm Hg, on the day of extubation
      - Time between intubation and inclusion below vs. greater or equal to its median value
      - Cough score below vs greater or equal to its median value

**CLASSIFICATION OF PROTOCOL VIOLATION**

Protocol violations were defined in 4.3. We will report protocol violations as:

- Number of patient in interventional group with any protocol violation vs. Number of patient in control group with any protocol violation
- And then detail by type of violation (a. then b.,…), i.e. for protocol violation a. : Number of patient in interventional group with protocol violation type a. vs. number of patient in control group with protocol violation type a.
  1. PRIMARY DATA ANALYSES

5.2.1 Outcome variables

5.2.1.1 Primary outcome

Primary outcome (time to successful extubation) is subjected to competitive risk (death). However, assuming competing risk will be identical in both groups, survival analysis will be done according to a cause-specific analysis (Cox model) with randomization strata as covariate. Hence, primary outcome will be assessed with the Hazard Ratio (HR) produced by Cox model.

Time to event will be:

- For patients successfully extubated: time from inclusion to successful extubation
- For patients who died before successful extubation or Day 90: time from inclusion to death, with censoring at this time point
- For patients not weaned from mechanical ventilation at Day 90 will be censored at this time point.

Two diagnosis will be performed:

1. Proportional hazard assumption will be checked through scaled Schoenfeld residuals statistical test and graphical plot
2. Verification of influential observations: outliers will be identified by 1) plotting deviance residuals and checking symmetrical distribution around zero and 2) plotting the estimated changes in the regression coefficients upon deleting each observation in turn (dfbeta) . In case of influential observation, the observation will be checked for error, corrected if wrong, or conserved if true.

If the proportional hazard assumption is not met, a chronological variable will be used, if possible, otherwise, a log-rank test will be performed.

To account for competing risk, a sensitivity analysis will be performed on the main outcome as follows: the main outcome will be described with cumulative incidence functions and analyzed with a Fine and Gray model with successful extubation as event and death as competitive risk, with randomization strata as covariate. Proportional hazard assumption will be checked by plotting Schoenfeld residuals against event time for each covariate. If the proportional hazard assumption is not met, a chronological variable will be used, if possible.

We will provide graphical representation using a Kaplan Meier curve for the Cox model, depicting the two groups without stratification and a cumulative incidence graph for Fine and Gray model depicting the two groups without stratification, with successful extubation and death as events.

Results will be reported a table with four columns (intervention group, control group, Hazard ratio, p-value). The subgroup analysis will be presented as a forest plot.

In order to express effect size, the median value of the difference between groups in time to successful extubation will be provided (along with its 95% confidence interval).

A second statistician will carry out the analysis of the primary outcome on the dataset.

5.2.1.2 Secondary outcomes

Analysis will be carried out as described in 5.1. Results will be reported a table with four columns (intervention group, control group, univariable absolute difference with 95% confidence interval, p-value).

5.2.2 Flow-chart, baseline and demographic data

A flow-chart will describe:

- Number of patients admitted to the ICU during study period
- Number of patients with more than 24h of mechanical ventilation
- Number of patients with at least 1 SBT (screened population)
- Number of patients without inclusion criteria
- Number of patients with exclusion criteria
- Number of patients eligible (i.e with inclusion and exclusion criteria and causes of non-inclusion)
- Number of patients included and randomized
- Number of patients secondary excluded and cause of exclusion
- Number of patients included in the analysis
- Number of lost to follow-up

Baseline and demographic data will be reported in a table with two columns (intervention group, control group). No comparison will be performed for baseline and demographic characteristics since the study is randomized.

5.2.3 Protocol violations

Analysis will be carried out as described in 5.1.

5.2.4 Daily assessments

Multiple analyses will be performed (methodology described in 5.1)

1. Description of daily assessments for the successful extubation.

The population of analysis will be patients that were successfully extubated. We will report (cf. 4.3):

- - SBT data (on the day of extubation)
  - extubation data
    - Extubation type
    - Post-extubation prophylactic NIV characteristics. Duration of NIV will be computed as the sum of NIV duration on the day of extubation and the following day
    - Post-extubation rescue NIV (during the 7 days following extubation
    - Post-extubation high-flow nasal oxygen (during the 7 days following extubation)
    - Respiratory physiotherapy (during the 7 days following extubation)

Results will be reported a table with four columns (intervention group, control group, p-value).

1. Description of reintubation episodes:

The population of analysis will be patients that experienced reintubation. Since patients can experience multiple reintubations, we will report the number of patients involved and the total number of reintubation. We will not correct for repeated measures. We will report:

- Reintubation data (cf. 4.3)
- Time elapsed since extubation: continuous variable (hours), equals to date/time of reintubation minus date/time of extubation

Results will be reported a table with four columns (intervention group, control group, univariable absolute difference with 95% confidence interval, p-value).

5.2.5 Tertiary outcomes

Analysis will be carried out as described in 5.1.

Tracheostomy data will be reported a table with four columns (overall population, intervention group, control group, p-value) or in text if N<5.

We will report the following tertiary outcomes in a table with four columns (intervention group, control group, univariable absolute difference with 95% confidence interval, p-value):

- - Cause of end of protocol No reintubation in the 7 days following extubation; Withdrawing of care or withholding of reintubation decision; ICU discharge; death; patient still intubated at Day 90.
- Severe adverse event
- Respiratory status at ICU discharge (Day 28, Day 90)
- Successful extubation status
- Successful extubation rank
- Number of reintubation
