# Supplementary material for: Pressure support and positive end-expiratory pressure versus T-piece during spontaneous breathing trial in difficult weaning from mechanical ventilation: study protocol for the SBT-ICU study
Source: Trials. 2022 Dec 12;23:993. doi: 10.1186/s13063-022-06896-4 (PMC9742015; doi:10.1186/s13063-022-06896-4)
Supplement: Supplementary file 9 — Additional file 9. [file 13063_2022_6896_MOESM9_ESM.pdf]

DocuSign Envelope ID: 727A650C-2E66-4A2E-8097-4C4464BF471E

HCL HOSPICES CIVILS DE LYON | LA RECHERCHE

Direction de la Recherche en Santé

Dossier suivi par :

Nom : Anaëlle LECOMTE
Fonction : Gestionnaire SAFIP
Tél : 04 72 40 68 62
Mail : anaelle.lecomte@chu-lyon.fr

CERTIFICATE

I, Alexandre PACHOT –Director of the Clinical Research in Health Department, certify that the research project SBT-ICU:

« Impact of the combination of pressure support and positive expiratory pressure during the respiratory weaning test compared to the T-piece on the time to successful extubation »,

carried out by Doctor Mehdi MEZIDI, is receiving financial support from the Direction of Clinical Research in Health of the Hospices Civils de Lyon. The total amount allocated is : 787.50 €

Lyon, 25th Jully 2022, for all legal intents and purposes.

DocuSigned by:

E6A68104667842A...

By delegation, Caroline GAY-LOMBARD, Financial and Administrative Manager

For the Director of Clinical Research in Health, Alexandre PACHOT
