# Supplementary material for: Pressure support and positive end-expiratory pressure versus T-piece during spontaneous breathing trial in difficult weaning from mechanical ventilation: study protocol for the SBT-ICU study
Source: Trials. 2022 Dec 12;23:993. doi: 10.1186/s13063-022-06896-4 (PMC9742015; doi:10.1186/s13063-022-06896-4)
Supplement: Supplementary file 10 — Additional file 10. [file 13063_2022_6896_MOESM10_ESM.pdf]

C P P - I l e - d e - F r a n c e V I
Hospitalier Pitié-Salpêtrière Group

Research project registered In Paris, February 25, 2019
N° 3-19 HPS Cat. 2
File n°19.01.17.71710
ID-RCB : 2019-A00106-51

The committee was seized on: January 22, 2019

a request for an opinion for the research project entitled:

"Impact of the combination of inspiratory aid and positive expiratory pressure during the respiratory weaning test compared to the T-piece on the time to successful extubation"
SBT-ICU study - 69HCL18_0982

. SBT-ICU Protocol - 69HCL18_0982 of 2/7/19
. Information note and relative consent form of 2/7/19
. Information note and patient consent form of 2/7/19
. Information note and continuation consent form of 2/7/19
. List of investigators of 1/11/19

whose promoter is: **Hospices Civils de Lyon**

whose coordinator is: **Doctor Mehdi MEZIDI**

The Committee considered the information relating to this project at its meeting on: **February 20th 2019**

Participated in the deliberation :
Nathalie BRION - Therapist (T)
Laurent CAPELLE - Neurosurgeon (T)
Christophe DEMONFAUCON - Representative of approved patient associations (T)
Micheline DENANCE - Representative of the approved associations of users of the health system (S)
Jacqueline DUNO - Qualified in legal matters (S)
Marie-Hélène FIEVET - Hospital Pharmacist (T)
Cloé GIQUEL - Qualified in legal matters (S)
Christiane LOOTENS - Representative of the approved associations of patients (S)
Marie-Cécile MASURE - Hospital Psychologist (T)
Thang NGUYEN - General Practitioner (T)
Sabine PLANCOULAINE - Biostatistician (S)
Sophie TEZENAS DU MONTCEL - Biostatistician (T)
Martyna TOMCZYK - EthicalLy Qualified (S)

THE COMMITTEE ADOPTED THE FOLLOWING DELIBERATION: FAVOURABLE OPINION

Motivation: The Committee considered that the benefit/risk balance is acceptable for the subjects participating in the research.

In accordance with Article R. 1123-26 of the Public Health Code, this notice becomes null and void if the research has not started within two years.

The President of the CPP
Professor Nathalie BRION

CPP IDF VI 47, Boulevard de l'Hôpital 75013 PARIS
Phone : 01 42 16 16 83 Fax : 01 42 16 27 15
