# Supplementary material for: Pressure support and positive end-expiratory pressure versus T-piece during spontaneous breathing trial in difficult weaning from mechanical ventilation: study protocol for the SBT-ICU study
Source: Trials. 2022 Dec 12;23:993. doi: 10.1186/s13063-022-06896-4 (PMC9742015; doi:10.1186/s13063-022-06896-4)
Supplement: Supplementary file 11 — Additional file 11. [file 13063_2022_6896_MOESM11_ESM.pdf]

C P P - I l e - d e - F r a n c e V I
Hospitalier Pitié-Salpêtrière Group

Research project registered In Paris, September 30, 2019

N° 3-19 HPS Cat. 2

File n°19.01.17.71710

ID-RCB: 2019-A00106-51

Documents submitted for committee approval:

. SBT-ICU Protocol - 69HCL18_0982 of 7/18/19

. Information note and patient consent form of 6/25/19

. Information note and continuation consent form of 6/25/19

. Information note and relative consent form of 6/25/19

. Attestation by an impartial third party of patient consent of 6/25/19

The committee was seized on: July 2, 2019

a request for an opinion on the documents referenced above relating to the protocol entitled:
"Impact of the combination of inspiratory aid and positive expiratory pressure during the respiratory weaning test compared to the T-piece on the time to successful extubation" SBT-ICU study - 69HCL18_0982

whose promoter is: **Hospices Civils de Lyon**

whose coordinator is: **Doctor Mehdi MEZIDI**

The Committee considered the information relating to this project at its meeting on:

September 11, 2019

Participated in the deliberation :

Kevin BIHAN - Hospital Pharmacist (T)
Nathalie BRION - Therapist (T)
Laurent CAPELLE - Neurosurgeon (T)
Christophe DEMONFAUCON - Representative of approved patient associations (T)
Micheline DENANCE - Representative of the approved associations of users of the health system (S)
Marie GICQUEL-BENADE – Social worker (T)
Cloé GIQUEL - Qualified in legal matters (S)
Clarisse GOUDIN - Qualified in legal matters (S)
Annie LE FRANC - Representative of the approved associations of patients (S)
Esther LELLOUCHE – Nurse (T)
Christiane LOOTENS - Representative of the approved associations of patients (S)
Marie-Cécile MASURE - Hospital Psychologist (T)
Michèle MEUNIER-ROTIVAL - Genetic researcher (T)
Thang NGUYEN - General Practitioner (T)
Sophie TEZENAS DU MONTCEL - Biostatistician (T)

THE COMMITTEE ADOPTED THE FOLLOWING DELIBERATION: FAVOURABLE OPINION

The President of the CPP
Professor Nathalie BRION

CPP IDF VI 47, Boulevard de l'Hôpital 75013 PARIS
Phone : 01 42 16 16 83 Fax : 01 42 16 27 15
