# Supplementary material for: Pressure support and positive end-expiratory pressure versus T-piece during spontaneous breathing trial in difficult weaning from mechanical ventilation: study protocol for the SBT-ICU study
Source: Trials. 2022 Dec 12;23:993. doi: 10.1186/s13063-022-06896-4 (PMC9742015; doi:10.1186/s13063-022-06896-4)
Supplement: Supplementary file 12 — Additional file 12. [file 13063_2022_6896_MOESM12_ESM.pdf]

Opinion of the Committee for the protection of persons

Committee for the protection of persons Ile de France VI on Substantial Amendment

CPP Information

Name of the CPP: Committee for the Protection of Persons Ile de France VI
Address: Pitié-Salpêtrière Hospital Group - 4 Force building - 47, boulevard de l'Hôpital 75013 PARIS France
Email: cppidf6.salpetriere@yahoo.fr

Promoter information

Organisation : Hospices Civils de Lyon
Full name : PACHOT Alexandre

Investigator

Investigator : Dr Mehdi MEZIDI

File information

SI Number : 19.01392.000319-MS02
National number: 2019-A00106-51
Internal reference : 69HCL18_0982
Regulations: Jardé Law
Research qualification: Category 2
Product or procedure: Excluding health products (products not mentioned in article L.5311-11 of the public health code)
Title: SBT-ICU study - Impact of the combination of inspiratory aid and positive expiratory pressure during the respiratory weaning test compared to the T-piece on the time to successful extubation
MS Number: 2
Reason for the MS : - Extension of the recruitment period
- Update od the promoter’s contact details
- Update archiving duration

Summary of previous opinions

File	Opinion	Date of issue
19.01392.000319	*Favourable*	09/16/2021

This file was studied in session on 10/13/2021.

The committee adopted the following deliberation:

Favourable Opinion

Persons having deliberated

College	Category	Full name
College I	Hospital Pharmacist	BIHAN Kevin
College I	Hospital Pharmacist	FIEVET Marie-Hélène
College I	RIPH Qualification - Other	BRION Nathalie
College I	General medicine specialist	NGUYEN Thang
College I	medical assistant	LELLOUCHE Esther
College I	RIPH Qualification - Biostatistics or epidemiology	TEZENAS du MONTCEL Sophie
College I	RIPH Qualification - Other	CAPELLE Laurent
College I	RIPH Qualification - Biostatistics or epidemiology	PLANCOULAINE Sabine
College II	Ethical competence	MEYOHAS Marie-Caroline
College II	Representative of approved association	LOOTENS Christiane
College II	Representative of approved association	LE FRANC Annie
College II	Legal competence	DUNO Jacqueline
College II	Representative of approved association	DENANCE Micheline
College II	Ethical competence	TOMCZYK Martyna
College II	Representative of approved association	DEMONFAUCON Christophe
College II	Legal competence	GOUDIN Clarisse
College II	Competence in human and social sciences or social action	MASURE Marie-Cécile

Documents analyzed by the CPP

Categorization	Entitled	Deposit date
ASS - Insurance	*2019-A00106-51_assurance_DMS2_20210917_SBT-ICU.pdf*	09/17/2021
Substantial change request letter	*2019-A00106-51_Courrier_DMS2_20210917_SBT-ICU.pdf*	09/17/2021
MS Application Form	*2019-A00106-51_FAEC_DMS2_v5_20210917_SBT-ICU.pdf*	09/17/2021
MS Application Form	*2019-A00106-51_demande_DMS2_20210917_SBT-ICU.pdf*	09/17/2021
INF - Information Document	*2019-A00106-51_Attestation témoin _V2_20210907_SBT-ICU_ama.pdf*	09/17/2021
INF - Information Document	*2019-A00106-51_NIFC patient _V4_20210907_SBT-ICU_ama.pdf*	09/17/2021

INF - Information Document	*2019-A00106-51_NIFC proche _V3_20210907_SBT-ICU_ama.pdf*	09/17/2021
INF - Information Document	*2019-A00106-51_NIFC poursuite _V4_20210907_SBT-ICU_ama.pdf*	09/17/2021
Information justifying the merits of each modification	*2019-A00106-51_Information justifiant le bien fondé_DMS2_20210917_SBT-ICU.pdf*	09/17/2021
PRO - Protocol	*2019-A00106-51_Protocole _V5_20210907_SBT-ICU_ama.pdf*	09/17/2021
RES - Summary	*2019-A00106-51_Résumé _V5_20210907_SBT-ICU_ama.pdf*	09/17/2021
Comparative table highlighting the changes made in the original application documents	*2019-A00106-51_Tableau comparatif des modifications_V2_20210907_SBT-ICU.pdf*	09/17/2021
Summary table of all MS and MNS that have occurred since the initial request	*2019-A00106-51_Tableau récapitulatif des MS et MNS_20210917_SBT-ICU.pdf*	09/17/2021

Done in Paris on October 27, 2021

The President of the CPP

Professor Nathalie BRION
