# Supplementary material for: Pressure support and positive end-expiratory pressure versus T-piece during spontaneous breathing trial in difficult weaning from mechanical ventilation: study protocol for the SBT-ICU study
Source: Trials. 2022 Dec 12;23:993. doi: 10.1186/s13063-022-06896-4 (PMC9742015; doi:10.1186/s13063-022-06896-4)
Supplement: Supplementary file 14 — Additional file 14. [file 13063_2022_6896_MOESM14_ESM.pdf]

Direction de la Recherche en Santé

NOTE D'INFORMATION A L'ATTENTION *DES PATIENTS POUR LA POURSUITE DE LA RECHERCHE*

Impact de la combinaison de l'aide inspiratoire et de la pression expiratoire positive pendant l'épreuve de sevrage respiratoire en comparaison de la pièce en T sur le délai jusqu'à l'extubation avec succès

SBT-ICU

Version 4 du 07/09/2021

Promoteur :

Hospices Civils de Lyon
BP 2251
3 quai des Célestins,
69229 LYON cedex 02

Investigateur coordonnateur:

Dr Mehdi MEZIDI
Réanimation Médicale
Hôpital de la Croix-Rousse — GHN
103 Grande Rue de la Croix-Rousse 69317 Lyon CEDEX 04
Tél : 04.26.10.92.75
Email : mehdi.mezidi@chu-lyon.fr

Madame, Monsieur,

En raison de la gravité de votre état, l'investigateur était dans l'impossibilité de vous demander votre consentement au préalable et vous avez été inclus dans le protocole de recherche clinique SBT-ICU le |___|___| / |___|___|/|___|___|___|___|, après consentement d'un de vos proches.

Ou, en raison de l'impossibilité de recueillir votre consentement écrit, nous avons recueilli votre accord oral à la participation à l'étude SBT-ICU devant un témoin impartial le |___|___| / |___|___|/|___|___|___|___|

Conformément au ***Code de la Santé Publique, article L1122-1*** *régissant les recherches impliquant la personne humaine, c'est à un membre de votre famille ou à un proche ou à une personne de confiance qu'a été demandé l'accord de votre participation à cette recherche.*

Vous êtes désormais apte à comprendre et à exprimer pleinement votre volonté. C'est pourquoi l'investigateur vous a présenté l'étude SBT-ICU et sollicite votre accord pour que vous poursuiviez votre participation à cette ***étude****.*

Ce document a pour objectif de vous donner toutes les informations relatives à cette étude de façon à vous permettre d'exercer au mieux votre liberté de décision. Ce document est obligatoire et son contenu est défini par la loi (Code de la Santé Publique, article L 1122-1). Il décrit précisément l'étude et mentionne toutes les autorisations réglementaires obtenues pour sa mise en œuvre.

Avant de prendre une décision, il est important que vous lisiez attentivement ces pages qui vous apporteront les informations nécessaires concernant les différents aspects de cette ***étude****. Vous devez conserver ce document. N'hésitez pas à poser des questions si vous ne comprenez pas certains éléments.*

La signature du formulaire de consentement devenue obligatoire par l'application du code de la Santé Publique (livre I, titres 2 et 3 du CSP), n'affecte aucunement vos droits légaux.

La poursuite de votre participation est entièrement volontaire. Si vous ne désirez pas poursuivre cette ***étude****, vous continuerez à bénéficier de la meilleure prise en charge médicale possible, conformément aux connaissances actuelles.*

Pourquoi cette recherche?

Vous respirez actuellement (ou avez respiré) grâce au ventilateur de réanimation qui est connecté à la sonde d'intubation car votre état le nécessite(ait). Comme votre état de santé s'est amélioré, nous avons envisagé/ envisageons de vous séparer du ventilateur (sevrage). Afin d'être sûr que vous soyez capable de respirer par vous-même, nous avons/allons réaliser quotidiennement des épreuves de sevrage. Ces dernières consistent à mimer les conditions que vous rencontreriez si vous étiez extubé (sans sonde d'intubation) et estimer si vous avez les forces nécessaires pour cela.

Actuellement, plusieurs stratégies de sevrage existent :

- Une stratégie de sevrage « assisté » où l'on maintient un faible niveau d'aide lors de l'épreuve de sevrage.
- Une stratégie de sevrage « non assistée » où aucune aide n'est délivrée lors du sevrage.

Ces deux stratégies sont appliquées couramment dans les réanimations françaises et européennes.

Afin de déterminer si une stratégie permet d'extuber plus rapidement et avec succès les patients, il est nécessaire de comparer ces deux stratégies. C'est le but de cette étude, car à ce jour il n'existe pas de preuve de la supériorité d'une stratégie par rapport à l'autre. Or il est très important pour les patients qui sont dans le même état de santé que vous, que nous puissions les extuber le plus rapidement possible afin d'éviter les complications liées à une intubation prolongée.

Si cette étude montre qu'une stratégie est meilleure, elle permettra alors de faire bénéficier à nos futurs patients de durées d'intubation plus courtes.

Quel est l'objectif de cette recherche?

L'objectif de cette étude est de déterminer si une stratégie de sevrage respiratoire permet d'extuber plus rapidement et avec succès les patients en comparaison à l'autre stratégie.

Les deux stratégies étudiées sont utilisées couramment en réanimation :

- Stratégie « assistée » : lors des épreuves de sevrage, vous restez connecté au respirateur avec de faibles niveaux d'aide respiratoire. Si votre état le nécessite, vous recevrez un support respiratoire après l'extubation via un masque de ventilation (ventilation non-invasive)
- Stratégie « non-assistée » : lors des épreuves de sevrage, vous êtes déconnecté du ventilateur et respirez seulement à travers la sonde d'intubation. Si votre état le nécessite, vous recevrez un support respiratoire après l'extubation via un masque de ventilation (ventilation non-invasive)

Afin de pouvoir comparer ces deux stratégies, il est nécessaire de tirer au sort la stratégie réalisée. Ainsi, dès que le tirage au sort a été réalisé, vous avez réalisé ou réaliserez quotidiennement le test de sevrage déterminé. Si vous êtes dans le groupe assisté, et que vous avez réussi ou réussissez l'épreuve de sevrage « assistée », vous avez réalisé ou réaliserez aussi une épreuve de sevrage « non assistée » pour déterminer si vous aviez ou avez besoin de ventilation non-invasive après votre extubation.

Quelle est la méthodologie de cette recherche ?

Après l'information de votre proche, le recueil de l'accord de votre proche, *ou* après vous avoir informé et recueilli votre accord oral devant un tiers impartial, et le tirage au sort, l'étude a débuté. Le protocole de sevrage se finit au maximum 90 jours après le début de l'étude. Le protocole de sevrage peut se finir avant J90 si vous êtes extubé avant.

Les patients inclus dans cette étude sont les patients qui n'ont pas réussi leur première épreuve de sevrage (non-assistée).

Environ 94 patients participeront à l'étude qui se déroule dans le service de réanimation médicale de l'Hôpital de la Croix Rousse.

Il n'est pas possible de participer à d'autres études portant sur le sevrage respiratoire pendant la durée de cette étude.

Comment va se dérouler cette recherche (ou comment elle a débuté)?

L'étude a commencé après l'information de votre proche par l'investigateur, le recueil du consentement de votre proche ou après vous avoir informé et recueilli votre accord oral devant un tiers impartial et après le tirage au sort.

Le premier jour de l'étude, et les jours suivants, tant que vous étiez ou êtes intubé et apte au sevrage, était réalisé ou est réalisé un test de sevrage respiratoire dans les 2 groupes de l'étude. Si vous êtes dans le groupe assisté, si vous réussissiez ou réussirez l'épreuve de sevrage « assistée », vous réalisiez ou réaliserez aussi une épreuve de sevrage « non assistée » pour déterminer si vous aviez ou avez besoin de ventilation non-invasive après votre extubation. Si vous êtes dans le groupe non assisté, un seul test de sevrage respiratoire aura été ou sera effectué, et la ventilation non invasive aura été ou sera utilisée en fonction des recommandations internationales.

Une fois extubé, le protocole de sevrage s'arrêtait ou s'arrête sauf si vous étiez ou êtes de nouveau intubé dans les sept jours suivants.

Nous avons vérifié ou vérifierons votre état de santé systématiquement le jour de la sortie de réanimation, à J28 et J90.
Si vous décidez d'arrêter votre participation à l'étude, vous serez pris en charge selon les habitudes du service.

Quels sont les bénéfices, les risques et les contraintes liés à votre participation ?

Bénéfices :
Cette étude permettra d'améliorer les connaissances sur le sevrage respiratoire des patients de ventilation. Si l'étude est positive, la stratégie assistée permettra aux futurs patients de bénéficier d'une réduction du temps passé sous ventilation mécanique.
Risques :
Les patients reçoivent deux stratégies utilisées quotidiennement dans les services de réanimation. Il n'est pas attendu de risques supplémentaires liés à cette étude.

Au total, le rapport bénéfices/risques ne paraît pas défavorable.
En cas de survenue d'un évènement indésirable grave, le médecin vous prenant en charge pourra arrêter l'étude s'il pense que cet évènement est dû à l'étude.

Quelles sont les éventuelles alternatives médicales?

En dehors du cadre de l'étude, vous recevriez l'une des deux stratégies selon les habitudes du service où vous êtes hospitalisé.
Seul le tirage au sort de la stratégie de sevrage est spécifique à cette recherche.

Quels sont vos droits ?

- ***Participation volontaire***

Vous êtes entièrement libre d'accepter ou de refuser de poursuivre votre participation à cette recherche sans que cela ne modifie la qualité des soins auxquels vous avez droit, ou les relations existant avec votre médecin ou l'investigateur.

Si vous décidez de poursuivre votre participation à cette recherche, mais que vous changiez d'avis au cours de celle-ci, vous pouvez à tout moment demander d'interrompre votre participation à l'étude sans aucun préjudice, sans justification de votre part et sans que votre responsabilité ne soit engagée. Dans ce cas, vos données recueillies jusque-là seront utilisées dans les résultats de l'étude.
D'autre part, s'il le juge nécessaire pour votre bien, l'investigateur pourra modifier votre suivi et vous pourrez continuer à bénéficier pleinement de sa **compétence.**

Plus généralement, votre participation à cette étude ne décharge en aucune façon le promoteur et les investigateurs de leurs devoirs envers vous.

A tout moment, toutes les informations que vous souhaiteriez obtenir ultérieurement concernant cette recherche vous seront communiquées dans la mesure du possible par votre médecin et/ou par l'investigateur. Vous serez tenu informé de toute nouvelle donnée importante concernant l'étude à laquelle vous acceptez de participer
L'investigateur, tout comme le promoteur, peut interrompre à tout moment votre participation à l'étude s'il juge que cela est dans votre intérêt, ou arrêter l'étude dans sa globalité pour des raisons médicales, administratives ou autres.
Pour pouvoir participer à cette étude, vous devez nécessairement être affilié à un régime d'assurance maladie telle que celui de la sécurité sociale.

Durant toute votre participation à cette recherche, il vous sera demandé de ne pas participer à une autre étude qui pourrait interférer avec les résultats du présent protocole de recherche.

Tous les frais médicaux liés à l'étude seront à la charge du promoteur. Il n'y aura pas de coût supplémentaire pour vous. Vous ne serez pas rémunéré(e) du fait de la participation à l'étude.

- ***Confidentialité et protection des données***

Dans le cadre de la recherche interventionnelle à risques et contraintes minimes à laquelle les Hospices Civils de Lyon vous proposent de poursuivre votre participation, un traitement informatique de vos données personnelles a été mis en œuvre pour permettre d'analyser les résultats de la recherche au regard de l'objectif de cette dernière. Le responsable du traitement des données est le promoteur, dont les coordonnées figurent sur la première page de ce document. Ce traitement des données a pour fondement juridique l'article 6 du Règlement Général sur la Protection des Données (RGPD) à savoir l'exécution d'une mission d'intérêt public dont est investi le responsable de traitement et les intérêts légitimes poursuivis par lui. De plus, au titre de l'article 9 du RGPD le responsable de traitement peut de manière exceptionnelle traiter des catégories particulières de données, incluant des données de santé notamment à des fins de recherche scientifique.

Pour l'analyse, les données médicales vous concernant seront transmises aux Hospices Civils de Lyon ou aux personnes ou sociétés agissant pour son compte, en France ou à l'étranger. Ces données seront identifiées par un code et/ou vos initiales. Ces données pourront également, dans des conditions assurant leur confidentialité, être transmises aux autorités de santé françaises ou étrangères et à d'autres entités en dehors des Hospices Civils de Lyon.

Les données seront transférées et collectées conformément à la méthodologie de référence MR001 de la Commission Nationale de l'Informatique et des Libertés (CNIL) pour laquelle les Hospices Civils de Lyon ont signé un engagement de conformité. Conformément à la réglementation française et européenne, les données de l'étude seront conservées 15 ans.

Par ailleurs, sauf opposition expresse de votre part adressée à l'investigateur *coordonnateur* dont les coordonnées figurent sur la première page de ce document, vos données recueillies dans le cadre de cette étude pourront être transmises ailleurs dans le monde et réutilisées par des partenaires publics ou privés lors de recherches ultérieures exclusivement à des fins scientifiques.

Si vous avez des questions ou des réclamations au sujet du traitement de vos données au cours de cette étude, vous pouvez contacter le DPO par voie électronique : dpo@chu-lyon.fr ou par courrier postal :
Le délégué à la protection des données
162 avenue Lacassagne
Bâtiment A – 3e étage – Bureau 316
69003 LYON
Si vous estimez, après avoir contacté le DPO des HCL, que vos droits sur vos données ne sont pas respectés, vous pouvez adresser une réclamation (plainte) à la CNIL :
https://www.cnil.fr/fr/webform/adresser-une-plainte

- **Exercer vos droits**

Vous pourrez également, à tout moment, exercer votre droit d'accès, de vérification, de correction, de limitation et d'opposition au traitement et à la transmission des données vous concernant en en faisant la demande auprès du médecin de votre choix ou auprès d'un investigateur de l'étude. Si vous souhaitez exercer votre droit à l'effacement de vos données, le responsable de traitement peut au titre des Articles 17.3.c et 17.3.d. du RGPD ne pas faire droit à cette demande si celle-ci est susceptible de rendre impossible ou de compromettre gravement la réalisation des objectifs de la recherche. Ainsi, vos données recueillies préalablement au retrait de votre consentement pourront ne pas être effacées et pourront continuer à être traitées dans les conditions prévues par la recherche.

Si les résultats de cette étude devaient être présentés dans des communications et/ou des publications scientifiques médicales, l'identité des participants n'apparaîtra d'aucune façon.

A l'issue de l'étude, les résultats globaux de la recherche pourront vous être communiqués sur simple demande auprès de l'investigateur coordonnateur de l'étude, le Dr MEZIDI. La base de données de l'étude rendue totalement anonyme pourra être transmise à d'autres chercheurs qui travailleraient sur le même sujet.

- ***Dispositions réglementaires***

Le Comité de Protection des Personnes Ile de France VI *(*47 Boulevard de l'Hôpital, 75013 PARIS*)* a émis un avis favorable à la réalisation de cette étude le 20/02/2019. Enfin, cette recherche respecte le règlement général sur la protection des données.
Le promoteur de cette recherche, les Hospices Civils de Lyon, BP 2251, quai des célestins, 69229 Lyon cedex 02, a souscrit une assurance de responsabilité civile auprès de la Société Hospitalière d'Assurance Mutuelle, 18 rue Edouard Rochet, 69008 Lyon, sous le numéro 159.077*.*
Les personnes ayant subi un préjudice après participation à une *recherche interventionnelle à risques et contraintes minimes* peuvent faire valoir leurs droits auprès de l'assureur du promoteur.

L'investigateur doit vous fournir toutes les explications nécessaires concernant cette recherche. Vous avez le droit d'arrêter votre participation à quel que moment que ce soit, et quel que soit le motif ; vous continueriez à bénéficier du suivi médical et cela n'affectera en rien votre surveillance future.

Qui pouvez-vous contacter pour toute question ?
Si vous avez des questions concernant l'étude, n'hésitez pas à nous les poser. Nous pouvons vous donner les informations complémentaires que vous souhaitez. Les noms et numéros de téléphone des personnes à contacter sont les suivants :

Investigateur coordonnateur de l'étude

Dr Mehdi MEZIDI
Réanimation Médicale
Hôpital de la Croix-Rousse — GHN
103 Grande Rue de la Croix-Rousse 69317 Lyon CEDEX 04
Tél : 04.26.10.92.75
Email : mehdi.mezidi@chu-lyon.fr

Investigateur de votre centre référent pour l'étude

Titre Prénom NOM : ……………………………………………………………………………………………
Service : ……………………………………………………………………………………………
Hôpital/Groupement : ……………………………………………………………………………………………..
Adresse : ……….
Tél : …………………………………

Nous vous remercions de l'attention que vous avez portée à la lecture de cette notice. Une copie de ce document vous sera remise pour que vous puissiez bénéficier de l'ensemble des informations concernant votre participation à l'étude.

Lorsque vous aurez lu cette note d'information, il vous sera proposé, si vous êtes d'accord, de donner votre consentement écrit en signant le formulaire préparé à cet effet.

Direction de la Recherche en Santé	**FORMULAIRE DE CONSENTEMENT**<br>***DES PATIENTS POUR LA POURSUITE DE LA RECHERCHE***

La loi 2012-300 du 5 mars 2012 relative aux recherches impliquant la personne humaine rend obligatoire le recueil dès que possible de l'accord écrit des patients pour la poursuite d'une recherche interventionnelle ou recherche interventionnelle à risques et contraintes minimes initiée en situation d'urgence. C'est un tel accord qui vous est demandé ci-dessous, pour continuer de participer à l'étude intitulée :

Impact of the combination of pressure support and positive end-expiratory pressure during spontaneous breathing trial versus T-piece on the time to successful extubation

SBT-ICU

Promoteur : Hospices Civils de Lyon
BP 2251
3 quai des Célestins,
69229 LYON cedex 02

Investigateur coordonnateur :

Dr Mehdi Mezidi
Réanimation Médicale
Hôpital de la Croix-Rousse — GHN
103 Grande Rue de la Croix-Rousse 69317 Lyon CEDEX 04
Tél : 04.26.10.92.75
Email : mehdi.mezidi@chu-lyon.fr

Je soussigné(e) …… *(nom, prénom)* certifie avoir lu et compris la note d'information qui m'a été remise.

J'ai eu la possibilité de poser toutes les questions que je souhaitais au *Pr/Dr* .... *(nom, prénom)* qui m'a expliqué la nature, les objectifs, les risques potentiels et les contraintes liées à ma participation à cette recherche.

Je connais la possibilité qui m'est réservée d'interrompre ma participation à cette recherche à tout moment sans avoir à justifier ma décision et je ferai mon possible pour en informer l'investigateur qui me suit dans la recherche. Cela ne remettra naturellement pas en cause la qualité des soins ultérieurs.

J'ai eu l'assurance que les décisions qui s'imposent pour ma santé seront prises à tout moment, conformément à l'état actuel des connaissances médicales.

J'ai bien compris que l'investigateur peut interrompre à tout moment ma participation à l'essai s'il le juge nécessaire.

Je suis informé(e) de la possibilité que mes données recueillies dans le cadre de cette étude puissent être réutilisées lors de recherches ultérieures exclusivement à des fins scientifiques et que je peux m'y opposer.

J'ai bien noté / été informé que cette recherche a reçu l'avis favorable du Comité de Protection des Personnes Ile de France VI le 20/02/2019 et a fait l'objet d'une déclaration à la Commission Nationale Informatique et Libertés (CNIL).

J'ai bien noté que cette recherche est menée conformément aux articles L1121-1 et suivants du Code de la Santé Publique, relatifs à la protection des personnes qui se prêtent à des recherches impliquant la personne humaine et conformément à la règlementation en vigueur.

Je certifie sur l'honneur être affilié à un régime de sécurité sociale ou bénéficiaire d'un tel régime.

Le promoteur de la recherche, les Hospices civils de Lyon, BP 2251, quai des célestins, 69229 Lyon cedex 02 a souscrit une assurance de responsabilité civile en cas de préjudice auprès de de la Société Hospitalière d'Assurance Mutuelle, 18 rue Edouard Rochet, 69008 Lyon, sous le numéro 159.077.

J'accepte que les personnes qui collaborent à cette recherche ou qui sont mandatées par le promoteur, ainsi qu'éventuellement le représentant des Autorités de Santé, aient accès à l'information contenue dans mon dossier médical dans le respect le plus strict de la confidentialité.

J'ai bien noté que, conformément aux dispositions de la loi relative à l'informatique, aux fichiers et aux libertés, je dispose d'un droit d'accès , de rectification de vérification, de correction et d'opposition à la transmission de mes données couvertes par le secret professionnel susceptibles d'être utilisées dans le cadre de cette recherche et d'être traitées. Ces droits s'exercent auprès de l'investigateur qui me suit dans le cadre de cette recherche et qui connaît mon identité.

J'ai été avisé qu'aucune indemnité n'est prévue pour ma participation à cette recherche.

Mon consentement ne décharge en rien l'investigateur et le promoteur de la recherche de leurs responsabilités à mon égard. Je conserve tous les droits garantis par la loi.

Les résultats globaux de la recherche me seront communiqués directement, si j'en fais la demande, conformément à la loi du 4 mars 2002 relative aux droits des malades et à la qualité du système de santé.
Je peux à tout moment demander des informations complémentaires au Dr/Pr …………………………………………..

Deux exemplaires originaux de ce formulaire de consentement ont été établis : un m'a été remis, l'autre a été remis à l'investigateur et sera conservé au minimum 15 ans après la fin de la recherche.

- **<u>Patient donnant son consentement :</u>**

Ayant disposé d'un temps de réflexion suffisant avant de prendre ma décision, j'accepte librement et volontairement :

- **de continuer à participer à la recherche *SBT-ICU***

NOM, Prénom du patient à la recherche : …….

Fait à : ……………………………………………………., le |___|___| / |___|___| / |___|___|___|___|

Signature du patient :

- **<u>Investigateur obtenant le consentement :</u>**

J'atteste que toutes les obligations liées à un consentement éclairé ont été satisfaites dans le cadre de ce projet de recherche clinique – que le participant a reçu une information relative à ses droits, que nous avons discuté de ce projet et que je lui ai expliqué en termes compréhensibles l'ensemble des informations contenues dans la notice. Je certifie également avoir laissé le participant me poser toutes les questions qu'il souhaitait et y avoir répondu.

NOM, Prénom de l'investigateur : …….

Fait à : ……………………………………………………., le |___|___| / |___|___| / |___|___|___|___|

Signature de l'investigateur :
