# Supplementary material for: Pressure support and positive end-expiratory pressure versus T-piece during spontaneous breathing trial in difficult weaning from mechanical ventilation: study protocol for the SBT-ICU study
Source: Trials. 2022 Dec 12;23:993. doi: 10.1186/s13063-022-06896-4 (PMC9742015; doi:10.1186/s13063-022-06896-4)
Supplement: Supplementary file 16 — Additional file 16. [file 13063_2022_6896_MOESM16_ESM.pdf]

Direction de la Recherche en Santé	**ATTESTATION PAR UN TIERS IMPARTIAL DU CONSENTEMENT PATIENT**

Impact de la combinaison de l'aide inspiratoire et de la pression expiratoire positive pendant l'épreuve de sevrage respiratoire en comparaison de la pièce en T sur le délai jusqu'à l'extubation avec succès

Etude SBT-ICU

Investigateur coordonnateur : Dr Mehdi MEZIDI
Coordonnées : Réanimation Médicale
Hôpital de la Croix-Rousse — GHN
103 Grande Rue de la Croix-Rousse
69317 Lyon CEDEX 04
Tél : 04.26.10.92.75

Promoteur : Hospices Civils de Lyon
Coordonnées : Direction de la recherche en Santé
(DRS) – Hospices Civils de Lyon – BP 2251, 3 quai des Célestins,
69229 LYON cedex 02

Je soussigné(e),

Nom – prénom : ____

Fonction : ____

Hôpital : ____

Atteste que Mr / Mme ____ *(Nom – prénom du patient)*

- a bien été informé(e) par le Dr/Pr___________________________________ *(Nom – prénom)* le /___/___/______/ (jj/mm/aaaa) de l'objet, des objectifs, du déroulement, de la durée, des contraintes, des bénéfices et risques potentiels, de tous les aspects légaux, relatifs à l'étude SBT-ICU,
- que la notice d'information de l'étude SBT-ICU lui a été lue de manière complète préalablement à son inclusion,
- qu'il/elle a manifestement compris les informations reçues,
- qu'il/elle a donné son accord oral pour participer à cette étude,
- qu'il/elle a été inclus suite à cet accord.

Date : /___/___/______/ (jj/mm/aaaa)　　　　　　　　　　Signature :
